# Large Language Model–Assisted Risk-of-Bias Assessment in Randomized Controlled Trials Using the Revised Risk-of-Bias Tool: Evaluation Study

**DOI:** 10.2196/70450

**Published:** 2025-06-24

**Authors:** Jiajie Huang, Honghao Lai, Weilong Zhao, Danni Xia, Chunyang Bai, Mingyao Sun, Jianing Liu, Jiayi Liu, Bei Pan, Jinhui Tian, Long Ge

**Affiliations:** 1 Department of Health Policy and Management School of Public Health Lanzhou University Lanzhou China; 2 Evidence-Based Social Science Research Center School of Public Health Lanzhou University Lanzhou China; 3 School of Nursing Southern Medical University Guangzhou China; 4 School of Nursing Peking University Beijing China; 5 College of Nursing Gansu University of Traditional Chinese Medicine Lanzhou China; 6 Evidence-Based Medicine Center School of Basic Medical Sciences Lanzhou University Lanzhou China; 7 Key Laboratory of Evidence Based Medicine of Gansu Province Lanzhou China

**Keywords:** systematic review, large language models, risk of bias 2, artificial intelligence, efficiency

## Abstract

**Background:**

The revised Risk-of-Bias tool (RoB2) overcomes the limitations of its predecessor but introduces new implementation challenges. Studies demonstrate low interrater reliability and substantial time requirements for RoB2 implementation. Large language models (LLMs) may assist in RoB2 implementation, although their effectiveness remains uncertain.

**Objective:**

This study aims to evaluate the accuracy of LLMs in RoB2 assessments to explore their potential as research assistants for bias evaluation.

**Methods:**

We systematically searched the Cochrane Library (through October 2023) for reviews using RoB2, categorized by interest in adhering or assignment. From 86 eligible reviews of randomized controlled trials (covering 1399 RCTs), we randomly selected 46 RCTs (23 per category). In addition, 3 experienced reviewers independently assessed all 46 RCTs using RoB2, recording assessment time for each trial. Reviewer judgments were reconciled through consensus. Furthermore, 6 RCTs (3 from each category) were randomly selected for prompt development and optimization. The remaining 40 trials established the internal validation standard, while Cochrane Reviews judgments served as external validation. Primary outcomes were extracted as reported in corresponding Cochrane Reviews. We calculated accuracy rates, Cohen κ, and time differentials.

**Results:**

We identified significant differences between Cochrane and reviewer judgments, particularly in domains 1, 4, and 5, likely due to different standards in assessing randomization and blinding. Among the 20 articles focusing on adhering, 18 Cochrane Reviews and 19 reviewer judgments classified them as “High risk,” while assignment-focused RCTs showed more heterogeneous risk distribution. Compared with Cochrane Reviews, LLMs demonstrated accuracy rates of 57.5% and 70% for overall (assignment) and overall (adhering), respectively. When compared with reviewer judgments, LLMs’ accuracy rates were 65% and 70% for these domains. The average accuracy rates for the remaining 6 domains were 65.2% (95% CI 57.6-72.7) against Cochrane Reviews and 74.2% (95% CI 64.7-83.9) against reviewers. At the signaling question level, LLMs achieved 83.2% average accuracy (95% CI 77.5-88.9), with accuracy exceeding 70% for most questions except 2.4 (assignment), 2.5 (assignment), 3.3, and 3.4. When domain judgments were derived from LLM-generated signaling questions using the RoB2 algorithm rather than direct LLM domain judgments, accuracy improved substantially for Domain 2 (adhering; 55-95) and overall (adhering; 70-90). LLMs demonstrated high consistency between iterations (average 85.2%, 95% CI 85.15-88.79) and completed assessments in 1.9 minutes versus 31.5 minutes for human reviewers (mean difference 29.6, 95% CI 25.6-33.6 minutes).

**Conclusions:**

LLMs achieved commendable accuracy when guided by structured prompts, particularly through processing methodological details through structured reasoning. While not replacing human assessment, LLMs demonstrate strong potential for assisting RoB2 evaluations. Larger studies with improved prompting could enhance performance.

## Introduction

Systematic reviews (SRs) are important for summarizing all relevant published evidence on a specific research question. They play a key role in developing guidelines and making health care decisions [1]. During the conduct of a systematic review, assessing the risk of bias (RoB) in the included primary studies is essential, as it allows the identification of potential flaws and assessment of the internal validity of the review’s results [2]. The Cochrane RoB tool is the most widely used for assessing RoB in randomized controlled trials (RCTs) in both Cochrane and non-Cochrane SRs [3]. In 2019, the revised version of this tool RoB2 was released to address limitations of the previous version, such as inconsistent domain use and the lack of an overall judgment domain. RoB2 evaluates potential biases from the randomization process, deviations from intended interventions, missing data, measurement of the outcomes, selection of the reported results, and overall bias. For each domain, RoB2 guides the reviewer in formulating a judgment on the RoB, which can be expressed as “low,” “some concern,” or “high.”

However, the study by Minozzi et al [4] demonstrated low interrater reliability (IRR) of RoB2, indicating significant challenges in its application. Further experiments were subsequently conducted to evaluate the reliability of RoB2, with the findings suggesting that the observed discrepancies in assessment might be attributed to a lack of sufficient subject matter expertise. This knowledge gap is particularly critical for accurately addressing the “deviations from intended interventions” and “measurement of outcomes” domains [5]. A multitude of questions and high professional knowledge requirements make RoB2 challenging to apply and increase the time required for systematic reviews. Studies have shown that the time needed to assess one outcome using RoB2 can range from approximately 28 to 40 minutes [4,5]. This is an important reason why only 69.3% of SRs use RoB2 or even 28.8% of SRs to evaluate multiple outcomes [6].

Large language models (LLMs) have gradually been embraced by the medical field due to their excellent text comprehension, information extraction, and language processing capabilities, and are regarded as key technologies that could revolutionize medical practice and research [7,8]. LLMs demonstrate unique advantages when dealing with the RoB2 assessment framework, they can not only identify logical connections between different parts of RCT reports through attention mechanisms but also precisely capture details in complex trial methodologies, which is particularly important for areas requiring comprehensive judgment. The chain-of-thought reasoning ability of LLMs enables them to simulate the step-by-step decision-making process required by professional reviewers performing RoB2 evaluations, thereby reducing inconsistencies caused by subjective human judgment. By maintaining standardized assessment criteria across different studies, LLMs have the potential to address the IRR issues frequently observed in previous research. Existing studies have confirmed that with specially designed prompting techniques, LLMs can successfully perform a modified version of the Cochrane ROB tool assessment, significantly reducing the human resources and time costs associated with traditional assessment methods [9,10]. However, given the multilevel complexity of the RoB2 structure and the vast amount of information that needs to be integrated during the assessment process, there is still insufficient evidence supporting the effectiveness of LLMs in performing complete RoB2 assessments in systematic reviews.

This research aims to explore prompt engineering methods based on the Claude (Anthropic) model to address the efficiency and consistency challenges currently faced in RoB2 assessment practice. Our main purpose is to comprehensively evaluate whether LLMs can skillfully apply the RoB2 tool to professionally assess RCTs and to compare its assessment results with those of human experts using strict noninferiority standards. Through this study, we hope to provide empirical foundations for establishing a reliable LLM-assisted systematic review methodology, thereby improving the efficiency and quality of evidence synthesis in medical research.

## Methods

### Study Design

This feasibility study aims to investigate the capability of LLMs in assessing the RoB of RCTs with RoB2.

### Data Source

We searched the Cochrane library using the terms: (Revised Cochrane RoB) OR RoB2 OR RoB-2 from inception through October 8, 2023, to identify systematic reviews that assess the RoB in RCTs with RoB2. We categorized the Cochrane Reviews based on the domain of deviations from intended interventions (assignment or adhering) of interest in Cochrane Reviews. We extracted all RCTs included in Cochrane Reviews, and the extracted results were entered into Microsoft Excel, with each RCT assigned a unique identifier. Subsequently, we generated 23 random numbers for both categories using a computer and matched these random numbers with the identifiers of the RCTs to determine which RCTs would be assessed. For each RCT included, we extracted the primary outcome as reported in the corresponding Cochrane review.

### Establishment of the Criterion Standard

We selected 3 experienced reviewers to assess the RoB of the 46 RCTs included. None of the reviewers had previous exposure to the selected RCTs. All reviewers completed a week-long training on systematic reviews to ensure a consistent understanding of RoB2. Three reviewers (WZ, DX, and CB) initially conducted independent judgments of 46 RCTs using standardized criteria, recording the time taken for the judgments. All results were resolved through consensus. We randomly selected 3 evaluation results from each category to construct the prompt, which were used as benchmarks to assess the accuracy of the answers generated by the LLMs.

### Prompt Construction

**Figure 1:**
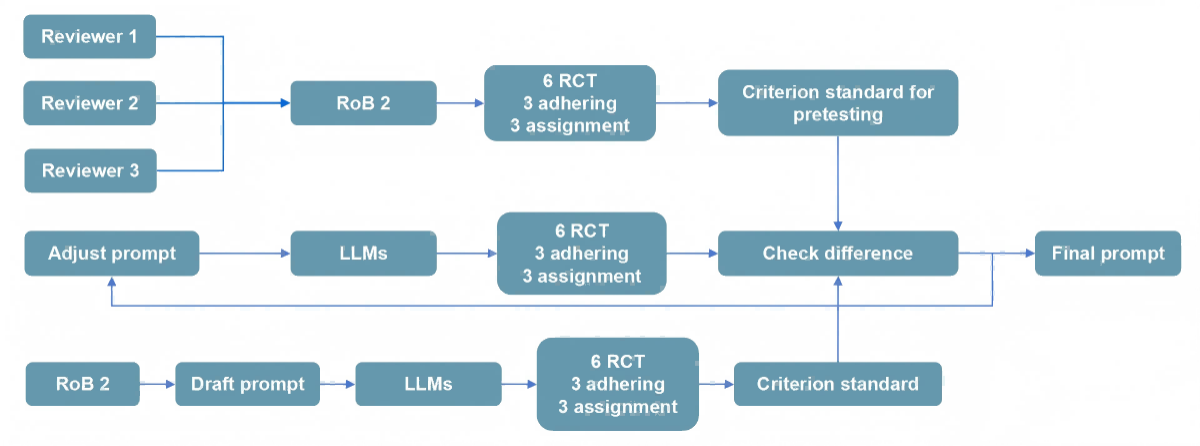
Flow diagram of the study. LLMs: large language models, RCTs: randomized controlled trials, RoB2: risk-of-bias tool.

We designed a structured draft prompt based on the documents publicly available on the RoB2 website. The prompt was intended to guide LLMs in completing the following tasks: identify and extract key information relevant to each signaling question within RoB2, respond to the signaling questions, make judgments on each domain, and provide the basis for the judgments. We used the draft prompt to assess 6 RCTs [11-16], compared the outcomes with the criterion standard, and iteratively refined the prompt based on the responses. This process was repeated until satisfactory performance was attained. Figure 1 shows the main study process. The final prompt can be found in Multimedia Appendix 1.

### LLMs Assessment

The final prompt was used to guide Claude 3.5 Sonnet in conducting assessments from October 10, 2023, to August 22, 2024, with no continued training or fine-tuning. The outputs were accurately transcribed into Multimedia Appendix 1. Any assessment interrupted by technical issues was excluded and promptly redone. Each RCT was assessed twice with Claude 3.5, using the same prompt and ensuring consistent model versions. Throughout the process, strict protocol adherence was maintained to ensure assessment quality.

### Statistical Analysis

We performed a descriptive analysis to evaluate the accuracy of the LLM-generated judgments in comparison with the judgments of reviewers and the judgments of Cochrane systematic reviews. This analysis focused on the accuracy of domain judgment and signaling question judgment. During prompt development and optimization, we observed that the inclusion of an “NA” option led to its overuse by the LLMs, potentially interfering with domain judgment. Consequently, the final prompt omitted instructions for generating “NA” responses. In our comparative analysis, signaling questions judged as “NA” by reviewers were excluded from accuracy calculations, as LLM-generated results for these questions would not influence the outcome. Due to the logical issues previously identified in the LLMs during previous research [9], we further investigated the discrepancies between the domain judgments generated by the LLMs and the judgments formed by the LLM-generated signaling questions according to the RoB2 algorithm. To measure the stability of LLMs, we calculated the consistent assessment rates and Cohen κ in domains and signaling questions [5]. To measure the IRR, we calculated the Fleiss’ κ for reviews for individual domains. We compared the time spent by the LLMs and the reviewer group to assess the efficiency gains obtained through using LLMs.

## Results

### Overview

**Figure 2:**
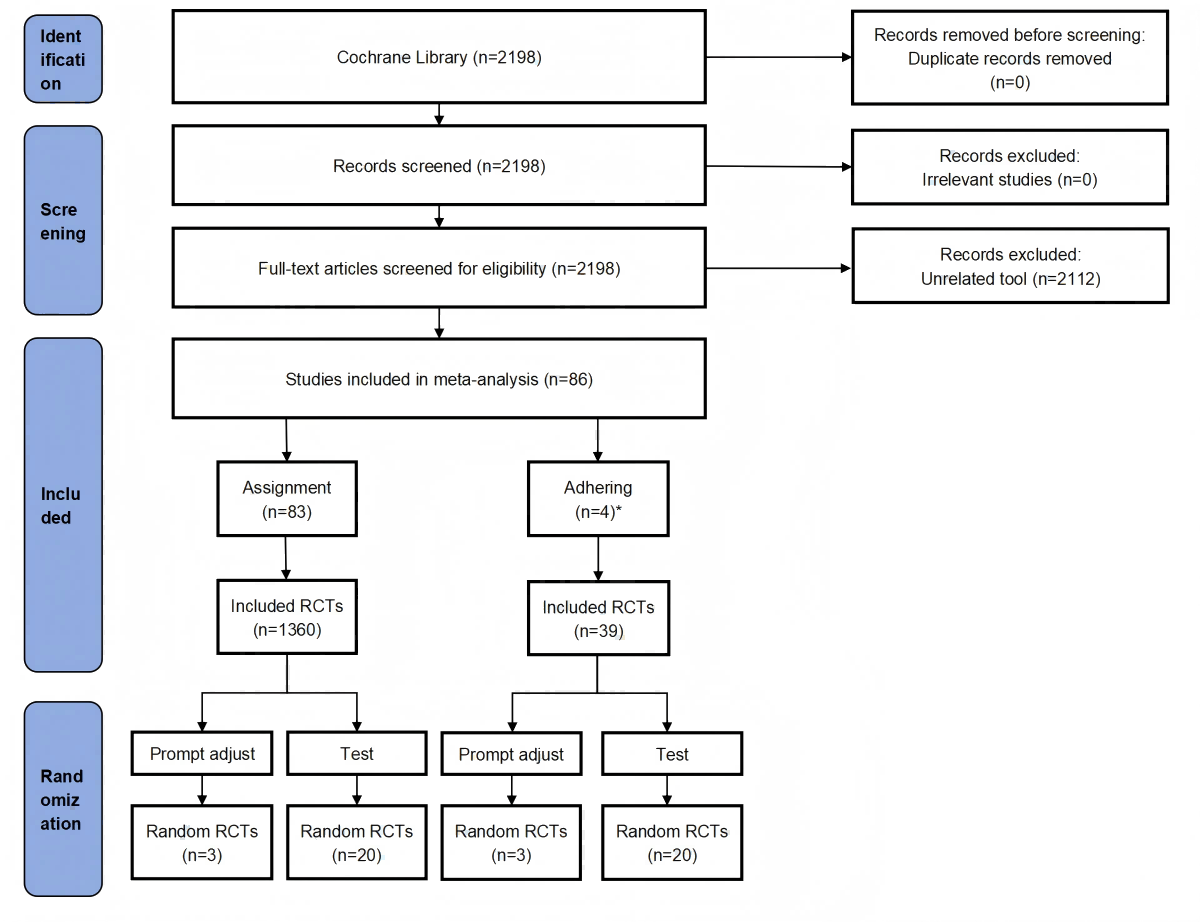
Literature screening and randomization flow diagram. RCT: randomized controlled trial.

Our initial search identified 2198 records. We subsequently reviewed the full texts of 2198 potentially eligible Cochrane Reviews. Finally, 86 Cochrane Reviews including 1399 RCTs were determined to meet the inclusion criteria (Figure 2). Details of included studies can be found in Table S1 in Multimedia Appendix 1.

### Characteristics of Included Cochrane Reviews and RCTs

Among 86 Cochrane Reviews, 83 were interested in the effect of assignment to the domain of bias due to deviations from intended interventions, and 4 were interested in adhering. Among these, 1 review reported on the assessment of both assignment and adhering. The 20 RCTs [17-36] interested in assignment were drawn from 19 Cochrane reviews, 2 of these studies focused on COVID-19, while the other studies focused on adolescents, patients who initiated in vitro fertilization, and others. The interventions included medications, exercise, supplements, meditation, and others. The 20 RCTs [37-56] interested in adhering were drawn from 4 Cochrane Reviews. The primary population of interest for the RCTs was chronic obstructive pulmonary disease (COPD), followed by asthma. Interventions included in the RCTs involved drugs, exercise, supplements, and others (Table S2 in Multimedia Appendix 1).

### RoB2 Assessment With Reviewers, the Cochrane Reviewers, and LLMs

Among the 20 articles focusing on adhering, 18 Cochrane Reviews and 19 reviewer judgments resulted in “High risk,” primarily due to low reporting rates of adhering-related information, which led to all articles examining adhering being classified as “High risk.” In contrast, the distribution of RoB of RCTs focusing on allocation was more heterogeneous. The majority of these RCTs were categorized as having “Some concerns,” with fewer RCTs categorized into “Low risk” or “High risk.” The judgments from both Cochrane Reviews and reviewers for the randomly selected RCTs and the consistency rate among them are presented in Table 1. The IRR of reviewers is 0.57, with detailed results available in the Multimedia Appendix 1.

**Table 1:** Risk-of-bias tool judgments.

RoB2^a^	Cochrane Reviews				Reviewers				IRR^b^		Consistency rate (%)
	Low risk	Some concern	High risk	Low risk		Some concern	High risk				
Domain 1	26	12	2	22		17	1	0.67		80	
Domain 2 (assignment)	10	8	2	7		11	2	0.27		85	
Domain 2 (adhering)	1	1	18	1		0	19	0.54		95	
Domain 3	24	3	13	28		1	11	0.87		85	
Domain 4	32	4	4	20		17	3	0.61		67.5	
Domain 5	18	21	1	22		18	0	0.58		77.5	
Overall (assignment)	4	12	4	2		15	3	0.57		85	
Overall (adhering)	0	0	20	0		0	20	0.89		100	

^a^RoB2: Risk-of-Bias tool.

^b^IRR: interrater reliability.

### Accuracy of LLMs Compared With Reviewers and the Cochrane Reviewers in Domain

The complete judgments are summarized in Multimedia Appendix 1. Compared with Cochrane Reviews, the LLMs’ judgments demonstrated accuracy rates of 57.5% and 70% for overall (assignment) and overall (adhering), respectively. When compared with reviewer judgments, the LLMs’ accuracy rates for overall (assignment) and overall (adhering) were 65% and 70%, respectively. The average accuracy rates for the remaining 6 domains were 65.2% (95% CI 58.9-71.4) and 74.2% (95% CI 65.3-83.1) when compared with Cochrane Reviews and reviewers, respectively. In summary, except for Domain 2 (both assignment and adhering), the LLMs exhibited relatively good accuracy rates in comparison to both Cochrane Reviews and reviewer judgments (Table 2).

**Table 2:** Accuracy of large language models compared with reviewers and the Cochrane reviewers in domain.

RoB2^a^	Cochrane Reviews (%)	Reviewers (%)
Domain 1	75	85
Domain 2 (assignment)	57.5	65
Domain 2 (adhering)	50	55
Domain 3	70	83.8
Domain 4	67.5	76.3
Domain 5	73.8	93.8
Overall (assignment)	57.5	65
Overall (adhering)	70	70

^a^RoB2: Risk-of-bias tool.

### Accuracy of LLMs Compared With Reviewers in Signaling Questions

**Figure 3:**
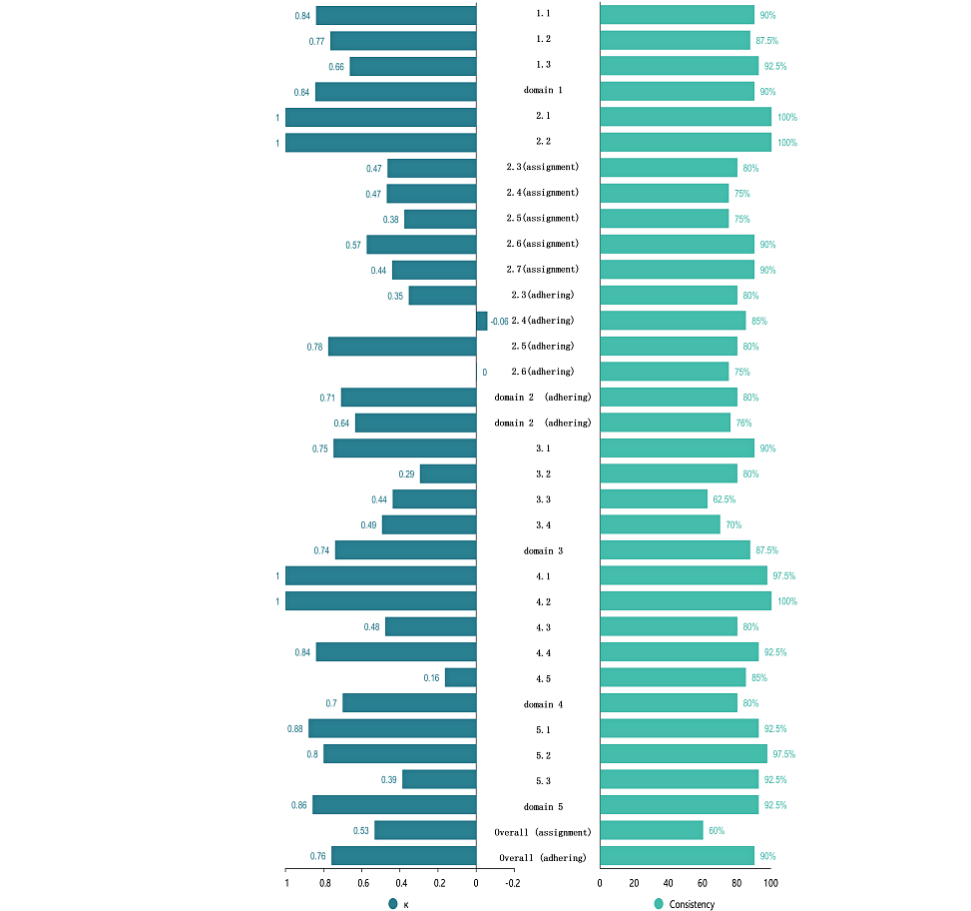
Cohen κ value and consistency between 2 large language model outputs.

Due to incomplete reporting of signaling question judgments in some Cochrane Reviews, our comparative analysis at the signaling question level was conducted exclusively against reviewer judgments. At the signaling question level, accuracy rates exceeded 70% for all questions except 2.4 (assignment), 2.5 (assignment), 3.3, and 3.4. The signaling questions 2.4 (assignment) and 2.5 (assignment) were excluded when calculating accuracy at the signaling question level, as they only had one available judgment after excluding the NA option. LLMs achieved an average accuracy of 83.2% (95% CI 77.5-88.9), excluding signaling questions 2.4 (assignment) and 2.5 (assignment; Multimedia Appendix 2). Given our previous findings of potential logical inconsistencies in LLM-generated judgments that deviated from prompt-specified methodologies, we conducted a further analysis. This investigation compared LLM-generated domain judgments with those derived from LLM-generated signaling question responses using the RoB2 decision algorithm. This secondary analysis revealed marked improvements in accuracy for Domain 2 (assignment) and overall (adhering) areas where LLM-generated domain judgments had initially demonstrated lower accuracy. Moderate accuracy improvements were also observed for Domain 2 (assignment), Domain 4, and overall (assignment). Other domains showed negligible differences (Figure 3 and Tables 3 -4).

**Table 3:** Accuracy of large language models compared with reviewers and the Cochrane reviewers in domain.

RoB2^a^	Cochrane Reviews (%)	Reviewers (%)
Domain 1	73.8	85
Domain 2 (assignment)	67.5	67.5
Domain 2 (adhering)	77.5	95
Domain 3	67.5	83.8
Domain 4	67.5	85
Domain 5	72.5	93.6
Overall (assignment)	62.5	65
Overall (adhering)	90	90

^a^RoB2: Risk-of-bias tool.

**Table 4:** Accuracy of large language model–generated and algorithm-derived judgment compared with Cochrane reviews and reviewers for each domain.

Domain	Cochrane Reviews		Reviewers	
	LLM^a^ generation (%)	Algorithm generation (%)	LLM generation (%)	Algorithm generation (%)
Domain 1	75	75	85	85
Domain 2 (assignment)	57.5	67.5	65	67.5
Domain 2 (adhering)	50	77.5	55	95
Domain 3	70	67.5	83.8	83.8
Domain 4	67.5	67.5	76.3	85
Overall (assignment)	65	65	57.5	62.5
Overall (adhering)	70	90	70	90

^a^LLM: large language model.

### Stability

Due to the homogeneity of RCTs in some signaling questions (eg, 2.4 adhering), a significant difference between the calculated Cohen κ and the actual consistent assessment rate (Figure 3). Consequently, this study primarily uses the stability of LLM-generated results as the benchmark for assessing the stability of LLM outputs. The LLMs demonstrated significant consistency between its 2 output iterations, with an average consistent assessment rate of 85.2% (95% CI 85.15-88.79). A 100% consistent assessment rate was achieved for signaling questions 2.1, 2.2, and 4.2, while an additional 12 signaling questions or domains exhibited a consistent assessment rate exceeding 90%. However, the consistent assessment rate for signaling question 3.3 and the “Overall (assignment)” domain fell below 70%.

### Efficiency

The mean assessment time of human reviewers was 31.5 (SD 12.9) minutes, while the LLMs completed assessments in 1.9 (SD 0.2) minutes. The mean difference in assessment time between human reviewers and the LLMs was 29.6 (95% CI 25.6-33.6) minutes, representing a 93.6% reduction from the average human assessment time.

## Discussion

### Principal Findings

In this study, we evaluated the accuracy of LLMs in assessing the RoB in RCTs with RoB2. To our knowledge, this is the first study using LLMs to assess RCTs through RoB2. Cochrane Reviews involve multiple research teams, each potentially applying different standards when judging specific signaling questions. This could lead to variations in the accuracy of LLM judgments. Therefore, we compared LLM-generated judgments with results from Cochrane Reviews and from 3 professional reviewers who assessed RCTs according to standardized criteria. This approach more accurately simulates the use of RoB2 and LLMs to assess the RoB of RCTs under consistent protocols.

We found differences between Cochrane systematic review judgments and reviewer judgments in Domains 1, 4, and 5. These differences are likely attributable to Cochrane Reviews assuming randomization and allocation concealment based on the authority of the RCT research team, such as: “States only that this was a randomized trial, without mentioning concealment. However, this was conducted by an experienced investigator and clinical trial network. These details were likely omitted for word‐count purposes.” In domain 4, the main disagreement concerned patient-reported subjective outcomes: when outcome assessors were blinded but patients were not, some Cochrane Reviews selected “N” for signaling question 4.4, reasoning that assessors were blinded, whereas our approach dictated selecting “Y.” Furthermore, to align with the LLMs’ analytical capabilities for existing information, reviewers only assessed content reported in the main text, excluding Multimedia Appendices and protocols. This approach may have resulted in reviewers missing details reported only in the Multimedia Appendices and protocols.

LLMs achieved an average accuracy of 83.2% in signaling questions, excluding signaling questions 2.4 (allocation) and 2.5 (allocation). The primary issue in signaling question 1.1 stems from the LLMs’ ability to extract randomization methods but made erroneous judgments. For instance, while correctly extracting the statement “The article does not describe specific methods for generating the random sequence, only stating ‘The randomization list was generated by an independent psychologist using the RAND function of Microsoft Excel 2010’,” LLMs incorrectly classified it as “NI.” Signaling question 2.3 predominantly exhibited insufficient information extraction, rendering LLMs unable to properly evaluate existing evidence. The errors in signaling question 3.1 mainly originated from the misidentification of both the number of participants randomized and those completing the intervention, leading to incorrect calculations of data completeness percentages. This fundamental error subsequently propagated to inaccuracies in questions 3.3 and 3.4. For signaling question 4.3, the LLM frequently neglected the concept of blinding outcome assessors. When articles explicitly mentioned outcome assessors, LLMs misclassified patient-reported outcomes as assessor-evaluated outcomes, and misinterpreted assessor-evaluated outcomes as patient-reported ones, resulting in faulty judgments. The errors of deviating from the predetermined judgment method and insufficient extraction of corresponding information were prevalent across other signaling questions as well. For instance, in signaling question 2.6 (adhering), judgments of “Y” were observed when “N” should have been selected according to the intention-to-treat analysis protocol. We explored potential solutions, such as having LLMs judge only one signaling question per response, which improved accuracy. However, due to Claude access frequency limitations and the tedious nature of decomposing individual signaling questions, we did not apply this approach as it would compromise the goal of rapid RoB2 assessment using LLMs. In addition, importing PDFs through Claude’s built-in plugin, rather than converting them to Word format beforehand, resulted in incomplete conversion of image content to text, leading to some information loss.

Furthermore, errors in judging signaling questions 2.4 (assignment) and 2.5 (assignment) were caused by the incorrect judgment of 2.3 (assignment) as “N”, leading LLMs to generate “NA” and blank judgments for 2.4 and 2.5. This cascading effect of errors in prerequisite questions affecting subsequent signaling questions was also observed in other questions with applicable preconditions.

In internal validation, LLMs’ accuracy in domains was lower than on signaling questions. This is because the domain is influenced by multiple signaling questions, but there is not a strong correlation between accuracy in the domain and signaling questions within the domain. Discrepancies between “NI” and “Y” or “NI” and “N” were inconsistent for signaling questions, but they all indicated the same RoB in a domain. Moreover, LLMs sometimes deviated from the RoB2 decision algorithm when generating domain judgments, instead making independent assessments of overall domain RoB, with a tendency to generate “Some Concerns” judgments. Consequently, we attempted to form domain judgments using Excel based on the RoB2 decision algorithm applied to signaling question judgments, rather than relying on LLM-generated domain judgments. Results showed significant improvements in Domains 2 (adhering), 2 (assignment), and overall (adhering), which were the domains with lower accuracy in LLM-generated judgments.

Although the accuracy of domain judgments generated by LLM is relatively low, the 83.2% accuracy of signaling questions is sufficient to give researchers confidence in LLM’s capabilities. Previous studies and reviews have shown that there are certain differences in the results of human reviewers (IRR=0.16) [4]. LLMs accuracy of 83.2% represents a level of consistency that is comparable to or may exceed human-assessed reliability in certain situations. However, LLMs still carry a certain risk of error, and the current level of LLM performance is not yet sufficient to completely replace independent assessments by researchers. Instead, LLMs are better suited to complement the work of researchers, potentially replacing the need for a second researcher in back-to-back assessments, thereby supplementing the detection of unidentified bias risks. In addition, when using LLM for RoB2 evaluation, researchers should clearly indicate in the literature the specific LLM, its version, and the prompt strategies adopted to ensure reproducibility and transparency.

### Limitations

This study has several limitations. First, due to the limited focus on adhering assessment in Cochrane Reviews and considering the number of eligible RCTs, we used a relatively small sample size. This may introduce certain biases, necessitating future large-scale studies to derive more trustworthy conclusions. In addition, due to this limitation, the research domain for adhering studies was restricted to COPD and asthma, which restricts the exploration of the bias of LLM in different medical domains. Second, the RoB of RCTs, which were classified as adhering, were high because of the judgments of Domain 2 (deviations from intended interventions), which precluded a thorough examination of the LLMs’ capability to assess low-risk studies of this domain. Third, current LLMs are unable to process extensive appendices in a single iteration. Consequently, our standardization process with human reviewers did not incorporate supplementary materials such as relevant registration documents and appendices. Our judgments were based solely on the full text of publications, with protocol availability as the only consideration for registration information, without further investigation into the completeness of the information. While this approach more precisely validates the LLMs’ judgment capabilities on available information, it slightly deviates from actual research practices. Fourth, potential biases within the LLM itself must be acknowledged, as these models may perpetuate or amplify biases present in their training data, potentially affecting their judgment of certain types of studies or methodologies.

### Future Studies

The application research LLMs for RoB2 assessments began in early 2023, but initial efforts were limited by lower intelligence levels of earlier models and suboptimal prompting strategies, resulting in modest accuracy. With rapid advancements in LLM capabilities, our current study demonstrates significantly improved performance, which suggests that the accuracy rates reported in this study are likely to further increase as LLM technology continues to evolve. Future research should monitor these advancements to track improvements in RoB2 assessment accuracy. Interestingly, during our validation process, we observed that modern LLMs can independently access and apply RoB2 information to evaluate RCTs even without extensive prompting. Our prompt engineering, therefore focused on correcting specific error patterns rather than providing comprehensive guidance. This raises an intriguing research question: how accurately could advanced LLMs perform RoB2 assessments with minimal or no specific prompting? Future studies could explore this “zero-shot” or “few-shot” performance to better understand the evolving capabilities of these models. In addition, due to the limited number of Cochrane Reviews focusing on adhering, particularly in COPD and asthma interventions, the generalizability of our findings for adhering assessment remains constrained. Future research should expand to other medical domains to validate these results across a broader range of conditions and interventions. Furthermore, studies examining how LLMs adapt to evolving medical knowledge and comparing their performance with other automated tools could provide valuable insights into the robustness and practical utility of LLM-assisted RoB2 assessments in dynamic research environments. Longitudinal studies evaluating model performance over time could also help measure consistency and adaptation to evolving medical guidelines and practices.

### Conclusions

In this study on applying LLMs to assess RoB2 in RCTs, we found that LLMs achieved commendable accuracy and consistency with a structured prompt, which particularly attributed to their ability to process complex methodological details and simulate assessors’ decision-making processes via chain-of-thought reasoning. While the accuracy of the LLMs had not yet reached the level that can completely replace human assessment, their high accuracy demonstrated strong potential in assisting researchers with RoB2 assessments.

## Appendix

Multimedia Appendix 1 Additional data.

Multimedia Appendix 2 Accuracy of large language models compared with reviewers in signaling question.

## Abbreviations

COPD: chronic obstructive pulmonary disease
IRR: interrater reliability
LLM: large language model
RCT: randomized controlled trial
RoB2: Risk-of-Bias tool
SR: systematic review

## Data Availability

Data are supplied in supporting files available for download along with the published manuscript.
