# Correction: Large Language Model–Assisted Risk-of-Bias Assessment in Randomized Controlled Trials Using the Revised Risk-of-Bias Tool: Evaluation Study

**DOI:** 10.2196/80519

**Published:** 2025-07-14

**Authors:** JMIR Editorial Office

**Affiliations:** 1 JMIR Publications Toronto, ON Canada

In “Large Language Model–Assisted Risk-of-Bias Assessment in Randomized Controlled Trials Using the Revised Risk-of-Bias Tool: Evaluation Study” (J Med Internet Res 2025;27:e70450) [1] published June 24, 2025, the study design was changed in error during editing. The title is corrected to be, “Large Language Model–Assisted Risk-of-Bias Assessment in Randomized Controlled Trials Using the Revised Risk-of-Bias Tool: Evaluation Study”.

The correction will appear in the online version of the paper on the JMIR Publications website, together with the publication of this correction notice. Because this was made after submission to PubMed, PubMed Central, and other full-text repositories, the corrected article has also been resubmitted to those repositories.
