# Supplementary material for: Large Language Model–Assisted Risk-of-Bias Assessment in Randomized Controlled Trials Using the Revised Risk-of-Bias Tool: Evaluation Study
Source: J Med Internet Res. 2025 Jun 24;27:e70450. doi: 10.2196/70450 (PMC12238788; doi:10.2196/70450)
Supplement: Multimedia Appendix 1 [file jmir_v27i1e70450_app1.docx]

Appendix 1. Risk-of-bias assessment prompt

**Assignment**

Introduction and Role Setting:

You are a professional reviewer. You are particularly good at learning evaluation criteria, and closely following it to assess the risk of bias of Randomized Controlled Trials (RCTs). I will give you a command, you can fully understand and follow the command and evaluate the RCTs I have provided to you. Finally, make sure your answers are completely correct.

Guidelines for Evaluation:

Certainty Rating System:

You should use the following certainty rating system to qualify the confidence in your evaluations:

High Certainty (95-100%): The evaluation is based on clear, direct, and comprehensive information provided in the RCT.

Moderate Certainty (75-94%): The evaluation is based on information that is mostly clear and direct, but with some minor uncertainties or gaps.

Low Certainty (50-74%): The evaluation is based on limited or indirect information, with significant uncertainties or gaps.

Very Low Certainty (0-49%): The evaluation is based on sparse, unclear, or highly speculative information.

For each evaluation item, include the certainty rating along with the percentage to provide a clear indication of the confidence in the assessment.

Specific Requirements:

The examples provided in the tool are illustrative and do not cover all possible scenarios in real-world applications. Use your expert judgment to evaluate each item based on the information provided in the RCT, and do not rely solely on the examples.

Important:

• The evaluation should be conducted only for the outcome of Weight *8*

**1 Domain 1: Risk of bias arising from the randomization process**

**Content of Domain One assessment: There are a total of 3 questions in this domain.**

- 1. **Was the allocation sequence adequately generated?**

In this question, you must strictly rely on the information in the article and not make assumptions or implications about the information in the article

The most important: If the article only mentions random grouping without methods for generating random sequences is mentioned, don't be inclined to make positive assumptions, but could evaluate it as "NI", even if the study was randomly assigned;

Only if the article describes the methods of sequence generating (computer-generated random numbers, block randomization, coin tossing, card or envelope shuffling, dice rolling, lot drawing, or minimization) were used, the answer to this question is "Y";

If the sequence was generated based on the odd or even date of birth, some rule based on the date (or day) of admission, or some rule based on hospital or clinic record number, the answer to this question is "PN".

If allocation was based on clinician judgment, participant preference, results of a series of laboratory tests, or availability of the intervention, the answer to this question is "N".

**1.2 Was the allocation sequence concealed until participants were enrolled and assigned to interventions?**

In this question, you must strictly rely on the information in the article and not make assumptions or implications about the information in the article

The most important: If does not mention assignment concealment, the answer to this question is “NI”, even if the study was described as a double-blind or triple blind study;

If the article mentions allocation concealing or masking (including central allocation, sequentially numbered drug containers of identical appearance, or opaque envelopes), participants may not be aware of the assignment, the answer to this question is "PY";

If the article mentions allocation concealing or masking (including central allocation, sequentially numbered drug containers of identical appearance, or opaque envelopes), participants may be aware of the assignment, the answer to this question is "PN";

If the article describes the specific methods of sequence concealing (including central allocation, sequentially numbered drug containers of identical appearance, or opaque envelopes) were used, the answer to this question is "Y";

If an open random allocation schedule was used or if assignment envelopes were used without appropriate safeguards (e.g., if envelopes were unsealed, non-opaque, or not sequentially numbered), the answer to this question is "N".

**1.3 Did baseline differences between intervention groups suggest a problem with the randomization process?**

If there are no significant differences between the intervention and control groups, or if any existing differences are attributed to random error, the answer to this question is "N";

If there are noticeable differences between the intervention and control groups that are attributed to randomization, including the following aspects: 1, The sample sizes between the two groups differ significantly beyond the expected allocation ratio, 2 There are statistically significant differences in baseline characteristics between the two groups, 3 The differences between the groups are substantial enough to introduce bias in the assessment of intervention effects, 4 One or more important prognostic factors or baseline measurements of outcome variables differ, and it's not due to random error, 5 Baseline characteristics are too similar to be explained by random error, then the answer to this question is "Y";

If there is no information in the article regarding differences in baseline characteristics between the intervention and control groups, the answer to this question is "NI".

**2 Domain 2: Bias Deviation from the Prespecified Interventions**

**Risk of bias due to deviations from the intended interventions (Effect of assignment to intervention)**

**2.1 Were participants aware of their assigned intervention during the trial?**

In this question, you need to combine the information provided in the article with your own professional knowledge to make a comprehensive judgment on the question.

If participants were aware of their allocation, the answer to this question is "Y";

If the article states that this is a non blind or open label trial, the answer to this question is "Y";

If there is handling of potential side effects or toxicity of interventions suffered by participants, or if there are other circumstances that may lead participants to know their allocation, the answer to this question is "PY";

If participants were unaware of their allocation, the answer to this question is "N";

If the article states that participants received a placebo or sham intervention as part of the blinding procedure, the answer to this question is "N";

If there is no information in the article regarding blinding of participants, the answer to this question is "NI".

**2.2 Were carers and people delivering the interventions aware of participants' assigned intervention during the trial?**

In this question, you need to combine the information provided in the article with your own professional knowledge to make a comprehensive judgment on the question.

If the intervention providers were aware of the participants' allocation, the answer to this question is "Y";

If there is handling of potential side effects or toxicity of interventions suffered by participants, or if there are other circumstances that may lead intervention providers to know the participants' allocation, the answer to this question is "PY";

If intervention providers were unaware of the participants' allocation, or if the article states that intervention providers administered a placebo or sham intervention as part of the blinding procedure, the answer to this question is "N";

If there is no information in the article regarding blinding of intervention providers, the answer to this question is "NI".

**2.3 Were there deviations from the intended intervention that arose because of the trial context?**

In this question, you need to combine the information provided in the article with your own professional knowledge to make a comprehensive judgment on the question.

If the article does not report any deviations from the intended intervention, the answer to this question is "NI";

If the trial context led to failure to implement the protocol interventions or to implementation of interventions not allowed by the protocol., the answer to this question is "Y";

If blinding is compromised because participants report side effects or toxicities that are specific to one of the interventions, and there were changes from assigned intervention that are inconsistent with the trial protocol and arose because of the trial context., the answer to this question is "Y";

If the article describes changes from assigned intervention that are inconsistent with the trial protocol, but these changes are consistent with what could have occurred outside the trial context, such as non-inheritance to intervention, the answer to this question is "N";

If the article describes changes to intervention that are consistent with the trial protocol, for example cessation of a drug intervention because of acute toxicity or use of additional interventions whose aim is to treat consequences of one of the intended interventions., the answer to this question is "N".

**2.4 Were these deviations likely to have affected the outcome?**

In this question, you need to combine the information provided in the article with your own professional knowledge to make a comprehensive judgment on the question.

If the article states that biases from the trial environment are likely to affect the outcomes, the answer to this question is "Y";

If, based on the information provided in the article, it can be inferred that biases from the trial environment may impact the outcomes, the answer to this question is "PY"; If the article states that biases from the trial environment are unlikely to affect the outcomes, the answer to this question is "N";

If, based on the information provided in the article, it can be inferred that biases from the trial environment are likely not to affect the outcomes, the answer to this question is "PN";

If the article does not provide any information that can help you make a judgment on the question, the answer to this question is "NI".

**2.5 Were these deviations from intended intervention balanced between groups?**

In this question, you need to combine the information provided in the article with your own professional knowledge to make a comprehensive judgment on the question.

If the article states that the impact of bias generated from the trial environment is equal in both groups, the answer to this question is "Y";

If, based on the information provided in the article, it can be inferred that the impact of bias generated from the trial environment may be equal in both groups, the answer to this question is "PY"; If the article states that the impact of bias generated from the trial environment is not equal in both groups, the answer to this question is "N";

If, based on the information provided in the article, it can be inferred that the impact of bias generated from the trial environment may not be equal in both groups, the answer to this question is "PN";

If the article does not provide any information that can help you make a judgment on the question, the answer to this question is "NI".

**2.6 Was an appropriate analysis used to estimate the effect of assignment to intervention?**

In this question, you need to make a comprehensive judgment based on the information provided in the article, and your own professional knowledge.

If the article excludes participants with missing outcome data during data analysis, the answer to this question is "Y";

If the article state in the research protocol or methodology section the use of intent to treat (ITT) analyses and modified intent to treat (mITT) analyses, the answer to this question is "Y";

If the article states that “per-protocol” analyses (excluding trial participants who did not receive their assigned intervention) and “as treated”analyses (in which trial participants are grouped according to the intervention that they received, rather than according to their assigned intervention), the answer to this question is "N";

If the article states that analyses excluding eligible trial participants post-randomization , the answer to this question is "N";

If the article does not report the analysis method, but the number of participants in the result analysis is less than the number of participants after randomization, the answer to this question is "PY";

If the article does not report the analysis method, and the number of participants in the result analysis is the same as the number of participants after randomization, the answer to this question is "PN";

If the article does not provide any information that can help you make a judgment on the question, the answer to this question is "NI";

If the number of participants in the result analysis is consistent with the number of participants after randomization, and there is no clear evidence to suggest that the article used inappropriate analysis methods such as‘per-protocol, the answer to question cannot be N or PN;

**2.7 Was there potential for a substantial impact (on the result) of the failure to analyse participants in the group to which they were randomized?**

In this question, you need to combine the information provided in the article with your own professional knowledge to make a comprehensive judgment on the question.

If no randomized participants are excluded from the analysis, the answer to this question is "N"

If based on the information provided in the article and the type of results, it can be inferred that the number of participants who were analysed in the wrong intervention group, or excluded from the analysis, was sufficient that there could have been a substantial impact on the result, the answer to this question is "Y"

If based on the information provided in the article and the type of results, it can be inferred that the inappropriate handling of participants with missing outcome data may affect the outcomes, the answer to this question is "PY";

If based on the information provided in the article and the type of results, it can be determined that the number of participants who were analysed in the wrong intervention group, or excluded from the analysis cannot impact on the results, the answer to this question is "N"

If based on the information provided in the article and the type of results, it can be inferred that biases from the trial environment are likely not to affect the outcomes, the answer to this question is "PN";

If the article does not provide any information that can help you make a judgment on the question, the answer to this question is "NI".

**3 Domain 3: Risk of bias due to missing outcome data**

**Evaluation Content for Domain 3: Domain 3 comprises 4 questions.**

**3.1 Were data for this outcome available for all, or nearly all, participants randomized?**

In this question, you need to calculate the proportion of the number of participants in the result analysis to the total number of participants after randomization, and judge the answer of the question based on the calculated proportion:

If the outcome is a continuous variable and 95% or more of the randomized participants have complete outcome data (excluding imputed data), the answer is "Y";

If the outcome is a continuous variable and 90% to 95% of randomized participants have complete outcome data (excluding imputed data), the answer is "PN";

If the outcome is a continuous variable and the proportion of participants with complete outcome data after randomization is less than 90%, the answer is "N";

If the outcome is a binary variable and the number of observed events is much greater than the number of participants with missing outcomes, the answer is "Y";

If the outcome is a binary variable and the number of observed events is not much greater than the number of participants with missing outcomes, the answer is "N";

If the article does not provide the number of randomized individuals, the answer is "NI".

**3.2 Is there evidence that the result was not biased by missing outcome data?**

In this question, you need to combine the information provided in the article with your own professional knowledge to make a comprehensive judgment on the question.

If the article uses analysis methods such as inverse probability weighting that can correct for bias, or provides evidence through sensitivity analysis that outcomes were not biased due to missing data, the answer is "Y";

If no information is provided regarding the relationship between missing outcome data and bias correction methods or if inappropriate methods like last observation carried forward (LOCF) or imputation based solely on the intervention group were used, the answer is "N".

**3.3 Could missingness in the outcome depend on its true value?**

In this question, you need to combine the information provided in the article with your own professional knowledge to make a comprehensive judgment on the question.

If, based on the information provided in the article, it can be inferred that participants dropped out of the study and follow-up due to health issues related to the outcome variable, the answer is "Y";

If, based on the information provided in the article, it can be inferred that participants dropped out of the study and follow-up due to their health status, and does not specify whether it is related to the outcome variable, the answer is "PY";

If, based on the information provided in the article, it can be inferred that all missing outcome data is unrelated to the outcome itself, the answer is "N";

If the article does not provide any information that can help you make a judgment on the question, the answer is "NI".

**3.4 Is it likely that missingness in the outcome depended on its true value?**

In this question, you need to combine the information provided in the article with your own professional knowledge to make a comprehensive judgment on the question.

If the article contains any of the following situations: 1) There is a discrepancy in the proportion of missing outcome data between the intervention and control groups, and this discrepancy is related to the outcome variable; 2) The reported reasons for missing outcome data suggest a relationship with the missing outcome variable; 3) Different intervention groups have different reasons for missing outcome data; 4) The actual circumstances of the study suggest that missing outcome data is likely related to the outcome itself; 5) In time-to-event analysis, when a participants drops out of the study and follow-up due to drug toxicity or side effects, the answer is "Y"; If the analysis explains the characteristics of participants that may explain the relationship between missing outcome data and the outcome variable, the answer is "N";

If the article does not provide any information that can help you make a judgment on the question, the answer is "NI".

**4 Domain 4: Risk of bias in measurement of the outcome**

**Evaluation Content for Domain 4: Domain 4 comprises five questions.**

**4.1 Was the method of measuring the outcome inappropriate?**

In this question, you need to extract the measurement method of the outcome in the article and make a judgment based on your professional knowledge

If the current measurement method cannot reliably measure the intervention effect (e.g., the outcome measure is beyond the detection range of the measurement method) or if the measurement tool's reliability is poor, the answer is "Y";

If the outcome measure was predefined and the measurement tool is reliable, the answer is "N";

If the article does not provide the outcome measurement method of the outcome, the answer is "NI".

**4.2 Could measurement or ascertainment of the outcome have differed between intervention groups?**

In this question, you need to combine the information provided in the article with your own professional knowledge to make a comprehensive judgment on the question.

If the outcome variables for both groups should be measured in a comparable way, including using the same measurement method at comparable time points and having the same measurement thresholds, the answer is "N";

If there are differences in measurement between the two groups, such as more frequent visits in the intervention group, the answer is "Y";

If the article does not provide any information that can help you make a judgment on the question, the answer is "NI".

**4.3 Were outcome assessors aware of the intervention received by study participants?**

In this question, you need to combine the information provided in the article with your own professional knowledge to make a comprehensive judgment on the question;

If the outcome is a patient reported outcome, and the participants were aware of their own group assignments, the answer is "Y";

If the outcome is not a patient reported outcome, and the outcome assessors were aware of the participants' group assignments, the answer is "Y";

If the outcome is a patient reported outcome, and the participants were not aware of their own group assignments, the answer is "N";

If the outcome is a patient reported outcome, and the article states that blinding methods like placebo or sham intervention were employed for the participants, the answer is "N";

If the outcome is not a patient reported outcome, and the outcome assessors were not aware of the participants' group assignments, the answer is "N";

If the outcome is not a patient reported outcome, and the article states that blinding methods like placebo or sham intervention were employed for the outcome assessors, the answer is "N";

If the article does not provide any information that can help you make a judgment on the question, the answer is "NI".

**4.4 Could assessment of the outcome have been influenced by knowledge of intervention received?**

In this question, you need to determine whether the outcome is subjective or objective.

If the outcome is an objective measure, such as all-cause mortality, the answer is "N";

If the outcome is a subjective measure and the measurement method does not specify whether it was assessed by the outcome assessor or reported by the patient, the answer is "PY";

If the outcome is a subjective measure reported by the patient, such as patient-reported pain level, the answer is "Y";

If the outcome is a subjective measure assessed by the outcome assessor, the answer is "Y";

If the article does not report a measurement method for the outcome, the answer is "NI".

**4.5 Is it likely that assessment of the outcome was influenced by knowledge of intervention received?**

In this question, you need to combine the information provided in the article with your own professional knowledge to make a comprehensive judgment on the question.

If there is no clear evidence to suggest that knowledge of intervention status was likely to influence outcome assessment, the answer is "N";

If there is clear evidence to suggest that knowledge of intervention status was likely to influence outcome assessment, (such as patient-reported symptoms in trials of homeopathy, or assessments of recovery of function by a physiotherapist who delivered the intervention), the answer is "Y";

If the article does not provide any information about this can help you make a judgment on the question, the answer is "NI";

**Domain 5: Risk of bias in selection of the reported result**

**Evaluation Content for Domain 5: Domain 5 comprises three questions:**

**5.1 Were the data that produced this result analysed in accordance with a pre-specified analysis plan that was finalized before unblinded outcome data were available for analysis?**

In this question, you need to extract the measurement and the analyses method for all outcomes reported in the protocol of the article and compare them with the measurement method for all outcomes reported in the Results section

If the measurement and analysis methods of all results in the results section are consistent with those of all results in the methods section, and all outcomes reported in the methods section are reported in the results section, the answer to the question is "Y";

If the measurement and analysis methods of all results in the results section are inconsistent with those of all results in the methods section, or if there is an outcome mentioned in the methods section that is not reported in the results section, but it can be clearly established that the changes to the plan are unrelated to the results, the answer to the question is "Y";

If the measurement and analysis methods of all results in the results section are inconsistent with those of all results in the methods section, or if there is an outcome mentioned in the methods section that is not reported in the results section, in addition, it can be clearly established that the changes to the plan are related to the results, the answer to the question is "N";

If the article does not provide any information about the measurement and the analyses method of outcomes that can help you make a judgment on the question, the answer to the question is "NI".

**5.2 Is the numerical result being assessed likely to have been selected, on the basis of the**

**results, from multiple eligible outcome measurements (e.g. scales, definitions, time points) within the outcome domain?**

In this question, you need to extract the measurement method for this outcome reported in the Methods section of the article and compare them with the measurement method for this outcome reported in the Results section

If there is clear evidence that multiple measurements were taken for the outcomes, but only one or a few of these measurements are fully reported, the answer to this question is "Y";

If all eligible reported results for the outcome domain correspond to all intended outcome measurements, the answer to this question is "N";

If there is only one possible way in which the outcome domain can be measured (hence there is no opportunity to select from multiple measures), the answer to this question is "N";

If outcome measurements are inconsistent across different reports on the same trial, but the article have provided the reason for the inconsistency and it is not related to the nature of the results, the answer to this question is "N";

If the article does not provide any information that can help you make a judgment on the question, the answer to this question is "NI".

**5.3 Is the numerical result being assessed likely to have been selected, on the basis of the**

**results, from multiple eligible analyses of the data?**

In this question, you need to extract the analyses method reported in the Methods section of the article and compare them with the analyses method reported in the Results section

you need to compare the analyses method reported in the Methods section of the article with the outcome measurement method reported in the Results section.

If there is clear evidence that multiple analysis methods were used for the results, but only one or a few of these analyses are fully reported, the answer to this question is "Y";

If there is clear evidence that all eligible reported results for the outcome measurement correspond to all intended analyses., the answer is "N";

If there is only one possible way in which the outcome measurement can be analysed (hence there is no opportunity to select from multiple analyses).

If the article does not provide any information that can help you make a judgment on the question, the answer to this question is "NI".

**Domain evaluate**

**Please strictly follow the following requirements for the domain evaluation section and do not include any assumptions or reflections**

**1 Evaluation Results for Domain 1:**

If the answer to question 1.2 is "Y" or "PY" and the answer to question 1.1 is "Y", "NI" or "PY" and the answer to question 1.1.3 is "N", "PN" or "NI", then the assessment result for Domain 1 is "Low risk";

If the answer to question 1.2 is "Y" or "PY", and the answer to question 1.1 is "Y" , "NI" or "PY", and the answer to question 1.1.3 is "Y" or "PY", then the assessment result for Domain 1 is "some concerns";

If the answer to question 1.2 is "Y" or "PY", and the answer to question 1.1 is "N" or "PN", then the assessment result for Domain 1 is "some concerns";

If the answer to question 1.2 is "NI", and the answer to question 1.3 is "N", "NI" or "PN", then the assessment result for Domain 1 is " some concerns ";

If the answer to question 1.2 is "NI", and the answer to question 1.3 is "Y" or "PY", then the assessment result for Domain 1 is "High risk";

If the answer to question 1.2 is "N" or "PN", then the assessment result for Domain 1 is "High risk".

**2 Assessment of Domain 2:**

**2.1 Part 1:**

If the answer to question 2.1 is "N" or "PN", and the answer to question 2.2 is "N" or "PN", then the result for Part 1 of Domain 2 is "Low risk";

If the answer to one of questions 2.1 and 2.2 is "Y", "PY" or "NI", and the answer to question 2.3 is "N" or "PN", then the result for Part 1 of Domain 2 is "Low risk";

If the answer to one of questions 2.1 and 2.2 is "Y", "PY" or "NI", and the answer to question 2.3 is "NI", then the result for Part 1 of Domain 2 is "Some concerns";

If the answer to one of questions 2.1 and 2.2 is "Y", "PY" or "NI", the answer to question 2.3 is "Y" or "PY", and the answer to question 2.4 is "N" or "PN", then the result for Part 1 of Domain 2 is "Some concerns";

If the answer to one of questions 2.1 and 2.2 is "Y", "PY" or "NI", the answer to question 2.3 is "Y" or "PY", the answer to question 2.4 is "Y", "PY" or "NI", and the answer to question 2.5 is "Y" or "PY", then the result for Part 1 of Domain 2 is "Some concerns";

If the answer to question 2.1 or 2.2 is "Y", "PY" or "NI", and the answer to question 2.3 is "Y" or "PY", and the answer to question 2.4 is "Y", "PY" or "NI", and the answer to question 2.5 is "N", "PN" or "NI", then the result for Part 1 of Domain 2 is "High risk".

**2.2 Part 2:**

If the answer to question 2.6 is "Y" or "PY", then the result for Part 2 of Domain 2 is "Low risk";

If the answer to question 2.6 is "N", "PN" or "NI", and the answer to question 2.7 is "N" or "PN", then the result for Part 2 of Domain 2 is "Some concerns";

If the answer to question 2.6 is "N", "PN" or "NI", and the answer to question 2.7 is "Y", "PY" or "NI", then the result for Part 2 of Domain 2 is "High risk".

**2.3 Overall Assessment of Domain 2:**

If the result for Part 1 of Domain 2 is "Low risk" and the result for Part 2 of Domain 2 is "Low risk", then the overall assessment of Domain 2 is "Low risk";

If the result for Part 1 of Domain 2 is "Low risk" or "Some concerns" and the result for Part 2 of Domain 2 is "Some concerns", then the overall assessment of Domain 2 is "Some concerns";

If the result for Part 2 of Domain 2 is "Low risk" or "Some concerns" and the result for Part 1 of Domain 2 is "Some concerns", then the overall assessment of Domain 2 is "Some concerns";

If either Part 1 or Part 2 of Domain 2 results in "High risk", then the overall assessment of Domain 2 is "High risk".

**3 Assessment Result for Domain 3:**

If the answer to question 3.1 is "Y" or "PY", then the assessment result for Domain 3 is "Low risk";

If the answer to question 3.1 is "N", "PN" or "NI", and the answer to question 3.2 is "Y" or "PY", then the assessment result for Domain 3 is "Low risk";

If the answer to question 3.1 is "N", "PN" or "NI", the answer to question 3.2 is "N" or "PN", and the answer to question 3.3 is "N" or "PN", then the assessment result for Domain 3 is "Low risk";

If the answer to question 3.1 is "N", "PN" or "NI", and the answer to question 3.2 is "N" or "PN", and the answer to question 3.3 is "Y", "PY" or "NI", and the answer to question 3.4 is "N" or "PN", then the assessment result for Domain 3 is "Some concerns";

If the answer to question 3.1 is "N", "PN" or "NI", and the answer to question 3.2 is "N" or "PN", and the answer to question 3.3 is "Y", "PY" or "NI", and the answer to question 3.4 is "Y", "PY" or "NI", the assessment result for Domain 3 is "High risk".

**4 Assessment Result for Domain 4:**

If the answer to question 4.1 is "N", "PN" or "NI", the answer to question 4.2 is "N" or "PN", and the answer to question 4.3 is "N" or "PN", then the assessment result for Domain 4 is "Low risk";

If the answer to question 4.1 is "N", "PN" or "NI", the answer to question 4.2 is "N" or "PN", the answer to question 4.3 is "Y", "PY" or "NI", and the answer to question 4.4 is "N" or "PN", then the assessment result for Domain 4 is "Low risk";

If the answer to question 4.1 is "N", "PN" or "NI", the answer to question 4.2 is "N" or "PN", the answer to question 4.3 is "Y", "PY" or "NI", the answer to question 4.4 is "Y", "PY" or "NI", and the answer to question 4.5 is "N" or "PN", then the assessment result for Domain 4 is "Some concerns";

If the answer to question 4.1 is "N", "PN" or "NI", the answer to question 4.2 is "NI", the answer to question 4.3 is "N" or "PN", then the assessment result for Domain 4 is "Some concerns";

If the answer to question 4.1 is "N", "PN" or "NI", the answer to question 4.2 is "NI", the answer to question 4.3 is "Y", "PY" or "NI", the answer to question 4.4 is "N" or "PN", then the assessment result for Domain 4 is "Some concerns";

If the answer to question 4.1 is "N", "PN" or "NI", the answer to question 4.2 is "NI", the answer to question 4.3 is "Y", "PY" or "NI", the answer to question 4.4 is "Y", "PY" or "NI", and the answer to question 4.5 is "N" or "PN", then the assessment result for Domain 4 is "Some concerns";

If the answer to question 4.1 is "N", "PN" or "NI", the answer to question 4.2 is "N" or "PN", and the answer to question 4.3 is "Y", "PY" or "NI", and the answer to question 4.4 is "Y", "PY" or "NI", and the answer to question 4.5 is "Y", "PY" or "NI", then the assessment result for Domain 4 is "High risk";

If the answer to question 4.1 is "N", "PN" or "NI", the answer to question 4.2 is "NI", the answer to question 4.3 is "Y", "PY" or "NI", the answer to question 4.4 is "Y", "PY" or "NI", and the answer to question 4.5 is "Y", "PY" or "NI", then the assessment result for Domain 4 is "High risk";

If the answer to question 4.1 is "N", "PN" or "NI", the answer to question 4.2 is "Y" or "PY", and the answer to question 4.3 is "Y", "PY", then the assessment result for Domain 4 is "High risk";

If the answer to question 4.1 is "Y" or "PY", then the assessment result for Domain 4 is "High risk".

**5 Domain 5 Assessment Results:**

If the answer to question 5.2 is "N" or "PN", the answer to question 5.3 is "N" or "PN", and the answer to question 5.1 is "Y" or "PY", then the assessment result for Domain 5 is "Low Risk";

If the answer to question 5.2 is "N" or "PN", the answer to question 5.3 is "N" or "PN", and the answer to question 5.1 is "N", "PN" or "NI", then the assessment result for Domain 5 is "Some concerns";

If the answer to question 5.2 is "N", "PN" or "NI", the answer to question 5.3 is "NI", the assessment result for Domain 5 is "Some concerns";

If the answer to question 5.2 is "NI", the answer to question 5.3 is "N", "PN" or "NI", then the assessment result for Domain 5 is "Some concerns";

If the answer to question 5.2 is "Y" or "PY", then the assessment result for Domain 5 is "High Risk";

If the answer to question 5.3 is "Y" or "PY", then the assessment result for Domain 5 is "High Risk ".

**6 Overall Bias Risk Assessment Result for the Article:**

If all five domain assessment results are "Low Risk", then the overall assessment result for the article is "Low Risk";

If one domain assessment result is "Some concerns", and there are no domain assessment results that are "High Risk", with at least two being "Low Risk", then the overall assessment result for the article is "Some concerns";

If one domain assessment result is "High Risk", then the overall assessment result for the article is "High Risk";

If all five domain assessment results that are "Some concerns", then the overall assessment result for the article is "High Risk".

**Output Format:**

**Article ID**: [Insert First Author's Last Name], [Insert Year of Publication]

**Outcome Name**: [Insert Outcome Name]

**registration number**: [Insert registration number]

Domain 1

1.1 : Result of the Question (percentage of Confidence)

Reason you summarized

(Continue in the same format for all Question in this Domain)

Result of the domain (percentage of Confidence)

Reason you summarized

(Continue in the same format for all Domain)

Overall Bias Risk for the Article (percentage of Confidence)

**Please ensure that all answers are based on the following instructions and do not need to add your subjective judgment. Finally, make sure your answers are completely correct.**

If 1.1 is NI, you need to double check if the article reports the use of computer-generated random numbers, block randomization, random number tables, and other methods to generate random sequences. If so, then 1.1 is Y

If the answer to question 1.2 is NI, you need to carefully check if the article mentions central allocation, envelopes and postcard, If so, then 1.2 is Y

The article does not report any deviations from the intended interventions.

If the proportion of the number of participants reported in the result analysis to the number of participants after randomization, and no clear evidence to suggest that the study used incorrect analytical methods, the answer to 2.6 question can not be "N" or "PN";

If there is no clear evidence that the article used appropriate methods such as ITT, mITT, etc., the answer to 2.6 cannot be Y or PY

The answer to 3.2 question can not be "NI";

Intention-to-treat analysis is not a method for correcting bias caused by missing data, and 3.2 cannot be used to determine Y just because the article used Intention-to-treat analysis

If there is no clear evidence to prove assessment of the outcome was influenced by knowledge of intervention receive, the answer to 4.5 is N;

If the article has not been registered or planned for publication, the answer to 5.1 is NI;

**Adhering**

Risk-of-bias assessment prompt

Introduction and Role Setting:

You are a professional reviewer. You are particularly good at learning evaluation criteria, and closely following it to assess the risk of bias of Randomized Controlled Trials (RCTs). I will give you a command, you can fully understand and follow the command and evaluate the RCTs I have provided to you. Finally, make sure your answers are completely correct.

Guidelines for Evaluation:

Certainty Rating System:

You should use the following certainty rating system to qualify the confidence in your evaluations:

High Certainty (95-100%): The evaluation is based on clear, direct, and comprehensive information provided in the RCT.

Moderate Certainty (75-94%): The evaluation is based on information that is mostly clear and direct, but with some minor uncertainties or gaps.

Low Certainty (50-74%): The evaluation is based on limited or indirect information, with significant uncertainties or gaps.

Very Low Certainty (0-49%): The evaluation is based on sparse, unclear, or highly speculative information.

For each evaluation item, include the certainty rating along with the percentage to provide a clear indication of the confidence in the assessment.

Specific Requirements:

The examples provided in the tool are illustrative and do not cover all possible scenarios in real-world applications. Use your expert judgment to evaluate each item based on the information provided in the RCT, and do not rely solely on the examples.

Important:

• The evaluation should be conducted only for the outcome of *Health-related quality of life*

**1 Domain 1: Risk of bias arising from the randomization process**

**Content of Domain One assessment: There are a total of 3 questions in this domain.**

- 1. **Was the allocation sequence adequately generated?**

In this question, you must strictly rely on the information in the article and not make assumptions or implications about the information in the article

If the article describes the methods of sequence generating (computer-generated random numbers, block randomization, coin tossing, card or envelope shuffling, dice rolling, lot drawing, or minimization) were used, the answer to this question is "Y";

If the sequence was generated based on the odd or even date of birth, some rule based on the date (or day) of admission, or some rule based on hospital or clinic record number, the answer to this question is "PN".

If allocation was based on clinician judgment, participant preference, results of a series of laboratory tests, or availability of the intervention, the answer to this question is "N".

**1.2 Was the allocation sequence concealed until participants were enrolled and assigned to interventions?**

In this question, you must strictly rely on the information in the article and not make assumptions or implications about the information in the article

The most important: If does not mention assignment concealment, the answer to this question is “NI”, even if the study was described as a double-blind or triple blind study;

If the article mentions allocation concealing or masking (including central allocation, sequentially numbered drug containers of identical appearance, or opaque envelopes), participants may not be aware of the assignment, the answer to this question is "PY";

If the article mentions allocation concealing or masking (including central allocation, sequentially numbered drug containers of identical appearance, or opaque envelopes), participants may be aware of the assignment, the answer to this question is "PN";

If the article describes the specific methods of sequence concealing (including central allocation, sequentially numbered drug containers of identical appearance, or opaque envelopes) were used, the answer to this question is "Y";

If an open random allocation schedule was used or if assignment envelopes were used without appropriate safeguards (e.g., if envelopes were unsealed, non-opaque, or not sequentially numbered), the answer to this question is "N".

**1.3 Did baseline differences between intervention groups suggest a problem with the randomization process?**

If there are no significant differences between the intervention and control groups, or if any existing differences are attributed to random error, the answer to this question is "N";

If there are noticeable differences between the intervention and control groups that are attributed to randomization, including the following aspects: 1, The sample sizes between the two groups differ significantly beyond the expected allocation ratio, 2 There are statistically significant differences in baseline characteristics between the two groups, 3 The differences between the groups are substantial enough to introduce bias in the assessment of intervention effects, 4 One or more important prognostic factors or baseline measurements of outcome variables differ, and it's not due to random error, 5 Baseline characteristics are too similar to be explained by random error, then the answer to this question is "Y";

If there is no information in the article regarding differences in baseline characteristics between the intervention and control groups, the answer to this question is "NI".

**2 Domain 2: Bias Deviation from the Prespecified Interventions**

**Risk of bias due to deviations from the intended interventions**

**2.2** **Effect of assignment to intervention**

**2.1 Were participants aware of their assigned intervention during the trial?**

In this question, you need to combine the information provided in the article with your own professional knowledge to make a comprehensive judgment on the question.

If participants were aware of their allocation, the answer to this question is "Y";

If there is handling of potential side effects or toxicity of interventions suffered by participants, or if there are other circumstances that may lead participants to know their allocation, the answer to this question is "PY";

If participants were unaware of their allocation, the answer to this question is "N";

If the article states that participants received a placebo or sham intervention as part of the blinding procedure, the answer to this question is "N";

If the article states that this is a non blind or open label experiment, the answer to this question is "N";

If there is no information in the article regarding blinding of participants, the answer to this question is "NI".

**2.2 Were carers and people delivering the interventions aware of participants' assigned intervention during the trial?**

In this question, you need to combine the information provided in the article with your own professional knowledge to make a comprehensive judgment on the question.

If the intervention providers were aware of the participants' allocation, the answer to this question is "Y";

If there is handling of potential side effects or toxicity of interventions suffered by participants, or if there are other circumstances that may lead intervention providers to know the participants' allocation, the answer to this question is "PY";

If intervention providers were unaware of the participants' allocation, or if the article states that intervention providers administered a placebo or sham intervention as part of the blinding procedure, the answer to this question is "N";

If there is no information in the article regarding blinding of intervention providers, the answer to this question is "NI".

**2.3 Were important nonprotocol interventions balanced across intervention groups?**

In this question, you need to combine the information provided in the article with your own professional knowledge to make a comprehensive judgment on the question.

If there are significant non protocol measures in the trial, such as (1) intervention are not inconsistent with the trial protocol; (2) trial participants might receive with or after starting their assigned intervention; and (3) are prognostic for the outcome; and they are imbalanced between groups, the answer to this question is "N";

If there are not significant non protocol measures in the trial, the answer to this question is "Y";

If the article does not provide any information that can help you make a judgment on the question, the answer to this question is "NI".

**2.4 Were there failures in implementing the intervention that could have affecte?**

In this question, you need to combine the information provided in the article with your own professional knowledge to make a comprehensive judgment on the question.

If more than 10% of the participants have failures in implementing the intervention, for example, Withdrawing from the trial due to reasons such as death, worsening of the condition, and doctor's advice, the answer to this question is "Y".

If implementation of the intervention was successful for most participants, the answer to this question is "N".

If the article does not provide any information that can help you make a judgment on the question, the answer to this question is "NI".

**2.5 Was there non-adherence to the assigned intervention regimen that could have affected participants’ outcomes?**

In this question, you need to combine the information provided in the article with your own professional knowledge to make a comprehensive judgment on the question.

If more than 10% of the participants have non-adherence to the assigned intervention, such as imperfect compliance with a sustained intervention, cessation of intervention, crossovers to the comparator intervention and switches to another active intervention, the answer to this question is "Y";

If there are not non-adherence to the assigned intervention in the trial, the answer to this question is "N";

If the article does not provide any information that can help you make a judgment on the question, the answer to this question is "NI";

**2.6 Was an appropriate analysis used to estimate the effect of adhering to the intervention?**

In this question, you need to combine the information provided in the article with your own professional knowledge to make a comprehensive judgment on the question.

If the article used appropriate analytical methods to evaluate the effectiveness of persistent interventions, like: (1) instrumental variable analyses to estimate the effect of receiving the assigned intervention in trials in which a single intervention, administered only at baseline and with all-or-nothing adherence, is compared with standard care; and (2) inverse probability weighting to adjust for censoring of participants who cease adherence to their assigned intervention, in trials of sustained treatment strategies, the answer to this question is "Y"

If the article used inappropriate analytical methods to evaluate the effectiveness of persistent interventions, like:(i) ‘Intention to treat (ITT) analysis’, (ii) ‘per protocol analysis’, (iii) ‘as-treated analysis’, (iv) ‘analysis by treatment received’, the answer to this question is "N"

If the article does not provide any information that can help you make a judgment on the question, the answer to this question is "NI".

**3 Domain 3: Risk of bias due to missing outcome data**

**Evaluation Content for Domain 3: Domain 3 comprises 4 questions.**

**3.1 Were data for this outcome available for all, or nearly all, participants randomized?**

In this question, you need to calculate the proportion of the number of participants in the result analysis to the total number of participants after randomization, and judge the answer of the question based on the calculated proportion:

If the outcome is a continuous variable and 95% or more of the randomized participants have complete outcome data (excluding imputed data), the answer is "Y";

If the outcome is a continuous variable and 90% to 95% of randomized participants have complete outcome data (excluding imputed data), the answer is "PN";

If the outcome is a continuous variable and the proportion of participants with complete outcome data after randomization is less than 90%, the answer is "N";

If the outcome is a binary variable and the number of observed events is much greater than the number of participants with missing outcomes, the answer is "Y";

If the outcome is a binary variable and the number of observed events is not much greater than the number of participants with missing outcomes, the answer is "N";

If the article does not provide the number of randomized individuals, the answer is "NI".

**3.2 Is there evidence that the result was not biased by missing outcome data?**

In this question, you need to combine the information provided in the article with your own professional knowledge to make a comprehensive judgment on the question.

If the article uses analysis methods such as inverse probability weighting that can correct for bias, or provides evidence through sensitivity analysis that outcomes were not biased due to missing data, the answer is "Y";

If no information is provided regarding the relationship between missing outcome data and bias correction methods or if inappropriate methods like last observation carried forward (LOCF) or imputation based solely on the intervention group were used, the answer is "N".

**3.3 Could missingness in the outcome depend on its true value?**

In this question, you need to combine the information provided in the article with your own professional knowledge to make a comprehensive judgment on the question.

If, based on the information provided in the article, it can be inferred that participants dropped out of the study and follow-up due to health issues related to the outcome variable, the answer is "Y";

If, based on the information provided in the article, it can be inferred that participants dropped out of the study and follow-up due to their health status, and does not specify whether it is related to the outcome variable, the answer is "PY";

If, based on the information provided in the article, it can be inferred that all missing outcome data is unrelated to the outcome itself, the answer is "N";

If the article does not provide any information that can help you make a judgment on the question, the answer is "NI".

**3.4 Is it likely that missingness in the outcome depended on its true value?**

In this question, you need to combine the information provided in the article with your own professional knowledge to make a comprehensive judgment on the question.

If the article contains any of the following situations: 1) There is a discrepancy in the proportion of missing outcome data between the intervention and control groups, and this discrepancy is related to the outcome variable; 2) The reported reasons for missing outcome data suggest a relationship with the missing outcome variable; 3) Different intervention groups have different reasons for missing outcome data; 4) The actual circumstances of the study suggest that missing outcome data is likely related to the outcome itself; 5) In time-to-event analysis, when a participants drops out of the study and follow-up due to drug toxicity or side effects, the answer is "Y"; If the analysis explains the characteristics of participants that may explain the relationship between missing outcome data and the outcome variable, the answer is "N";

If the article does not provide any information that can help you make a judgment on the question, the answer is "NI".

**4 Domain 4: Risk of bias in measurement of the outcome**

**Evaluation Content for Domain 4: Domain 4 comprises five questions.**

**4.1 Was the method of measuring the outcome inappropriate?**

In this question, you need to extract the measurement method of the outcome in the article and make a judgment based on your professional knowledge

If the current measurement method cannot reliably measure the intervention effect (e.g., the outcome measure is beyond the detection range of the measurement method) or if the measurement tool's reliability is poor, the answer is "Y";

If the outcome measure was predefined and the measurement tool is reliable, the answer is "N";

If the article does not provide the outcome measurement method of the outcome, the answer is "NI".

**4.2 Could measurement or ascertainment of the outcome have differed between intervention groups?**

In this question, you need to combine the information provided in the article with your own professional knowledge to make a comprehensive judgment on the question.

If the outcome variables for both groups should be measured in a comparable way, including using the same measurement method at comparable time points and having the same measurement thresholds, the answer is "N";

If there are differences in measurement between the two groups, such as more frequent visits in the intervention group, the answer is "Y";

If the article does not provide any information that can help you make a judgment on the question, the answer is "NI".

**4.3 Were outcome assessors aware of the intervention received by study participants?**

In this question, you need to combine the information provided in the article with your own professional knowledge to make a comprehensive judgment on the question;

If the outcome measurement is based on patient reported results and the participants were aware of their own group assignments, the answer is "Y";

If the outcome measurement is not based on patient reported results and the outcome assessors were aware of the participants' group assignments, the answer is "Y";

If the outcome measurement is based on patient reported results and participants are not aware of their group assignments, the answer is "N";;

If the outcome measurement is based on patient reported results and the article states that blinding methods like placebo or sham intervention were employed for the participants, the answer is "N";

If the outcome measurement is not based on patient reported results and the outcome assessors were not aware of the participants' group assignments, the answer is "N";

If the outcome measurement is not based on patient reported results and the article states that blinding methods like placebo or sham intervention were employed for the outcome assessors, the answer is "N";

If the article does not provide any information that can help you make a judgment on the question, the answer is "NI".

**4.4 Could assessment of the outcome have been influenced by knowledge of intervention received?**

In this question, you need to determine whether the outcome is subjective or objective.

If the outcome is an objective measure, such as all-cause mortality, the answer is "N";

If the outcome is a subjective measure and the measurement method does not specify whether it was assessed by the outcome assessor or reported by the patient, the answer is "PY";

If the outcome is a subjective measure reported by the patient, such as patient-reported pain level, the answer is "Y";

If the outcome is a subjective measure assessed by the outcome assessor, the answer is "Y";

If the article does not report a measurement method for the outcome, the answer is "NI".

**4.5 Is it likely that assessment of the outcome was influenced by knowledge of intervention received?**

In this question, you need to combine the information provided in the article with your own professional knowledge to make a comprehensive judgment on the question

If there is no clear evidence to suggest that knowledge of intervention status was likely to influence outcome assessment, the answer is "N";

If there is clear evidence to suggest that knowledge of intervention status was likely to influence outcome assessment, (such as patient-reported symptoms in trials of homeopathy, or assessments of recovery of function by a physiotherapist who delivered the intervention), the answer is "Y";

If the article does not provide any information about this can help you make a judgment on the question, the answer is "NI";

**Domain 5: Risk of bias in selection of the reported result**

**Evaluation Content for Domain 5: Domain 5 comprises three questions:**

**5.1 Were the data that produced this result analysed in accordance with a pre-specified analysis plan that was finalized before unblinded outcome data were available for analysis?**

In this question, you need to extract the measurement and the analyses method for all outcomes reported in the Methods section of the article and compare them with the measurement method for all outcomes reported in the Results section

If the measurement and analysis methods of all results in the results section are consistent with those of all results in the methods section, and all outcomes reported in the methods section are reported in the results section, the answer to the question is "Y";

If the measurement and analysis methods of all results in the results section are inconsistent with those of all results in the methods section, or if there is an outcome mentioned in the methods section that is not reported in the results section, but it can be clearly established that the changes to the plan are unrelated to the results, the answer to the question is "Y";

If the measurement and analysis methods of all results in the results section are inconsistent with those of all results in the methods section, or if there is an outcome mentioned in the methods section that is not reported in the results section, in addition, it can be clearly established that the changes to the plan are related to the results, the answer to the question is "N";

If the article does not provide any information about the measurement and the analyses method of outcomes that can help you make a judgment on the question, the answer to the question is "NI".

**5.2 Is the numerical result being assessed likely to have been selected, on the basis of the**

**results, from multiple eligible outcome measurements (e.g. scales, definitions, time points) within the outcome domain?**

In this question, you need to extract the measurement method for this outcome reported in the Methods section of the article and compare them with the measurement method for this outcome reported in the Results section

If there is clear evidence that multiple measurements were taken for the outcomes, but only one or a few of these measurements are fully reported, the answer to this question is "Y";

If all eligible reported results for the outcome domain correspond to all intended outcome measurements, the answer to this question is "N";

If there is only one possible way in which the outcome domain can be measured (hence there is no opportunity to select from multiple measures), the answer to this question is "N";

If outcome measurements are inconsistent across different reports on the same trial, but the article have provided the reason for the inconsistency and it is not related to the nature of the results, the answer to this question is "N";

If the article does not provide any information that can help you make a judgment on the question, the answer to this question is "NI".

**5.3 Is the numerical result being assessed likely to have been selected, on the basis of the**

**results, from multiple eligible analyses of the data?**

In this question, you need to extract the analyses method reported in the Methods section of the article and compare them with the analyses method reported in the Results section

you need to compare the analyses method reported in the Methods section of the article with the outcome measurement method reported in the Results section.

If there is clear evidence that multiple analysis methods were used for the results, but only one or a few of these analyses are fully reported, the answer to this question is "Y";

If there is clear evidence that all eligible reported results for the outcome measurement correspond to all intended analyses., the answer is "N";

If there is only one possible way in which the outcome measurement can be analysed (hence there is no opportunity to select from multiple analyses).

If the article does not provide any information that can help you make a judgment on the question, the answer to this question is "NI".

**Domain evaluate**

**Please strictly follow the following requirements for the domain evaluation section and do not include any assumptions or reflections**

**1 Evaluation Results for Domain 1:**

If the answer to question 1.2 is "Y" or "PY" and the answer to question 1.1 is "Y", "NI" or "PY" and the answer to question 1.1.3 is "N", "PN" or "NI", then the assessment result for Domain 1 is "Low risk";

If the answer to question 1.2 is "Y" or "PY", and the answer to question 1.1 is "Y" , "NI" or "PY", and the answer to question 1.1.3 is "Y" or "PY", then the assessment result for Domain 1 is "some concerns";

If the answer to question 1.2 is "Y" or "PY", and the answer to question 1.1 is "N" or "PN", then the assessment result for Domain 1 is "some concerns";

If the answer to question 1.2 is "NI", and the answer to question 1.3 is "N", "NI" or "PN", then the assessment result for Domain 1 is " some concerns ";

If the answer to question 1.2 is "NI", and the answer to question 1.3 is "Y" or "PY", then the assessment result for Domain 1 is "High risk";

If the answer to question 1.2 is "N" or "PN", then the assessment result for Domain 1 is "High risk".

**2 Assessment of Domain 2:**

If the answer to question 2.1 is "N" or "PN", the answer to question 2.2 is "N" or "PN", the answer to question 2.4 is "NA", "N" or "PN", and the answer to question 2.5 is "NA", "N" or "PN", then the result for Domain 2 is "Low risk";

If the answer to one of questions 2.1 and 2.2 is "Y", "PY" or "NI", the answer to question 2.3 is "NA", "Y" or "PY", the answer to question 2.4 is "NA", "N" or "PN", and the answer to question 2.5 is "NA", "N" or "PN", then the result for Domain 2 is "Low risk";

If the answer to one of questions 2.1 and 2.2 is "Y", "PY" or "NI", the answer to question 2.3 is "NA", "Y" or "PY", the answer to one of questions 2.4 and 2.5 is "Y", "PY" or "NI",, and the answer to question 2.6 is "Y" or "PY", then the result for Domain 2 is "Some concerns";

If the answer to one of questions 2.1 and 2.2 is "Y", "PY" or "NI", the answer to question 2.3 is "NI", "N" or "PN", and the answer to question 2.6 is "Y" or "PY", then the result for Domain 2 is "Some concerns";

If the answer to one of questions 2.1 and 2.2 is "Y", "PY" or "NI", the answer to question 2.3 is "NA", "Y" or "PY", the answer to one of questions 2.4 and 2.5 is "Y", "PY" or "NI", and the answer to question 2.6 is "NI", "N" or "PN", then the result for Domain 2 is "High risk";

If the answer to one of questions 2.1 and 2.2 is "Y", "PY" or "NI", the answer to question 2.3 is "NI", "N" or "PN", and the answer to question 2.6 is "NI", "N" or "PN", then the result for Domain 2 is "High risk";

**3 Assessment Result for Domain 3:**

If the answer to question 3.1 is "Y" or "PY", then the assessment result for Domain 3 is "Low risk";

If the answer to question 3.1 is "N", "PN" or "NI", and the answer to question 3.2 is "Y" or "PY", then the assessment result for Domain 3 is "Low risk";

If the answer to question 3.1 is "N", "PN" or "NI", the answer to question 3.2 is "N" or "PN", and the answer to question 3.3 is "N" or "PN", then the assessment result for Domain 3 is "Low risk";

If the answer to question 3.1 is "N", "PN" or "NI", and the answer to question 3.2 is "N" or "PN", and the answer to question 3.3 is "Y", "PY" or "NI", and the answer to question 3.4 is "N" or "PN", then the assessment result for Domain 3 is "Some concerns";

If the answer to question 3.1 is "N", "PN" or "NI", and the answer to question 3.2 is "N" or "PN", and the answer to question 3.3 is "Y", "PY" or "NI", and the answer to question 3.4 is "Y", "PY" or "NI", the assessment result for Domain 3 is "High risk".

**4 Assessment Result for Domain 4:**

If the answer to question 4.1 is "N", "PN" or "NI", the answer to question 4.2 is "N" or "PN", and the answer to question 4.3 is "N" or "PN", then the assessment result for Domain 4 is "Low risk";

If the answer to question 4.1 is "N", "PN" or "NI", the answer to question 4.2 is "N" or "PN", the answer to question 4.3 is "Y", "PY" or "NI", and the answer to question 4.4 is "N" or "PN", then the assessment result for Domain 4 is "Low risk";

If the answer to question 4.1 is "N", "PN" or "NI", the answer to question 4.2 is "N" or "PN", the answer to question 4.3 is "Y", "PY" or "NI", the answer to question 4.4 is "Y", "PY" or "NI", and the answer to question 4.5 is "N" or "PN", then the assessment result for Domain 4 is "Some concerns";

If the answer to question 4.1 is "N", "PN" or "NI", the answer to question 4.2 is "NI", the answer to question 4.3 is "N" or "PN", then the assessment result for Domain 4 is "Some concerns";

If the answer to question 4.1 is "N", "PN" or "NI", the answer to question 4.2 is "NI", the answer to question 4.3 is "Y", "PY" or "NI", the answer to question 4.4 is "N" or "PN", then the assessment result for Domain 4 is "Some concerns";

If the answer to question 4.1 is "N", "PN" or "NI", the answer to question 4.2 is "NI", the answer to question 4.3 is "Y", "PY" or "NI", the answer to question 4.4 is "Y", "PY" or "NI", and the answer to question 4.5 is "N" or "PN", then the assessment result for Domain 4 is "Some concerns";

If the answer to question 4.1 is "N", "PN" or "NI", the answer to question 4.2 is "N" or "PN", and the answer to question 4.3 is "Y", "PY" or "NI", and the answer to question 4.4 is "Y", "PY" or "NI", and the answer to question 4.5 is "Y", "PY" or "NI", then the assessment result for Domain 4 is "High risk";

If the answer to question 4.1 is "N", "PN" or "NI", the answer to question 4.2 is "NI", the answer to question 4.3 is "Y", "PY" or "NI", the answer to question 4.4 is "Y", "PY" or "NI", and the answer to question 4.5 is "Y", "PY" or "NI", then the assessment result for Domain 4 is "High risk";

If the answer to question 4.1 is "N", "PN" or "NI", the answer to question 4.2 is "Y" or "PY", and the answer to question 4.3 is "Y", "PY", then the assessment result for Domain 4 is "High risk";

If the answer to question 4.1 is "Y" or "PY", then the assessment result for Domain 4 is "High risk".

**5 Domain 5 Assessment Results:**

If the answer to question 5.2 is "N" or "PN", the answer to question 5.3 is "N" or "PN", and the answer to question 5.1 is "Y" or "PY", then the assessment result for Domain 5 is "Low Risk";

If the answer to question 5.2 is "N" or "PN", the answer to question 5.3 is "N" or "PN", and the answer to question 5.1 is "N", "PN" or "NI", then the assessment result for Domain 5 is "Some concerns";

If the answer to question 5.2 is "N", "PN" or "NI", the answer to question 5.3 is "NI", the assessment result for Domain 5 is "Some concerns";

If the answer to question 5.2 is "NI", the answer to question 5.3 is "N", "PN" or "NI", then the assessment result for Domain 5 is "Some concerns";

If the answer to question 5.2 is "Y" or "PY", then the assessment result for Domain 5 is "High Risk";

If the answer to question 5.3 is "Y" or "PY", then the assessment result for Domain 5 is "High Risk ".

**6 Overall Bias Risk Assessment Result for the Article:**

If all five domain assessment results are "Low Risk", then the overall assessment result for the article is "Low Risk";

If one domain assessment result is "Some concerns", and there are no domain assessment results that are "High Risk", with at least two being "Low Risk", then the overall assessment result for the article is "Some concerns";

If one domain assessment result is "High Risk", then the overall assessment result for the article is "High Risk";

If all five domain assessment results that are "Some concerns", then the overall assessment result for the article is "High Risk".

**Output Format:**

**Article ID**: [Insert First Author's Last Name], [Insert Year of Publication]

**Outcome Name**: [Insert Outcome Name]

**registration number**: [Insert registration number]

Domain 1

1.1 : Result of the Question (percentage of Confidence)

Reason you summarized

(Continue in the same format for all Question in this Domain)

Result of the domain (percentage of Confidence)

Reason you summarized

(Continue in the same format for all Domain)

Overall Bias Risk for the Article (percentage of Confidence)

**Please ensure that all answers are based on the following instructions and do not need to add your subjective judgment. Finally, make sure your answers are completely correct.**

If 1.1 is NI, you need to double check if the article reports the use of computer-generated random numbers, block randomization, random number tables, and other methods to generate random sequences. If so, then 1.1 is Y

If the answer to question 1.2 is NI, you need to carefully check if the article mentions central allocation, envelopes and postcard, If so, then 1.2 is Y

If more than 10% of the participants have failures in implementing, such as withdrawing from the trial, 2.4 is "Y".

If more than 10% of the participants have non-adherence to the assigned intervention, 2.5 is "Y";

If no information reported regarding adherence in article, 2.5 is NI

If 2.6 is NI or Y, you need to double check if the analysis method of the article report is Intention to treat (ITT) analysis, per protocol analysis, as-treated analysis or analysis by treatment received, and other methods to generate random sequences. If so, then 2.6 is N

The answer to 3.2 can not be "NI"

Intention-to-treat analysis is not a method for correcting bias caused by missing data, and 3.2 cannot be used to determine Y just because the article used Intention-to-treat analysis

If there is no clear evidence to prove assessment of the outcome was influenced by knowledge of intervention receive,the the answer to 4.5 is N;

If the article has not been registered or planned for publication, the answer to 5.1 is NI;

Appendix 2. Output results for cluade-3

## Study 1-1

Here is my revised assessment of the risk of bias for this randomized controlled trial, focusing on the Quality of Life outcome:

**Article ID**: Pressler, 2016

**Outcome Name**: Quality of Life

**Registration number**: NCT01935297

Domain 1: Risk of bias arising from the randomization process

1.1: Was the allocation sequence random? PY (75% certainty)

The article states patients were "randomly allocated 1:1 according to a computer-generated allocation program code".

1.2: Was the allocation sequence concealed until participants were enrolled and assigned to interventions? NI (50% certainty)

The article does not provide any information about allocation concealment.

1.3: Did baseline differences between intervention groups suggest a problem with the randomization process? N (80% certainty)

Table I shows baseline characteristics were similar between groups, with no statistically significant differences noted.

Result of Domain 1: Some concerns (70% certainty)

While a computer-generated randomization sequence was used and baseline characteristics appear balanced, key details about allocation concealment are not reported.

Domain 2: Risk of bias due to deviations from intended interventions

2.1: Were participants aware of their assigned intervention during the trial? Y (90% certainty)

Given the nature of the exercise intervention, participants would have been aware of their group assignment.

2.2: Were carers and people delivering the interventions aware of participants' assigned intervention during the trial? Y (90% certainty)

Those delivering the exercise intervention would necessarily be aware of group assignments.

2.3: Were there deviations from the intended intervention that arose because of the trial context? NI (75% certainty)

The article does not report any deviations from the intended interventions.

2.4: If Y/PY to 2.3: Were these deviations likely to have affected the outcome? NA

2.5: If Y/PY to 2.4: Were these deviations from intended intervention balanced between groups? NA

2.6: Was an appropriate analysis used to estimate the effect of assignment to intervention? Y (80% certainty)

The article states a per-protocol analysis was performed, which is appropriate given the low number of dropouts unrelated to the intervention.

2.7: If N/PN/NI to 2.6: Was there potential for a substantial impact (on the result) of the failure to analyse participants in the group to which they were randomized? NA

Result of Domain 2: Low risk (80% certainty)

While participants and providers were aware of assignments, this is unavoidable for an exercise intervention. The analysis appears appropriate given the circumstances.

Domain 3: Missing outcome data

3.1: Were data for this outcome available for all, or nearly all, participants randomized? Y (90% certainty)

27 of 30 randomized participants completed the study, which is 90%.

3.2: If N/PN/NI to 3.1: Is there evidence that the result was not biased by missing outcome data? NA

3.3: If N/PN to 3.2: Could missingness in the outcome depend on its true value? NA

3.4: If Y/PY/NI to 3.3: Is it likely that missingness in the outcome depended on its true value? NA

Result of Domain 3: Low risk (90% certainty)

There was minimal missing outcome data, with 90% of randomized participants included in the analysis.

Domain 4: Risk of bias in measurement of the outcome

4.1: Was the method of measuring the outcome inappropriate? N (80% certainty)

Quality of life was measured using validated questionnaires (KCCQ and SF-12) that are appropriate for this population.

4.2: Could measurement or ascertainment of the outcome have differed between intervention groups? N (80% certainty)

The article does not suggest any differences in how quality of life was measured between groups.

4.3: Were outcome assessors aware of the intervention received by study participants? Y (90% certainty)

Quality of life was measured by self-report questionnaires, so participants (who were aware of their intervention) were the outcome assessors.

4.4: If Y/PY/NI to 4.3: Could assessment of the outcome have been influenced by knowledge of intervention received? Y (90% certainty)

Self-reported quality of life could be influenced by participants' knowledge of their intervention assignment.

4.5: If Y/PY/NI to 4.4: Is it likely that assessment of the outcome was influenced by knowledge of intervention received? PY (70% certainty)

It's plausible that participants' expectations about the effects of exercise could have influenced their quality of life ratings.

Result of Domain 4: High risk (80% certainty)

The self-reported nature of the outcome and participants' awareness of intervention creates high risk of bias in outcome measurement.

Domain 5: Risk of bias in selection of the reported result

5.1: Were the data that produced this result analysed in accordance with a pre-specified analysis plan that was finalized before unblinded outcome data were available for analysis? Y (50% certainty)

The study was pre-registered on ClinicalTrials.gov (NCT01935297) and the analysis plan seems pre-specified.

5.2: Is the numerical result being assessed likely to have been selected, on the basis of the results, from multiple eligible outcome measurements within the outcome domain? N (75% certainty)

The article reports results for all subscales of the quality of life measures, suggesting no selective reporting of favorable outcomes.

5.3: Is the numerical result being assessed likely to have been selected, on the basis of the results, from multiple eligible analyses of the data? N (75% certainty)

The article does not suggest multiple analyses were conducted with only favorable results reported.

Result of Domain 5: Low risk (70% certainty)

While there's no clear evidence of selective reporting.

Overall Bias Risk for the Article: High risk (80% confidence)

The overall risk of bias is high, primarily due to the high risk in Domain 4 (measurement of the outcome). The self-reported nature of quality of life measurement in an unblinded trial creates significant potential for bias. There are also some concerns about the randomization process and analysis plan, though these are less severe. While the study provides valuable pilot data on exercise after TAVI, the results should be interpreted cautiously given these methodological limitations.

## Study 1-2

Here is my assessment of the risk of bias for this randomized controlled trial, focusing on the Quality of Life outcome:

**Article ID**: Pressler, 2016

**Outcome Name**: Quality of Life

**Registration number**: NCT01935297

Domain 1: Risk of bias arising from the randomization process

1.1: Was the allocation sequence random? NI (50% certainty)

The article only mentions random allocation without describing the specific method used to generate the random sequence.

1.2: Was the allocation sequence concealed until participants were enrolled and assigned to interventions? NI (50% certainty)

The article does not provide any information about allocation concealment.

1.3: Did baseline differences between intervention groups suggest a problem with the randomization process? N (80% certainty)

Table I shows baseline characteristics were similar between groups, with no statistically significant differences noted.

Result of Domain 1: Some concerns (70% certainty)

While baseline characteristics appear balanced, key details about the randomization process are not reported.

Domain 2: Risk of bias due to deviations from intended interventions

2.1: Were participants aware of their assigned intervention during the trial? Y (90% certainty)

Given the nature of the exercise intervention, participants would have been aware of their group assignment.

2.2: Were carers and people delivering the interventions aware of participants' assigned intervention during the trial? Y (90% certainty)

Those delivering the exercise intervention would necessarily be aware of group assignments.

2.3: Were there deviations from the intended intervention that arose because of the trial context? N (75% certainty)

The article does not report any deviations from the intended interventions.

2.4: If Y/PY to 2.3: Were these deviations likely to have affected the outcome? NA

2.5: If Y/PY to 2.4: Were these deviations from intended intervention balanced between groups? NA

2.6: Was an appropriate analysis used to estimate the effect of assignment to intervention? Y (80% certainty)

The article states a per-protocol analysis was performed, which is appropriate given the low number of dropouts unrelated to the intervention.

2.7: If N/PN/NI to 2.6: Was there potential for a substantial impact (on the result) of the failure to analyse participants in the group to which they were randomized? NA

Result of Domain 2: Low risk (80% certainty)

While participants and providers were aware of assignments, this is unavoidable for an exercise intervention. The analysis appears appropriate given the circumstances.

Domain 3: Missing outcome data

3.1: Were data for this outcome available for all, or nearly all, participants randomized? Y (90% certainty)

27 of 30 randomized participants completed the study, which is over 90%.

3.2: If N/PN/NI to 3.1: Is there evidence that the result was not biased by missing outcome data? NA

3.3: If N/PN to 3.2: Could missingness in the outcome depend on its true value? NA

3.4: If Y/PY/NI to 3.3: Is it likely that missingness in the outcome depended on its true value? NA

Result of Domain 3: Low risk (90% certainty)

There was minimal missing outcome data, with over 90% of randomized participants included in the analysis.

Domain 4: Risk of bias in measurement of the outcome

4.1: Was the method of measuring the outcome inappropriate? N (80% certainty)

Quality of life was measured using validated questionnaires (KCCQ and SF-12) that are appropriate for this population.

4.2: Could measurement or ascertainment of the outcome have differed between intervention groups? N (80% certainty)

The article does not suggest any differences in how quality of life was measured between groups.

4.3: Were outcome assessors aware of the intervention received by study participants? Y (90% certainty)

Quality of life was measured by self-report questionnaires, so participants (who were aware of their intervention) were the outcome assessors.

4.4: If Y/PY/NI to 4.3: Could assessment of the outcome have been influenced by knowledge of intervention received? Y (90% certainty)

Self-reported quality of life could be influenced by participants' knowledge of their intervention assignment.

4.5: If Y/PY/NI to 4.4: Is it likely that assessment of the outcome was influenced by knowledge of intervention received? PY (70% certainty)

It's plausible that participants' expectations about the effects of exercise could have influenced their quality of life ratings.

Result of Domain 4: High risk (80% certainty)

The self-reported nature of the outcome and participants' awareness of intervention creates high risk of bias in outcome measurement.

Domain 5: Risk of bias in selection of the reported result

5.1: Were the data that produced this result analysed in accordance with a pre-specified analysis plan that was finalized before unblinded outcome data were available for analysis? Y (50% certainty)

The study was pre-registered on ClinicalTrials.gov (NCT01935297) and the analysis plan seems pre-specified.

5.2: Is the numerical result being assessed likely to have been selected, on the basis of the results, from multiple eligible outcome measurements within the outcome domain? N (75% certainty)

The article reports results for all subscales of the quality of life measures, suggesting no selective reporting of favorable outcomes.

5.3: Is the numerical result being assessed likely to have been selected, on the basis of the results, from multiple eligible analyses of the data? N (75% certainty)

The article does not suggest multiple analyses were conducted with only favorable results reported.

Result of Domain 5: Low risk (70% certainty)

While there's no clear evidence of selective reporting.

Overall Bias Risk for the Article: High risk (80% confidence)

The overall risk of bias is high, primarily due to the high risk in Domain 4 (measurement of the outcome). The self-reported nature of quality of life measurement in an unblinded trial creates significant potential for bias. There are also some concerns about the randomization process and analysis plan, though these are less severe. While the study provides valuable pilot data on exercise after TAVI, the results should be interpreted cautiously given these methodological limitations.

## Study 2-1

**Article ID**: Ireland, 2017

**Outcome Name**: Stress rate

Domain 1

1.1 : NI (High Certainty: 95%)

The article only mentions that participants were randomly assigned to the intervention or control group, without providing any details on the specific methods used to generate the random allocation sequence.

1.2 : NI (High Certainty: 95%)

The article does not mention any methods used to conceal the allocation sequence, such as central allocation or opaque envelopes distribution.

1.3 : N (High Certainty: 90%)

The article does not report any significant baseline differences between the intervention and control groups that would suggest problems with the randomization process. It states that conditions were equivalent at pretest for prior experience with, appeal for, and expectations of mindfulness.

Result of the domain: Some concerns (High Certainty: 95%)

Based on the answers to the signaling questions, especially the lack of information on allocation sequence generation and concealment, there are some concerns regarding risk of bias arising from the randomization process. The groups appeared similar at baseline, but more information would be needed for a low risk of bias judgment.

Domain 2

2.1 : Y (High Certainty: 95%)

As an open-label trial where the intervention involved weekly mindfulness training workshops, the participants were almost certainly aware of their assigned condition during the trial.

2.2 : Y (High Certainty: 95%)

The article states that participants in the intervention group were given weekly one-hour training workshops, so the facilitators delivering the intervention were aware of the participants' assigned condition.

2.3 : NI (High Certainty: 95%)

The article does not report whether there were any deviations from the intended intervention that arose because of the trial context.

2.4 : NI (High Certainty: 95%)

There is no information provided in the article to judge whether any deviations from intended intervention were likely to have affected the outcome.

2.5 : NI (High Certainty: 95%)

There is no information provided in the article to judge whether any deviations from intended intervention were balanced between the intervention and control groups.

2.6 : PY (High Certainty: 95%)

The article does not explicitly state the analysis method, but the results include all randomized participants (23 in intervention, 21 in control), suggesting an intention-to-treat approach. There is no indication of incorrect analysis methods that would impact the number analyzed.

2.7 : N (High Certainty: 95%)

The analysis includes all randomized participants, so there is no indication of a failure to analyze participants in their assigned groups that would have a substantial impact on results.

Overall Assessment of Domain 2: Some concerns (High Certainty: 95%)

There are some concerns regarding risk of bias due to deviations from intended interventions. The trial was open-label and there is no information on whether deviations occurred or how they were handled, but an appropriate analysis including all randomized participants appears to have been used.

Domain 3

3.1 : Y (High Certainty: 95%)

The article reports the number of participants analyzed for the stress outcome at each time point, which is the same as the number randomized to each group (23 in intervention, 21 in control). This indicates outcome data was available for all randomized participants.

3.2 : N (Moderate Certainty: 80%)

No evidence of methods to correct for bias due to missing data..

3.3 : PN (Moderate Certainty: 80%)

The article does not discuss reasons for missing outcome data. As data was available for all participants, it seems unlikely missingness would depend on the true value, but there is some uncertainty.

3.4 : PN (Moderate Certainty: 80%)

The article does not discuss reasons for missing outcome data. As data was available for all participants, it seems unlikely missingness would depend on the true value, but there is some uncertainty.

Result of the domain: Low risk (High Certainty: 90%)

Outcome data was available for all randomized participants, so risk of bias due to missing outcome data appears low. There is no clear indication that missing data was extensive or related to the outcome, but some uncertainty remains given the limited information provided.

Domain 4

4.1 : PN (High Certainty: 95%)

The article states that perceived stress was measured using the 10-item Perceived Stress Scale (PSS), which is a validated self-report scale. This appears to be an appropriate method for measuring the stress outcome.

4.2 : N (High Certainty: 95%)

There is no indication that measurement or ascertainment of the stress outcome differed between the intervention and control groups. The same self-report scale appears to have been administered to both groups at the same three timepoints.

4.3 : Y (High Certainty: 90%)

While the article does not explicitly state whether outcome assessors (i.e., participants providing self-report data) were aware of the intervention received, it is very likely they were aware given the open-label nature of the trial.

4.4 : Y (High Certainty: 95%)

Since stress was measured by self-report and participants were likely aware of their intervention assignment, assessment of the stress outcome could have been influenced by knowledge of the intervention received.

4.5 : N (High Certainty: 95%)

While the self-reported nature of the stress outcome and lack of blinding could have influenced responses, there is no clear evidence provided in the article that this was likely to have occurred.

Result of the domain: High risk (High Certainty: 95%)

There is a high risk of bias in measurement of the stress outcome. While an appropriate self-report measure was used consistently across groups, participants were likely unblinded, which could have influenced their responses on this subjective measure. Lack of blinding of outcome assessors (participants) introduces a high risk of bias.

Domain 5

5.1 : NI (High Certainty: 95%)

The article does not mention a pre-specified analysis plan, so it is unclear if the reported results were analyzed in accordance with such a plan that was finalized before unblinded outcome data were available.

5.2 : PN (High Certainty: 95%)

The article reports using the Perceived Stress Scale measured at three time points. There is no clear indication that the reported results for stress were selected from multiple eligible outcome measurements.

5.3 : PN (High Certainty: 95%)

The article reports results based on multilevel modeling of the stress outcome measured at three timepoints. There is no clear indication that the reported results were selected from multiple eligible analyses.

Result of the domain: Some concerns (High Certainty: 95%)

There are some concerns regarding risk of bias in selection of the reported stress results. The lack of a pre-specified analysis plan makes it difficult to determine if the reported outcome measurements and analyses were selectively chosen, but there are no clear indications of selective reporting.

Overall Bias Risk for the Article: High risk (High Certainty: 95%)

Based on the signaling questions, this trial is judged to be at high risk of bias for the stress outcome, primarily due to significant concerns about lack of participant blinding in this open-label trial which used a subjective self-reported outcome measure. There are also some concerns related to the randomization process and lack of a pre-specified analysis plan. While an appropriate analysis including all participants appears to have been used and missing data is not a major concern, the potential for bias in measurement of the subjective outcome in unblinded participants leads to an overall judgment of high risk of bias.

## Study 2-2

Based on the information provided in the study and following the evaluation criteria, here is my assessment of the risk of bias for the Ireland et al. 2017 article:

**Article ID**: Ireland, 2017

**Outcome Name**: Stress rate

Domain 1: Risk of bias arising from the randomization process

1.1: NI (75% confidence)

Reason: The article only mentions random assignment without specifying the method for generating the random sequence.

1.2: NI (75% confidence)

Reason: The article does not mention allocation concealment.

1.3: N (80% confidence)

Reason: The article states "Conditions were equivalent pretest" regarding several baseline characteristics, suggesting no significant differences between groups.

Result of Domain 1: Some concerns (80% confidence)

Reason: While randomization was mentioned, details on sequence generation and allocation concealment were not provided. However, baseline characteristics appeared balanced between groups.

Domain 2: Risk of bias due to deviations from the intended interventions

2.1: Y (90% confidence)

Reason: Participants were likely aware of their assigned intervention, as the control group received extra break time while the intervention group attended mindfulness training sessions.

2.2: Y (90% confidence)

Reason: Given the nature of the interventions, those delivering them were likely aware of participants' assignments.

2.3: NI (75% confidence)

Reason: The article does not report any deviations from the intended intervention.

2.4: NI (75% confidence)

Reason: No information is provided to judge whether potential deviations affected the outcome.

2.5: NI (75% confidence)

Reason: No information is provided to assess whether potential deviations were balanced between groups.

2.6: Y (85% confidence)

Reason: The article appears to have included all randomized participants in the analysis, based on the reported sample sizes.

2.7: N (85% confidence)

Reason: As all randomized participants seem to have been included in the analysis, there is likely no substantial impact from failure to analyze participants in their assigned groups.

Result of Domain 2: Some concerns (80% confidence)

Reason: While participants and intervention providers were likely aware of assignments, there is no clear evidence of deviations from intended interventions, and the analysis appears to have included all participants.

Domain 3: Missing outcome data

3.1: Y (90% confidence)

Reason: The article does not report any missing outcome data, and the analysis appears to include all 44 randomized participants.

3.2: N (75% confidence)

Reason: No information is provided about methods to handle missing data or sensitivity analyses.

3.3: N (85% confidence)

Reason: As all participants appear to have been included in the analysis, it is unlikely that any potential missing data depended on its true value.

3.4: N (85% confidence)

Reason: Given that all participants seem to have been included, it is unlikely that any potential missingness in the outcome depended on its true value.

Result of Domain 3: Low risk (85% confidence)

Reason: There appears to be complete outcome data for all or nearly all participants.

Domain 4: Risk of bias in measurement of the outcome

4.1: N (90% confidence)

Reason: The study used the Perceived Stress Scale, which is a validated measure for assessing stress.

4.2: N (85% confidence)

Reason: The outcome was likely measured in the same way for both groups, as the same scale was used.

4.3: Y (90% confidence)

Reason: As this was a self-reported outcome and participants were aware of their intervention, outcome assessors (the participants themselves) were aware of the intervention received.

4.4: Y (85% confidence)

Reason: Perceived stress is a subjective measure reported by the participants.

4.5: PY (75% confidence)

Reason: Given that participants were aware of their intervention and the outcome was subjective, there is a possibility that assessment could have been influenced by knowledge of the intervention received.

Result of Domain 4: High risk (80% confidence)

Reason: While the measurement tool was appropriate, the subjective nature of the outcome and participants' awareness of their intervention could have influenced the assessment.

Domain 5: Risk of bias in selection of the reported result

5.1: Y (85% confidence)

Reason: The analysis methods reported in the results section appear consistent with those described in the methods section.

5.2: N (80% confidence)

Reason: The study appears to report results for the pre-specified outcome measure (PSS) without evidence of selective reporting based on multiple outcome measurements.

5.3: N (80% confidence)

Reason: There is no evidence of multiple analyses of the data or selective reporting of analyses.

Result of Domain 5: Low risk (80% confidence)

Reason: The reported results appear to be consistent with the pre-specified analysis plan without evidence of selective reporting.

Overall Bias Risk for the Article: Some concerns (80% confidence)

Reason: While there are low risks in some domains (missing outcome data and selection of reported results), there are some concerns regarding the randomization process and deviations from intended interventions. The main issue is the high risk of bias in outcome measurement due to the subjective nature of the outcome and participants' awareness of their intervention. However, these concerns are somewhat mitigated by the use of a randomized controlled design and validated outcome measures..

## Study 3-1

Here is my assessment of the risk of bias for the specified outcome in the article:

**Article ID**: Beigel, 2020

**Outcome Name**: All-cause mortality at up to day 28

**Registration number**: NCT04280705

Domain 1: Risk of bias arising from the randomization process

1.1: Y (95% confidence)

Reason: The article states "Patients were randomly assigned in a 1:1 ratio to receive either remdesivir or placebo. Randomization was stratified by study site and disease severity at enrollment."

1.2: Y (95% confidence)

Reason: The article mentions "A matching placebo was administered according to the same schedule and in the same volume as the active drug. A normal saline placebo was used at the European sites and at some non-European sites owing to a shortage of matching placebo; for these sites, the remdesivir and placebo infusions were masked with an opaque bag and tubing covers to maintain blinding."

1.3: N (90% confidence)

Reason: The baseline characteristics table shows no significant differences between groups that would suggest a problem with randomization.

Result of Domain 1: Low risk (95% confidence)

Reason: Adequate random sequence generation and allocation concealment were described, and baseline differences do not suggest problems with the randomization process.

Domain 2: Risk of bias due to deviations from intended interventions

2.1: N (90% confidence)

Reason: The study is described as double-blind, and placebo was used to maintain blinding of participants.

2.2: N (90% confidence)

Reason: The study is described as double-blind, suggesting care providers were also blinded.

2.3: NI (80% confidence)

Reason: The article does not specifically report on deviations from intended interventions due to the trial context.

2.4: NI (80% confidence)

Reason: Without information on deviations, their impact cannot be determined.

2.5: NI (80% confidence)

Reason: Without information on deviations, balance between groups cannot be determined.

2.6: Y (90% confidence)

Reason: The article states "The primary analysis was a stratified log-rank test of time to recovery with remdesivir as compared with placebo, with stratification by disease severity." This suggests an appropriate intention-to-treat analysis.

2.7: N (85% confidence)

Reason: The article reports that 531 of 541 patients assigned to remdesivir and 517 of 521 assigned to placebo received the assigned treatment, indicating minimal exclusions from the analysis.

Result of Domain 2: Low risk (85% confidence)

Reason: The study was double-blinded, used appropriate analysis methods, and had minimal exclusions from the assigned groups.

Domain 3: Risk of bias due to missing outcome data

3.1: Y (95% confidence)

Reason: The article reports that 517 patients in the remdesivir group and 508 in the placebo group completed the trial through day 29, recovered, or died. This represents over 95% of randomized participants.

3.2: N (85% confidence)

Reason: There is no evidence of methods used to correct for bias due to missing data. However, with very little missing data, this is unlikely to be a significant source of bias.

3.3: PN (80% confidence)

Reason: With very little missing data, it's unlikely that missingness depends on the true value, though this is not explicitly stated.

3.4: PN (80% confidence)

Reason: Again, with very little missing data, it's unlikely that missingness depends on its true value to a degree that would impact results.

Result of Domain 3: Low risk (90% confidence)

Reason: There is very little missing outcome data, minimizing the risk of bias from this source.

Domain 4: Risk of bias in measurement of the outcome

4.1: N (95% confidence)

Reason: All-cause mortality is an objective outcome that is unlikely to be measured inappropriately.

4.2: N (95% confidence)

Reason: All-cause mortality should be measured consistently between groups.

4.3: N (90% confidence)

Reason: The study is described as double-blind, suggesting outcome assessors were likely unaware of intervention received.

4.4: N (95% confidence)

Reason: All-cause mortality is an objective outcome that is unlikely to be influenced by knowledge of the intervention received.

4.5: N (95% confidence)

Reason: As an objective outcome, assessment of all-cause mortality is very unlikely to be influenced by knowledge of the intervention received.

Result of Domain 4: Low risk (95% confidence)

Reason: All-cause mortality is an objective outcome unlikely to be biased by measurement or ascertainment issues.

Domain 5: Risk of bias in selection of the reported result

5.1: Y (90% confidence)

Reason: The trial was registered (NCT04280705) and the reported outcomes align with those pre-specified.

5.2: N (90% confidence)

Reason: All-cause mortality is a singular outcome measure without multiple ways to be measured.

5.3: N (90% confidence)

Reason: The article reports hazard ratios from a pre-specified Cox proportional hazards model, suggesting no selection among multiple analyses.

Result of Domain 5: Low risk (90% confidence)

Reason: The trial was pre-registered and the reported results align with the pre-specified measures and analyses.

Overall Bias Risk for the Article: Low risk (90% confidence)

The study demonstrates low risk of bias across all domains for the outcome of all-cause mortality at up to day 28. The randomization process was well-described, the study was double-blinded, there was minimal missing data, the outcome is objective and unlikely to be influenced by knowledge of treatment, and the reported results align with pre-specified measures. While some details are not explicitly reported (e.g., specific deviations from intended interventions), the overall design and conduct of the trial suggest a low risk of bias in the reported results.

## Study 3-2

Here is my assessment of the risk of bias for the Beigel et al. 2020 study on remdesivir for COVID-19:

**Article ID**: Beigel, 2020

**Outcome Name**: All-cause mortality at up to day 28

**Registration number**: NCT04280705

Domain 1: Risk of bias arising from the randomization process

1.1: Y (High Certainty, 95%)

The study states "Patients were randomly assigned in a 1:1 ratio to receive either remdesivir or placebo. Randomization was stratified by study site and disease severity at enrollment."

1.2: Y (High Certainty, 95%)

The study reports "A normal saline placebo was used at the European sites and at some non-European sites owing to a shortage of matching placebo; for these sites, the remdesivir and placebo infusions were masked with an opaque bag and tubing covers to maintain blinding."

1.3: N (High Certainty, 95%)

Table 1 shows baseline characteristics were well-balanced between groups.

Result of Domain 1: Low risk (High Certainty, 95%)

Randomization was appropriately conducted and allocation was concealed. Baseline differences do not suggest problems with the randomization process.

Domain 2: Risk of bias due to deviations from the intended interventions

2.1: N (High Certainty, 95%)

The study was double-blinded: "We conducted a double-blind, randomized, placebo-controlled trial of intravenous remdesivir in adults who were hospitalized with Covid-19"

2.2: N (High Certainty, 95%)

As above, the study was double-blinded.

2.3: PN (Moderate Certainty, 80%)

No clear deviations from intended interventions are reported. Some protocol-allowed deviations likely occurred but were balanced between groups.

2.4: PN (Moderate Certainty, 80%)

No evidence that any deviations affected the outcome.

2.5: PY (Moderate Certainty, 80%)

Any deviations were likely balanced between groups due to blinding.

2.6: Y (High Certainty, 95%)

The study used an intention-to-treat analysis: "The primary analysis was a stratified log-rank test of time to recovery with remdesivir as compared with placebo, with stratification by disease severity."

2.7: N (High Certainty, 95%)

All randomized participants were included in the primary analysis.

Result of Domain 2: Low risk (High Certainty, 90%)

The study was appropriately blinded and used intention-to-treat analysis. Any deviations from intended interventions were likely balanced between groups.

Domain 3: Risk of bias due to missing outcome data

3.1: Y (High Certainty, 95%)

Data was available for nearly all randomized participants. Only 14 in the remdesivir group and 9 in the placebo group terminated participation early.

3.2: N (Moderate Certainty, 80%)

No evidence of methods to correct for bias due to missing data.

3.3: PN (Moderate Certainty, 80%)

Unlikely that missingness depended on true outcome, as mortality data should be available even for participants who discontinued.

3.4: PN (Moderate Certainty, 80%)

As above, unlikely that missingness depended on true outcome for mortality data.

Result of Domain 3: Low risk (High Certainty, 90%)

Very little missing data, and unlikely to depend on true outcome for mortality.

Domain 4: Risk of bias in measurement of the outcome

4.1: N (High Certainty, 95%)

All-cause mortality is an objective outcome that is unlikely to be measured inappropriately.

4.2: N (High Certainty, 95%)

Mortality should be measured the same way for both groups.

4.3: N (High Certainty, 95%)

The study was double-blinded, so outcome assessors were likely unaware of intervention received.

4.4: N (High Certainty, 95%)

All-cause mortality is an objective outcome that is unlikely to be influenced by knowledge of the intervention.

4.5: N (High Certainty, 95%)

As above, all-cause mortality is unlikely to be influenced by knowledge of the intervention.

Result of Domain 4: Low risk (High Certainty, 95%)

All-cause mortality is an objective outcome unlikely to be biased by measurement or assessment.

Domain 5: Risk of bias in selection of the reported result

5.1: Y (Moderate Certainty, 80%)

The study was registered (NCT04280705) and the primary outcome is reported as pre-specified.

5.2: N (High Certainty, 90%)

Mortality was a secondary outcome and appears to be reported as pre-specified.

5.3: N (High Certainty, 90%)

No evidence of selection of analysis method for mortality outcome.

Result of Domain 5: Low risk (High Certainty, 90%)

The study was pre-registered and outcomes appear to be reported as planned.

Overall Bias Risk for the Article: Low risk (High Certainty, 90%)

The study has a low risk of bias across all domains. It was appropriately randomized, blinded, and analyzed. The outcome of all-cause mortality is objective and unlikely to be biased. The study was pre-registered and reported results as planned.

## Study 4-1

Here is my assessment of the risk of bias for the study by Hu et al. (2020):

**Article ID**: Hu, 2020

**Outcome Name**: Functional ability: independence with activities of daily living at discharge from hospital

**registration number**: Not reported

Domain 1: Risk of bias arising from the randomization process

1.1: NI (50% confidence)

Reason: The study only mentions "randomized controlled trial" without describing the specific method of sequence generation.

1.2: NI (50% confidence)

Reason: The article does not mention any methods used to conceal the allocation sequence.

1.3: N (80% confidence)

Reason: The baseline characteristics were similar between groups, with no significant differences reported.

Result of Domain 1: Some concerns (75% confidence)

Reason: The randomization process was not clearly described, but baseline characteristics were balanced.

Domain 2: Risk of bias due to deviations from the intended interventions

2.1: Y (90% confidence)

Reason: Participants were aware of their assigned intervention as the intervention involved physical activities.

2.2: Y (90% confidence)

Reason: Carers and people delivering the interventions were aware of participants' assigned intervention as they had to assist or encourage participants.

2.3: NI (50% confidence)

Reason: The article does not report any deviations from the intended intervention.

2.4: NI (50% confidence)

Reason: No information is provided to judge whether deviations affected the outcome.

2.5: NI (50% confidence)

Reason: No information is provided to judge whether deviations were balanced between groups.

2.6: PY (70% confidence)

Reason: The number of participants analyzed is less than the number randomized, suggesting some exclusions, but no clear evidence of inappropriate analysis methods.

2.7: PN (70% confidence)

Reason: The number of excluded participants was small and similar across groups, likely not substantially impacting results.

Result of Domain 2: Some concerns (75% confidence)

Reason: Participants and personnel were aware of assignments, but impact of any deviations is unclear. Analysis appears appropriate.

Domain 3: Missing outcome data

3.1: N (90% confidence)

Reason: Less than 90% of randomized participants were included in the analysis (114 randomized, 39+38+37=114 analyzed).

3.2: N (80% confidence)

Reason: No evidence of methods to correct for bias from missing data is reported.

3.3: PY (70% confidence)

Reason: Reasons for missing data (e.g. unable to contact, refused) could plausibly be related to the outcome.

3.4: NI (50% confidence)

Reason: Insufficient information to determine if missingness in the outcome depended on its true value.

Result of Domain 3: High risk (80% confidence)

Reason: Substantial missing data with no correction, and missingness potentially related to the outcome.

Domain 4: Risk of bias in measurement of the outcome

4.1: N (80% confidence)

Reason: The Katz ADL score is an appropriate and validated measure for functional ability.

4.2: N (80% confidence)

Reason: The measurement methods and timing appear consistent across intervention groups.

4.3: Y (90% confidence)

Reason: The outcome is patient-reported and participants were aware of their intervention assignment.

4.4: Y (90% confidence)

Reason: The ADL score is a subjective measure reported by patients who were aware of their intervention.

4.5: N (70% confidence)

Reason: While patients' knowledge of intervention could influence their reporting, there is no clear evidence that it substantially affected outcome assessment.

Result of Domain 4: Some concerns (75% confidence)

Reason: While the outcome measure is appropriate, it is subjective and patients were aware of their intervention, creating potential for bias.

Domain 5: Risk of bias in selection of the reported result

5.1: NI (50% confidence)

Reason: The article does not mention a pre-specified analysis plan or trial registration.

5.2: N (80% confidence)

Reason: The reported results appear to correspond to all intended outcome measurements for ADL.

5.3: N (80% confidence)

Reason: The reported results appear to correspond to the intended analyses for ADL.

Result of Domain 5: Some concerns (75% confidence)

Reason: While reported results match expected measures and analyses, lack of a pre-specified plan creates some uncertainty.

Overall Bias Risk for the Article: High risk (80% confidence)

Reason: The study has some concerns in multiple domains and high risk of bias due to missing outcome data, which could significantly impact the reliability of the results for this outcome.

## Study 4-2

Here is my assessment based on the provided article and instructions:

**Article ID**: Hu, 2020

**Outcome Name**: Functional ability: independence with activities of daily living at discharge from hospital

**Registration number**: Not reported

Domain 1

1.1: NI (75% confidence)

Reason: The study mentions randomization but does not describe the specific method used to generate the allocation sequence.

1.2: NI (75% confidence)

Reason: The article does not mention any methods used to conceal the allocation sequence.

1.3: N (95% confidence)

Reason: The baseline characteristics were similar between groups with no significant differences reported.

Result of domain 1: Some concerns (90% confidence)

Reason: While randomization was used, key details about sequence generation and allocation concealment are not reported, raising some concerns about potential bias in the randomization process.

Domain 2

2.1: Y (95% confidence)

Reason: The intervention involved patients actively participating in exercises, so they were aware of their assigned intervention.

2.2: Y (95% confidence)

Reason: The researchers provided supervision and assistance to the intervention group, so they were aware of participants' assigned interventions.

2.3: N (90% confidence)

Reason: No deviations from intended interventions arising from the trial context are reported.

2.4: N (90% confidence)

Reason: As no deviations are reported, they are unlikely to have affected the outcome.

2.5: N (90% confidence)

Reason: As no deviations are reported, there are no differences between groups to assess.

2.6: PY (80% confidence)

Reason: The analysis appears to include all randomized participants, but the exact analysis method is not explicitly stated.

2.7: PN (80% confidence)

Reason: The number analyzed matches the number randomized, suggesting minimal exclusions that would substantially impact results.

Result of domain 2: Some concerns (85% confidence)

Reason: While the intervention was not blinded, there do not appear to be major deviations from intended interventions. However, the lack of clarity on the exact analysis method raises some concerns.

Domain 3

3.1: Y (95% confidence)

Reason: Data was available for 114 out of 150 randomized participants (76%), which exceeds the 95% threshold for continuous outcomes.

3.2: N (90% confidence)

Reason: No evidence is provided that results were not biased by missing outcome data.

3.3: PY (80% confidence)

Reason: Reasons for missing data are not fully reported, but may be related to patients' health status and thus potentially to the outcome.

3.4: NI (75% confidence)

Reason: Insufficient information is provided to determine if missingness depended on the true value of the outcome.

Result of domain 3: Some concerns (85% confidence)

Reason: While data was available for most participants, the potential relationship between missingness and the outcome raises some concerns about bias due to missing data.

Domain 4

4.1: N (90% confidence)

Reason: The Katz ADL score is an appropriate and validated measure for assessing functional ability in older adults.

4.2: N (90% confidence)

Reason: The outcome was measured consistently across groups using the same tool.

4.3: Y (95% confidence)

Reason: The outcome was patient-reported and patients were aware of their intervention assignment.

4.4: Y (95% confidence)

Reason: ADL is a subjective measure reported by patients who were aware of their intervention.

4.5: N (80% confidence)

Reason: While patients were aware of their intervention, there is no clear evidence that this knowledge influenced their outcome assessment.

Result of domain 4: Some concerns (85% confidence)

Reason: While the measurement method was appropriate, the subjective nature of the outcome and lack of blinding raises some concerns about potential bias in measurement.

Domain 5

5.1: NI (75% confidence)

Reason: The article does not mention pre-registration or a pre-specified analysis plan.

5.2: N (90% confidence)

Reason: The reported results appear to correspond to the planned measurements described in the methods.

5.3: N (90% confidence)

Reason: There is no evidence of multiple analyses or selective reporting based on results.

Result of domain 5: Some concerns (85% confidence)

Reason: While there is no clear evidence of selective reporting, the lack of a pre-specified analysis plan raises some concerns.

Overall Bias Risk for the Article: Some concerns (85% confidence)

Reason: While the study has several strengths, including appropriate randomization and outcome measurement, the lack of blinding, potential for bias due to missing data, and absence of a pre-specified analysis plan contribute to some concerns about potential bias across multiple domains. No single domain was assessed as high risk, but the cumulative effect of these concerns warrants caution in interpreting the results.

## Study 5-1

**Article ID**: Novotna, 2011

**Outcome Name**: Quality of Life

Domain 1

1.1 : NI (High Certainty - 95%)

The article states that randomization was performed but does not specify the method used to generate the allocation sequence.

1.2: NI (75% confidence)

The article does not mention central allocation or envelopes for allocation concealment.

1.3: N (90% confidence)

Baseline characteristics appear balanced between groups after randomization.

Result of Domain 1: Some concerns (80% confidence)

Lack of information on allocation concealment raises some concerns.

Domain 2

2.1: Y (90% confidence)

The study is described as double-blind, but participants were aware of their assigned intervention during Phase A.

2.2: Y (90% confidence)

Care providers were aware of participants' assignments during Phase A.

2.3: NI (75% confidence)

No information is provided about deviations from intended interventions.

2.4: NI (75% confidence)

Insufficient information to determine if deviations affected the outcome.

2.5: NI (75% confidence)

Insufficient information to determine if deviations were balanced between groups.

2.6: Y (90% confidence)

An intention-to-treat analysis was used for the primary endpoint.

2.7: N (85% confidence)

The ITT analysis likely minimized the impact of any missing data or protocol deviations.

Result of Domain 2: Some concerns (80% confidence)

While appropriate analyses were used, lack of information on deviations raises some concerns.

Domain 3

3.1: Y (90% confidence)

The study reports using ITT analysis for the primary endpoint.

3.2: Y (90% confidence)

The study used ITT analysis, which helps address potential bias from missing data.

3.3: PN (80% confidence)

The small amount of missing data is unlikely to depend on the true outcome value.

3.4: NI (75% confidence)

Insufficient information to determine if missingness depended on the true value.

Result of Domain 3: Low risk (85% confidence)

The use of ITT analysis and small amount of missing data suggest low risk of bias.

Domain 4

4.1: N (85% confidence)

Quality of life was measured using validated scales (EQ-5D and SF-36).

4.2: N (90% confidence)

The measurement methods appear consistent between groups.

4.3: Y (90% confidence)

The study is described as double-blind, but participants were aware of their assignment during Phase A.

4.4: Y (90% confidence)

Quality of life is a subjective outcome that could be influenced by knowledge of the intervention.

4.5: N (85% confidence)

There is no clear evidence that assessment of the outcome was influenced by knowledge of intervention received.

Result of Domain 4: Some concerns (80% confidence)

While validated measures were used, the subjective nature of the outcome raises some concerns.

Domain 5

5.1: Y (90% confidence)

The statistical analysis plan was pre-specified.

5.2: N (85% confidence)

Results appear to be reported for all expected quality of life measures.

5.3: N (85% confidence)

No evidence of selective reporting based on results.

Result of Domain 5: Low risk (85% confidence)

The analysis appears to follow a pre-specified plan without evidence of selective reporting.

Overall Bias Risk for the Article: Some concerns (80% confidence)

While many aspects of the study design are robust, the lack of information on allocation concealment and potential for unblinding during Phase A raise some concerns about bias.

## Study 5-2

**Article ID**: Novotna, 2011

**Outcome Name**: Quality of Life

Domain 1

1.1: NI (75% confidence)

The study is described as randomized, but specific methods for generating the random sequence are not reported.

1.2: NI (75% confidence)

The article does not mention central allocation or envelopes for allocation concealment.

1.3: N (90% confidence)

Baseline characteristics appear balanced between groups after randomization.

Result of Domain 1: Some concerns (80% confidence)

Lack of information on randomization method and allocation concealment raises some concerns.

Domain 2

2.1: Y (90% confidence)

Participants were aware of their assigned intervention during Phase A, which was single-blind.

2.2: Y (90% confidence)

Care providers were aware of participants' assignments during Phase A.

2.3: NI (75% confidence)

No information is provided about deviations from intended interventions.

2.4: NI (75% confidence)

Insufficient information to determine if deviations affected the outcome.

2.5: NI (75% confidence)

Insufficient information to determine if deviations were balanced between groups.

2.6: Y (90% confidence)

An intention-to-treat analysis was used for the primary endpoint.

2.7: N (85% confidence)

The ITT analysis likely minimized the impact of any missing data or protocol deviations.

Result of Domain 2: Some concerns (80% confidence)

While appropriate analyses were used, lack of information on deviations raises some concerns.

Domain 3

3.1: Y (90% confidence)

The study reports using ITT analysis for the primary endpoint, indicating all randomized participants were included in the analysis.

3.2: Y (90% confidence)

The study used ITT analysis, which helps address potential bias from missing data.

3.3: PN (80% confidence)

The small amount of missing data is unlikely to depend on the true outcome value.

3.4: NI (75% confidence)

Insufficient information to determine if missingness depended on the true value.

Result of Domain 3: Low risk (85% confidence)

The use of ITT analysis and small amount of missing data suggest low risk of bias.

Domain 4

4.1: N (85% confidence)

Quality of life was measured using validated scales (EQ-5D and SF-36).

4.2: N (90% confidence)

The measurement methods appear consistent between groups.

4.3: Y (90% confidence)

The study had a single-blind phase (Phase A) where participants were aware of their assignment.

4.4: Y (90% confidence)

Quality of life is a subjective outcome that could be influenced by knowledge of the intervention.

4.5: N (85% confidence)

There is no clear evidence that assessment of the outcome was influenced by knowledge of intervention received.

Result of Domain 4: Some concerns (80% confidence)

While validated measures were used, the subjective nature of the outcome and single-blind phase raise some concerns.

Domain 5

5.1: Y (90% confidence)

The statistical analysis plan was pre-specified.

5.2: N (85% confidence)

Results appear to be reported for all expected quality of life measures.

5.3: N (85% confidence)

No evidence of selective reporting based on results.

Result of Domain 5: Low risk (85% confidence)

The analysis appears to follow a pre-specified plan without evidence of selective reporting.

Overall Bias Risk for the Article: Some concerns (80% confidence)

While many aspects of the study design are robust, the lack of information on randomization method, allocation concealment, and potential for unblinding during Phase A raise some concerns about bias. However, the use of ITT analysis and validated outcome measures mitigate some of these concerns. I apologize for any inconsistencies in my previous responses. I'll reassess the study based on your instructions and the article content, ensuring strict adherence to the guidelines.

**Article ID**: Novotna, 2011

**Outcome Name**: Quality of Life

Domain 1

1.1: NI (75% confidence)

The study is described as randomized, but specific methods for generating the random sequence are not reported.

1.2: NI (75% confidence)

The article does not mention central allocation or envelopes for allocation concealment.

1.3: N (90% confidence)

Baseline characteristics appear balanced between groups after randomization.

Result of Domain 1: Some concerns (80% confidence)

Lack of information on randomization method and allocation concealment raises some concerns.

Domain 2

2.1: Y (90% confidence)

Participants were aware of their assigned intervention during Phase A, which was single-blind.

2.2: Y (90% confidence)

Care providers were aware of participants' assignments during Phase A.

2.3: NI (75% confidence)

No information is provided about deviations from intended interventions.

2.4: NI (75% confidence)

Insufficient information to determine if deviations affected the outcome.

2.5: NI (75% confidence)

Insufficient information to determine if deviations were balanced between groups.

2.6: Y (90% confidence)

An intention-to-treat analysis was used for the primary endpoint.

2.7: N (85% confidence)

The ITT analysis likely minimized the impact of any missing data or protocol deviations.

Result of Domain 2: Some concerns (80% confidence)

While appropriate analyses were used, lack of information on deviations raises some concerns.

Domain 3

3.1: Y (90% confidence)

The study reports using ITT analysis for the primary endpoint, indicating all randomized participants were included in the analysis.

3.2: Y (90% confidence)

The study used ITT analysis, which helps address potential bias from missing data.

3.3: PN (80% confidence)

The small amount of missing data is unlikely to depend on the true outcome value.

3.4: NI (75% confidence)

Insufficient information to determine if missingness depended on the true value.

Result of Domain 3: Low risk (85% confidence)

The use of ITT analysis and small amount of missing data suggest low risk of bias.

Domain 4

4.1: N (85% confidence)

Quality of life was measured using validated scales (EQ-5D and SF-36).

4.2: N (90% confidence)

The measurement methods appear consistent between groups.

4.3: Y (90% confidence)

The study had a single-blind phase (Phase A) where participants were aware of their assignment.

4.4: Y (90% confidence)

Quality of life is a subjective outcome that could be influenced by knowledge of the intervention.

4.5: N (85% confidence)

There is no clear evidence that assessment of the outcome was influenced by knowledge of intervention received.

Result of Domain 4: Some concerns (80% confidence)

While validated measures were used, the subjective nature of the outcome and single-blind phase raise some concerns.

Domain 5

5.1: Y (90% confidence)

The statistical analysis plan was pre-specified.

5.2: N (85% confidence)

Results appear to be reported for all expected quality of life measures.

5.3: N (85% confidence)

No evidence of selective reporting based on results.

Result of Domain 5: Low risk (85% confidence)

The analysis appears to follow a pre-specified plan without evidence of selective reporting.

Overall Bias Risk for the Article: Some concerns (80% confidence)

While many aspects of the study design are robust, the lack of information on randomization method, allocation concealment, and potential for unblinding during Phase A raise some concerns about bias. However, the use of ITT analysis and validated outcome measures mitigate some of these concerns.

## Study 6-1

Article ID: Bart, 2005

Outcome Name: Weight

Domain 1

1.1 : NI (High Certainty - 95%)

The article states that randomization was performed but does not specify the method used to generate the allocation sequence.

1.2 : NI (High Certainty - 95%)

The article does not mention any specific methods used to conceal the allocation sequence until participants were enrolled and assigned to interventions.

1.3 : N (High Certainty - 95%)

Table 1 shows the baseline characteristics were similar between the two groups, suggesting the randomization process was not compromised.

Some concerns (High Certainty - 95%)

The article does not provide information on the methods used for allocation sequence generation or concealment. However, the baseline characteristics were balanced, lowering concerns about the randomization process.

Domain 2

2.1 : Y (High Certainty - 100%)

The article states that this was an unblinded study, so the participants were aware of their assigned interventions.

2.2 : Y (High Certainty - 100%)

As an unblinded study, the personnel delivering the interventions were aware of the participants' assigned interventions.

2.3 : N (High Certainty - 95%)

The article does not report any deviations from the intended interventions that arose because of the trial context.

2.4 : N/A

2.5 : N/A

2.6 : Y (High Certainty - 100%)

The article states that all group comparisons were performed on an intention-to-treat basis, which is an appropriate way to estimate the effect of assignment to intervention.

2.7 : N (High Certainty - 100%)

An intention-to-treat analysis was used, so all randomized participants were included in the analysis in the group to which they were randomized.

Low risk (High Certainty - 100%)

Participants and personnel were unblinded, but no major deviations from intended interventions were reported. An appropriate intention-to-treat analysis was used.

Domain 3

3.1 : Y (High Certainty - 100%)

The article states that "All group comparisons were performed on an intention-to-treat basis", indicating that data was available for all or nearly all randomized participants.

Low risk (High Certainty - 100%)

The use of intention-to-treat analysis suggests outcome data was available for all randomized participants, resulting in a low risk of bias from missing outcome data.

Domain 4

4.1 : N (High Certainty - 100%)

Measuring weight is a well-established and reliable method for assessing fluid loss in heart failure patients.

4.2 : N (High Certainty - 95%)

The article states that weights were obtained by research personnel using the same scale with the patient shoeless in a hospital gown at baseline and 24 hours in both groups.

4.3 : Y (High Certainty - 100%)

As an unblinded study, the outcome assessors were aware of the participants' assigned interventions.

4.4 : N (High Certainty - 100%)

Weight is an objective outcome that would not be influenced by knowledge of the intervention received when assessed in a standardized manner as described.

4.5 : N (High Certainty - 100%)

There is no clear evidence that the weight assessment was influenced by knowledge of the intervention received. The use of a standardized scale and procedure across both groups minimizes this risk.

Some concerns (High Certainty - 95%)

While the outcome assessors were unblinded, the use of an objective outcome (weight) assessed in a standardized manner across groups lowers concerns about bias in the outcome assessment. However, the lack of blinding still raises some minor concerns.

Domain 5

5.1 : NI (High Certainty - 100%)

The article does not mention a pre-specified analysis plan or whether one was finalized before unblinded outcome data were available for analysis.

5.2 : N (High Certainty - 100%)

The article describes assessing weight loss at the pre-specified timepoints of 24 hours (primary) and 48 hours (secondary). There is no indication that the reported outcome was selected from among multiple measurements.

5.3 : N (High Certainty - 100%)

The article reports the pre-specified primary outcome analysis of weight change at 24 hours. There is no indication that this result was selected from multiple analyses of the weight data.

Some concerns (High Certainty - 100%)

No information is provided about a pre-specified analysis plan to judge if the reported outcome was selectively reported from among different outcome measurements or analyses. However, the outcome was assessed and reported as pre-specified in the methods.

Overall Bias Risk for the Article

Some concerns (High Certainty - 95%)

Three domains (randomization, missing data, reporting) were rated as "some concerns" due to lack of information on allocation concealment, adherence to pre-specified analysis plans, and minor issues with outcome assessment. However, no domains were rated as high risk of bias.

## Study 6-2

**Article ID**: Bart, 2005

**Outcome Name**: Weight loss

Domain 1

1.1 : NI (High Certainty - 100%)

The article mentions randomization but does not provide specific details on the method used to generate the allocation sequence.

1.2 : NI (High Certainty - 100%)

The article does not provide any information on allocation concealment.

1.3 : N (High Certainty - 100%)

The article states the baseline characteristics of the two groups in Table 1, which shows no major imbalances between the groups that would suggest a problem with the randomization process.

Some concerns (High Certainty - 100%)

The article does not provide information on the allocation sequence generation or concealment. However, the balanced baseline characteristics suggest the randomization process was likely adequate. With no information on allocation concealment, the risk of bias is assessed as some concerns.

Domain 2

2.1 : Y (High Certainty - 100%)

The ultrafiltration intervention would be apparent to participants, so they were likely aware of their assigned group.

2.2 : Y (High Certainty - 100%)

The personnel delivering the ultrafiltration intervention would know which participants were assigned to receive it.

2.3 : NI (High Certainty - 100%)

The article does not report whether there were deviations from the intended interventions that arose because of the trial context.

2.4 : NI (High Certainty - 100%)

There is no information provided to determine if any deviations from intended intervention were likely to have affected the outcome.

2.5 : NI (High Certainty - 100%)

No information is provided on whether any deviations from intended intervention were balanced between groups.

2.6 : Y (High Certainty - 100%)

The article states an intention-to-treat analysis was used, which is an appropriate method to estimate the effect of assignment to intervention.

2.7 : PN (Moderate Certainty - 80%)

With a small number of participants not included in the final analysis and the use of intention-to-treat analysis, there is unlikely to be substantial potential for impact from failing to analyze participants in their assigned group. However, this cannot be ruled out with certainty.

Some concerns (Moderate Certainty - 80%)

Participants and personnel were aware of intervention assignments, but it is unclear if there were deviations that could have affected the outcome. An appropriate intention-to-treat analysis was used. Overall, there are some concerns about risk of bias due to deviations from intended interventions.

Domain 3

3.1 : PY (Moderate Certainty - 75%)

The article states "All group comparisons were performed on an intention-to-treat basis", suggesting data were likely available for nearly all randomized participants. However, Figure 3 showing weight loss appears to have a small amount of missing data, so there is some uncertainty.

3.2 : N (High Certainty - 100%)

The article does not mention any methods used to account for missing data, such as sensitivity analyses, that would provide evidence the result was not biased by missing data.

3.3 : PN (Low Certainty - 60%)

Based on the information provided, it seems somewhat unlikely that missingness in the weight outcome would depend on its true value. However, this cannot be ruled out with certainty.

3.4 : NI (High Certainty - 100%)

There is no information provided to assess whether missingness in the weight outcome was likely to depend on its true value.

Some concerns (Moderate Certainty - 80%)

The intention-to-treat analysis suggests data were available for most participants, but Figure 3 indicates a small amount of missing weight data. No methods to account for this are described. While missingness seems somewhat unlikely to depend on the true weight values, this is not certain. Overall, there are some concerns about risk of bias from missing outcome data.

Domain 4

4.1 : N (High Certainty - 100%)

Measuring weight is a well-established and reliable outcome measure.

4.2 : N (High Certainty - 100%)

The article states "Weights were obtained by research personnel at baseline and 24 h using the same scale with the patient shoeless in a hospital gown", indicating the measurement of weight was consistent across groups.

4.3 : NI (High Certainty - 100%)

No information is provided on whether research personnel measuring weight were blinded to intervention assignments.

4.4 : PN (Low Certainty - 60%)

Weight is an objective measure, but without information on blinding of outcome assessors, it cannot be ruled out with certainty that knowledge of the intervention could have influenced the assessment.

4.5 : PN (Low Certainty - 60%)

Without information on blinding of outcome assessors, the possibility that knowledge of the intervention influenced the weight assessment cannot be ruled out, although this seems somewhat unlikely for an objective measure.

Some concerns (Moderate Certainty - 75%)

Weight was measured consistently across groups. As an objective measure, risk of bias is likely low. However, lack of information on blinding of outcome assessors raises some concerns about potential for knowledge of intervention to influence the assessment.

Domain 5

5.1 : NI (High Certainty - 100%)

No information is provided on whether a pre-specified analysis plan was used.

5.2 : N (High Certainty - 100%)

The article provides details on the pre-specified weight loss outcome measurement, stating "The primary end point was weight loss 24 h after obtaining consent." There is no indication of selection from multiple outcome measurements.

5.3 : NI (High Certainty - 100%)

There is no information provided on whether the weight outcome was selected from multiple potential analyses of the data.

Some concerns (High Certainty - 100%)

The pre-specified nature of the weight loss outcome measurement suggests low risk of bias in selection of the reported result. However, without information on a pre-specified analysis plan and potential selection of the reported result from multiple analyses, there are some residual concerns in this domain.

Overall Bias Risk for the Article

High risk (High Certainty - 100%)

There are some concerns related to randomization, deviations from intended interventions, and missing outcome data due to lack of information on allocation concealment, blinding, and a small amount of missing data. There are also some concerns about potential for selection of the reported weight result from multiple analyses. Taken together, the uncertainties across multiple domains suggest an overall high risk of bias for the weight loss outcome in this trial.

## Study 7-1

Here is my assessment of the risk of bias for the Pinto-Fraga et al. 2015 study:

**Article ID**: Pinto-Fraga, 2016

**Outcome Name**: Patient-reported symptom scores—Qazi: LE alone

**registration number**: NCT02051023

Domain 1

1.1: Y (90% confidence)

The study reports using "blocked randomization of size 2" to generate the random allocation sequence.

1.2: Y (95% confidence)

The study used "sealed opaque envelopes" identified by treatment codes for allocation concealment.

1.3: N (90% confidence)

There were no significant differences in baseline characteristics between groups reported.

Result of Domain 1: Low risk (90% confidence)

Randomization and allocation concealment procedures appear adequate, with no baseline imbalances reported.

Domain 2

2.1: N (90% confidence)

The study is described as "double-masked" indicating participants were unaware of their assigned intervention.

2.2: N (90% confidence)

As a double-masked study, those delivering interventions were also likely unaware of assignments.

2.3: NI (80% confidence)

The article does not report whether there were deviations from intended interventions due to the trial context.

2.4: NI (80% confidence)

With no reported deviations, their impact on outcomes cannot be determined.

2.5: NI (80% confidence)

Without reported deviations, balance between groups cannot be assessed.

2.6: Y (90% confidence)

The analysis appears to include all randomized participants (41 randomized, 40 analyzed).

2.7: N (90% confidence)

With minimal exclusions from analysis, impact on results is likely minimal.

Result of Domain 2: Low risk (85% confidence)

Masking procedures appear adequate, with no clear evidence of deviations or issues with analysis.

Domain 3

3.1: Y (95% confidence)

Data appears to be available for nearly all randomized participants (40/41).

3.2: N (90% confidence)

No evidence is provided of methods to correct for potential bias from missing data.

3.3: PN (80% confidence)

With minimal missing data, it seems unlikely missingness depends on true values.

3.4: PN (80% confidence)

With minimal missing data, it seems unlikely missingness depends strongly on true values.

Result of Domain 3: Low risk (85% confidence)

Very little missing data, with low risk of bias from missingness.

Domain 4

4.1: N (90% confidence)

Standard methods for measuring dry eye outcomes were used.

4.2: N (95% confidence)

Measurement methods appear consistent between groups.

4.3: N (90% confidence)

The study is described as double-masked, indicating outcome assessors were likely unaware of intervention received.

4.4: Y (85% confidence)

Patient-reported symptom scores are subjective and could potentially be influenced by knowledge of intervention.

4.5: N (80% confidence)

There is no clear evidence that assessment was actually influenced by knowledge of intervention.

Result of Domain 4: Low risk (85% confidence)

Outcome measurement appears appropriate and unbiased, though some potential for bias in subjective measures.

Domain 5

5.1: Y (90% confidence)

The study was registered (NCT02051023) indicating a pre-specified analysis plan.

5.2: N (85% confidence)

The reported results appear to match the expected outcomes for this type of study.

5.3: N (85% confidence)

The analyses reported appear standard for this type of study.

Result of Domain 5: Low risk (85% confidence)

No clear evidence of selective reporting of results.

Overall Bias Risk for the Article: Low risk (85% confidence)

All domains were assessed as low risk, with no major concerns identified in the study's methods or reporting.

## Study 7-2

Here is my assessment of the risk of bias for the Pinto-Fraga 2016 study:

**Article ID**: Pinto-Fraga, 2016

**Outcome Name**: Patient‐reported symptom scores—Qazi: LE alone

**registration number**: NCT02051023

Domain 1

1.1: Y (High Certainty, 95%)

The study states "Patients randomly received 1 drop 4 times daily of either topical 0.1% fluorometholone (FML group) or topical polyvinyl alcohol (PA group) for 22 days."

1.2: Y (High Certainty, 95%)

The study mentions "The original labels of these bottles were removed and bottles were relabeled with the study number and a coded lot number to avoid potential identification of the treatment by site personnel or patients."

1.3: N (High Certainty, 95%)

The study reports "There were no significant differences in age or gender distribution between the 2 groups (P ¼ 0.37 and P ¼ 0.66, respectively). For baseline clinical characteristics, there were no statistical differences (P0.06) between groups in corneal and conjunctival staining, hyperemia, TBUT, unanesthetized Schirmer test results, BCVA, tear osmolarity, or IOP (Table 4)."

Result of Domain 1: Low risk (High Certainty, 95%)

The randomization process appears to have been adequately implemented with no baseline imbalances between groups.

Domain 2

2.1: N (High Certainty, 95%)

The study states it was "double-masked".

2.2: N (High Certainty, 95%)

The study states it was "double-masked".

2.3: NI (High Certainty, 95%)

The article does not provide information on deviations from the intended intervention.

2.4: NI (High Certainty, 95%)

There is no information provided to assess this.

2.5: NI (High Certainty, 95%)

There is no information provided to assess this.

2.6: Y (Moderate Certainty, 80%)

The study states "All patients were instructed to use the treatment with a dose regimen of 1 drop, 4 times daily in both eyes during the entire 22-day duration of the study." This suggests an intention-to-treat analysis.

2.7: PN (Moderate Certainty, 80%)

There is no indication of substantial exclusions or issues with the analysis.

Result of Domain 2: Low risk (Moderate Certainty, 80%)

While some details are unclear, the double-masking and apparent intention-to-treat analysis suggest low risk of bias.

Domain 3

3.1: Y (High Certainty, 95%)

The study included 41 patients, with 21 in the FML group and 19 in the PA group. This suggests complete or nearly complete outcome data.

3.2: N (High Certainty, 95%)

There is no evidence of methods used to correct for bias due to missing outcome data.

3.3: PN (Moderate Certainty, 80%)

There is no indication that missingness depends on the true value.

3.4: PN (Moderate Certainty, 80%)

There is no evidence suggesting missingness depends on the true value.

Result of Domain 3: Low risk (High Certainty, 90%)

There appears to be complete or nearly complete outcome data with no evidence of bias due to missing data.

Domain 4

4.1: N (High Certainty, 95%)

The SANDE questionnaire is an appropriate method for measuring patient-reported symptoms.

4.2: N (High Certainty, 95%)

The measurement method was the same for both groups.

4.3: N (High Certainty, 95%)

The study was described as double-masked.

4.4: Y (High Certainty, 95%)

The outcome is subjective and patient-reported.

4.5: N (Moderate Certainty, 80%)

While the outcome is subjective, the double-masking should mitigate influence on assessment.

Result of Domain 4: Low risk (High Certainty, 90%)

The double-masked design and use of a standardized questionnaire suggest low risk of bias in outcome measurement.

Domain 5

5.1: Y (High Certainty, 95%)

The study was registered (NCT02051023) prior to beginning.

5.2: N (High Certainty, 95%)

There is no evidence of selection of results based on multiple outcome measurements.

5.3: N (High Certainty, 95%)

There is no evidence of selection of results based on multiple analyses.

Result of Domain 5: Low risk (High Certainty, 95%)

Pre-registration and no evidence of selective reporting suggest low risk of bias.

Overall Bias Risk for the Article: Low risk (High Certainty, 90%)

All domains were assessed as low risk, with generally high certainty. The double-masked design, pre-registration, and complete outcome data are key strengths reducing risk of bias.

## Study 8-1

Here is my assessment of the risk of bias for the specified article:

**Article ID**: Dissanayake, 2019

**Outcome Name**: Atopic dermatitis by 1-3 years

**Registration number**: JPRN-UMIN000010838

Domain 1

1.1: Y (90% confidence)

The study used computerized random numbers for randomization.

1.2: Y (95% confidence)

Randomization was performed at a central location using a computerized system.

1.3: N (85% confidence)

No significant baseline differences were reported between groups.

Result of Domain 1: Low risk (90% confidence)

Allocation was adequately concealed and there were no baseline imbalances.

Domain 2

2.1: Y (95% confidence)

This was an open label trial, so participants were aware of their allocation.

2.2: Y (95% confidence)

As an open label trial, intervention providers were aware of allocations.

2.3: NI (80% confidence)

No information provided on deviations from intended interventions.

2.4: NI (80% confidence)

Insufficient information to determine if deviations affected outcomes.

2.5: NI (80% confidence)

No information on whether deviations were balanced between groups.

2.6: PN (85% confidence)

Number analyzed matches number randomized, suggesting all participants were included in analysis.

2.7: PN (85% confidence)

Analysis appears to have included all randomized participants.

Result of Domain 2: Some concerns (85% confidence)

Participants and providers were aware of allocations, but impact of any deviations is unclear.

Domain 3

3.1: Y (95% confidence)

459/549 (83.6%) of randomized participants were included in the 1 year analysis.

3.2: N (90% confidence)

No evidence of methods to correct for bias from missing data.

3.3: NI (80% confidence)

Reasons for missing data not provided.

3.4: NI (80% confidence)

Insufficient information to determine if missingness likely depended on true value.

Result of Domain 3: Some concerns (85% confidence)

Moderate amount of missing data without clear evidence it was addressed appropriately.

Domain 4

4.1: N (90% confidence)

Outcomes were measured using standard definitions and criteria for atopic dermatitis.

4.2: N (90% confidence)

Outcome measurement methods appear consistent across groups.

4.3: Y (95% confidence)

This was an open label trial, so outcome assessors were aware of allocations.

4.4: Y (90% confidence)

Diagnosis of atopic dermatitis can be influenced by knowledge of interventions.

4.5: N (85% confidence)

No clear evidence that assessment was influenced by knowledge of interventions.

Result of Domain 4: Some concerns (85% confidence)

Outcome assessors were aware of interventions, but unclear if this influenced assessment.

Domain 5

5.1: Y (90% confidence)

Analysis appears to follow the prespecified plan in the registered protocol.

5.2: N (90% confidence)

All prespecified outcomes appear to be reported.

5.3: N (90% confidence)

No evidence of selecting results from multiple analyses.

Result of Domain 5: Low risk (90% confidence)

No evidence of selective reporting or analysis.

Overall Bias Risk for the Article: Some concerns (85% confidence)

Multiple domains had some concerns, primarily related to lack of blinding and potential for missing data to influence results.

## Study 8-2

Here is my assessment of the risk of bias for the provided study:

**Article ID**: Dissanayake, 2019

**Outcome Name**: Atopic dermatitis by 1-3 years

**Registration number**: JPRN-UMIN000010838

Domain 1

1.1: NI (80% confidence)

The article only mentions randomization without specifying the method for generating the random sequence.

1.2: Y (90% confidence)

The article states "Randomization was performed at a central location."

1.3: N (85% confidence)

Table 1 shows baseline characteristics were similar between groups.

Result of Domain 1: Low risk (80% confidence)

Allocation was concealed, and baseline characteristics were balanced, despite lack of details on sequence generation.

Domain 2

2.1: Y (95% confidence)

The interventions (emollient application, synbiotics) could not be blinded to participants.

2.2: Y (95% confidence)

The interventions could not be blinded to people delivering them.

2.3: N (90% confidence)

No deviations from intended interventions due to trial context are reported.

2.4: N (90% confidence)

Not applicable as no deviations reported.

2.5: N (90% confidence)

Not applicable as no deviations reported.

2.6: Y (85% confidence)

The article states "All analyses were performed using SAS® version 9.3" suggesting appropriate analysis.

2.7: N (85% confidence)

No evidence of substantial impact from failure to analyze by randomized group.

Result of Domain 2: Some concerns (80% confidence)

Participants and personnel were not blinded, but this is unlikely to have substantially impacted results.

Domain 3

3.1: Y (95% confidence)

459 out of 549 randomized participants (83.6%) had outcome data at 1 year.

3.2: N (90% confidence)

No evidence of methods to correct for bias from missing data.

3.3: N (85% confidence)

No clear reason why missingness would depend on true outcome.

3.4: N (85% confidence)

No evidence that missingness depended on true outcome value.

Result of Domain 3: Low risk (85% confidence)

Over 80% of participants had outcome data, with no clear evidence of bias from missing data.

Domain 4

4.1: N (90% confidence)

AD diagnosis based on standardized criteria.

4.2: N (90% confidence)

No evidence of differential measurement between groups.

4.3: Y (95% confidence)

Parents aware of intervention status would have reported AD symptoms.

4.4: Y (90% confidence)

AD diagnosis partly based on parent-reported symptoms.

4.5: N (85% confidence)

No clear evidence that knowledge of intervention influenced outcome assessment.

Result of Domain 4: Some concerns (80% confidence)

Parents aware of intervention reported AD symptoms, but used standardized criteria.

Domain 5

5.1: Y (90% confidence)

Analysis plan pre-specified in registered protocol.

5.2: N (85% confidence)

All pre-specified outcomes appear to be reported.

5.3: N (85% confidence)

No evidence of selecting results from multiple analyses.

Result of Domain 5: Low risk (85% confidence)

Analysis appears to follow pre-specified plan with no evidence of selective reporting.

Overall Bias Risk: Some concerns (80% confidence)

Main concerns are lack of blinding and potential for bias in parent-reported symptoms, but otherwise well-conducted trial.

## Study 9-1

**Article ID**: Frattarelli, 2003

**Outcome Name**: Multiple pregnancy rate

Domain 1: Risk of bias arising from the randomization process

1.1: Y (90% confidence)

Reason: The article clearly states that randomization was done using a computer-generated randomization table, which is an acceptable method of sequence generation.

1.2: PY (80% confidence)

Reason: The article states "The sequences of randomization were concealed until intervention was assigned", suggesting allocation concealment, but does not provide specific details on the method used.

1.3: N (90% confidence)

Reason: The baseline characteristics reported in Table 1 do not show any significant differences between the day 3 and day 5 groups.

Result of Domain 1: Low risk (85% confidence)

Reason: Randomization was properly implemented, allocation was likely concealed, and baseline characteristics are balanced.

Domain 2: Risk of bias due to deviations from intended interventions

2.1: Y (90% confidence)

Reason: Given the nature of the intervention (day 3 vs day 5 embryo transfer), participants would be aware of their assigned group.

2.2: Y (90% confidence)

Reason: Given the nature of the intervention, care providers would be aware of the assigned group when performing the embryo transfer.

2.3: NI (80% confidence)

Reason: The article does not provide specific information about deviations from the intended interventions.

2.6: NI (80% confidence)

Reason: The article does not explicitly state the analysis method used, such as intention-to-treat analysis.

2.7: NI (80% confidence)

Reason: Without clear information on the analysis method, it's difficult to assess the potential impact on results.

Result of Domain 2: Some concerns (85% confidence)

Reason: While participants and providers were aware of the intervention, this is inherent to the type of intervention. However, lack of information about potential deviations and analysis method raises some concerns.

Domain 3: Missing outcome data

3.1: Y (95% confidence)

Reason: All 49 randomized participants appear to be included in the analysis.

Result of Domain 3: Low risk (95% confidence)

Reason: There does not appear to be any missing outcome data.

Domain 4: Risk of bias in measurement of the outcome

4.1: N (90% confidence)

Reason: Multiple pregnancy rate is an objective outcome that can be reliably measured.

4.2: N (90% confidence)

Reason: The method of measuring multiple pregnancy rate would be the same for both groups.

4.3: Y (90% confidence)

Reason: Given the nature of the intervention, outcome assessors would be aware of the intervention received.

4.4: N (90% confidence)

Reason: Multiple pregnancy rate is an objective outcome that is unlikely to be influenced by knowledge of the intervention.

4.5: N (90% confidence)

Reason: There is no clear evidence that assessment of multiple pregnancy rate was influenced by knowledge of the intervention.

Result of Domain 4: Low risk (90% confidence)

Reason: While outcome assessors were likely aware of the intervention, the outcome is objective and unlikely to be influenced by this knowledge.

Domain 5: Risk of bias in selection of the reported result

5.1: NI (70% confidence)

Reason: The article does not mention a pre-specified analysis plan or trial registration.

5.2: N (80% confidence)

Reason: The reported results for multiple pregnancy rate appear to be consistent with the methods described.

5.3: N (80% confidence)

Reason: There is no evidence of selection of results based on multiple analyses.

Result of Domain 5: Some concerns (75% confidence)

Reason: While there's no clear evidence of selective reporting, the lack of a pre-specified analysis plan raises some concerns.

Overall Bias Risk for the Article: Some concerns (85% confidence)

## Study 9-2

Thank you for the detailed instructions. I will carefully follow these guidelines when evaluating the risk of bias for this randomized controlled trial. I'll make sure to check for specific randomization and allocation concealment methods, avoid assumptions, and thoroughly search the article before answering any question as "NI". Let me proceed with the evaluation:

Domain 1: Risk of bias arising from the randomization process

1.1: Y (90% confidence)

Reason: The article explicitly states that randomization was done using a computer-generated randomization table, which is an adequate method for generating a random sequence.

1.2: PY (80% confidence)

Reason: The article states "The sequences of randomization were concealed until intervention was assigned", suggesting allocation concealment, but does not provide details on the specific method used.

1.3: N (90% confidence)

Reason: The baseline characteristics between groups appear similar based on the data provided in Table 1, with no significant differences noted.

Result of domain 1: Low risk (85% confidence)

Reason: Randomization was adequately generated and allocation appears to have been concealed, with no evidence of baseline imbalances between groups.

Domain 2: Risk of bias due to deviations from intended interventions

2.1: PY (75% confidence)

Reason: The nature of the intervention (day 3 vs day 5 embryo transfer) makes it likely that participants were aware of their assigned group.

2.2: Y (90% confidence)

Reason: The clinicians performing the embryo transfers would necessarily be aware of which day the transfer was occurring.

2.3: NI (80% confidence)

Reason: The article does not provide specific information about deviations from the intended interventions.

2.4: NI (75% confidence)

Reason: Due to lack of information about deviations, it's unclear if they would have affected the outcome.

2.5: NI (75% confidence)

Reason: Without information on deviations, it's not possible to determine if they were balanced between groups.

2.6: NI (80% confidence)

Reason: The article does not explicitly state the analysis method used, such as intention-to-treat analysis.

2.7: NI (75% confidence)

Reason: Without clear information on the analysis method, it's not possible to determine the potential impact of any failure to analyze participants in the groups to which they were randomized.

Result of domain 2: Some concerns (80% confidence)

Reason: While participants and personnel were likely aware of intervention assignments, there is insufficient information about potential deviations from intended interventions and the analysis method used.

Domain 3: Missing outcome data

3.1: Y (95% confidence)

Reason: Outcome data appears to be available for all or nearly all randomized participants (49/57 = 86%).

3.2: N/A

3.3: N/A

3.4: N/A

Result of domain 3: Low risk (90% confidence)

Reason: There appears to be minimal missing outcome data, with similar numbers between groups.

Domain 4: Risk of bias in measurement of the outcome

4.1: N (90% confidence)

Reason: Multiple pregnancy rate is an objective outcome that can be reliably measured through ultrasound.

4.2: N (85% confidence)

Reason: The measurement of multiple pregnancy rate would likely be consistent between groups.

4.3: Y (90% confidence)

Reason: Given the nature of the intervention, the clinicians assessing pregnancy outcomes were likely aware of group assignments.

4.4: N (85% confidence)

Reason: Multiple pregnancy rate is an objective outcome that is unlikely to be influenced by knowledge of the intervention received.

4.5: N (80% confidence)

Reason: There is no clear evidence that assessment of multiple pregnancy rate was influenced by knowledge of the intervention received.

Result of domain 4: Low risk (85% confidence)

Reason: While outcome assessors were likely aware of interventions, the objective nature of the outcome makes bias in its measurement unlikely.

Domain 5: Risk of bias in selection of the reported result

5.1: NI (70% confidence)

Reason: The article does not mention pre-registration or a pre-specified analysis plan.

5.2: N (80% confidence)

Reason: The reported results for multiple pregnancy rate appear to correspond to a single, pre-specified analysis.

5.3: N (80% confidence)

Reason: There is no evidence of selective reporting based on the results.

Result of domain 5: Some concerns (75% confidence)

Reason: While there's no clear evidence of selective reporting, the lack of a pre-specified analysis plan raises some concerns.

Overall Bias Risk for the Article: Some concerns (80% confidence)

## Study 10-1

Article ID: Griffiths, 2019

Outcome Name: Self-harm

Registration number: NCT02771691

Domain 1: Risk of bias arising from the randomization process

1.1: Y (90% confidence)

Reason: The article states "Randomisation (at the individual level) was independent and concealed, using randomised-permuted blocks". Randomized-permuted blocks is a recognized method for generating a random sequence.

1.2: Y (90% confidence)

Reason: The article clearly states that randomization was "independent and concealed". This indicates that proper allocation concealment was used.

1.3: N (80% confidence)

Reason: The article reports no significant differences between groups at baseline, except for BPD scores which were higher in the treatment group.

Result: Low risk (85% confidence)

Reason: The study used appropriate methods for both random sequence generation and allocation concealment. The difference in BPD scores at baseline is not enough to suggest a problem with the randomization process.

Domain 2: Risk of bias due to deviations from intended interventions

2.1: Y (90% confidence)

Reason: Participants were aware of their group assignment as blinding was not possible for this intervention.

2.2: Y (90% confidence)

Reason: Treatment providers were aware of participants' group assignments.

2.3: NI (70% confidence)

Reason: The article does not report if there were deviations from the intended intervention due to the trial context.

2.4: NI (70% confidence)

Reason: Not enough information is provided to determine if any deviations likely affected the outcome.

2.5: NI (70% confidence)

Reason: No information is provided about whether any deviations were balanced between groups.

2.6: Y (80% confidence)

Reason: The article states that intent-to-treat analysis was used.

2.7: N (80% confidence)

Reason: Intent-to-treat analysis was used, so participants were analyzed in their assigned groups.

Result: Some concerns (75% confidence)

Reason: While intent-to-treat analysis was used, the lack of blinding and limited information about protocol deviations raises some concerns.

Domain 3: Missing outcome data

3.1: Y (95% confidence)

Complete data for hospital records outcome at 36 weeks.

3.2: Y (80% confidence)

Multiple imputation used for missing data.

3.3: N (80% confidence)

Complete data for hospital records, missingness unlikely to depend on true value.

3.4: N (80% confidence)

Not applicable due to complete data for hospital records.

Result of the domain: Low risk (85% confidence) Complete data for key outcome, appropriate handling of missing data for others.

Domain 4: Risk of bias in measurement of the outcome

4.1: N (80% confidence)

Reason: Self-report and hospital record measures of self-harm appear appropriate.

4.2: N (80% confidence)

Reason: The outcome measures were applied equally to both groups.

4.3: Y (90% confidence)

Reason: For self-reported outcomes, participants were aware of their intervention status.

4.4: Y (90% confidence)

Reason: Self-reported self-harm is a subjective measure that could be influenced by knowledge of intervention received.

4.5: N (70% confidence)

Reason: There is no clear evidence that assessment was influenced by knowledge of intervention received.

Result: Some concerns (75% confidence)

Reason: While measures were appropriate, the subjective nature of self-reported outcomes raises some concerns.

Domain 5: Risk of bias in selection of the reported result

5.1: Y (80% confidence)

Reason: The analyses appear to follow the pre-specified plan outlined in the methods.

5.2: N (80% confidence)

Reason: All pre-specified outcomes appear to be reported.

5.3: N (80% confidence)

Reason: There is no evidence of selective reporting based on the results.

Result: Low risk (80% confidence)

Reason: The reported results appear to follow the pre-specified analysis plan without evidence of selective reporting.

Overall Bias Risk for the Article: High risk (80% confidence)

Reason: The high risk of bias due to missing outcome data is the primary driver of the overall high risk rating. While other domains have some concerns or low risk, the substantial loss to follow-up is a major limitation of this study.

## Study 10-2

I understand. I will carefully evaluate the article based on the provided instructions, focusing on the outcome of self-harm. I'll assess each domain and question according to the guidelines, providing my reasoning and confidence level for each assessment. I'll be sure to double-check key points like randomization methods, allocation concealment, analysis methods, and outcome assessment. I'll also search thoroughly for relevant information before answering "NI" to any question. Let me begin the evaluation:

**Article ID**: Griffiths, 2019

**Outcome Name**: Self-harm

**registration number**: NCT02771691

Domain 1: Risk of bias arising from the randomization process

1.1: Y (90% confidence)

The article states "Randomisation (at the individual level) was independent and concealed, using randomised-permuted blocks adjusted to permit access to group treatment without undue delay."

1.2: Y (90% confidence)

The article states "Group allocation remained concealed until completion of self-report ratings."

1.3: Y (80% confidence)

There were significant baseline differences between groups on the BPD total score.

Result: Some concerns (85% confidence)

While randomization and allocation concealment were reported, there were baseline imbalances between groups.

Domain 2: Risk of bias due to deviations from intended interventions

2.1: PY (80% confidence)

The nature of the intervention (group therapy) makes it likely participants were aware of their allocation.

2.2: PY (80% confidence)

The therapists delivering the intervention were likely aware of participants' allocation.

2.3: NI (70% confidence)

The article does not report on deviations from the intended intervention.

2.4: NI (70% confidence)

Not enough information to determine if deviations affected the outcome.

2.5: NI (70% confidence)

Not enough information to determine if deviations were balanced between groups.

2.6: Y (90% confidence)

The article explicitly reports using intent-to-treat analyses.

2.7: PN (70% confidence)

The use of intent-to-treat analysis suggests minimal impact of analyzing participants in the wrong group.

Result: Some concerns (75% confidence)

While ITT analysis was used, there is limited information on intervention adherence and deviations.

Domain 3: Missing outcome data

3.1: N (90% confidence)

Only 50% of participants completed post-treatment assessments and 42% completed follow-up.

3.2: N (80% confidence)

No evidence that results were not biased by missing data.

3.3: PY (70% confidence)

Participants may have dropped out due to worsening self-harm.

3.4: NI (70% confidence)

Not enough information to determine if missingness depended on its true value.

Result: High risk (80% confidence)

There was substantial missing data that likely depended on the true outcome values.

Domain 4: Risk of bias in measurement of the outcome

4.1: N (90% confidence)

Self-report and hospital records are appropriate measures of self-harm.

4.2: N (90% confidence)

The outcome was measured consistently across groups.

4.3: N (80% confidence)

Self-reported outcomes were completed before group allocation was revealed.

4.4: Y (90% confidence)

Self-reported self-harm is a subjective measure.

4.5: N (70% confidence)

No clear evidence that assessment was influenced by knowledge of intervention received.

Result: Low risk (80% confidence)

The outcome measures were appropriate and assessed consistently between groups.

Domain 5: Risk of bias in selection of the reported result

5.1: Y (90% confidence)

The article mentions a pre-specified analysis plan.

5.2: N (70% confidence)

All expected outcomes appear to be reported.

5.3: N (70% confidence)

There is no evidence of multiple analyses of the data.

Result: Low risk (80% confidence)

A pre-specified analysis plan was mentioned, and all expected outcomes were reported without evidence of selective reporting.

Overall Bias Risk for the Article: High risk (80% confidence)

The high risk rating is primarily due to the substantial missing outcome data. There are also some concerns about baseline imbalances between groups. However, the study demonstrates strengths in its randomization process, outcome measurement, and adherence to a pre-specified analysis plan.

## Study 11-1

Based on the information provided in the article, here is my assessment of the risk of bias:

**Article ID**: Horby, 2021

**Outcome Name**: All-cause mortality within 28 days after randomization

**Registration number**: NCT04381936

Domain 1: Risk of bias arising from the randomization process

1.1: Y (High Certainty - 95%)

Reason: The article states "Randomization was performed with the use of a Web-based system with concealment of the trial-group assignment."

1.2: Y (High Certainty - 95%)

Reason: The article mentions "Randomization was performed with the use of a Web-based system with concealment of the trial-group assignment."

1.3: N (High Certainty - 95%)

Reason: The article states "There was a significant (P = 0.01) difference in the mean age between patients in the dexamethasone group and those in the usual care group, but there were no significant differences between the groups in any other baseline characteristic."

Result of Domain 1: Low risk (High Certainty - 95%)

Reason: Allocation sequence was adequately generated and concealed, and baseline differences do not suggest a problem with the randomization process.

Domain 2: Risk of bias due to deviations from intended interventions

2.1: Y (High Certainty - 95%)

Reason: The article states "Patients and local members of the trial staff were aware of the assigned treatments."

2.2: Y (High Certainty - 95%)

Reason: As above, trial staff were aware of the assigned treatments.

2.3: N (Moderate Certainty - 85%)

Reason: No clear evidence of deviations from intended interventions beyond what would be expected in usual practice.

2.4: N/A

2.5: N/A

2.6: Y (High Certainty - 95%)

Reason: The article states "All information presented in this report is based on a data cutoff of December 14, 2020. Information regarding the primary and secondary outcomes is complete for 99.9% of trial participants."

2.7: N (High Certainty - 95%)

Reason: With 99.9% complete data, there is no substantial impact from failure to analyze participants in the group to which they were randomized.

Result of Domain 2: Low risk (High Certainty - 95%)

Reason: Despite participants and personnel being aware of assigned interventions, there is no evidence of deviation from intended interventions that likely impacted the outcome.

Domain 3: Missing outcome data

3.1: Y (High Certainty - 95%)

Reason: The article states "Information regarding the primary and secondary outcomes is complete for 99.9% of trial participants."

3.2: N (High Certainty - 95%)

Reason: With 99.9% complete data, no methods to correct for bias were necessary.

3.3: N (High Certainty - 95%)

Reason: With 99.9% complete data, missingness is unlikely to depend on the true value.

3.4: N/A

Result of Domain 3: Low risk (High Certainty - 95%)

Reason: Very little missing outcome data, unlikely to have influenced the results.

Domain 4: Risk of bias in measurement of the outcome

4.1: N (High Certainty - 95%)

Reason: All-cause mortality is an objective outcome that is unlikely to be inappropriately measured.

4.2: N (High Certainty - 95%)

Reason: All-cause mortality is unlikely to be measured differently between groups.

4.3: Y (High Certainty - 95%)

Reason: The trial was open-label, so outcome assessors were aware of intervention received.

4.4: N (High Certainty - 95%)

Reason: All-cause mortality is an objective outcome that is unlikely to be influenced by knowledge of the intervention received.

4.5: N (High Certainty - 95%)

Reason: As above, all-cause mortality is objective and unlikely to be influenced by knowledge of the intervention.

Result of Domain 4: Low risk (High Certainty - 95%)

Reason: While the trial was open-label, the primary outcome of all-cause mortality is objective and unlikely to be influenced by knowledge of the intervention.

Domain 5: Risk of bias in selection of the reported result

5.1: Y (High Certainty - 95%)

Reason: The trial was pre-registered (NCT04381936) and the primary outcome is reported as pre-specified.

5.2: N (High Certainty - 95%)

Reason: The primary outcome of 28-day mortality was clearly pre-specified and reported as such.

5.3: N (High Certainty - 95%)

Reason: There is no evidence of selection of the reported result from multiple analyses.

Result of Domain 5: Low risk (High Certainty - 95%)

Reason: The outcome was pre-specified and reported as planned, with no evidence of selection from multiple measurements or analyses.

Overall Bias Risk for the Article: Low risk (High Certainty - 95%)

Reason: All domains were assessed as low risk of bias, with high certainty for most judgments. The randomization process was appropriate, there were no substantial deviations from intended interventions, very little missing data, the primary outcome was objective, and reporting followed the pre-specified analysis plan.

## Study 11-2

Based on my review of the provided article, here is my assessment of the risk of bias:

**Article ID**: Horby, 2021

**Outcome Name**: All-cause mortality within 28 days after randomization

**registration number**: NCT04381936

Domain 1: Risk of bias arising from the randomization process

1.1: Y (High Certainty - 95%)

Reason: The article states "Randomization was performed with the use of a Web-based system with concealment of the trial-group assignment."

1.2: Y (High Certainty - 95%)

Reason: The article mentions "Randomization was performed with the use of a Web-based system with concealment of the trial-group assignment."

1.3: N (High Certainty - 95%)

Reason: The article states "There was a significant (P = 0.01) difference in the mean age between patients in the dexamethasone group and those in the usual care group, but there were no significant differences between the groups in any other baseline characteristic."

Result of the domain: Low risk (High Certainty - 95%)

Reason: Allocation sequence was adequately generated and concealed, and baseline differences do not suggest a problem with the randomization process.

Domain 2: Risk of bias due to deviations from intended interventions

2.1: Y (High Certainty - 95%)

Reason: The article states "Patients and local members of the trial staff were aware of the assigned treatments."

2.2: Y (High Certainty - 95%)

Reason: The article states "Patients and local members of the trial staff were aware of the assigned treatments."

2.3: N (Moderate Certainty - 85%)

Reason: No clear evidence of deviations from intended interventions due to trial context.

2.6: Y (High Certainty - 95%)

Reason: The article states "All information presented in this report is based on a data cutoff of December 14, 2020. Information regarding the primary and secondary outcomes is complete for 99.9% of trial participants."

2.7: N (High Certainty - 95%)

Reason: Nearly all randomized participants were analyzed.

Result of the domain: Low risk (High Certainty - 95%)

Reason: Participants and personnel were aware of assigned intervention, but this is unlikely to have affected outcomes. Appropriate analysis was used to estimate the effect of assignment to intervention.

Domain 3: Missing outcome data

3.1: Y (High Certainty - 95%)

Reason: The article states "Information regarding the primary and secondary outcomes is complete for 99.9% of trial participants."

3.2: N (High Certainty - 95%)

Reason: With nearly complete outcome data, there is no evidence that results were biased by missing data.

3.3: N (High Certainty - 95%)

Reason: With nearly complete outcome data, missingness in the outcome is unlikely to depend on its true value.

3.4: N (High Certainty - 95%)

Reason: With nearly complete outcome data, missingness in the outcome is unlikely to depend on its true value.

Result of the domain: Low risk (High Certainty - 95%)

Reason: Nearly complete outcome data for mortality.

Domain 4: Risk of bias in measurement of the outcome

4.1: N (High Certainty - 95%)

Reason: All-cause mortality is an unambiguous outcome.

4.2: N (High Certainty - 95%)

Reason: All-cause mortality is unlikely to be affected by knowledge of intervention received.

4.3: Y (High Certainty - 95%)

Reason: The trial was open-label, so outcome assessors were aware of intervention received.

4.4: N (High Certainty - 95%)

Reason: All-cause mortality is an objective outcome unlikely to be influenced by knowledge of intervention received.

4.5: N (High Certainty - 95%)

Reason: All-cause mortality is an objective outcome unlikely to be influenced by knowledge of intervention received.

Result of the domain: Low risk (High Certainty - 95%)

Reason: The outcome measure is unlikely to be influenced by knowledge of the intervention received.

Domain 5: Risk of bias in selection of the reported result

5.1: Y (High Certainty - 95%)

Reason: The trial was registered (NCT04381936) and the primary outcome matches the registration.

5.2: N (High Certainty - 95%)

Reason: The reported outcome measurements align with those pre-specified.

5.3: N (High Certainty - 95%)

Reason: The reported outcome analyses align with those pre-specified.

Result of the domain: Low risk (High Certainty - 95%)

Reason: The outcome measurements and analyses were clearly pre-specified and reported as planned.

Overall Bias Risk for the Article: Low risk (High Certainty - 95%)

Reason: All domains were assessed as low risk of bias.

## Study 12-1

Here is my evaluation of the risk of bias for the randomized controlled trial by Beautrais et al. (2010):

**Article ID**: Beautrais, 2010

**Outcome Name**: Self-harm re-presentations

Domain 1: Risk of bias arising from the randomization process

1.1: Y (90% confidence)

Reason: The study states that "Randomisation was based on a random number sequence available only to research staff. The number sequence was computer-generated in SAS 9.1 for Windows using a uniform distribution to generate a sequence of random numbers between 0 and 1."

1.2: PY (80% confidence)

Reason: The study mentions that "Randomisation was performed post-recruitment and post-consent by research staff who were not involved in the recruitment or clinical care of participants." While this suggests allocation concealment, it does not explicitly state that the allocation sequence was concealed until participants were enrolled and assigned.

1.3: Y (85% confidence)

Reason: Table 2 shows a significant difference between groups in the number of prior self-harm visits in the past 12 months (p<0.07), suggesting a problem with the randomization process.

Result of Domain 1: High risk (85% confidence)

Reason: While the randomization method was appropriate, there is evidence of baseline imbalances between groups, indicating a potential problem with the randomization process.

Domain 2: Risk of bias due to deviations from intended interventions

2.1: Y (90% confidence)

Reason: Participants would be aware of whether they received postcards or not.

2.2: Y (90% confidence)

Reason: The intervention (mailing postcards) would be known to staff implementing it.

2.3: NI (80% confidence)

Reason: The study does not report any deviations from the intended intervention arising from the trial context.

2.4: NI (80% confidence)

Reason: No information is provided to assess if any deviations affected the outcome.

2.5: NI (80% confidence)

Reason: No information is provided to assess if any deviations were balanced between groups.

2.6: Y (90% confidence)

Reason: The study states "All statistical analysis was performed in SAS 9.1 for Windows. The results of the trial were analysed using the intention-to-treat design."

2.7: N (85% confidence)

Reason: An intention-to-treat analysis was used, and all randomized participants were included in the analysis.

Result of Domain 2: Some concerns (80% confidence)

Reason: While participants and staff were aware of the intervention, an appropriate intention-to-treat analysis was used. However, there is insufficient information about potential deviations from intended interventions.

Domain 3: Missing outcome data

3.1: Y (95% confidence)

Reason: Figure 1 shows that all randomized participants (153 in intervention group, 174 in control group) were followed up and analyzed.

3.2: NI (80% confidence)

Reason: The study does not provide information about methods to handle missing data or conduct sensitivity analyses.

3.3: N (90% confidence)

Reason: Outcome data appears to be complete for all participants, so missingness is unlikely to depend on its true value.

3.4: N (90% confidence)

Reason: As outcome data is complete, it is unlikely that missingness depends on the true value.

Result of Domain 3: Low risk (90% confidence)

Reason: Outcome data appears to be complete for all randomized participants.

Domain 4: Risk of bias in measurement of the outcome

4.1: N (90% confidence)

Reason: The outcome measures (re-presentations for self-harm) seem appropriate and were assessed through hospital records.

4.2: N (85% confidence)

Reason: The study states that re-presentations were assessed in the same way for both groups using psychiatric emergency service records and hospital medical records.

4.3: N (85% confidence)

Reason: The study states "Participants' randomisation status was not conveyed to clinical or data-collection staff."

4.4: N (90% confidence)

Reason: Re-presentations for self-harm assessed through hospital records are an objective measure unlikely to be influenced by knowledge of the intervention.

4.5: N (90% confidence)

Reason: As an objective measure from hospital records, assessment of the outcome was unlikely to be influenced by knowledge of the intervention received.

Result of Domain 4: Low risk (85% confidence)

Reason: The outcome measure was appropriate, collected similarly for both groups, and assessors were blinded to intervention status.

Domain 5: Risk of bias in selection of the reported result

5.1: Y (85% confidence)

Reason: The analysis methods described in the Methods section match those reported in the Results, and all outcomes mentioned in Methods are reported in Results.

5.2: N (80% confidence)

Reason: The reported results seem to correspond to all intended outcome measurements described in the Methods.

5.3: N (80% confidence)

Reason: The reported analyses appear to correspond to the planned analyses described in the Methods section.

Result of Domain 5: Low risk (80% confidence)

Reason: The reported results correspond to the pre-specified analyses and outcome measures.

Overall Bias Risk for the Article: High risk (85% confidence)

The main concern is the baseline imbalance between groups in prior self-harm visits, indicating a potential problem with the randomization process. While other domains show low risk of bias, this issue in Domain 1 leads to an overall high risk of bias assessment.

## Study 12-2

I apologize for the confusion. I'll provide a complete assessment of all domains based on the information given and the corrections you provided.

Article ID: Beautrais, 2010

Outcome Name: Self-harm re-presentations

Domain 1: Risk of bias arising from the randomization process

1.1: Y (95% confidence)

Reason: The study used computer-generated random numbers for randomization.

1.2: Y (95% confidence)

Reason: The study used central allocation for concealment.

1.3: Y (80% confidence)

Reason: There was a significant difference between groups in number of prior self-harm visits in the past 12 months, suggesting a problem with the randomization process.

Result of Domain 1: Some concerns (85% confidence)

Reason: While adequate randomization and allocation concealment methods were used, there was a significant baseline imbalance between groups, which raises some concerns about the randomization process.

Domain 2: Risk of bias due to deviations from intended interventions

2.1: PY (60% confidence)

Reason: Participants likely aware of intervention due to receiving postcards, but not explicitly stated.

2.2: PY (60% confidence)

Reason: Staff likely aware of intervention group due to sending postcards, but not explicitly stated.

2.3: NI (70% confidence)

Reason: No information provided on deviations from intended interventions.

2.4: NI (70% confidence)

Reason: No information to judge if deviations affected outcomes.

2.5: NI (70% confidence)

Reason: No information to judge if deviations were balanced between groups.

2.6: Y (80% confidence)

Reason: Study states intention-to-treat analysis was used.

2.7: N (80% confidence)

Reason: All randomized participants were analyzed in their assigned groups.

Result of Domain 2: Some concerns (70% confidence)

Reason: Participants and staff likely aware of intervention, but appropriate analysis used and no evidence of substantial impact from deviations.

Domain 3: Missing outcome data

3.1: Y (90% confidence)

Reason: Outcome data available for all randomized participants.

3.2: Y (80% confidence)

Reason: Intention-to-treat analysis used, so unlikely to be biased by missing data.

3.3: N (80% confidence)

Reason: No missing outcome data.

3.4: N (80% confidence)

Reason: No missing outcome data.

Result of Domain 3: Low risk (85% confidence)

Reason: Complete outcome data available for all participants and appropriate analysis used.

Domain 4: Risk of bias in measurement of the outcome

4.1: N (80% confidence)

Reason: Self-harm re-presentations measured through hospital records, which is an appropriate method.

4.2: N (80% confidence)

Reason: Same measurement method used for both groups.

4.3: N (80% confidence)

Reason: Outcome assessed through hospital records, so assessors likely unaware of intervention received.

4.4: N (80% confidence)

Reason: Self-harm re-presentations is an objective outcome unlikely to be influenced by knowledge of intervention.

4.5: N (80% confidence)

Reason: No clear evidence that assessment was influenced by knowledge of intervention.

Result of Domain 4: Low risk (80% confidence)

Reason: Objective outcome measure from hospital records, unlikely to be biased by knowledge of intervention.

Domain 5: Risk of bias in selection of the reported result

5.1: Y (80% confidence)

Reason: Analysis appears to follow pre-specified plan described in methods.

5.2: N (80% confidence)

Reason: Single pre-specified outcome measure reported.

5.3: N (80% confidence)

Reason: Single pre-specified analysis approach reported.

Result of Domain 5: Low risk (80% confidence)

Reason: Analysis and reporting appear to follow pre-specified plan with no evidence of selection based on results.

Overall Bias Risk for the Article: Some concerns (80% confidence)

The "Some concerns" assessment is primarily due to the baseline imbalance between groups in prior self-harm visits, despite adequate randomization and allocation concealment methods. While most domains showed low risk of bias, the imbalance at baseline and some concerns about blinding raise some concerns about the overall validity of the trial results.

## Study 13-1

Here is my evaluation of the risk of bias for the specified outcome in the Huhn 2020 study:

**Article ID**: Huhn, 2020

**Outcome Name**: Service use - readmission to hospital (combined time points)

**Registration number**: NCT02307396

Domain 1: Risk of bias arising from the randomization process

1.1: NI (75% confidence)

Reason: The article does not describe specific methods for generating the random sequence, only stating "The randomization list was generated by an independent psychologist using the RAND function of Microsoft Excel 2010."

1.2: Y (90% confidence)

Reason: The article states "Only once the patient was recorded, allocation was made known to the treating psychiatrist and the participant", indicating allocation was concealed until enrollment.

1.3: N (80% confidence)

Reason: Table 1 shows baseline characteristics were generally similar between groups, with no major imbalances suggestive of problems with randomization.

Result of domain 1: Low risk (80% confidence)

Reason: While sequence generation methods are not fully clear, allocation was concealed and baseline characteristics were balanced, suggesting the randomization process was likely adequate overall.

Domain 2: Risk of bias due to deviations from intended interventions

2.1: Y (95% confidence)

Reason: The study design was open-label, so participants were aware of their assigned intervention.

2.2: Y (95% confidence)

Reason: As an open-label study, the clinicians delivering the interventions were aware of assignments.

2.3: N (80% confidence)

Reason: No deviations from intended interventions arising from the trial context are reported.

2.4: N/A

2.5: N/A

2.6: Y (90% confidence)

Reason: The article states "The intention-to-treat-sample consisted of all participants randomized into the trial", indicating an appropriate ITT analysis was used.

2.7: N (85% confidence)

Reason: All randomized participants were analyzed in their assigned groups.

Result of domain 2: Some concerns (80% confidence)

Reason: While participants and clinicians were aware of assignments, appropriate analysis methods were used and no major deviations were reported.

Domain 3: Missing outcome data

3.1: Y (95% confidence)

Reason: Outcome data was available for all 20 randomized participants.

3.2: Y (90% confidence)

Reason: With complete outcome data, there is no evidence of bias due to missing data.

3.3: N (85% confidence)

Reason: With complete outcome data, missingness could not depend on true values.

3.4: N/A

Result of domain 3: Low risk (90% confidence)

Reason: Outcome data was complete for all participants.

Domain 4: Risk of bias in measurement of the outcome

4.1: N (85% confidence)

Reason: Readmission to hospital is an objective outcome that can be reliably measured.

4.2: N (80% confidence)

Reason: The article does not indicate any differences in measurement between groups.

4.3: Y (95% confidence)

Reason: In this open-label study, outcome assessors were aware of intervention assignments.

4.4: N (85% confidence)

Reason: Readmission to hospital is an objective outcome unlikely to be influenced by knowledge of the intervention received.

4.5: N (80% confidence)

Reason: There is no clear evidence that assessment of this objective outcome was influenced by knowledge of the intervention.

Result of domain 4: Low risk (80% confidence)

Reason: While assessors were aware of assignments, the outcome is objective and unlikely to be influenced by this knowledge.

Domain 5: Risk of bias in selection of the reported result

5.1: Y (90% confidence)

Reason: The study was registered (NCT02307396) and the reported analyses appear to align with the pre-specified plan.

5.2: N (85% confidence)

Reason: There is no evidence of selection from multiple outcome measurements or analyses.

5.3: N (85% confidence)

Reason: There is no evidence of selection from multiple analyses of the outcome.

Result of domain 5: Low risk (85% confidence)

Reason: The results appear to be reported as pre-specified without evidence of selection based on results.

Overall Bias Risk for the Article: Low risk (80% confidence)

The study has some methodological limitations, particularly related to blinding, but uses appropriate analysis methods and has complete outcome data for an objective primary outcome. While some concerns remain, the overall risk of bias appears low for this outcome.

## Study 13-2

Here is my assessment of the risk of bias for the specified outcome in this study:

**Article ID**: Huhn, 2021

**Outcome Name**: Service use - readmission to hospital (combined time points)

**Registration number**: NCT02307396

Domain 1

1.1: NI (75% confidence)

Reason: The article only mentions random grouping without describing specific methods for generating random sequences.

1.2: NI (75% confidence)

Reason: The article does not mention allocation concealment methods.

1.3: N (90% confidence)

Reason: There were no significant differences between groups in baseline characteristics, except for family history of psychiatric illness.

Result of Domain 1: Some concerns (80% confidence)

Reason: Allocation concealment is unclear, but baseline characteristics were generally balanced between groups.

Domain 2

2.1: Y (90% confidence)

Reason: The study was described as "single-blind", implying participants were aware of their allocation.

2.2: Y (90% confidence)

Reason: The intervention involved medication reduction, which providers would be aware of.

2.3: N (80% confidence)

Reason: No deviations from intended interventions due to trial context were reported.

2.4: N/A

2.5: N/A

2.6: Y (80% confidence)

Reason: The article states "The intention-to-treat-sample consisted of all participants randomized into the trial."

2.7: N (80% confidence)

Reason: All randomized participants were included in the analysis.

Result of Domain 2: Some concerns (80% confidence)

Reason: Participants and providers were aware of allocations, but this was unlikely to significantly impact results given the objective nature of the outcome.

Domain 3

3.1: Y (90% confidence)

Reason: Only 1 participant out of 20 (5%) was lost to follow-up.

3.2: N (80% confidence)

Reason: No evidence of methods to correct for potential bias from the single missing participant.

3.3: N (80% confidence)

Reason: The single missing participant stopped medication against protocol, suggesting missingness was likely unrelated to true outcome value.

3.4: N/A

Result of Domain 3: Low risk (85% confidence)

Reason: Very little missing data, and missingness likely unrelated to true outcome.

Domain 4

4.1: N (90% confidence)

Reason: Hospital readmission is an objective outcome measure.

4.2: N (90% confidence)

Reason: The outcome was measured the same way for both groups.

4.3: Y (90% confidence)

Reason: Outcome assessors were likely aware of interventions since the study was single-blind.

4.4: N (90% confidence)

Reason: Hospital readmission is an objective outcome unlikely to be influenced by knowledge of intervention.

4.5: N (90% confidence)

Reason: No clear evidence that assessment was influenced by knowledge of intervention.

Result of Domain 4: Low risk (90% confidence)

Reason: Objective outcome measure unlikely to be influenced by lack of blinding.

Domain 5

5.1: NI (75% confidence)

Reason: The article does not clearly state if analysis plans were pre-specified before unblinded data was available.

5.2: N (80% confidence)

Reason: Hospital readmission is a straightforward outcome without multiple ways to measure.

5.3: N (80% confidence)

Reason: No evidence of multiple analyses of the data.

Result of Domain 5: Some concerns (75% confidence)

Reason: Unclear if analysis plans were pre-specified, but low risk of selective reporting given the nature of the outcome.

Overall Bias Risk for the Article: Some concerns (80% confidence)

Reason: Some concerns in domains 1, 2 and 5, but low risk in domains 3 and 4. The main issues are unclear allocation concealment and analysis plans, but the objective nature of the outcome mitigates many potential sources of bias.

## Study 14-1

Here is my assessment of the risk of bias for the study by Ooi et al., 2021:

**Article ID**: Ooi, 2021

**Outcome Name**: Weight loss

Domain 1: Risk of bias arising from the randomization process

1.1: NI (75% certainty)

Reason: The study mentions "block randomly assigned" but does not describe specific randomization methods.

1.2: NI (75% certainty)

Reason: The article does not mention allocation concealment methods.

1.3: N (90% certainty)

Reason: Table 2 shows no significant differences in baseline characteristics between groups.

Result of Domain 1: Some concerns (80% certainty)

Reason: Allocation concealment is unclear, but baseline characteristics appear balanced.

Domain 2: Risk of bias due to deviations from intended interventions

2.1: Y (90% certainty)

Reason: Participants were aware of their diet assignment.

2.2: Y (90% certainty)

Reason: Dietitians providing counseling were aware of group assignments.

2.3: NI (70% certainty)

Reason: No information provided on deviations from intended interventions.

2.4: NI (70% certainty)

Reason: Insufficient information to determine if deviations affected outcomes.

2.5: NI (70% certainty)

Reason: No information on whether deviations were balanced between groups.

2.6: Y (90% certainty)

Reason: Analysis appears to include all randomized participants.

2.7: N (80% certainty)

Reason: No evidence of substantial impact from analyzing participants in assigned groups.

Result of Domain 2: Some concerns (75% certainty)

Reason: Participants and personnel were aware of interventions, but impact of any deviations is unclear.

Domain 3: Risk of bias due to missing outcome data

3.1: PN (85% certainty)

Reason: Completion rate was approximately 83% (109/132).

3.2: N (80% certainty)

Reason: No information provided on methods to handle missing data.

3.3: PY (70% certainty)

Reason: Reasons for dropout not fully reported; may relate to difficulty adhering to diet.

3.4: NI (70% certainty)

Reason: Insufficient information to determine if missingness related to outcome.

Result of Domain 3: Some concerns (75% certainty)

Reason: Moderate amount of missing data with unclear reasons and handling.

Domain 4: Risk of bias in measurement of the outcome

4.1: N (90% certainty)

Reason: Weight measurement is an appropriate and reliable method.

4.2: N (90% certainty)

Reason: Weight likely measured consistently across groups.

4.3: N (85% certainty)

Reason: Weight measurement unlikely to be influenced by assessor knowledge of intervention.

4.4: N (90% certainty)

Reason: Weight is an objective measure unlikely to be influenced by knowledge of intervention.

4.5: N (90% certainty)

Reason: No evidence that assessment was influenced by knowledge of intervention.

Result of Domain 4: Low risk (90% certainty)

Reason: Weight is an objective measure unlikely to be biased by knowledge of intervention.

Domain 5: Risk of bias in selection of the reported result

5.1: NI (70% certainty)

Reason: No information provided on pre-specified analysis plan.

5.2: N (80% certainty)

Reason: Reported weight outcomes appear to align with methods.

5.3: N (80% certainty)

Reason: No evidence of selection of results based on multiple analyses.

Result of Domain 5: Some concerns (75% certainty)

Reason: Lack of information on pre-specified analysis plan, but reported results appear appropriate.

Overall Bias Risk for the Article: Some concerns (80% certainty).

## Study 14-2

Thank you for the additional guidance. I will reassess the study based on these instructions and provide a revised evaluation.

**Article ID**: Ooi, 2021

**Outcome Name**: Weight loss

Domain 1: Risk of bias arising from the randomization process

1.1: NI (80% confidence)

Reason: The study mentions "block randomly assigned" but does not describe specific randomization methods.

1.2: NI (80% confidence)

Reason: No information on allocation concealment. No mention of central allocation, envelopes, or postcards.

1.3: N (80% confidence)

Reason: Table 2 shows baseline characteristics were similar between groups.

Result of Domain 1: Some concerns (80% confidence)

Reason: Randomization performed, but details on method and allocation concealment lacking.

Domain 2: Risk of bias due to deviations from the intended interventions

2.1: Y (90% confidence)

Reason: Participants were aware of their diet assignment, as meals were provided.

2.2: Y (90% confidence)

Reason: Those delivering the interventions likely aware of group assignments.

2.3: NI (70% confidence)

Reason: No report on deviations from intended interventions.

2.4: NI (70% confidence)

Reason: No information on whether deviations affected the outcome.

2.5: NI (70% confidence)

Reason: No information on balance of deviations between groups.

2.6: NI (80% confidence)

Reason: No clear evidence of using ITT, mITT, or other appropriate analysis methods.

2.7: NI (70% confidence)

Reason: Unable to determine impact on results without clear information on analysis method.

Result of Domain 2: Some concerns (75% confidence)

Reason: Participants and personnel were aware of assignments, unclear impact of potential deviations.

Domain 3: Risk of bias due to missing outcome data

3.1: N (85% confidence)

Reason: 109 out of 132 randomized participants included in analysis, which is less than 95%.

3.2: N (80% confidence)

Reason: No mention of methods to correct for bias due to missing data.

3.3: NI (70% confidence)

Reason: No information on reasons for missing data.

3.4: NI (70% confidence)

Reason: Unable to determine if missingness related to true outcome value.

Result of Domain 3: High risk (80% confidence)

Reason: Less than 95% of randomized participants included in analysis, no correction for missing data.

Domain 4: Risk of bias in measurement of the outcome

4.1: N (90% confidence)

Reason: Weight measurement is appropriate and reliable.

4.2: N (90% confidence)

Reason: Weight likely measured consistently across groups.

4.3: N (85% confidence)

Reason: Weight measurement is objective, unlikely affected by assessor awareness.

4.4: N (90% confidence)

Reason: Weight is an objective measure unlikely influenced by knowledge of intervention.

4.5: N (90% confidence)

Reason: No evidence that assessment was influenced by intervention knowledge.

Result of Domain 4: Low risk (90% confidence)

Reason: Weight is an objective measure unlikely to be biased.

Domain 5: Risk of bias in selection of the reported result

5.1: NI (80% confidence)

Reason: No information on pre-specified analysis plan or trial registration.

5.2: N (80% confidence)

Reason: Reported results align with methods described.

5.3: N (80% confidence)

Reason: No evidence of selecting from multiple analyses.

Result of Domain 5: Some concerns (80% confidence)

Reason: Lack of pre-specified analysis plan, but no clear evidence of selective reporting.

Overall Bias Risk for the Article: High risk (80% confidence)

## Study 15-1

Here is my assessment of the risk of bias for the article based on the provided instructions:

**Article ID**: Kawanishi, 2014

**Outcome Name**: Recurrent suicidal behavior (attempted suicide or completed suicide)

**Registration number**: NCT00736918 and UMIN-CTR (C000000444)

Domain 1

1.1: Y (95% confidence)

Reason: The study used an internet-based randomization system, which implies computer-generated random allocation.

1.2: Y (95% confidence)

Reason: The study used an internet-based system operated by a central, independent data centre for allocation.

1.3: N (90% confidence)

Reason: Baseline characteristics were well balanced between groups, suggesting no problems with randomization.

Result of Domain 1: Low risk (95% confidence)

Reason: Allocation was adequately generated and concealed, with no baseline imbalances.

Domain 2

2.1: Y (95% confidence)

Reason: Participants were not blinded to their assigned intervention.

2.2: Y (95% confidence)

Reason: Case managers providing interventions were not blinded.

2.3: NI (90% confidence)

Reason: The article does not report any deviations from the intended interventions.

2.4: NI (90% confidence)

Reason: No information provided on whether deviations affected the outcome.

2.5: NI (90% confidence)

Reason: No information provided on whether deviations were balanced between groups.

2.6: Y (90% confidence)

Reason: Analyses were done according to the intention-to-treat principle.

2.7: N (90% confidence)

Reason: Analyses were done according to intention-to-treat, so participants were analyzed in their assigned groups.

Result of Domain 2: Some concerns (80% confidence)

Reason: Participants and personnel were not blinded, but analyses were appropriate.

Domain 3

3.1: Y (95% confidence)

Reason: Data was available for over 95% of randomized participants.

3.2: N (90% confidence)

Reason: No evidence provided that results were not biased by missing data.

3.3: PN (80% confidence)

Reason: No clear indication that missingness depended on true outcome values.

3.4: PN (80% confidence)

Reason: No evidence suggesting missingness depended on true outcome values.

Result of Domain 3: Low risk (90% confidence)

Reason: Minimal missing data and no clear evidence of bias from missing data.

Domain 4

4.1: N (90% confidence)

Reason: The outcome measurement method appears appropriate.

4.2: N (90% confidence)

Reason: No indication of differential measurement between groups.

4.3: N (95% confidence)

Reason: Outcome assessors were masked to group allocation.

4.4: N (90% confidence)

Reason: The primary outcome is relatively objective.

4.5: N (90% confidence)

Reason: No clear evidence that assessment was influenced by knowledge of intervention.

Result of Domain 4: Low risk (90% confidence)

Reason: Appropriate outcome measurement with blinded assessors.

Domain 5

5.1: Y (95% confidence)

Reason: The study was registered and analyses appear to follow the pre-specified plan.

5.2: N (90% confidence)

Reason: No indication of selection of results from multiple outcome measurements.

5.3: N (90% confidence)

Reason: No indication of selection of results from multiple analyses.

Result of Domain 5: Low risk (90% confidence)

Reason: Pre-specified analysis plan was followed with no evidence of selective reporting.

Overall Bias Risk for the Article: Low risk (85% confidence)

Reason: Most domains were assessed as low risk, with only some concerns for Domain 2 due to lack of blinding. The use of appropriate randomization, allocation concealment, outcome assessment, and analysis methods mitigate potential biases.

## Study 15-2

I understand. I will carefully review the provided article and assess the risk of bias for the specified outcome using the given criteria. I will follow the instructions closely, avoid subjective judgments, and provide a comprehensive evaluation with confidence ratings for each domain and the overall assessment. I'll now proceed with the analysis.

**Article ID**: Kawanishi, 2014

**Outcome Name**: Recurrent suicidal behavior (attempted suicide or completed suicide)

**registration number**: NCT00736918 and UMIN-CTR (C000000444)

Domain 1

1.1: Y (High Certainty 95%)

Reason: The study used an internet-based randomization system with minimisation method for sequence generation.

1.2: Y (High Certainty 95%)

Reason: The study used central allocation via an internet-based system operated by an independent data centre.

1.3: N (High Certainty 95%)

Reason: Baseline characteristics were well balanced between the groups, as stated in the article.

Result of the domain: Low risk (High Certainty 95%)

Reason: The allocation sequence was adequately generated and concealed, and there were no baseline differences suggesting problems with the randomization process.

Domain 2

2.1: Y (High Certainty 95%)

Reason: Participants were aware of their assigned intervention as the study was not blinded.

2.2: Y (High Certainty 95%)

Reason: Carers and people delivering the interventions were aware of participants' assigned intervention as the study was not blinded.

2.3: NI (High Certainty 95%)

Reason: The article does not report any deviations from the intended intervention.

2.4: NI (High Certainty 95%)

Reason: The article does not provide information on whether deviations affected the outcome.

2.5: NI (High Certainty 95%)

Reason: The article does not provide information on whether deviations were balanced between groups.

2.6: Y (Moderate Certainty 85%)

Reason: The study used intention-to-treat analysis, which is an appropriate method to estimate the effect of assignment to intervention.

2.7: N (High Certainty 95%)

Reason: All randomized participants were included in the analysis.

Result of the domain: Some concerns (Moderate Certainty 85%)

Reason: While participants and providers were aware of the intervention, there's no clear evidence of deviations from intended interventions. The use of intention-to-treat analysis mitigates some concerns.

Domain 3

3.1: Y (High Certainty 95%)

Reason: The article states that 460 participants were included in the intention-to-treat analysis for the intervention group and 454 for the control group, which is nearly all randomized participants.

3.2: N (High Certainty 95%)

Reason: The article does not provide evidence that the result was not biased by missing outcome data.

3.3: N (Moderate Certainty 80%)

Reason: There's no indication that missingness in the outcome depended on its true value.

3.4: N (Moderate Certainty 80%)

Reason: Based on the available information, it's unlikely that missingness in the outcome depended on its true value.

Result of the domain: Low risk (High Certainty 95%)

Reason: Data were available for nearly all randomized participants, and there's no evidence suggesting that missing data biased the results.

Domain 4

4.1: N (High Certainty 95%)

Reason: The method of measuring the outcome (recurrent suicidal behavior) appears appropriate.

4.2: N (High Certainty 95%)

Reason: The measurement of the outcome was consistent between intervention groups.

4.3: N (High Certainty 95%)

Reason: The article states that outcome assessors were masked to group allocation.

4.4: N (High Certainty 95%)

Reason: As outcome assessors were blinded, assessment of the outcome was unlikely to be influenced by knowledge of the intervention received.

4.5: N (High Certainty 95%)

Reason: There is no clear evidence to suggest that assessment of the outcome was influenced by knowledge of intervention received.

Result of the domain: Low risk (High Certainty 95%)

Reason: The outcome measurement methods were appropriate, consistent between groups, and assessors were blinded.

Domain 5

5.1: Y (High Certainty 95%)

Reason: The study was registered (NCT00736918 and UMIN-CTR C000000444) and the reported analyses align with the pre-specified plan.

5.2: N (High Certainty 95%)

Reason: The reported results correspond to all intended outcome measurements.

5.3: N (High Certainty 95%)

Reason: The reported analyses correspond to the pre-specified analysis plan.

Result of the domain: Low risk (High Certainty 95%)

Reason: The results were analyzed and reported according to a pre-specified plan, with no evidence of selection based on results.

Overall Bias Risk for the Article: Some concerns (Moderate Certainty 85%)

The overall risk of bias for this article is assessed as "Some concerns" primarily due to the lack of blinding of participants and providers (Domain 2). However, the study demonstrates strong methodological qualities in other domains, including proper randomization and allocation concealment, appropriate handling of outcome data, blinded outcome assessment, and adherence to a pre-specified analysis plan. The use of intention-to-treat analysis helps mitigate some of the concerns raised by the lack of blinding. While not achieving a "Low risk" rating overall, the study appears to have been conducted with generally high methodological rigor.

## Study 16-1

**Article ID**: Liljeroos, 2015

**Outcome Name**: Hospital admission

Domain 1

1.1 NI (High Certainty - 95%)

The article only mentions that dyads were randomized, but does not specify the methods used to generate the allocation sequence.

1.2 NI (High Certainty - 95%)

The article does not mention any methods used to conceal the allocation sequence such as central allocation or opaque envelopes.

1.3 N (Moderate Certainty - 80%)

The article states there were no differences between the patients or partners in the control and intervention group at baseline, suggesting no problems with the randomization process.

Some concerns (High Certainty - 95%)

Based on the lack of information about allocation sequence generation and concealment, there are some concerns regarding the risk of bias arising from the randomization process, despite the groups appearing balanced at baseline.

Domain 2

2.1 Y (High Certainty - 95%)

As this was a non-blinded trial, participants were aware of their assigned intervention.

2.2 Y (High Certainty - 95%)

The nurses delivering the intervention were aware of the participants' assigned intervention.

2.3 NI (High Certainty - 95%)

After searching the article again, no information was found on whether there were deviations from the intended intervention that arose because of the trial context.

2.4 NI (High Certainty - 95%)

After searching the article again, no information was found on whether deviations from the intended intervention were likely to have affected the outcome.

2.5 NI (High Certainty - 95%)

After searching the article again, no information was found on whether deviations from the intended intervention were balanced between groups.

2.6 Y (Low Certainty - 60%)

The article states that the results were analyzed using intention-to-treat analysis including all randomized dyads, suggesting an appropriate analysis to estimate the effect of assignment to intervention.

2.7 NI (High Certainty - 95%)

After searching the article again, no information was found on whether there was potential for a substantial impact on the result from failure to analyze participants in the group to which they were randomized.

Some concerns (High Certainty - 95%)

The lack of blinding raises some concerns, and there is insufficient information to determine whether deviations arose, their potential impact, and whether they were balanced between groups. However, an intention-to-treat analysis was reportedly used. Overall, there are some concerns regarding risk of bias due to deviations from the intended interventions.

Domain 3

3.1 Y (High Certainty - 100%)

Table 3 in the article provides readmission data for all randomized participants - 71 in the intervention group and 84 in the control group. This indicates that outcome data was available for all, or nearly all, participants randomized.

3.2 PY (Low Certainty - 60%)

Although outcome data was available for all randomized participants, the article does not clearly describe methods used to ensure the result was not biased by missing outcome data.

3.3 PN (Moderate Certainty - 80%)

As data was available for all randomized participants, it is unlikely that missingness in the outcome depended on its true value.

3.4 PN (Moderate Certainty - 80%)

As data was available for all randomized participants, it is unlikely that missingness in the outcome depended on its true value.

Low risk (High Certainty - 100%)

Outcome data was available for all randomized participants, as shown in Table 3. This represents low risk of bias due to missing outcome data.

Domain 4

4.1 N (High Certainty - 95%)

Hospital readmission is an objective, reliable outcome measure.

4.2 N (High Certainty - 95%)

There is no indication that the measurement or ascertainment of hospital readmissions differed between the intervention groups. This is routinely collected data.

4.3 N (High Certainty - 95%)

Outcome assessors would not be aware of intervention received, as hospital admission data is collected routinely and objectively.

4.4 N (High Certainty - 100%)

The outcome of hospital admission is objective and not likely to be influenced by knowledge of the intervention received.

4.5 N (High Certainty - 100%)

There is no evidence that the assessment of hospital admissions was influenced by knowledge of intervention received. This is objective data routinely collected in patient records.

Low risk (High Certainty - 100%)

The outcome was objectively measured through hospital records and is very unlikely to be influenced by knowledge of the intervention received. There is no evidence of any issues with the outcome measurement.

Domain 5

5.1 NI (High Certainty - 95%)

After searching the article again, no information was found on whether the data that produced the result were analyzed in accordance with a pre-specified plan that was finalized before unblinded outcome data were available.

5.2 N (High Certainty - 95%)

Hospital admission is an objective outcome that can only be measured in one way. There is no indication that the reported outcome was selected from multiple eligible outcome measurements.

5.3 N (High Certainty - 95%)

Hospital admission is an objective outcome that allows only one main method of analysis. There is no indication that the reported result was selected from multiple eligible analyses of the data.

Some concerns (High Certainty - 95%)

The lack of information about a pre-specified analysis plan raises some concerns about possible selective reporting. However, for this objective outcome, selective reporting of the measurement or analysis is unlikely.

Overall Bias Risk for the Article: Some concerns (Moderate Certainty - 80%)

There are some concerns related to the randomization process, deviations from intended interventions, and selection of the reported results. However, the risk of bias due to missing outcome data is low, as outcome data was available for all randomized participants per Table 3. Measurement of the outcome also appears to be at low risk of bias. Considering these factors together, there are some remaining concerns about risk of bias for this outcome.

## Study 16-2

Thank you for the feedback. I have carefully re-evaluated the article based on your suggestions. Here is the updated assessment:

**Article ID**: Liljeroos, 2015

**Outcome Name**: Hospital admission

Domain 1

1.1 : NI (95% Confidence)

The article does not mention the methods used for generating the random allocation sequence.

1.2 : NI (95% Confidence)

The article does not mention any methods of allocation concealment such as central allocation or sequentially numbered opaque envelopes.

1.3 : NI (100% Confidence)

The article does not provide any information regarding differences in baseline characteristics between the intervention and control groups.

Some concerns (95% Confidence)

Based on the rules provided, if 1.2 is NI and 1.3 is NI, the domain should be rated as some concerns.

Domain 2

2.1 : PY (80% Confidence)

As this was an educational intervention, participants were likely aware of their group assignment, though it is not explicitly stated.

2.2 : PY (80% Confidence)

The intervention involved nurses delivering education to the dyads, so the nurses were likely aware of group assignments.

2.3 : NI (100% Confidence)

No information is provided about deviations from intended intervention due to trial context.

2.4 : NI (100% Confidence)

No information is provided to judge if deviations were likely to impact the outcome.

2.5 : NI (100% Confidence)

No information is provided to judge if deviations were balanced between groups.

2.6 : Y (90% Confidence)

The article states they used intention-to-treat analysis, which is an appropriate method to estimate the effect of assignment to intervention.

2.7 : PN (80% Confidence)

The number analyzed (155 dyads) matches the number randomized, so there is probably little potential for impact from failing to analyze participants in the assigned group. However, without more details on those not included in the 24-month analysis, some minor uncertainty remains.

Some concerns (80% Confidence)

There are several "NI" ratings and the information to firmly judge 2.7 is limited. Per the criteria, this leads to an overall domain rating of some concerns.

Domain 3

3.1 : Y (100% Confidence)

The article states they used intention-to-treat analysis including all randomized dyads. For a binary outcome like hospital admission, this suggests data was available for nearly all randomized participants.

Low Risk (100% Confidence)

Since 3.1 is Y, this domain is low risk.

Domain 4

4.1 : N (100% Confidence)

The outcomes of death and hospital admission are objective outcomes unlikely to be influenced by the measurement method.

4.2 : PN (90% Confidence)

The outcomes of death and hospital admission were likely ascertained the same way between groups, but this is not explicitly stated.

4.3 : PN (90% Confidence)

For the objective outcomes of death and hospital admission, it is unlikely assessors were aware of intervention received, but this is not explicitly stated.

4.4 : N (100% Confidence)

The outcomes of death and hospital admission are objective measures.

4.5 : N (100% Confidence)

There is no clear evidence that assessment of the objective outcomes of death and hospital admission would be influenced by knowledge of the intervention received.

Low risk (100% Confidence)

Based on the objective nature of the outcomes and responses to the signaling questions, this domain is low risk.

Domain 5

5.1 : NI (100% Confidence)

No information is provided on whether the data was analyzed in accordance with a pre-specified plan.

5.2 : N (90% Confidence)

For the objective outcome of hospital admission, there are unlikely to be multiple eligible outcome measurements.

5.3 : N (90% Confidence)

There is unlikely to be multiple eligible analyses for the outcome of hospital admission. The time-to-event and dichotomous outcome analyses are appropriate.

Some concerns (100% Confidence)

With 5.1 NI, 5.2 N, and 5.3 N, this meets the criteria for some concerns.

Overall Bias

High risk (80% Confidence)

With Domain 2 and Domain 5 having some concerns, and no domains being low risk, the overall risk of bias is high per the criteria provided.

## Study 17-1

**Article ID**: Tauber, 2011

**Outcome Name**: Microbiological efficacy rate

Domain 1: Risk of bias arising from the randomization process

1.1: NI (50% confidence)

The study is described as randomized but no information is provided about the method of sequence generation.

1.2: NI (50% confidence)

No information is provided about allocation concealment.

1.3: N (80% confidence)

No significant differences in baseline characteristics between groups are reported.

Result of Domain 1: Some concerns (75% confidence)

The randomization process is not described in detail, but there are no apparent issues with baseline imbalances.

Domain 2: Risk of bias due to deviations from the intended interventions

2.1: N (90% confidence)

The study is described as double-masked, implying participants were unaware of their assigned intervention.

2.2: N (90% confidence)

The study is described as double-masked, implying those delivering interventions were unaware of assignments.

2.3: NI (50% confidence)

No information is provided about deviations from intended interventions.

2.4: NI (50% confidence)

No information is provided to judge if deviations affected the outcome.

2.5: NI (50% confidence)

No information is provided to judge if deviations were balanced between groups.

2.6: Y (90% confidence)

The analysis appears to include all randomized participants.

2.7: N (80% confidence)

There is no indication that participants were analyzed in the wrong groups.

Result of Domain 2: Low risk (80% confidence)

The study was double-masked and appears to have analyzed all participants as randomized.

Domain 3: Risk of bias due to missing outcome data

3.1: Y (90% confidence)

847 out of 1179 randomized patients (72%) had bacterial isolates recovered and were included in the microbiological analysis.

3.2: NI (50% confidence)

No information is provided about methods to handle missing data.

3.3: NI (50% confidence)

No information is provided about reasons for missing data.

3.4: NI (50% confidence)

No information is provided to judge if missingness depended on the true outcome value.

Result of Domain 3: Some concerns (70% confidence)

A substantial proportion of randomized patients were not included in the analysis, but reasons for exclusion are not clear.

Domain 4: Risk of bias in measurement of the outcome

4.1: N (80% confidence)

Standard microbiological methods were used to measure the outcome.

4.2: N (80% confidence)

The same microbiological methods were used for both groups.

4.3: N (90% confidence)

The study was double-masked, implying outcome assessors were unaware of intervention assignments.

4.4: N (80% confidence)

Microbiological eradication is an objective outcome unlikely to be influenced by knowledge of the intervention.

4.5: N (80% confidence)

There is no evidence that assessment was influenced by knowledge of the intervention.

Result of Domain 4: Low risk (85% confidence)

The outcome was objectively measured using standard methods in a double-masked study.

Domain 5: Risk of bias in selection of the reported result

5.1: Y (80% confidence)

The study was registered (NCT00759148), implying a pre-specified analysis plan.

5.2: N (80% confidence)

The reported results correspond to all expected measurements of the outcome.

5.3: N (80% confidence)

The reported results correspond to the only logical analysis approach for this outcome.

Result of Domain 5: Low risk (80% confidence)

The study was pre-registered and the reported results appear appropriate and comprehensive.

Overall Bias Risk for the Article: Some concerns (75% confidence)

While the study was double-masked, pre-registered, and used objective outcome measures, there are some concerns due to lack of detail about the randomization process and handling of missing data.

## Study 17-2

Based on the provided information and instructions, here is my assessment of the risk of bias for the study:

**Article ID**: Tauber, 2011

**Outcome Name**: Mental state

Domain 1: Risk of bias arising from the randomization process

1.1: NI (75% confidence)

The article states patients were randomized in a 1:1 ratio but does not describe the specific method of sequence generation.

1.2: PY (80% confidence)

The study is described as double-masked, suggesting allocation was likely concealed, but specific methods are not described.

1.3: N (90% confidence)

Table 1 shows baseline characteristics were similar between groups, suggesting no major issues with randomization.

Result: Some concerns (80% confidence)

While randomization was performed, key details on sequence generation and allocation concealment are lacking.

Domain 2: Risk of bias due to deviations from intended interventions

2.1: N (90% confidence)

The study is described as double-masked, indicating participants were unaware of their assigned intervention.

2.2: N (90% confidence)

As a double-masked study, those delivering interventions were likely unaware of assignments.

2.3: NI (75% confidence)

No information is provided about deviations from intended interventions.

2.4: NI (75% confidence)

Without information on deviations, their impact cannot be determined.

2.5: NI (75% confidence)

No information to assess if deviations were balanced between groups.

2.6: Y (95% confidence)

Analysis was performed on the intent-to-treat population.

2.7: N (90% confidence)

With ITT analysis, there is low risk of substantial impact from analyzing participants in the wrong group.

Result: Low risk (85% confidence)

The double-masked design and ITT analysis suggest low risk of bias in this domain.

Domain 3: Missing outcome data

3.1: Y (95% confidence)

847 of 1180 randomized patients (71.8%) had bacterial cultures and were included in the MBITT analysis.

3.2: NI (75% confidence)

No information is provided on methods to correct for bias from missing data.

3.3: NI (75% confidence)

Reasons for missing outcome data are not reported.

3.4: NI (75% confidence)

Without information on reasons for missing data, this cannot be determined.

Result: Some concerns (80% confidence)

While a high proportion of randomized patients were analyzed, information on handling of missing data is lacking.

Domain 4: Measurement of the outcome

4.1: N (90% confidence)

Microbiological culture is an appropriate and reliable method for assessing bacterial eradication.

4.2: N (95% confidence)

The methods describe identical procedures for obtaining and processing specimens from both groups.

4.3: N (90% confidence)

As a double-masked study, outcome assessors were likely unaware of treatment assignments.

4.4: N (95% confidence)

Microbiological culture is an objective measure unlikely to be influenced by knowledge of the intervention.

4.5: N (95% confidence)

As an objective measure, assessment of bacterial eradication is unlikely to be influenced by knowledge of the intervention.

Result: Low risk (90% confidence)

The use of objective microbiological outcomes in a double-masked study design suggests low risk of bias.

Domain 5: Selection of the reported result

5.1: Y (90% confidence)

The methods and results sections describe consistent outcome measures and analyses.

5.2: N (90% confidence)

The prespecified microbiological outcomes are reported as described in the methods.

5.3: N (90% confidence)

There is no evidence of selection among multiple analyses.

Result: Low risk (90% confidence)

Reported results appear consistent with the prespecified analysis plan.

Overall Bias Risk for the Article: Some concerns (80% confidence)

While the study design and analysis methods suggest low risk in several domains, key details are lacking regarding the randomization process and handling of missing data. These limitations introduce some concerns about potential bias, though the objective nature of the primary outcome helps mitigate these risks.

## Study 18-1

**Article ID**: Botella-Carretero, 2008

**Outcome Name**: Incidence of pressure ulcers

Domain 1: Risk of bias arising from the randomization process

1.1: NI (90% confidence)

Reason: The article only mentions random grouping without specifying methods for generating random sequences.

1.2: NI (90% confidence)

Reason: The article does not mention allocation concealment methods such as central allocation or opaque envelopes.

1.3: N (80% confidence)

Reason: The article states there were no significant differences in baseline characteristics between groups.

Result of Domain 1: Some concerns (85% confidence)

Reason: The allocation sequence generation and concealment are unclear, but baseline differences do not suggest problems with randomization.

Domain 2: Risk of bias due to deviations from intended interventions

2.1: Y (90% confidence)

Reason: This was an open-label trial, so participants were aware of their assigned intervention.

2.2: Y (90% confidence)

Reason: As an open-label trial, care providers were also aware of participants' assigned interventions.

2.3: NI (80% confidence)

Reason: The article does not report any deviations from intended interventions.

2.4: NI (80% confidence)

Reason: Without information on deviations, their effect on outcomes cannot be determined.

2.5: NI (80% confidence)

Reason: There is no information to assess if any deviations were balanced between groups.

2.6: Y (90% confidence)

Reason: The article states they used intent-to-treat analysis, and the number of participants in the result analysis is consistent with the number randomized.

2.7: N (90% confidence)

Reason: With intent-to-treat analysis, participants were likely analyzed in their assigned groups.

Result of Domain 2: Some concerns (85% confidence)

Reason: While the trial was open-label, appropriate analysis was used and major deviations were not reported.

Domain 3: Risk of bias due to missing outcome data

3.1: Y (95% confidence)

Reason: The article states they used intent-to-treat analysis, indicating that all randomized participants were included in the analysis.

3.2: N (90% confidence)

Reason: The use of intent-to-treat analysis suggests that missing data was not corrected for using methods like multiple imputation.

3.3: PN (80% confidence)

Reason: Reasons for missing data (e.g. transfer to other hospital, medical complications) seem unlikely to be related to pressure ulcer incidence.

3.4: PN (80% confidence)

Reason: No evidence suggests missingness depended on the true outcome value.

Result of Domain 3: Low risk (85% confidence)

Reason: Intent-to-treat analysis was used, and missing data seems unlikely to bias results.

Domain 4: Risk of bias in measurement of the outcome

4.1: NI (80% confidence)

Reason: The article does not specify how pressure ulcers were measured or diagnosed.

4.2: N (85% confidence)

Reason: There is no indication that measurement differed between groups.

4.3: Y (90% confidence)

Reason: In this open-label trial, outcome assessors were likely aware of intervention assignments.

4.4: Y (85% confidence)

Reason: Knowledge of the intervention could influence assessment of pressure ulcers.

4.5: N (80% confidence)

Reason: There is no clear evidence that assessment of the outcome was influenced by knowledge of intervention received.

Result of Domain 4: Some concerns (85% confidence)

Reason: While outcome assessors were likely aware of interventions, there's no clear evidence this influenced assessment.

Domain 5: Risk of bias in selection of the reported result

5.1: NI (80% confidence)

Reason: No information is provided about a pre-specified analysis plan.

5.2: N (85% confidence)

Reason: Pressure ulcers were likely measured in only one way.

5.3: N (85% confidence)

Reason: There is no evidence of selective reporting based on results.

Result of Domain 5: Some concerns (80% confidence)

Reason: While selective reporting seems unlikely, the lack of a pre-specified analysis plan raises some concerns.

Overall Bias Risk for the Article: High risk (85% confidence)

Reason: There are some concerns in multiple domains, particularly related to the open-label design and lack of information on allocation concealment and analysis plans. While no single domain is at high risk, the cumulative concerns across domains result in an overall high risk of bias.

## Study 18-2

Thank you for the additional guidance. I will review the assessment once more, strictly adhering to the instructions and avoiding any subjective judgment:

**Article ID**: Botella-Carretero, 2008

**Outcome Name**: Incidence of pressure ulcers

Domain 1: Risk of bias arising from the randomization process

1.1: NI (95% confidence)

Reason: The article only mentions random grouping without specifying methods for generating random sequences.

1.2: NI (95% confidence)

Reason: The article does not mention allocation concealment methods such as central allocation or envelopes.

1.3: N (90% confidence)

Reason: The article states there were no significant differences in baseline characteristics between groups.

Result of Domain 1: Some concerns (95% confidence)

Reason: The allocation sequence generation and concealment are unclear, but baseline differences do not suggest problems with randomization.

Domain 2: Risk of bias due to deviations from intended interventions

2.1: Y (95% confidence)

Reason: This was an open-label trial, so participants were aware of their assigned intervention.

2.2: Y (95% confidence)

Reason: As an open-label trial, care providers were also aware of participants' assigned interventions.

2.3: NI (90% confidence)

Reason: The article does not report any deviations from intended interventions.

2.4: NI (90% confidence)

Reason: Without information on deviations, their effect on outcomes cannot be determined.

2.5: NI (90% confidence)

Reason: There is no information to assess if any deviations were balanced between groups.

2.6: Y (95% confidence)

Reason: The article states they used intent-to-treat analysis, and the number of participants in the result analysis is consistent with the number randomized.

2.7: N (95% confidence)

Reason: With intent-to-treat analysis, participants were likely analyzed in their assigned groups.

Result of Domain 2: Some concerns (95% confidence)

Reason: While the trial was open-label, appropriate analysis was used and major deviations were not reported.

Domain 3: Risk of bias due to missing outcome data

3.1: Y (95% confidence)

Reason: The article states they used intent-to-treat analysis, indicating that all randomized participants were included in the analysis.

3.2: N (95% confidence)

Reason: The use of intent-to-treat analysis suggests that missing data was not corrected for using methods like multiple imputation.

3.3: PN (90% confidence)

Reason: Reasons for missing data (e.g. transfer to other hospital, medical complications) seem unlikely to be related to pressure ulcer incidence.

3.4: PN (90% confidence)

Reason: No evidence suggests missingness depended on the true outcome value.

Result of Domain 3: Low risk (95% confidence)

Reason: Intent-to-treat analysis was used, and missing data seems unlikely to bias results.

Domain 4: Risk of bias in measurement of the outcome

4.1: NI (90% confidence)

Reason: The article does not specify how pressure ulcers were measured or diagnosed.

4.2: N (90% confidence)

Reason: There is no indication that measurement differed between groups.

4.3: Y (95% confidence)

Reason: In this open-label trial, outcome assessors were likely aware of intervention assignments.

4.4: Y (90% confidence)

Reason: Knowledge of the intervention could influence assessment of pressure ulcers.

4.5: N (90% confidence)

Reason: There is no clear evidence that assessment of the outcome was influenced by knowledge of intervention received.

Result of Domain 4: Some concerns (95% confidence)

Reason: While outcome assessors were likely aware of interventions, there's no clear evidence this influenced assessment.

Domain 5: Risk of bias in selection of the reported result

5.1: NI (90% confidence)

Reason: No information is provided about a pre-specified analysis plan.

5.2: N (90% confidence)

Reason: Pressure ulcers were likely measured in only one way.

5.3: N (90% confidence)

Reason: There is no evidence of selective reporting based on results.

Result of Domain 5: Some concerns (90% confidence)

Reason: While selective reporting seems unlikely, the lack of a pre-specified analysis plan raises some concerns.

Overall Bias Risk for the Article: High risk (95% confidence)

Reason: There are some concerns in multiple domains. Although no single domain is at high risk, the cumulative concerns across domains result in an overall high risk of bias.

## Study 19-1

Here is my revised assessment of the risk of bias for the study by van Anholt et al., 2010:

**Article ID**: van Anholt, 2010

**Outcome Name**: Incidence of pressure ulcers

Domain 1: Risk of bias arising from the randomization process

1.1: NI (95% confidence)

Reason: The article only mentions random allocation without specifying methods for generating random sequences.

1.2: NI (95% confidence)

Reason: The article does not mention allocation concealment methods such as central allocation or envelopes.

1.3: N (90% confidence)

Reason: The article states there were no statistically significant differences in baseline characteristics between groups.

Result of Domain 1: Some concerns (95% confidence)

Reason: The allocation sequence generation and concealment are unclear, but baseline differences do not suggest problems with randomization.

Domain 2: Risk of bias due to deviations from intended interventions

2.1: N (90% confidence)

Reason: The article states this was a double-blind trial, so participants were likely unaware of their assigned intervention.

2.2: N (90% confidence)

Reason: As a double-blind trial, care providers were likely unaware of participants' assigned interventions.

2.3: NI (90% confidence)

Reason: The article does not report any deviations from intended interventions.

2.4: NI (90% confidence)

Reason: Without information on deviations, their effect on outcomes cannot be determined.

2.5: NI (90% confidence)

Reason: There is no information to assess if any deviations were balanced between groups.

2.6: Y (95% confidence)

Reason: The article states they used intention-to-treat analysis, and the number of participants in the result analysis is consistent with the number randomized.

2.7: N (95% confidence)

Reason: With intention-to-treat analysis, participants were likely analyzed in their assigned groups.

Result of Domain 2: Low risk (90% confidence)

Reason: The trial was double-blind and used appropriate analysis. Major deviations were not reported.

Domain 3: Risk of bias due to missing outcome data

3.1: Y (95% confidence)

Reason: The article states they used intention-to-treat analysis, indicating that all randomized participants were included in the analysis.

3.2: N (95% confidence)

Reason: The use of intention-to-treat analysis suggests that missing data was not corrected for using methods like multiple imputation.

3.3: PN (90% confidence)

Reason: Reasons for missing data (e.g. death, hospitalization) seem unlikely to be related to pressure ulcer incidence.

3.4: PN (90% confidence)

Reason: No evidence suggests missingness depended on the true outcome value.

Result of Domain 3: Low risk (90% confidence)

Reason: Intention-to-treat analysis was used, and missing data seems unlikely to bias results.

Domain 4: Risk of bias in measurement of the outcome

4.1: N (90% confidence)

Reason: The article specifies that pressure ulcer size was measured by length and width using a ruler.

4.2: N (90% confidence)

Reason: There is no indication that measurement differed between groups.

4.3: N (95% confidence)

Reason: In this double-blind trial, outcome assessors were likely unaware of intervention assignments.

4.4: Y (90% confidence)

Reason: Knowledge of the intervention could influence assessment of pressure ulcers.

4.5: N (90% confidence)

Reason: There is no clear evidence that assessment of the outcome was influenced by knowledge of intervention received.

Result of Domain 4: Low risk (90% confidence)

Reason: The outcome was measured objectively and assessors were likely blinded to intervention status.

Domain 5: Risk of bias in selection of the reported result

5.1: Y (90% confidence)

Reason: The article mentions a predefined statistical plan.

5.2: N (90% confidence)

Reason: Pressure ulcers were measured in only one way.

5.3: N (90% confidence)

Reason: There is no evidence of selective reporting based on results.

Result of Domain 5: Low risk (90% confidence)

Reason: There was a pre-specified analysis plan and no evidence of selective reporting.

Overall Bias Risk for the Article: Low risk (90% confidence)

Reason: Most domains were assessed as low risk, with some concerns only in the randomization domain. The double-blind design and appropriate analysis methods reduce the overall risk of bias.

## Study 19-2

Here is my revised assessment of the risk of bias for the study by van Anholt et al., 2010:

**Article ID**: van Anholt, 2010

**Outcome Name**: Incidence of pressure ulcers

Domain 1: Risk of bias arising from the randomization process

1.1: NI (95% confidence)

Reason: The article only mentions random allocation without specifying methods for generating random sequences.

1.2: NI (95% confidence)

Reason: The article does not mention allocation concealment methods such as central allocation or envelopes.

1.3: N (90% confidence)

Reason: The article states there were no statistically significant differences in baseline characteristics between groups.

Result of Domain 1: Some concerns (95% confidence)

Reason: The allocation sequence generation and concealment are unclear, but baseline differences do not suggest problems with randomization.

Domain 2: Risk of bias due to deviations from intended interventions

2.1: N (95% confidence)

Reason: The article states this was a double-blind trial, so participants were unaware of their assigned intervention.

2.2: N (95% confidence)

Reason: As a double-blind trial, care providers were unaware of participants' assigned interventions.

2.3: NI (90% confidence)

Reason: The article does not report any deviations from intended interventions.

2.4: NI (90% confidence)

Reason: Without information on deviations, their effect on outcomes cannot be determined.

2.5: NI (90% confidence)

Reason: There is no information to assess if any deviations were balanced between groups.

2.6: Y (95% confidence)

Reason: The article states they used intention-to-treat analysis, and the number of participants in the result analysis is consistent with the number randomized.

2.7: N (95% confidence)

Reason: With intention-to-treat analysis, participants were analyzed in their assigned groups.

Result of Domain 2: Low risk (95% confidence)

Reason: The trial was double-blind and used appropriate analysis. Major deviations were not reported.

Domain 3: Risk of bias due to missing outcome data

3.1: Y (95% confidence)

Reason: The article states they used intention-to-treat analysis, indicating that all 43 randomized participants were included in the analysis.

3.2: N (95% confidence)

Reason: The use of intention-to-treat analysis suggests that missing data was not corrected for using methods like multiple imputation.

3.3: PN (90% confidence)

Reason: Reasons for missing data (e.g. death, hospitalization) seem unlikely to be related to pressure ulcer incidence.

3.4: PN (90% confidence)

Reason: No evidence suggests missingness depended on the true outcome value.

Result of Domain 3: Low risk (95% confidence)

Reason: Intention-to-treat analysis was used, and missing data seems unlikely to bias results.

Domain 4: Risk of bias in measurement of the outcome

4.1: N (95% confidence)

Reason: The article specifies that pressure ulcer size was measured by length and width using a ruler, which is an appropriate method.

4.2: N (95% confidence)

Reason: There is no indication that measurement differed between groups.

4.3: N (95% confidence)

Reason: In this double-blind trial, outcome assessors were unaware of intervention assignments.

4.4: Y (90% confidence)

Reason: Knowledge of the intervention could influence assessment of pressure ulcers.

4.5: N (90% confidence)

Reason: There is no clear evidence that assessment of the outcome was influenced by knowledge of intervention received.

Result of Domain 4: Low risk (95% confidence)

Reason: The outcome was measured objectively and assessors were blinded to intervention status.

Domain 5: Risk of bias in selection of the reported result

5.1: Y (95% confidence)

Reason: The article mentions analyses were performed according to a predefined statistical plan.

5.2: N (95% confidence)

Reason: Pressure ulcers were measured in only one way as specified in the methods.

5.3: N (95% confidence)

Reason: There is no evidence of selective reporting based on results.

Result of Domain 5: Low risk (95% confidence)

Reason: There was a pre-specified analysis plan and no evidence of selective reporting.

Overall Bias Risk for the Article: Low risk (95% confidence)

Reason: Most domains were assessed as low risk, with some concerns only in the randomization domain. The double-blind design and appropriate analysis methods reduce the overall risk of bias.

## Study 20-1

Article ID: González-Ortega, 2021

Outcome Name: Mental state

Domain 1: Risk of bias arising from the randomization process

1.1: Was the allocation sequence random? Y (95% confidence)

Reason: The article states "After signing informed consent, the patients were randomised to one of the treatment groups (TAU or combined clinical treatment) using random allocation software."

1.2: Was the allocation sequence concealed until participants were enrolled and assigned to interventions? PY (80% confidence)

Reason: The article mentions "The allocation sequence was designed by an independent person not otherwise involved in the trial." This suggests some concealment, but full details are not provided.

1.3: Were there baseline imbalances that suggest a problem with the randomization process? N (90% confidence)

Reason: The article states "There were no clinical or functional differences at baseline between the CBT+TAU and the TAU groups."

Result of Domain 1: Low risk of bias (90% confidence)

Reason: The randomization process appears to have been properly implemented with no major issues identified.

Domain 2: Risk of bias due to deviations from intended interventions

2.1: Were participants aware of their assigned intervention during the trial? Y (95% confidence)

Reason: Given the nature of the interventions (CBT+TAU vs TAU alone), participants would have been aware of their group assignment.

2.2: Were carers and people delivering the interventions aware of participants' assigned intervention during the trial? Y (95% confidence)

Reason: The therapists delivering the interventions would have known which treatment they were providing.

2.3: Were there deviations from the intended intervention that arose because of the experimental context? PN (70% confidence)

Reason: No deviations are reported, and the article states "TAU was provided in all centres, according to the standard procedures of the Spanish National Health Service for FEP patients."

2.4: Were these deviations likely to have affected the outcome? NI (50% confidence)

Reason: No information is provided about potential effects of any deviations.

2.5: Were these deviations from intended intervention balanced between groups? NI (50% confidence)

Reason: No information is provided about balance of any deviations between groups.

2.6: Was an appropriate analysis used to estimate the effect of assignment to intervention? Y (90% confidence)

Reason: The article reports using ANCOVA to analyze outcomes, adjusting for baseline scores.

2.7: Was there potential for a substantial impact (on the result) of the failure to analyze participants in the group to which they were randomized? N (85% confidence)

Reason: The article states only 7 out of 184 participants were excluded from analysis, which is unlikely to substantially impact results.

Result of Domain 2: Some concerns (75% confidence)

Reason: While participants and providers were aware of interventions, appropriate analysis methods were used and there is no clear evidence of problematic deviations from intended interventions.

Domain 3: Missing outcome data

3.1: Were data for this outcome available for all, or nearly all, participants randomized? Y (95% confidence)

Reason: Only 7 out of 184 participants (3.8%) were excluded from analysis.

3.2: Is there evidence that the result was not biased by missing outcome data? Y (85% confidence)

Reason: The low rate of missing data (3.8%) is unlikely to substantially bias results.

3.3: Could missingness in the outcome depend on its true value? PN (70% confidence)

Reason: The reasons given for exclusion (not attending all sessions or post-treatment assessment) do not seem clearly related to the outcome.

3.4: Is it likely that missingness in the outcome depended on its true value? PN (70% confidence)

Reason: As above, the reasons for missingness do not appear strongly related to the outcome.

Result of Domain 3: Low risk of bias (85% confidence)

Reason: There was very little missing outcome data and no strong indication it was related to the true outcome.

Domain 4: Risk of bias in measurement of the outcome

4.1: Was the method of measuring the outcome inappropriate? N (90% confidence)

Reason: The study used established scales like PANSS, HDRS, and STAI to measure mental state outcomes.

4.2: Could measurement or ascertainment of the outcome have differed between intervention groups? N (85% confidence)

Reason: The same measurement methods were used for both groups at the same timepoints.

4.3: Were outcome assessors aware of the intervention received by study participants? N (85% confidence)

Reason: The article states "The assessment was thus carried out by a researcher who was blind to the patient allocation process."

4.4: Could assessment of the outcome have been influenced by knowledge of intervention received? PN (75% confidence)

Reason: Assessors were blinded, but some outcomes like HDRS involve clinical judgment which could potentially be influenced.

4.5: Is it likely that assessment of the outcome was influenced by knowledge of intervention received? PN (70% confidence)

Reason: While possible, there is no specific evidence that blinded outcome assessment was compromised.

Result of Domain 4: Low risk of bias (80% confidence)

Reason: Appropriate outcome measures were used, assessors were blinded, and there is no clear evidence of bias in outcome measurement.

Domain 5: Risk of bias in selection of the reported result

5.1: Were the data that produced this result analyzed in accordance with a pre-specified analysis plan that was finalized before unblinded outcome data were available for analysis? PY (75% confidence)

Reason: While a full pre-specified analysis plan is not provided, the methods section outlines the planned analyses.

5.2: Is the numerical result being assessed likely to have been selected, on the basis of the results, from multiple eligible outcome measurements within the outcome domain? N (80% confidence)

Reason: The reported outcomes align with those described in the methods, with no evidence of selective reporting.

5.3: Is the numerical result being assessed likely to have been selected, on the basis of the results, from multiple eligible analyses of the data? N (80% confidence)

Reason: The analyses reported align with those described in the methods section, with no evidence of selective analysis reporting.

Result of Domain 5: Low risk of bias (80% confidence)

Reason: There is no clear evidence of selective outcome or analysis reporting.

Overall risk of bias: Some concerns (75% confidence)

Reason: While most domains show low risk of bias, there are some concerns about potential bias due to awareness of interventions by participants and providers. However, the use of blinded outcome assessment and appropriate analysis methods mitigate these concerns to some extent.

## Study 20-2

Thank you for providing the full article. I have carefully reviewed it and will now assess the risk of bias for the mental state outcome as requested. Here is my evaluation:

Article ID: González-Ortega, 2021

Outcome Name: Mental state

Domain 1: Risk of bias arising from the randomization process

1.1: Was the allocation sequence random? Y (95% confidence)

Reason: The article states "After signing informed consent, the patients were randomised to one of the treatment groups (TAU or combined clinical treatment) using random allocation software."

1.2: Was the allocation sequence concealed until participants were enrolled and assigned to interventions? PY (80% confidence)

Reason: The article mentions "The allocation sequence was designed by an independent person not otherwise involved in the trial." This suggests some concealment, but full details are not provided.

1.3: Were there baseline imbalances that suggest a problem with the randomization process? N (90% confidence)

Reason: The article states "There were no clinical or functional differences at baseline between the CBT+TAU and the TAU groups."

Result of Domain 1: Low risk of bias (90% confidence)

Reason: The randomization process appears to have been properly implemented with no major issues identified.

Domain 2: Risk of bias due to deviations from intended interventions

2.1: Were participants aware of their assigned intervention during the trial? Y (95% confidence)

Reason: Given the nature of the interventions (CBT+TAU vs TAU alone), participants would have been aware of their group assignment.

2.2: Were carers and people delivering the interventions aware of participants' assigned intervention during the trial? Y (95% confidence)

Reason: The therapists delivering the interventions would have known which treatment they were providing.

2.3: Were there deviations from the intended intervention that arose because of the experimental context? PN (70% confidence)

Reason: No deviations are reported, and the article states "TAU was provided in all centres, according to the standard procedures of the Spanish National Health Service for FEP patients."

2.4: Were these deviations likely to have affected the outcome? NI (50% confidence)

Reason: No information is provided about potential effects of any deviations.

2.5: Were these deviations from intended intervention balanced between groups? NI (50% confidence)

Reason: No information is provided about balance of any deviations between groups.

2.6: Was an appropriate analysis used to estimate the effect of assignment to intervention? Y (90% confidence)

Reason: The article reports using ANCOVA to analyze outcomes, adjusting for baseline scores.

2.7: Was there potential for a substantial impact (on the result) of the failure to analyze participants in the group to which they were randomized? N (85% confidence)

Reason: The article states only 7 out of 184 participants were excluded from analysis, which is unlikely to substantially impact results.

Result of Domain 2: Some concerns (75% confidence)

Reason: While participants and providers were aware of interventions, appropriate analysis methods were used and there is no clear evidence of problematic deviations from intended interventions.

Domain 3: Missing outcome data

3.1: Were data for this outcome available for all, or nearly all, participants randomized? Y (95% confidence)

Reason: Only 7 out of 184 participants (3.8%) were excluded from analysis.

3.2: Is there evidence that the result was not biased by missing outcome data? Y (85% confidence)

Reason: The low rate of missing data (3.8%) is unlikely to substantially bias results.

3.3: Could missingness in the outcome depend on its true value? PN (70% confidence)

Reason: The reasons given for exclusion (not attending all sessions or post-treatment assessment) do not seem clearly related to the outcome.

3.4: Is it likely that missingness in the outcome depended on its true value? PN (70% confidence)

Reason: As above, the reasons for missingness do not appear strongly related to the outcome.

Result of Domain 3: Low risk of bias (85% confidence)

Reason: There was very little missing outcome data and no strong indication it was related to the true outcome.

Domain 4: Risk of bias in measurement of the outcome

4.1: Was the method of measuring the outcome inappropriate? N (90% confidence)

Reason: The study used established scales like PANSS, HDRS, and STAI to measure mental state outcomes.

4.2: Could measurement or ascertainment of the outcome have differed between intervention groups? N (85% confidence)

Reason: The same measurement methods were used for both groups at the same timepoints.

4.3: Were outcome assessors aware of the intervention received by study participants? N (85% confidence)

Reason: The article states "The assessment was thus carried out by a researcher who was blind to the patient allocation process."

4.4: Could assessment of the outcome have been influenced by knowledge of intervention received? PN (75% confidence)

Reason: Assessors were blinded, but some outcomes like HDRS involve clinical judgment which could potentially be influenced.

4.5: Is it likely that assessment of the outcome was influenced by knowledge of intervention received? PN (70% confidence)

Reason: While possible, there is no specific evidence that blinded outcome assessment was compromised.

Result of Domain 4: Low risk of bias (80% confidence)

Reason: Appropriate outcome measures were used, assessors were blinded, and there is no clear evidence of bias in outcome measurement.

Domain 5: Risk of bias in selection of the reported result

5.1: Were the data that produced this result analyzed in accordance with a pre-specified analysis plan that was finalized before unblinded outcome data were available for analysis? PY (75% confidence)

Reason: While a full pre-specified analysis plan is not provided, the methods section outlines the planned analyses.

5.2: Is the numerical result being assessed likely to have been selected, on the basis of the results, from multiple eligible outcome measurements within the outcome domain? N (80% confidence)

Reason: The reported outcomes align with those described in the methods, with no evidence of selective reporting.

5.3: Is the numerical result being assessed likely to have been selected, on the basis of the results, from multiple eligible analyses of the data? N (80% confidence)

Reason: The analyses reported align with those described in the methods section, with no evidence of selective analysis reporting.

Result of Domain 5: Low risk of bias (80% confidence)

Reason: There is no clear evidence of selective outcome or analysis reporting.

Overall risk of bias: Some concerns (75% confidence)

Reason: While most domains show low risk of bias, there are some concerns about potential bias due to awareness of interventions by participants and providers. However, the use of blinded outcome assessment and appropriate analysis methods mitigate these concerns to some extent.

Study 21-1

Thank you for the additional clarification. I'll review the article again and make sure my evaluation strictly adheres to these instructions.

**Article ID**: Bischoff, 2012

**Outcome Name**: Health-related quality of life (measured by N score)

**Registration number**: NCT00128765

Domain 1

1.1: PY (95% confidence)

Reason: The article mentions "We randomised participants by using a computer generated two block randomisation procedure with stratification..."

1.2: NI (95% confidence)

Reason: The article does not mention central allocation, envelopes, or postcards for allocation concealment.

1.3: N (90% confidence)

Reason: Table 1 shows baseline characteristics were well balanced between groups, except for sex. This minor imbalance is likely due to chance rather than a problem with randomization.

Result of the domain: Some concerns (95% confidence)

Reason: While the randomization method is described, allocation concealment is unclear.

Domain 2

2.1: Y (95% confidence)

Reason: Patients were aware of their group assignment, as the interventions could not be blinded.

2.2: Y (95% confidence)

Reason: Practice nurses delivered the interventions and were aware of group assignments.

2.3: NA (95% confidence)

Reason: The article does not mention any non-protocol interventions.

2.4: Y (90% confidence)

Reason: In the self-management group, 13% of patients did not receive any session or telephone contact, which is >10%.

2.5: Y (90% confidence)

Reason: In the routine monitoring group, 11% of patients did not receive any consultation, which is >10%.

2.6: N (95% confidence)

Reason: The analysis was intention-to-treat, which is inappropriate for evaluating compliance.

Result of the domain: High risk (95% confidence)

Reason: Participants and providers were aware of assignments, >10% had implementation failures or non-adherence, and an appropriate analysis was not used to estimate the effect of adhering to the intervention.

Domain 3

3.1: PN (90% confidence)

Reason: About 16% of participants dropped out, which is between 5-10% for the continuous outcome.

3.2: N (95% confidence)

Reason: No methods to handle missing data are described.

3.3: PY (80% confidence)

Reason: Missingness may depend on the true value, as participants with worse health status may be more likely to drop out.

3.4: PN (75% confidence)

Reason: While missingness may be related to the outcome, there is no clear evidence it is likely.

Result of the domain: Some concerns (85% confidence)

Reason: There was some missing data that was not appropriately handled, but it likely did not substantially bias the results.

Domain 4

4.1: N (95% confidence)

Reason: The Chronic Respiratory Questionnaire is a validated measure for COPD-specific quality of life.

4.2: N (90% confidence)

Reason: The outcome was measured consistently across groups.

4.3: Y (95% confidence)

Reason: This is a patient-reported outcome and patients were aware of their assignments.

4.4: Y (95% confidence)

Reason: Quality of life is a subjective measure that could be influenced by knowledge of the intervention received.

4.5: N (90% confidence)

Reason: There is no clear evidence that assessment was influenced by knowledge of the intervention.

Result of the domain: Some concerns (90% confidence)

Reason: The outcome measure was appropriate and consistent, but knowledge of the intervention could have influenced patient reporting.

Domain 5

5.1: NI (95% confidence)

Reason: No information is provided about a pre-specified analysis plan or trial registration.

5.2: N (85% confidence)

Reason: The reported results align with the outcomes and measures described in the methods.

5.3: N (85% confidence)

Reason: The analyses described in the methods align with those reported in the results.

Result of the domain: Some concerns (90% confidence)

Reason: While the reported analyses seem appropriate, it is unclear if they were pre-specified.

Overall Bias Risk for the Article: High risk (95% confidence)

Reason: There are some concerns across multiple domains, but the most significant issue is the high risk of bias in Domain 2 due to lack of blinding, substantial implementation failures/non-adherence, and inappropriate analysis methods.

Study 21-2

Here is my assessment of the risk of bias for the Bischoff 2012 study:

**Article ID**: Bischoff, 2012

**Outcome Name**: Health-related quality of life (CRQ total score)

**Registration number**: NCT00128765

Domain 1: Risk of bias arising from the randomization process

1.1: Y (90% confidence)

Reason: The study used a "computer generated two block randomisation procedure", which is an adequate method of generating a random sequence.

1.2: NI (70% confidence)

Reason: The article does not provide details on allocation concealment methods.

1.3: N (80% confidence)

Reason: Baseline characteristics were generally well balanced between groups, with only minor differences noted.

Result of Domain 1: Some concerns (75% confidence)

Reason: While randomization was adequate and baseline characteristics were balanced, allocation concealment was not described.

Domain 2: Risk of bias due to deviations from the intended interventions

2.1: Y (90% confidence)

Reason: Participants were aware of their intervention group as blinding was not possible given the nature of the interventions.

2.2: Y (90% confidence)

Reason: Practice nurses delivering the interventions were aware of participants' assigned groups.

2.3: NA (80% confidence)

Reason: The article does not mention any important non-protocol interventions.

2.4: N (70% confidence)

Reason: The interventions appear to have been implemented as intended based on the process evaluation reported.

2.5: N (70% confidence)

Reason: There is no clear evidence of substantial non-adherence to the interventions.

2.6: N (80% confidence)

Reason: The analysis was intention-to-treat, which is not an appropriate method to estimate the effect of adhering to the intervention.

Result of Domain 2: High risk (80% confidence)

Reason: Participants and personnel were aware of intervention assignments, and an inappropriate analysis method was used to estimate the effect of adherence.

Domain 3: Missing outcome data

3.1: N (90% confidence)

Reason: Only 139 out of 165 randomized participants (84%) completed the 24-month follow-up.

3.2: N (80% confidence)

Reason: No information is provided on methods to correct for bias due to missing data.

3.3: PN (70% confidence)

Reason: The article states that baseline characteristics did not differ between dropouts and participants who finished follow-up.

3.4: PN (70% confidence)

Reason: As noted above, dropouts appeared similar to completers in baseline characteristics.

Result of Domain 3: Some concerns (75% confidence)

Reason: There was a moderate amount of missing data, but dropouts appeared similar to completers.

Domain 4: Risk of bias in measurement of the outcome

4.1: N (80% confidence)

Reason: The study used the validated chronic respiratory questionnaire to measure health-related quality of life.

4.2: N (80% confidence)

Reason: The outcome measures appear to have been applied consistently across groups.

4.3: N (90% confidence)

Reason: The article states that outcome assessors were blinded to group assignments.

4.4: Y (80% confidence)

Reason: Quality of life is a subjective measure that could potentially be influenced by knowledge of the intervention received.

4.5: N (70% confidence)

Reason: While the outcome is subjective, there is no clear evidence that assessment was actually influenced by knowledge of the intervention.

Result of Domain 4: Low risk (80% confidence)

Reason: The outcome was measured using a validated tool by blinded assessors, and there is no clear evidence of bias in the measurement.

Domain 5: Risk of bias in selection of the reported result

5.1: Y (80% confidence)

Reason: The trial was registered (NCT00128765) and the reported outcomes match those pre-specified.

5.2: N (70% confidence)

Reason: The reported results appear to correspond to all intended outcome measurements described in the methods.

5.3: N (70% confidence)

Reason: There is no clear evidence of selective reporting based on the results.

Result of Domain 5: Low risk (75% confidence)

Reason: The study was pre-registered and reported on all pre-specified outcomes without evidence of selective reporting.

Overall Bias Risk for the Article: High risk (80% confidence)

The overall assessment is high risk of bias, primarily due to the open-label design and use of inappropriate analysis methods to estimate the effect of adherence in Domain 2. While some domains have only some concerns or low risk, the high risk of bias in Domain 2 leads to an overall high risk assessment.

Study 22-1

Here is my assessment of the risk of bias for the Coultas 2005 study:

**Article ID**: Coultas, 2005

**Outcome Name**: Health-related quality of life

**Registration number**: Not reported

Domain 1: Risk of bias arising from the randomization process

1.1: NI (70% confidence)

Reason: The article only mentions random allocation without describing specific methods for generating the random sequence.

1.2: NI (75% confidence)

Reason: The article does not mention any allocation concealment methods.

1.3: N (80% confidence)

Reason: Table 2 shows baseline characteristics were generally similar between groups, with only minor differences in gender and one social support domain.

Result of Domain 1: Some concerns (75% confidence)

Reason: While baseline characteristics were mostly balanced, the randomization and allocation concealment processes were not clearly described.

Domain 2: Risk of bias due to deviations from the intended interventions

2.1: Y (90% confidence)

Reason: Participants were aware of their intervention group as this was an open-label study comparing usual care to nurse-assisted interventions.

2.2: Y (90% confidence)

Reason: The nurses delivering the interventions were aware of participants' assigned groups.

2.3: NI (80% confidence)

Reason: The article does not mention any important non-protocol interventions.

2.4: N (70% confidence)

Reason: Table 1 indicates the interventions were geNnerally implemented as intended.

2.5: NI (70% confidence)

Reason: The article does not report significant non-adherence issues.

2.6: N (80% confidence)

Reason: The analysis appears to be intention-to-treat, which is not an appropriate method to estimate the effect of adhering to the intervention.

Result of Domain 2: High risk (80% confidence)

Reason: Participants and personnel were aware of intervention assignments, and an inappropriate analysis method was used.

Domain 3: Missing outcome data

3.1: N (90% confidence)

Reason: Figure 1 shows only 69.6% of randomized participants completed the study.

3.2: N (80% confidence)

Reason: No information is provided on methods to correct for bias due to missing data.

3.3: PY (70% confidence)

Reason: Patients who dropped out had more severe airflow obstruction and lower quality of life, suggesting missingness may depend on true values.

3.4: PY (70% confidence)

Reason: As noted above, dropout was associated with disease severity and quality of life.

Result of Domain 3: High risk (80% confidence)

Reason: There was substantial missing data that likely depended on the true outcome values.

Domain 4: Risk of bias in measurement of the outcome

4.1: N (80% confidence)

Reason: The study used validated questionnaires to measure health-related quality of life.

4.2: N (80% confidence)

Reason: The outcome measures appear to have been applied consistently across groups.

4.3: Y (90% confidence)

Reason: Quality of life was self-reported by participants who were aware of their intervention group.

4.4: Y (90% confidence)

Reason: Quality of life is a subjective measure that could be influenced by knowledge of the intervention received.

4.5: N (70% confidence)

Reason: While the outcome is subjective, there is no clear evidence that assessment was actually influenced by knowledge of the intervention.

Result of Domain 4: Some concerns (75% confidence)

Reason: While appropriate measures were used, the subjective nature of the outcome and participants' awareness of their intervention group creates some risk of bias.

Domain 5: Risk of bias in selection of the reported result

5.1: NI (80% confidence)

Reason: The article does not mention a pre-specified analysis plan or trial registration.

5.2: N (70% confidence)

Reason: The reported results appear to correspond to the planned outcome measures described in the methods.

5.3: N (70% confidence)

Reason: There is no clear evidence of selective reporting based on the results.

Result of Domain 5: Some concerns (75% confidence)

Reason: While there are no clear signs of selective reporting, the lack of a pre-specified analysis plan creates some uncertainty.

Overall Bias Risk for the Article: High risk (80% confidence)

The overall assessment is high risk of bias, primarily due to the open-label design, substantial missing data that was likely related to the true outcomes, and use of subjective self-reported outcomes. While some domains have only some concerns, the high risk of bias in domains 2 and 3 leads to an overall high risk assessment.

Study 22-2

Here is my assessment of the risk of bias for the Coultas 2005 study:

**Article ID**: Coultas, 2005

**Outcome Name**: Health-related quality of life

**Registration number**: Not reported

Domain 1: Risk of bias arising from the randomization process

1.1: NI (75% confidence)

Reason: The article only mentions random grouping without describing methods for generating random sequences.

1.2: NI (75% confidence)

Reason: The article does not mention allocation concealment.

1.3: N (80% confidence)

Reason: There were no significant differences between groups in baseline characteristics, except for gender distribution.

Result of Domain 1: Some concerns (75% confidence)

Reason: Allocation concealment was not described, but baseline characteristics were generally balanced between groups.

Domain 2: Risk of bias due to deviations from intended interventions

2.1: Y (90% confidence)

Reason: Patients were aware of their group assignment as this was an open-label study.

2.2: Y (90% confidence)

Reason: Nurses delivering the interventions were aware of patients' group assignments.

2.3: NI (70% confidence)

Reason: The article does not provide information on non-protocol interventions.

2.4: N (80% confidence)

Reason: There is no indication of substantial implementation failures.

2.5: NI (80% confidence)

Reason: The article does not report that more than 10% of participants had non-adherence to the assigned intervention.

2.6: N (90% confidence)

Reason: The analysis used was intention-to-treat, which is considered inappropriate for evaluating compliance effects.

Result of Domain 2: Some concerns (80% confidence)

Reason: Participants and personnel were aware of intervention assignments, but no major deviations from intended interventions were reported.

Domain 3: Risk of bias due to missing outcome data

3.1: N (90% confidence)

Reason: Only 69.6% of randomized participants completed the study, which is less than 90%.

3.2: N (80% confidence)

Reason: No analysis methods to correct for bias from missing data were reported.

3.3: PY (70% confidence)

Reason: Patients who dropped out had more severe airflow obstruction and lower quality of life, suggesting missingness may depend on true values.

3.4: PY (70% confidence)

Reason: The reasons for missing data suggest a possible relationship with the outcome.

Result of Domain 3: High risk (80% confidence)

Reason: There was substantial missing data that likely depended on the true outcome values.

Domain 4: Risk of bias in measurement of the outcome

4.1: N (90% confidence)

Reason: Validated quality of life measures (SF-36, SGRQ) were used.

4.2: N (90% confidence)

Reason: The same outcome measures were used for all groups.

4.3: Y (90% confidence)

Reason: For patient-reported outcomes, participants were aware of their intervention.

4.4: Y (90% confidence)

Reason: Quality of life is a subjective measure reported by patients who were aware of their treatment.

4.5: N (70% confidence)

Reason: There is no clear evidence that assessment was influenced by knowledge of the intervention.

Result of Domain 4: Some concerns (80% confidence)

Reason: While appropriate measures were used consistently, the subjective nature of the outcomes and participants' awareness of treatment could introduce some bias.

Domain 5: Risk of bias in selection of the reported result

5.1: NI (80% confidence)

Reason: The article does not mention pre-registration or a pre-specified analysis plan.

5.2: N (80% confidence)

Reason: All expected outcomes appear to be reported.

5.3: N (80% confidence)

Reason: There is no evidence of selective reporting based on results.

Result of Domain 5: Some concerns (80% confidence)

Reason: While there's no clear evidence of selective reporting, the lack of a pre-specified analysis plan raises some concerns.

Overall Bias Risk for the Article: High risk (80% confidence)

The overall risk of bias is high, primarily due to the high risk of bias from missing outcome data. There are also some concerns in all other domains, particularly regarding the lack of information on randomization procedures and allocation concealment, as well as the open-label nature of the study affecting outcome measurement.

Study 23-1

Here is my assessment of the risk of bias for the outcome "Physical activity measured by the IPAQ (moderate)" in the Curran 2022 study:

Article ID: Curran, 2022

Outcome Name: Physical activity measured by the IPAQ (moderate)

Registration number: NCT03672058

Domain 1: Risk of bias arising from the randomization process

1.1: Y (High Certainty - 95%)

Reason: The study states "a researcher independent of the recruitment process (MC) will complete the first random allocation using a sealed opaque envelope", which indicates an adequate randomization method was used.

1.2: Y (High Certainty - 95%)

Reason: The study mentions using "sealed opaque envelope" for allocation concealment, which is an appropriate method.

1.3: N (High Certainty - 95%)

Reason: The study reports baseline characteristics in Table 1 and states "There were no significant differences in any baseline characteristics between the groups, apart from self-reported shortness of breath (SOB) whereby the INT group had significantly higher SOB than the AC group."

Result of Domain 1: Low risk (High Certainty - 95%)

Reason: Adequate randomization and allocation concealment methods were used, and there were no major baseline differences between groups suggesting problems with the randomization process.

Domain 2: Risk of bias due to deviations from the intended interventions

2.1: Y (High Certainty - 95%)

Reason: Participants were aware of their assigned intervention as they received either a Fitbit with feedback or just a Fitbit.

2.2: Y (High Certainty - 95%)

Reason: The physiotherapists providing the intervention were aware of participants' assignments.

2.3: NI (High Certainty - 95%)

Reason: The article does not provide information about non-protocol interventions.

2.4: N (High Certainty - 95%)

Reason: The article states "There were no adverse events reported by either group throughout the study duration", suggesting no major implementation failures.

2.5: N (High Certainty - 95%)

Reason: The study reports high compliance with step count goals (75%) in the intervention group, suggesting no major non-adherence issues.

2.6: N (High Certainty - 95%)

Reason: The primary analysis was not designed to estimate the effect of adhering to the intervention. The study used intention-to-treat principles.

Result of Domain 2: Some concerns (High Certainty - 95%)

Reason: Participants and personnel were aware of assigned interventions, but there is no evidence of major deviations from intended interventions beyond what would be expected in usual practice.

Domain 3: Missing outcome data

3.1: Y (Moderate Certainty - 80%)

Reason: While exact numbers are not provided, Figure 1 shows that all randomized participants (except 1 withdrawal) were analyzed, suggesting nearly complete outcome data.

3.2: N (High Certainty - 95%)

Reason: The article does not mention using methods that can correct for bias due to missing data.

3.3: PN (Moderate Certainty - 80%)

Reason: While not explicitly stated, it seems unlikely that missingness would depend on the true value of physical activity, given the nature of the intervention and outcome.

3.4: N (Moderate Certainty - 80%)

Reason: There is no indication that missingness in the outcome depended on its true value.

Result of Domain 3: Low risk (Moderate Certainty - 80%)

Reason: There appears to be little missing outcome data, and what is missing is unlikely to depend on its true value.

Domain 4: Risk of bias in measurement of the outcome

4.1: N (High Certainty - 95%)

Reason: The IPAQ is a validated measure for physical activity.

4.2: N (High Certainty - 95%)

Reason: The measurement method (IPAQ) should be comparable between groups.

4.3: Y (High Certainty - 95%)

Reason: The IPAQ is a self-reported measure, and participants were aware of their intervention.

4.4: Y (High Certainty - 95%)

Reason: Self-reported physical activity is a subjective measure that could be influenced by knowledge of the intervention received.

4.5: PY (Moderate Certainty - 80%)

Reason: While there's no clear evidence, it's plausible that self-reported physical activity could be influenced by participants' knowledge of receiving an intervention aimed at increasing physical activity.

Result of Domain 4: High risk (High Certainty - 95%)

Reason: The outcome is subjectively measured by participants who were aware of their intervention, which could influence their reporting.

Domain 5: Risk of bias in selection of the reported result

5.1: Y (High Certainty - 95%)

Reason: The study was pre-registered (NCT03672058) and the analysis appears to follow the pre-specified plan.

5.2: N (High Certainty - 95%)

Reason: There is no evidence of multiple outcome measurements or analyses for this outcome.

5.3: N (High Certainty - 95%)

Reason: There is no evidence of multiple analyses of the data for this outcome.

Result of Domain 5: Low risk (High Certainty - 95%)

Reason: The study was pre-registered and there's no evidence of selective reporting.

Overall Bias Risk for the Article: High risk (High Certainty - 95%)

Reason: While randomization, handling of missing data, and selection of reported results appear appropriate, there are some concerns about deviations from intended interventions. The high risk of bias in measurement of the outcome (due to its subjective nature and participants' awareness of the intervention) leads to an overall high risk of bias assessment.

Study 23-2

Here is my assessment of the risk of bias for the Curran 2022 study:

Article ID: Curran, 2022

Outcome Name: Physical activity measured by the IPAQ (moderate)

Registration number: NCT03672058

Domain 1: Risk of bias arising from the randomization process

1.1: Y (High certainty - 95%)

Reason: The study states "a researcher independent of the recruitment process (MC) will complete the first random allocation using a sealed opaque envelope."

1.2: Y (High certainty - 95%)

Reason: The study mentions using "sealed opaque envelope" for allocation concealment.

1.3: N (High certainty - 90%)

Reason: The study reports baseline characteristics and states "There were no significant differences in any baseline characteristics between the groups, apart from self-reported shortness of breath (SOB) whereby the INT group had significantly higher SOB than the AC group."

Result of Domain 1: Low risk (High certainty - 90%)

Reason: Randomization and allocation concealment were adequate, and baseline characteristics were similar between groups except for one measure.

Domain 2: Risk of bias due to deviations from the intended interventions

2.1: Y (High certainty - 95%)

Reason: Participants were aware of their assigned intervention as they received a Fitbit and feedback.

2.2: Y (High certainty - 95%)

Reason: The physiotherapists providing the intervention were aware of participants' assignments.

2.3: NI (Low certainty - 70%)

Reason: No information is provided about non-protocol interventions.

2.4: N (Moderate certainty - 80%)

Reason: The study does not report any significant implementation failures.

2.5: N (Moderate certainty - 80%)

Reason: The study reports high adherence: "Overall step count goals achievement by the INT group was 75%."

2.6: Y (High certainty - 90%)

Reason: The analysis method is described as "intention-to-treat (ITT) principles".

Result of Domain 2: Some concerns (Moderate certainty - 80%)

Reason: While participants and personnel were aware of assignments, adherence was high and appropriate analysis methods were used. However, there is insufficient information on some aspects.

Domain 3: Risk of bias due to missing outcome data

3.1: Y (High certainty - 90%)

Reason: The study provides a CONSORT flow diagram showing minimal loss to follow-up.

3.2: N (High certainty - 90%)

Reason: No evidence of methods to correct for bias due to missing data.

3.3: N (Moderate certainty - 80%)

Reason: There is no indication that missingness depends on the true value.

3.4: N (Moderate certainty - 80%)

Reason: Given the low dropout rate and use of ITT analysis, it's unlikely missingness depends on the true value.

Result of Domain 3: Low risk (High certainty - 90%)

Reason: There was minimal missing data and appropriate methods were used to handle it.

Domain 4: Risk of bias in measurement of the outcome

4.1: N (High certainty - 90%)

Reason: The IPAQ is described as a validated measure for physical activity.

4.2: N (High certainty - 90%)

Reason: The measurement method (IPAQ) appears to be the same for both groups.

4.3: Y (High certainty - 95%)

Reason: The IPAQ is a self-reported measure, and participants were aware of their intervention.

4.4: Y (High certainty - 95%)

Reason: As a subjective self-reported measure, the IPAQ could be influenced by knowledge of the intervention.

4.5: N (Moderate certainty - 80%)

Reason: There is no clear evidence that assessment was influenced by knowledge of the intervention.

Result of Domain 4: High risk (High certainty - 90%)

Reason: While the measurement method is appropriate, the self-reported nature of the outcome could be influenced by participants' knowledge of their intervention.

Domain 5: Risk of bias in selection of the reported result

5.1: Y (High certainty - 95%)

Reason: The study was pre-registered on ClinicalTrials.gov (NCT03672058) and the analysis plan seems pre-specified.

5.2: N (High certainty - 90%)

Reason: The reported results align with the pre-specified outcomes and measures.

5.3: N (High certainty - 90%)

Reason: The reported analyses align with the pre-specified analysis plan.

Result of Domain 5: Low risk (High certainty - 90%)

Reason: The study was pre-registered and there's no evidence of selective reporting.

Overall Bias Risk for the Article: High risk (Moderate certainty - 80%)

Reason: While some domains show low risk of bias, there is a high risk in domain 4 due to the self-reported nature of the outcome measure. The overall assessment is high risk primarily due to this concern.

Study 24-1

Here is my assessment of the risk of bias for the Fan 2012 study:

**Article ID**: Fan, 2012

**Outcome Name**: Health-related quality of life

**Registration number**: NCT00395083

Domain 1: Risk of bias arising from the randomization process

1.1: Y (High Certainty, 95%)

Reason: The study states "Patients were randomly assigned, stratifying by site based on random, permuted blocks of variable size."

1.2: PY (Moderate Certainty, 80%)

Reason: The study mentions allocation concealment but does not provide specific details: "Staff blinded to study group performed telephone-based assessment of COPD exacerbations and hospitalizations, and all hospitalizations were blindly adjudicated."

1.3: N (High Certainty, 95%)

Reason: Table 1 shows baseline characteristics were generally similar between groups.

Result of Domain 1: Low risk (High Certainty, 95%)

Reason: Randomization was adequately performed, allocation was likely concealed, and baseline differences do not suggest problems with the randomization process.

Domain 2: Risk of bias due to deviations from intended interventions

2.1: Y (High Certainty, 95%)

Reason: Patients were aware of their intervention assignment as it involved educational sessions.

2.2: Y (High Certainty, 95%)

Reason: Care providers were aware of intervention assignment as they delivered the educational sessions.

2.3: NI (Low Certainty, 60%)

Reason: No information provided on non-protocol interventions.

2.4: N (Moderate Certainty, 80%)

Reason: The study reports high compliance with the intervention: "87% completed all 4 individual educational visits and 57% completed the group visit."

2.5: N (Moderate Certainty, 80%)

Reason: No indication of substantial non-adherence to assigned intervention.

2.6: N (High Certainty, 95%)

Reason: Analysis was intention-to-treat, which is not appropriate for evaluating the effect of adhering to the intervention.

Result of Domain 2: Some concerns (Moderate Certainty, 80%)

Reason: While participants and providers were aware of intervention status, there is no evidence of substantial deviations from intended interventions.

Domain 3: Risk of bias due to missing outcome data

3.1: Y (High Certainty, 95%)

Reason: Data was available for over 95% of randomized participants (209/217 in usual care, 208/209 in intervention).

3.2: Y (Moderate Certainty, 80%)

Reason: Analysis was intention-to-treat, suggesting missing data was unlikely to bias results.

3.3: N (Moderate Certainty, 80%)

Reason: No indication that missingness depended on true outcome value.

3.4: N (Moderate Certainty, 80%)

Reason: No evidence that missingness depended on true outcome value.

Result of Domain 3: Low risk (High Certainty, 90%)

Reason: Very little missing data and no evidence that missingness biased results.

Domain 4: Risk of bias in measurement of the outcome

4.1: N (High Certainty, 95%)

Reason: Health-related quality of life was measured using validated questionnaires (St. George's Respiratory Questionnaire and SF-12).

4.2: N (High Certainty, 95%)

Reason: No indication that measurement methods differed between groups.

4.3: N (High Certainty, 95%)

Reason: Outcome assessors were blinded: "Staff blinded to study group performed telephone-based assessment of COPD exacerbations and hospitalizations".

4.4: Y (High Certainty, 95%)

Reason: Health-related quality of life is a subjective outcome that could be influenced by knowledge of intervention.

4.5: N (Moderate Certainty, 80%)

Reason: No clear evidence that assessment was influenced by knowledge of intervention received.

Result of Domain 4: Low risk (High Certainty, 90%)

Reason: Outcome measurement was appropriate and unbiased, with blinded outcome assessment.

Domain 5: Risk of bias in selection of the reported result

5.1: Y (High Certainty, 95%)

Reason: The study was registered (NCT00395083) and outcomes appear to be reported as pre-specified.

5.2: N (High Certainty, 95%)

Reason: Reported outcome measurements align with those specified in methods.

5.3: N (High Certainty, 95%)

Reason: No evidence of selection of results based on multiple analyses.

Result of Domain 5: Low risk (High Certainty, 95%)

Reason: Analysis and reporting appear to follow a pre-specified plan without selective reporting.

Overall Bias Risk for the Article: Low risk (High Certainty, 90%)

Reason: Most domains were assessed as low risk, with only some concerns in Domain 2. The randomization, outcome measurement, and analysis methods appear robust.

Study 24-2

Here is my assessment of the risk of bias for the Fan et al. 2012 study:

**Article ID**: Fan, 2012

**Outcome Name**: Health-related quality of life

**Registration number**: NCT00395083

Domain 1: Risk of bias arising from the randomization process

1.1: Y (High Certainty - 95%)

Reason: The study describes using computer-generated random numbers for sequence generation.

1.2: Y (High Certainty - 95%)

Reason: The article mentions central allocation was used to conceal the allocation sequence.

1.3: N (Moderate Certainty - 85%)

Reason: Baseline characteristics between groups appear similar, with no significant differences noted.

Result of Domain 1: Low risk (High Certainty - 95%)

Reason: Adequate randomization and allocation concealment, with no baseline imbalances.

Domain 2: Risk of bias due to deviations from intended interventions

2.1: Y (High Certainty - 95%)

Reason: Participants were aware of their assigned intervention due to the nature of the educational program.

2.2: Y (High Certainty - 95%)

Reason: Care providers were aware of participants' assigned interventions.

2.3: NI (Low Certainty - 60%)

Reason: No clear information provided on non-protocol interventions.

2.4: N (Moderate Certainty - 80%)

Reason: No failures in implementing the intervention were reported.

2.5: N (Moderate Certainty - 80%)

Reason: Case managers completed 89% of scheduled reinforcement calls, suggesting adherence to the intervention was generally good

2.6: N (High Certainty - 95%)

Reason: An intention-to-treat analysis was used, which is considered inappropriate for evaluating adherence effects.

Result of Domain 2: High risk (High Certainty - 90%)

Reason: Participants and providers were aware of assignments, and inappropriate analysis methods were used.

Domain 3: Risk of bias due to missing outcome data

3.1: Y (High Certainty - 95%)

Reason: Over 95% of randomized participants had complete outcome data.

3.2: Y (Moderate Certainty - 80%)

Reason: Missingness unlikely to depend on true outcome value given high completion rate.

3.3: N (Moderate Certainty - 80%)

Reason: Missingness unlikely related to true outcome value given high completion rate.

3.4: N (Moderate Certainty - 80%)

Reason: No evidence that missingness depended on true outcome value.

Result of Domain 3: Low risk (High Certainty - 90%)

Reason: Very low amount of missing data, unlikely to bias results.

Domain 4: Risk of bias in measurement of the outcome

4.1: N (High Certainty - 95%)

Reason: The St. George's Respiratory Questionnaire is an appropriate and validated measure for COPD quality of life.

4.2: N (High Certainty - 90%)

Reason: The measurement methods were comparable between groups.

4.3: Y (High Certainty - 95%)

Reason: Outcome assessors were aware of intervention received, as this was a patient-reported outcome.

4.4: Y (High Certainty - 95%)

Reason: Knowledge of intervention likely influenced self-reported quality of life.

4.5: Y (Moderate Certainty - 80%)

Reason: As a subjective patient-reported outcome, assessment was likely influenced by knowledge of intervention.

Result of Domain 4: High risk (High Certainty - 90%)

Reason: Subjective outcome likely influenced by participants' knowledge of intervention received.

Domain 5: Risk of bias in selection of the reported result

5.1: Y (High Certainty - 95%)

Reason: The study was registered and analyses appear to follow the pre-specified plan.

5.2: N (Moderate Certainty - 80%)

Reason: No evidence of selection from multiple outcome measurements.

5.3: N (Moderate Certainty - 80%)

Reason: No evidence of selection from multiple analyses of the outcome.

Result of Domain 5: Low risk (High Certainty - 90%)

Reason: Analysis appears to follow pre-specified plan with no evidence of selective reporting.

Overall Bias Risk for the Article: High risk (High Certainty - 90%)

Reason: High risk of bias in domains 2 and 4 due to lack of blinding and subjective outcome measurement.

Study 25-1

Here is my assessment of the risk of bias for the FitzGerald 2004 study:

**Article ID**: FitzGerald, 2004

**Outcome Name**: Treatment failure: need for systemic corticosteroids (of those starting inhaler)

**registration number**: Not reported

Domain 1

1.1: Y (High Certainty 95%)

Reason: The study states "Patients were randomised to treatment groups at visit 2 according to a blocked computer generated randomisation list for each centre."

1.2: Y (High Certainty 95%)

Reason: The study mentions "An independent pharmacist randomly allocated individuals to active or placebo inhalers", implying central allocation.

1.3: N (High Certainty 95%)

Reason: Table 1 shows baseline characteristics were similar between groups.

Result of the domain: Low risk (High Certainty 95%)

Reason: Adequate randomization and allocation concealment with no baseline imbalances.

Domain 2

2.1: N (High Certainty 95%)

Reason: The study states it was a "double blind" trial and used placebo inhalers.

2.2: N (High Certainty 95%)

Reason: As a double-blind study using placebo inhalers, carers and people delivering interventions were also unaware of allocation.

2.3: Y (Moderate Certainty 80%)

Reason: No information is provided about non-protocol interventions, so we assume they were balanced.

2.4: N (High Certainty 95%)

Reason: The article does not report significant failures in implementing the intervention. Compliance was reported as high.

2.5: N (High Certainty 95%)

Reason: The article reports high compliance rates (>95%) for both groups.

2.6: Y (High Certainty 95%)

Reason: The analysis was described as "all patients treated" (APT) which is similar to intention-to-treat.

Result of the domain: Low risk (High Certainty 90%)

Reason: Participants and personnel were blinded, and there's no clear evidence of deviations from intended interventions.

Domain 3

3.1: Y (High Certainty 95%)

Reason: 98 out of 98 (100%) participants who had an exacerbation were included in the analysis.

3.2: Y (High Certainty 95%)

Reason: All patients who had an exacerbation were included in the analysis.

3.3: N (High Certainty 90%)

Reason: There's no indication that missingness in the outcome depended on its true value.

3.4: N (High Certainty 90%)

Reason: No evidence suggests missingness depended on the true outcome value.

Result of the domain: Low risk (High Certainty 95%)

Reason: Complete outcome data for all participants who had an exacerbation.

Domain 4

4.1: N (High Certainty 95%)

Reason: The primary outcome (treatment failure) is appropriate and clearly defined.

4.2: N (High Certainty 95%)

Reason: The study used placebo controls, ensuring comparable measurement between groups.

4.3: N (High Certainty 95%)

Reason: The use of placebo controls indicates outcome assessors were unaware of intervention received.

4.4: N (High Certainty 90%)

Reason: While some components of the primary outcome are subjective (e.g., unstable asthma), others are objective (e.g., need for oral corticosteroids).

4.5: N (High Certainty 90%)

Reason: The blinded nature of the study makes it unlikely that assessment of the outcome was influenced by knowledge of the intervention received.

Result of the domain: Low risk (High Certainty 90%)

Reason: Appropriate blinding and mostly objective outcome measurement.

Domain 5

5.1: NI (Moderate Certainty 80%)

Reason: The article does not mention pre-registration or a pre-specified analysis plan.

5.2: N (High Certainty 95%)

Reason: The primary outcome is clearly defined and reported as pre-specified.

5.3: N (High Certainty 95%)

Reason: The analysis methods appear consistent between the methods and results sections.

Result of the domain: Some concerns (Moderate Certainty 80%)

Reason: While the reported outcomes match the methods, there's no clear evidence of a pre-specified analysis plan.

Overall Bias Risk for the Article: Some concerns (Moderate Certainty 80%)

Reason: While most domains show low risk of bias, the lack of information about a pre-specified analysis plan raises some concerns. However, the study appears to be well-designed and conducted overall.

Study 25-2

Here is my assessment of the risk of bias for this study:

**Article ID**: FitzGerald, 2004

**Outcome Name**: Treatment failure: need for systemic corticosteroids (of those starting inhaler)

**Registration number**: Not reported

Domain 1

1.1: Y (High Certainty 95%)

Reason: The study states "Patients were randomised to treatment groups at visit 2 according to a blocked computer generated randomisation list for each centre."

1.2: Y (High Certainty 95%)

Reason: The study used central allocation, stating "An independent pharmacist randomly allocated individuals to active or placebo inhalers..."

1.3: N (High Certainty 95%)

Reason: Table 1 shows baseline characteristics were well balanced between groups.

Result of Domain 1: Low risk (High Certainty 95%)

Reason: Adequate randomization and allocation concealment, with no baseline imbalances.

Domain 2

2.1: N (High Certainty 95%)

Reason: The study is described as double-blind with placebo inhalers used.

2.2: N (High Certainty 95%)

Reason: The study is described as double-blind, indicating care providers were also blinded.

2.3: N (Moderate Certainty 80%)

Reason: No information suggests imbalanced non-protocol interventions between groups.

2.4: N (High Certainty 90%)

Reason: Only 16 out of 290 patients (5.5%) discontinued for reasons other than exacerbations, which is less than 10%.

2.5: N (Moderate Certainty 80%)

Reason: Compliance was reported as high in both groups (>95%) based on self-reported data.

2.6: N (High Certainty 95%)

Reason: The analysis is described as "all patients treated" (APT), which is equivalent to intention-to-treat for those experiencing exacerbations.

Result of Domain 2: Low risk (High Certainty 90%)

Reason: The study was blinded and there is little evidence of deviations from intended interventions.

Domain 3

3.1: Y (High Certainty 95%)

Reason: Of 290 randomized patients, 98 experienced exacerbations and were included in the primary analysis.

3.2: Y (Moderate Certainty 80%)

Reason: All patients who experienced exacerbations were included in the analysis.

3.3: N (Moderate Certainty 75%)

Reason: Reasons for withdrawals were similar between groups and mostly unrelated to the outcome.

3.4: N (Moderate Certainty 75%)

Reason: No evidence suggests missingness in the outcome depended on its true value.

Result of Domain 3: Low risk (High Certainty 90%)

Reason: Data was available for all patients who experienced exacerbations, with appropriate analysis methods used.

Domain 4

4.1: N (High Certainty 95%)

Reason: Treatment failure (need for systemic corticosteroids, unscheduled visits, or unstable asthma) is an appropriate measure of outcome.

4.2: N (High Certainty 95%)

Reason: The outcome was measured the same way for both groups.

4.3: N (High Certainty 95%)

Reason: The study was double-blind, so outcome assessors were not aware of intervention received.

4.4: N (High Certainty 90%)

Reason: The need for systemic corticosteroids or unscheduled visits are objective measures unlikely to be influenced by knowledge of the intervention. Unstable asthma was defined by objective criteria.

4.5: N (High Certainty 90%)

Reason: As mostly objective measures were used, assessment of this outcome is unlikely to have been influenced by knowledge of the intervention.

Result of Domain 4: Low risk (High Certainty 95%)

Reason: The outcome measurement was appropriate, consistent, and blinded.

Domain 5

5.1: NI (Moderate Certainty 80%)

Reason: No information is provided about pre-specification of the analysis plan or trial registration.

5.2: N (High Certainty 90%)

Reason: The primary outcome is clearly defined and reported as pre-specified.

5.3: N (High Certainty 90%)

Reason: The analysis methods appear consistent with the pre-specified approach.

Result of Domain 5: Some concerns (Moderate Certainty 80%)

Reason: While the outcome and analysis appear pre-specified, lack of information about a formal analysis plan raises some concerns.

Overall Bias Risk for the Article: Some concerns (Moderate Certainty 85%)

Reason: While most domains show low risk of bias, the lack of information about pre-specification of the analysis plan in Domain 5 leads to an overall assessment of some concerns.

Study 26-1

Based on the provided instructions and the article content, here is my assessment of the risk of bias for the Harrison 2004 study:

**Article ID**: Harrison, 2004

**Outcome Name**: Treatment failure: need for systemic corticosteroids (of those starting inhaler)

**registration number**: Not reported

Domain 1

1.1: Y (High Certainty 95%)

Reason: The article states "An independent pharmacist randomly allocated individuals to active or placebo inhalers using computer-generated random number tables."

1.2: Y (High Certainty 95%)

Reason: The article mentions "An independent pharmacist randomly allocated individuals to active or placebo inhalers", implying central allocation.

1.3: N (Moderate Certainty 85%)

Reason: The baseline characteristics table shows no significant differences between groups.

Result of the domain: Low risk (High Certainty 95%)

Reason: Adequate randomization and allocation concealment with no baseline imbalances.

Domain 2

2.1: N (High Certainty 95%)

Reason: The article states "participants were unaware of their allocation" and mentions use of placebo inhalers.

2.2: N (High Certainty 95%)

Reason: The study used placebo inhalers, indicating carers and people delivering interventions were also unaware of allocation.

2.3: Y (Moderate Certainty 80%)

Reason: No information is provided about non-protocol interventions, so we assume they were balanced.

2.4: N (Moderate Certainty 80%)

Reason: The article does not report significant failures in implementing the intervention.

2.5: N (Low Certainty 70%)

Reason: The article does not provide specific information about adherence rates.

2.6: N (High Certainty 95%)

Reason: The analysis was described as "intention-to-treat".

Result of the domain: Low risk (High Certainty 90%)

Reason: Participants and personnel were blinded, and there's no clear evidence of deviations from intended interventions.

Domain 3

3.1: Y (High Certainty 95%)

Reason: 353 out of 390 (90.5%) randomized participants completed the study.

3.2: N (Moderate Certainty 80%)

Reason: No evidence of methods to correct for bias due to missing data.

3.3: PN (Moderate Certainty 80%)

Reason: Reasons for withdrawal appear unrelated to the outcome (lost to follow-up, personal reasons, GP advice).

3.4: N (Moderate Certainty 80%)

Reason: No evidence suggests missingness depended on the true outcome value.

Result of the domain: Low risk (High Certainty 90%)

Reason: High completion rate and low likelihood of bias from missing data.

Domain 4

4.1: N (High Certainty 95%)

Reason: The primary outcome (need for oral prednisolone) is appropriate and objective.

4.2: N (High Certainty 95%)

Reason: The study used placebo controls, ensuring comparable measurement between groups.

4.3: N (High Certainty 95%)

Reason: The use of placebo controls indicates outcome assessors were unaware of intervention received.

4.4: N (High Certainty 95%)

Reason: The primary outcome (need for oral prednisolone) is an objective measure.

4.5: N (High Certainty 95%)

Reason: As an objective measure, assessment of the outcome is unlikely to be influenced by knowledge of the intervention.

Result of the domain: Low risk (High Certainty 95%)

Reason: Appropriate blinding and objective outcome measurement.

Domain 5

5.1: NI (Moderate Certainty 80%)

Reason: The article does not mention pre-registration or a pre-specified analysis plan.

5.2: N (High Certainty 95%)

Reason: The primary outcome is clearly defined and reported as pre-specified.

5.3: N (High Certainty 95%)

Reason: The analysis methods appear consistent between the methods and results sections.

Result of the domain: Some concerns (Moderate Certainty 80%)

Reason: While the reported outcomes match the methods, there's no clear evidence of a pre-specified analysis plan.

Overall Bias Risk for the Article: Some concerns (Moderate Certainty 80%)

Reason: While most domains show low risk of bias, the lack of information about a pre-specified analysis plan raises some concerns. However, the study appears to be well-designed and conducted overall.

Study 26-2

Based on the information provided in the article, here is my assessment of the risk of bias:

**Article ID**: Harrison, 2004

**Outcome Name**: Treatment failure: need for systemic corticosteroids (of those starting inhaler)

**registration number**: Not reported

Domain 1

1.1: Y (High Certainty 95%)

Reason: The article states "An independent pharmacist randomly allocated individuals to active or placebo inhalers using computer-generated random number tables."

1.2: Y (High Certainty 95%)

Reason: The article mentions "An independent pharmacist randomly allocated individuals to active or placebo inhalers", indicating central allocation was used.

1.3: N (Moderate Certainty 85%)

Reason: Table 1 shows baseline characteristics were well-matched between groups, with no significant differences noted.

Result of Domain 1: Low risk (High Certainty 95%)

Reason: Adequate randomization and allocation concealment, with no baseline imbalances.

Domain 2

2.1: N (High Certainty 95%)

Reason: The study is described as double-blind with placebo inhalers used.

2.2: N (High Certainty 95%)

Reason: The study is described as double-blind, indicating care providers were also blinded.

2.3: N (Moderate Certainty 80%)

Reason: No information suggests imbalanced non-protocol interventions between groups.

2.4: N (Moderate Certainty 80%)

Reason: While some participants withdrew, it was less than 10% in each group.

2.5: NI (Low Certainty 60%)

Reason: No specific information is reported regarding adherence to the assigned intervention.

2.6: N (High Certainty 95%)

Reason: The analysis is described as intention-to-treat.

Result of Domain 2: Low risk (Moderate Certainty 85%)

Reason: The study was blinded and there is little evidence of deviations from intended interventions.

Domain 3

3.1: Y (High Certainty 95%)

Reason: Only 37 out of 390 participants (9.5%) withdrew or were lost to follow-up.

3.2: N (Moderate Certainty 80%)

Reason: No evidence of methods to correct for bias due to missing data.

3.3: PN (Moderate Certainty 75%)

Reason: Reasons for withdrawals were similar between groups and mostly unrelated to the outcome.

3.4: N (Moderate Certainty 75%)

Reason: No evidence suggests missingness in the outcome depended on its true value.

Result of Domain 3: Low risk (High Certainty 90%)

Reason: Low amount of missing data, with appropriate analysis methods used.

Domain 4

4.1: N (High Certainty 95%)

Reason: The need for oral prednisolone is an appropriate measure of treatment failure.

4.2: N (High Certainty 95%)

Reason: The outcome was measured the same way for both groups.

4.3: N (High Certainty 95%)

Reason: The study was double-blind, so outcome assessors were not aware of intervention received.

4.4: N (High Certainty 95%)

Reason: The need for oral prednisolone is an objective measure unlikely to be influenced by knowledge of the intervention.

4.5: N (High Certainty 95%)

Reason: As an objective measure, assessment of this outcome is unlikely to have been influenced by knowledge of the intervention.

Result of Domain 4: Low risk (High Certainty 95%)

Reason: The outcome measurement was appropriate, consistent, and blinded.

Domain 5

5.1: NI (Moderate Certainty 80%)

Reason: No information is provided about pre-specification of the analysis plan or trial registration.

5.2: N (High Certainty 90%)

Reason: The primary outcome is clearly defined and reported as pre-specified.

5.3: N (High Certainty 90%)

Reason: The analysis methods appear consistent with the pre-specified approach.

Result of Domain 5: Some concerns (Moderate Certainty 80%)

Reason: While the outcome and analysis appear pre-specified, lack of information about a formal analysis plan raises some concerns.

Overall Bias Risk for the Article: Some concerns (Moderate Certainty 85%)

Reason: While most domains show low risk of bias, the lack of information about pre-specification of the analysis plan in Domain 5 leads to an overall assessment of some concerns.

Study 27-1

Here is my assessment of the risk of bias for the Hernández et al. 2015 study:

Article ID: Hernández, 2015

Outcome Name: Health-related quality of life

Registration number: Not reported

Domain 1: Risk of bias arising from the randomization process

1.1: Y (95% confidence)

Reason: The article states "A computer-generated list of random numbers with no restrictions and administered by personnel who were not involved in the study ensured blinded randomisation (1:1 ratio)"

1.2: Y (95% confidence)

Reason: The article states "A computer-generated list of random numbers with no restrictions and administered by personnel who were not involved in the study ensured blinded randomisation (1:1 ratio)"

1.3: N (90% confidence)

Reason: Table 1 shows no significant baseline differences between groups.

Result of Domain 1: Low risk (95% confidence)

Reason: Adequate randomization and allocation concealment, with no concerning baseline differences.

Domain 2: Risk of bias due to deviations from intended interventions

2.1: Y (95% confidence)

Reason: Participants were aware of their assigned intervention as this was an open-label study.

2.2: Y (95% confidence)

Reason: Care providers were aware of participants' assigned interventions.

2.3: NI (75% confidence)

Reason: No information provided on non-protocol interventions.

2.4: N (80% confidence)

Reason: No information suggests >10% failure in implementation.

2.5: NI (80% confidence)

Reason: No specific information is reported regarding adherence to the assigned intervention.

2.6: N (95%) Reason: The article reports using intention-to-treat analysis.

Result of Domain 2: Some concerns (75% confidence)

Reason: Participants and providers were aware of assigned interventions, but impact on outcomes is unclear.

Domain 3: Risk of bias due to missing outcome data

3.1: N (90% confidence)

Reason: 114 out of 155 (73.5%) patients completed the 12-month assessment.

3.2: N (90% confidence)

Reason: No evidence of methods to correct for bias due to missing data.

3.3: PY (80% confidence)

Reason: Missingness may be related to health status.

3.4: NI (75% confidence)

Reason: Insufficient information to determine if missingness depends on true value.

Result of Domain 3: High risk (80% confidence)

Reason: Substantial missing data with potential relationship to true outcome.

Domain 4: Risk of bias in measurement of the outcome

4.1: N (90% confidence)

Reason: SGRQ is an appropriate measure for health-related quality of life in COPD.

4.2: N (90% confidence)

Reason: No evidence of differential measurement methods between groups.

4.3: Y (95% confidence)

Reason: Outcome was patient-reported and patients were aware of intervention received.

4.4: Y (95% confidence)

Reason: Health-related quality of life is a subjective outcome.

4.5: N (80% confidence)

Reason: No clear evidence that assessment was influenced by knowledge of intervention received.

Result of Domain 4: Some concerns (80% confidence)

Reason: Subjective outcome with patient awareness of intervention, but no clear evidence of influence on assessment.

Domain 5: Risk of bias in selection of the reported result

5.1: NI (75% confidence)

Reason: No information on pre-specified analysis plan.

5.2: N (80% confidence)

Reason: SGRQ reported as expected for health-related quality of life.

5.3: N (80% confidence)

Reason: No evidence of selection from multiple analyses.

Result of Domain 5: Some concerns (75% confidence)

Reason: Lack of information on pre-specified analysis plan, but no clear evidence of selective reporting.

Overall Bias Risk for the Article: High risk (80% confidence)

Reason: High risk of bias due to missing outcome data, with some concerns in other domains.

Study 27-2

Here is my assessment of the risk of bias for the Hernández et al. 2015 study:

**Article ID**: Hernández, 2015

**Outcome Name**: Health-related quality of life

**Registration number**: Not reported

Domain 1

1.1: NI (95%)

Reason: After double-checking, the article does not report the use of computer-generated random numbers, block randomization, random number tables, or other methods to generate random sequences.

1.2: NI (95%)

Reason: After careful checking, the article does not mention central allocation, envelopes, or postcards for allocation concealment.

1.3: N (95%)

Reason: The article states that the two groups showed similar characteristics at entry, except for vaccination rates.

Result of the domain: Some concerns (95%)

Reason: While randomization was performed, details on sequence generation and allocation concealment are not provided.

Domain 2

2.1: Y (95%)

Reason: The study was not blinded.

2.2: Y (95%)

Reason: Care providers were aware of participants' assigned interventions.

2.3: NI (70%)

Reason: The article does not provide information on non-protocol interventions.

2.4: N (80%)

Reason: The article does not report that more than 10% of participants had failures in implementing the intervention.

2.5: NI (80%)

Reason: The article does not report that more than 10% of participants had non-adherence to the assigned intervention.

2.6: N (95%)

Reason: The article reports using intention-to-treat analysis.

Result of the domain: High risk (85%)

Reason: Participants and care providers were aware of the assigned interventions, and the analysis method is considered inappropriate for evaluating compliance.

Domain 3

3.1: N (90%)

Reason: Less than 90% of randomized participants had complete outcome data.

3.2: N (95%)

Reason: The article does not report using appropriate methods to address missing data.

3.3: PY (80%)

Reason: Patients lost to follow-up had worse baseline characteristics related to the outcome.

3.4: PY (75%)

Reason: It is likely that missingness in the outcome depended on its true value.

Result of the domain: High risk (85%)

Reason: There was substantial missing data, likely related to the outcome, without appropriate methods to address it.

Domain 4

4.1: N (90%)

Reason: The study used a validated tool for measuring health-related quality of life in COPD patients.

4.2: N (85%)

Reason: The article does not indicate any differences in outcome measurement between groups.

4.3: Y (95%)

Reason: As this was an unblinded study, outcome assessors were likely aware of the intervention received by participants.

4.4: Y (95%)

Reason: Health-related quality of life is a subjective measure reported by patients who were aware of their treatment assignment.

4.5: N (80%)

Reason: There is no clear evidence to prove assessment of the outcome was influenced by knowledge of intervention received.

Result of the domain: Some concerns (85%)

Reason: While the outcome measure was subjective and participants were aware of their treatment assignment, there's no clear evidence of influence on the assessment.

Domain 5

5.1: NI (95%)

Reason: The article does not mention a pre-specified analysis plan or trial registration.

5.2: N (85%)

Reason: The article reports the total score and subscores of the SGRQ as expected for this outcome measure.

5.3: N (85%)

Reason: The article does not provide evidence of multiple analyses of the data for this outcome.

Result of the domain: Some concerns (85%)

Reason: While there's no evidence of selective reporting, the lack of a pre-specified analysis plan raises some concerns.

Overall Bias Risk for the Article: High risk (85%)

Reason: The study has high risk of bias in multiple domains, particularly related to lack of blinding and missing outcome data.

Study 28-1

Here is my assessment of the risk of bias for this study:

**Article ID**: Johnson-Warrington, 2016

**Outcome Name**: Health-related quality of life (CRQ-SR)

**Registration number**: ISRCTN84599369

Domain 1

1.1: Y (95%)

Reason: The article states "Participants were randomized to receive usual care or SPACE for COPD via a web-based, concealed allocation program (www.sealedenvelope.com) using simple random permuted block 1:1 randomization by VJ-W."

1.2: Y (95%)

Reason: The article mentions "concealed allocation program" for randomization.

1.3: N (95%)

Reason: Table 1 shows baseline characteristics were similar between groups.

Result of the domain: Low risk (95%)

Reason: Proper randomization and allocation concealment were used, and baseline characteristics were similar.

Domain 2

2.1: Y (95%)

Reason: Due to the nature of the intervention, participants were aware of their group assignment.

2.2: Y (95%)

Reason: Care providers were aware of participants' assigned interventions.

2.3: NI (70%)

Reason: The article does not provide information on non-protocol interventions.

2.4: N (80%)

Reason: The article does not report that more than 10% of participants had failures in implementing the intervention.

2.5: NI (80%)

Reason: The article does not report that more than 10% of participants had non-adherence to the assigned intervention.

2.6: N (95%)

Reason: The article reports using intention-to-treat analysis, which is considered inappropriate for evaluating compliance.

Result of the domain: High risk (85%)

Reason: Participants and care providers were aware of the assigned interventions, and intention-to-treat analysis was used.

Domain 3

3.1: Y (90%)

Reason: 71 out of 78 randomized participants (91%) had complete outcome data.

3.2: N (95%)

Reason: The article does not report using appropriate methods to address missing data.

3.3: N (80%)

Reason: The article does not suggest that missingness depends on the true value of the outcome.

3.4: N (80%)

Reason: There is no evidence that missingness in the outcome depended on its true value.

Result of the domain: Low risk (85%)

Reason: High completion rate and no evidence of missingness related to the outcome.

Domain 4

4.1: N (90%)

Reason: The study used the validated CRQ-SR to measure health-related quality of life.

4.2: N (85%)

Reason: The article does not indicate any differences in outcome measurement between groups.

4.3: Y (95%)

Reason: As this was an unblinded study, outcome assessors were likely aware of the intervention received by participants.

4.4: Y (95%)

Reason: Health-related quality of life is a subjective measure reported by patients who were aware of their treatment assignment.

4.5: N (80%)

Reason: There is no clear evidence to prove assessment of the outcome was influenced by knowledge of intervention received.

Result of the domain: Some concerns (85%)

Reason: While the outcome measure was subjective and participants were aware of their treatment assignment, there's no clear evidence of influence on the assessment.

Domain 5

5.1: Y (95%)

Reason: The article mentions trial registration (ISRCTN84599369).

5.2: N (85%)

Reason: The article reports all expected subscores of the CRQ-SR.

5.3: N (85%)

Reason: The article does not provide evidence of multiple analyses of the data for this outcome.

Result of the domain: Low risk (85%)

Reason: The study was pre-registered and reported outcomes as expected.

Overall Bias Risk for the Article: Some concerns (85%)

Reason: While randomization and outcome reporting appear appropriate, the lack of blinding and use of intention-to-treat analysis raise some concerns about potential bias.

Study 28-2

Based on the information provided in the article, here is my assessment of the risk of bias:

**Article ID**: Johnson-Warrington, 2016

**Outcome Name**: Health-related quality of life

**Registration number**: ISRCTN84599369

Domain 1: Risk of bias arising from the randomization process

1.1: Y (95% confidence)

Reason: The study used "a web-based, concealed allocation program (www.sealedenvelope.com) using simple random permuted block 1:1 randomization"

1.2: Y (95% confidence)

Reason: The study used "a web-based, concealed allocation program" which indicates central allocation was used to conceal the allocation sequence

1.3: N (90% confidence)

Reason: The baseline characteristics table shows no significant differences between groups

Result of Domain 1: Low risk (95% confidence)

Reason: Allocation was adequately generated and concealed, and baseline differences do not suggest problems with the randomization process

Domain 2: Risk of bias due to deviations from intended interventions

2.1: Y (90% confidence)

Reason: Participants were likely aware of their assigned intervention as blinding is not mentioned

2.2: Y (90% confidence)

Reason: Carers/people delivering interventions were likely aware of participants' assigned intervention as blinding is not mentioned

2.3: NI (80% confidence)

Reason: No information provided on non-protocol interventions

2.4: N (85% confidence)

Reason: No information suggesting >10% failure in implementation

2.5: NI (85% confidence)

Reason: No information suggesting >10% non-adherence

2.6: N (90% confidence)

Reason: Analysis was intention-to-treat

Result of Domain 2: Some concerns (85% confidence)

Reason: Participants and personnel were likely aware of intervention assignments, but this knowledge likely did not lead to deviations from intended interventions

Domain 3: Missing outcome data

3.1: PN (90% confidence)

Reason: 36/39 (92%) in usual care and 35/39 (90%) in intervention group had follow-up data

3.2: N (85% confidence)

Reason: No evidence provided that results were not biased by missing data

3.3: PY (80% confidence)

Reason: Missingness may be related to health status, which could be related to outcome

3.4: N (80% confidence)

Reason: No clear evidence that missingness depended on true value

Result of Domain 3: Some concerns (85% confidence)

Reason: Some missing outcome data, but not clear if this biased results

Domain 4: Measurement of the outcome

4.1: N (90% confidence)

Reason: CRQ-SR is an appropriate measure for health-related quality of life in COPD

4.2: N (90% confidence)

Reason: No indication that measurement differed between groups

4.3: Y (95% confidence)

Reason: CRQ-SR is self-reported and participants were aware of intervention

4.4: Y (95% confidence)

Reason: CRQ-SR is a subjective measure reported by patients

4.5: N (85% confidence)

Reason: No clear evidence that assessment was influenced by knowledge of intervention

Result of Domain 4: Some concerns (90% confidence)

Reason: Outcome measure was appropriate but subjective and participants were aware of intervention

Domain 5: Selection of the reported result

5.1: Y (90% confidence)

Reason: Study was registered (ISRCTN84599369) and analysis appears to follow pre-specified plan

5.2: N (85% confidence)

Reason: No evidence of selecting from multiple outcome measurements

5.3: N (85% confidence)

Reason: No evidence of selecting from multiple analyses

Result of Domain 5: Low risk (85% confidence)

Reason: Analysis appears to follow pre-specified plan with no evidence of selective reporting

Overall Bias Risk for the Article: Some concerns (85% confidence)

Reason: Some concerns in domains 2, 3 and 4, but low risk in domains 1 and 5. No high risk domains.

Study 29-1

Here is my assessment of the risk of bias for the Jorm et al. 2010 study:

**Article ID**: Jorm, 2010

**Outcome Name**: Individual mental health: 6 months to a year

**Registration number**: ACTRN12608000188336

Domain 1: Risk of bias arising from the randomization process

1.1: Y (High Certainty - 95%)

Reason: The study states "A computerized random number generator (Random Integers option in Random.org) was used to carry out a simple randomization procedure".

1.2: Y (High Certainty - 95%)

Reason: The study reports "The allocation sequence was concealed from J.F. until it was time to advise participants of their random assignment and send educational materials as required; that is, after baseline data was collected."

1.3: N (Moderate Certainty - 80%)

Reason: The baseline characteristics reported in Table 1 do not show any significant differences between groups that would suggest a problem with randomization.

Result of Domain 1: Low risk (High Certainty - 95%)

Reason: The allocation sequence was adequately generated and concealed, and there is no evidence of baseline imbalances.

Domain 2: Risk of bias due to deviations from the intended interventions

2.1: Y (High Certainty - 95%)

Reason: The nature of the interventions (e-learning CD, manual, or waiting list) meant participants were aware of their assigned intervention.

2.2: Y (High Certainty - 95%)

Reason: This was a self-administered intervention, so the people delivering it (the participants themselves) were aware of the intervention.

2.3: NI (Low Certainty - 60%)

Reason: The study does not provide information on non-protocol interventions.

2.4: N (Moderate Certainty - 80%)

Reason: Figure 1 shows that less than 10% of participants in each group failed to complete the intervention.

2.5: NI (Low Certainty - 60%)

Reason: The study does not provide specific information on adherence to the interventions.

2.6: N (High Certainty - 95%)

Reason: The study states "An intention-to-treat approach was used, with all participants included in the analyses."

Result of Domain 2: Some concerns (Moderate Certainty - 80%)

Reason: Participants were aware of their assigned interventions, but there is no evidence of substantial deviations from the intended interventions.

Domain 3: Missing outcome data

3.1: Y (High Certainty - 95%)

Reason: Figure 1 shows that over 95% of randomized participants were included in the analysis for each group.

3.2: N (High Certainty - 95%)

Reason: No evidence of methods to correct for bias due to missing data.

3.3: N (Moderate Certainty - 80%)

Reason: There is no indication that missingness depended on the true value of the outcome.

3.4: N (Moderate Certainty - 80%)

Reason: There is no evidence to suggest that missingness depended on the true value of the outcome.

Result of Domain 3: Low risk (High Certainty - 95%)

Reason: There was very little missing outcome data, and an appropriate analysis method was used.

Domain 4: Risk of bias in measurement of the outcome

4.1: N (High Certainty - 90%)

Reason: The outcome measures used appear to be appropriate and validated.

4.2: N (High Certainty - 90%)

Reason: The measurement methods were the same for all intervention groups.

4.3: Y (High Certainty - 95%)

Reason: Participants were aware of their intervention and self-reported most outcomes.

4.4: Y (High Certainty - 90%)

Reason: Many of the outcomes were subjective measures that could be influenced by knowledge of the intervention received.

4.5: PY (Moderate Certainty - 75%)

Reason: Given the nature of the self-reported outcomes, it's likely that assessment was influenced by knowledge of the intervention, but there's no direct evidence of this.

Result of Domain 4: High risk (Moderate Certainty - 80%)

Reason: The outcomes were subjective and likely influenced by participants' knowledge of their intervention.

Domain 5: Risk of bias in selection of the reported result

5.1: Y (High Certainty - 95%)

Reason: The study was registered prior to commencement (ACTRN12608000188336) and the reported analyses appear to align with the pre-specified plan.

5.2: N (Moderate Certainty - 80%)

Reason: The reported results correspond to all intended outcome measurements.

5.3: N (Moderate Certainty - 80%)

Reason: There is no evidence of multiple eligible analyses of the data.

Result of Domain 5: Low risk (High Certainty - 90%)

Reason: The study was pre-registered and there is no evidence of selective reporting.

Overall Bias Risk for the Article: High risk (Moderate Certainty - 80%)

Reason: While the randomization process and handling of missing data were good, the high risk of bias in measurement of outcomes (due to participants' awareness of intervention and subjective outcomes) leads to an overall high risk of bias assessment.

Study 29-2

I apologize for the misunderstanding in my previous response. I will revise the assessment based on your instructions, particularly regarding 3.2 and intention-to-treat analysis. Here's the corrected evaluation:

**Article ID**: Jorm, 2010

**Outcome Name**: Individual mental health: 6 months to a year

**Registration number**: ACTRN12608000188336

Domain 1

1.1: Y (95% Confidence)

Reason: The article states "A computerized random number generator (Random Integers option in Random.org) was used to carry out a simple randomization procedure".

1.2: Y (95% Confidence)

Reason: The article mentions "The allocation sequence was concealed from J.F. until it was time to advise participants of their random assignment and send educational materials as required; that is, after baseline data was collected."

1.3: N (80% Confidence)

Reason: The article does not report any significant baseline differences between groups that would suggest a problem with randomization.

Result of the domain: Low risk (95% Confidence)

Reason: The allocation sequence was adequately generated and concealed, and there is no evidence of baseline imbalances.

Domain 2

2.1: Y (95% Confidence)

Reason: The article states "Blinding of participants was not possible."

2.2: Y (95% Confidence)

Reason: Since participants were aware of their intervention, those delivering the interventions would also have been aware.

2.3: NI (50% Confidence)

Reason: The article does not provide information on non-protocol interventions.

2.4: Y (95% Confidence)

Reason: The article reports that 13% of participants did not complete the follow-up questionnaire, which is more than 10% of participants.

2.5: NI (50% Confidence)

Reason: The article does not provide information regarding adherence to the assigned intervention.

2.6: N (95% Confidence)

Reason: The article used intention-to-treat analysis.

Result of the domain: High risk (80% Confidence)

Reason: Participants and intervention providers were aware of assigned interventions, more than 10% of participants did not complete the follow-up, and there is insufficient information about adherence to the intervention.

Domain 3

3.1: N (95% Confidence)

Reason: The article reports that 87% of participants completed the follow-up questionnaire, which is below the 95% threshold for continuous outcomes.

3.2: N (80% Confidence)

Reason: The study used an intention-to-treat approach, but this is not a method for correcting bias caused by missing data.

3.3: N (80% Confidence)

Reason: There is no indication that missingness depended on the true value of the outcome.

3.4: N (80% Confidence)

Reason: There is no evidence suggesting that missingness in the outcome depended on its true value.

Result of the domain: Some concerns (80% Confidence)

Reason: There was some missing outcome data, and no clear evidence of methods to correct for potential bias due to missing data.

Domain 4

4.1: N (95% Confidence)

Reason: The outcome measures used appear to be appropriate and validated.

4.2: N (95% Confidence)

Reason: The measurement methods were the same for all intervention groups.

4.3: Y (95% Confidence)

Reason: Participants were aware of their intervention and some outcomes were self-reported.

4.4: Y (95% Confidence)

Reason: Some outcomes were subjective and self-reported, which could be influenced by knowledge of the intervention received.

4.5: N (80% Confidence)

Reason: There is no clear evidence that assessment of the outcome was influenced by knowledge of the intervention received.

Result of the domain: Some concerns (80% Confidence)

Reason: While outcome measures were appropriate, participants' knowledge of their intervention could have influenced self-reported outcomes.

Domain 5

5.1: Y (95% Confidence)

Reason: The trial was registered (ACTRN12608000188336) and the reported outcomes align with those pre-specified.

5.2: N (90% Confidence)

Reason: The reported results correspond to all intended outcome measurements.

5.3: N (90% Confidence)

Reason: There is no evidence of multiple eligible analyses of the data.

Result of the domain: Low risk (90% Confidence)

Reason: The study appears to have followed a pre-specified analysis plan and reported all intended outcomes.

Overall Bias Risk for the Article: High risk (80% Confidence)

Study 30-1

Here is my assessment of the risk of bias for the Kessler et al. 2018 study:

**Article ID**: Kessler, 2018

**Outcome Name**: Health-related quality of life

**registration number**: NCT01241526

Domain 1: Risk of bias arising from the randomization process

1.1: Y (High Certainty, 95%)

Reason: The study states "Patients were allocated to groups in a 1:1 fashion according to a pre-specified randomisation list generated before the study by a partial-minimisation computer algorithm under supervision of the study sponsor."

1.2: Y (High Certainty, 95%)

Reason: The study reports "Patients were assigned a randomisation number by study staff at each centre in sequential numerical order through a telephone-based interactive voice response system."

1.3: N (Moderate Certainty, 85%)

Reason: Table 1 shows baseline characteristics were generally similar between groups, with no major imbalances noted.

Result of Domain 1: Low risk (High Certainty, 95%)

Reason: Adequate random sequence generation and allocation concealment, with no baseline imbalances suggesting problems with randomization.

Domain 2: Risk of bias due to deviations from intended interventions

2.1: Y (High Certainty, 95%)

Reason: The study was open-label, so participants were aware of their assigned intervention.

2.2: Y (High Certainty, 95%)

Reason: As an open-label study, care providers were also aware of participants' assigned interventions.

2.3: NI (Low Certainty, 60%)

Reason: The article does not provide clear information on non-protocol interventions.

2.4: N (Moderate Certainty, 80%)

Reason: The study reports high adherence to the intervention, with 66.7% of patients achieving at least 80% of planned coaching sessions.

2.5: N (Moderate Certainty, 80%)

Reason: High adherence is reported, with 89.7% of patients achieving at least 80% compliance for weekly health status transmissions.

2.6: Y (Moderate Certainty, 85%)

Reason: The primary analysis was conducted on the intention-to-treat population.

Result of Domain 2: Low risk (Moderate Certainty, 80%)

Reason: Despite awareness of intervention assignment, there is no evidence of substantial deviations from intended interventions that would bias results.

Domain 3: Risk of bias due to missing outcome data

3.1: Y (High Certainty, 95%)

Reason: 137/157 (87%) patients in the disease management group and 128/162 (79%) in the usual management group completed 12 months of follow-up, which is >95% for the primary outcome.

3.2: Y (Moderate Certainty, 80%)

Reason: The primary analysis was conducted on the intention-to-treat population, which helps mitigate bias from missing data.

3.3: NI (Low Certainty, 60%)

Reason: The article does not provide clear information on reasons for missing data in relation to the outcome.

3.4: NI (Low Certainty, 60%)

Reason: Insufficient information to determine if missingness depended on the true value.

Result of Domain 3: Low risk (Moderate Certainty, 80%)

Reason: High completion rates and use of ITT analysis suggest low risk of bias from missing data.

Domain 4: Risk of bias in measurement of the outcome

4.1: N (High Certainty, 95%)

Reason: Health-related quality of life was measured using the validated SGRQ-C questionnaire.

4.2: N (High Certainty, 95%)

Reason: The SGRQ-C questionnaire would have been administered similarly in both groups.

4.3: Y (High Certainty, 95%)

Reason: As an open-label study, outcome assessors were likely aware of intervention assignments.

4.4: Y (High Certainty, 95%)

Reason: Health-related quality of life is a subjective outcome that could be influenced by knowledge of intervention.

4.5: N (Moderate Certainty, 80%)

Reason: While the outcome is subjective, there is no clear evidence that assessment was actually influenced by knowledge of intervention received.

Result of Domain 4: Some concerns (Moderate Certainty, 85%)

Reason: While the outcome measure was appropriate, knowledge of intervention assignment could potentially influence this subjective outcome.

Domain 5: Risk of bias in selection of the reported result

5.1: Y (Moderate Certainty, 80%)

Reason: The study was registered (NCT01241526) and outcomes appear to be reported as pre-specified.

5.2: N (Moderate Certainty, 80%)

Reason: Health-related quality of life results are reported comprehensively using the SGRQ-C total score.

5.3: N (Moderate Certainty, 80%)

Reason: There is no evidence of selective reporting from multiple analyses.

Result of Domain 5: Low risk (Moderate Certainty, 80%)

Reason: Results appear to be reported as pre-specified without evidence of selection based on results.

Overall Bias Risk for the Article: Some concerns (Moderate Certainty, 85%)

Reason: While most domains were assessed as low risk, there are some concerns regarding potential bias in measurement of the subjective quality of life outcome in this open-label study. However, other aspects of the study design and conduct help mitigate overall risk of bias.

Study 30-2

Based on the information provided in the article, here is my assessment of the risk of bias:

**Article ID**: Kessler, 2018

**Outcome Name**: Health-related quality of life

**Registration number**: NCT01241526

Domain 1: Risk of bias arising from the randomization process

1.1: NI (Low Certainty, 60%)

Reason: The article does not provide specific details about the sequence generation method. It only mentions a "pre-specified randomisation list generated before the study by a partial-minimisation computer algorithm".

1.2: Y (High Certainty, 95%)

Reason: The article states "Patients were assigned a randomisation number by study staff at each centre in sequential numerical order through a telephone-based interactive voice response system."

1.3: N (Moderate Certainty, 80%)

Reason: The article reports that "Patients in both groups at baseline were similar for most characteristics".

Result of Domain 1: Low risk (Moderate Certainty, 80%)

Reason: While the sequence generation method is unclear, allocation concealment was well described and baseline differences do not suggest problems with randomization.

Domain 2: Risk of bias due to deviations from intended interventions

2.1: Y (High Certainty, 95%)

Reason: The study was open-label, so participants were aware of their assigned intervention.

2.2: Y (High Certainty, 95%)

Reason: As an open-label study, care providers were also aware of participants' assigned interventions.

2.3: NI (Low Certainty, 50%)

Reason: The article does not provide information on non-protocol interventions.

2.4: N (Moderate Certainty, 80%)

Reason: The article reports high compliance with the intervention, suggesting no major implementation failures.

2.5: N (Moderate Certainty, 80%)

Reason: The article reports that "66.7% of patients achieved at least 80% of their planned phone and group coaching sessions, and 89.7% achieved at least 80% compliance for weekly telephone health status transmissions."

2.6: N (High Certainty, 95%)

Reason: The article states that analyses were performed on the intention-to-treat population.

Result of Domain 2: Some concerns (Moderate Certainty, 75%)

Reason: While the study was open-label, there is no clear evidence of deviations from intended interventions that likely affected the outcome.

Domain 3: Risk of bias due to missing outcome data

3.1: Y (High Certainty, 90%)

Reason: The article reports that 137/157 (87%) patients in the disease management group and 128/162 (79%) in the usual management group completed at least 12 months of follow-up.

3.2: N (Moderate Certainty, 70%)

Reason: The article does not report using methods to correct for bias due to missing data.

3.3: PY (Low Certainty, 60%)

Reason: Given the severity of COPD in the study population, it's possible that missing data could be related to health status.

3.4: NI (Very Low Certainty, 40%)

Reason: The article does not provide enough information to assess whether missingness in the outcome depended on its true value.

Result of Domain 3: Some concerns (Low Certainty, 65%)

Reason: While data was available for most participants, there is a possibility that missing data could be related to the true outcome value.

Domain 4: Risk of bias in measurement of the outcome

4.1: N (High Certainty, 90%)

Reason: Health-related quality of life was measured using the SGRQ-C, which is an appropriate and validated tool.

4.2: N (High Certainty, 90%)

Reason: The article does not suggest any differences in outcome measurement between groups.

4.3: Y (High Certainty, 95%)

Reason: As an open-label study, outcome assessors were likely aware of intervention status.

4.4: Y (High Certainty, 90%)

Reason: Health-related quality of life is a subjective measure reported by patients who were aware of their intervention status.

4.5: N (Low Certainty, 60%)

Reason: While the outcome is subjective, there is no clear evidence that assessment was influenced by knowledge of the intervention received.

Result of Domain 4: High risk (Moderate Certainty, 85%)

Reason: The outcome is subjective and was assessed by participants who were aware of their intervention status.

Domain 5: Risk of bias in selection of the reported result

5.1: Y (High Certainty, 90%)

Reason: The study was registered (NCT01241526) and the reported outcomes align with those pre-specified.

5.2: N (Moderate Certainty, 80%)

Reason: The article reports the SGRQ-C total score as pre-specified, without evidence of selective reporting from multiple outcome measurements.

5.3: N (Moderate Certainty, 80%)

Reason: The article reports the analysis as pre-specified, without evidence of selection from multiple analyses.

Result of Domain 5: Low risk (High Certainty, 85%)

Reason: The study was pre-registered and reported outcomes align with those pre-specified without evidence of selective reporting.

Overall Bias Risk for the Article: High risk (Moderate Certainty, 80%)

Reason: While some domains show low risk of bias, the high risk in measurement of the outcome (due to the subjective nature of the outcome and lack of blinding) leads to an overall assessment of high risk of bias.

Study 31-1

Based on the information provided in the article, here is my assessment of the risk of bias:

**Article ID**: Lenferink, 2019

**Outcome Name**: COPD exacerbation days

**Registration number**: ACTRN12612000514808

Domain 1: Risk of bias arising from the randomization process

1.1: Y (High Certainty, 95%)

Reason: The article states "using a computerised minimisation program" for randomization, which is an adequate method of sequence generation.

1.2: Y (High Certainty, 95%)

Reason: The article mentions "an independent research assistant, who was blinded to the treatment assignment and randomisation schedule", indicating proper allocation concealment.

1.3: N (Moderate Certainty, 80%)

Reason: The baseline characteristics were generally similar between groups, with only the 6-minute walk distance showing a significant difference.

Result of Domain 1: Low risk (High Certainty, 95%)

Reason: Adequate randomization and allocation concealment, with only minor baseline differences.

Domain 2: Risk of bias due to deviations from intended interventions

2.1: Y (High Certainty, 95%)

Reason: Due to the nature of the self-management intervention, participants were aware of their assigned intervention.

2.2: Y (High Certainty, 95%)

Reason: The intervention providers were aware of the participants' allocation as they delivered the self-management intervention.

2.3: NI (Low Certainty, 60%)

Reason: The article does not provide clear information about non-protocol interventions.

2.4: N (Moderate Certainty, 80%)

Reason: The article does not report significant failures in implementing the intervention.

2.5: N (Moderate Certainty, 80%)

Reason: The article does not report significant non-adherence to the assigned intervention.

2.6: N (High Certainty, 95%)

Reason: The article states "Analyses were conducted on an intention-to-treat basis", which is not an appropriate method to estimate the effect of adhering to the intervention.

Result of Domain 2: Some concerns (Moderate Certainty, 80%)

Reason: While participants and providers were aware of the intervention, there's no evidence of significant deviations from intended interventions.

Domain 3: Risk of bias due to missing outcome data

3.1: Y (High Certainty, 95%)

Reason: The article reports that 83.3% of the self-management group and 84.8% of the usual care group completed the 12-month follow-up, which is over 95% of randomized participants.

3.2: Y (Moderate Certainty, 80%)

Reason: The article describes using negative binomial regression imputation for missing diary data, which can help correct for bias.

3.3: N (Moderate Certainty, 80%)

Reason: There's no clear indication that missingness depends on the true value of the outcome.

3.4: N (Moderate Certainty, 80%)

Reason: No evidence suggests that missingness depends on the true value of the outcome.

Result of Domain 3: Low risk (High Certainty, 95%)

Reason: High follow-up rates and appropriate handling of missing data.

Domain 4: Risk of bias in measurement of the outcome

4.1: N (High Certainty, 95%)

Reason: The method of measuring COPD exacerbation days using daily symptom diaries is appropriate.

4.2: N (High Certainty, 95%)

Reason: The measurement of the outcome was consistent across intervention groups.

4.3: Y (High Certainty, 95%)

Reason: Participants were aware of their intervention status and reported their symptoms in daily diaries.

4.4: Y (High Certainty, 95%)

Reason: The outcome (COPD exacerbation days) is based on patient-reported symptoms, which could be influenced by knowledge of the intervention.

4.5: N (Moderate Certainty, 80%)

Reason: While the outcome is subjective, there's no clear evidence that assessment was influenced by knowledge of the intervention.

Result of Domain 4: Some concerns (Moderate Certainty, 80%)

Reason: While the outcome measurement was appropriate, it's based on patient-reported symptoms which could be influenced by knowledge of the intervention.

Domain 5: Risk of bias in selection of the reported result

5.1: Y (High Certainty, 95%)

Reason: The study was registered (ACTRN12612000514808) and the reported analyses align with the pre-specified plans.

5.2: N (High Certainty, 95%)

Reason: The reported outcome measurements align with those pre-specified in the methods.

5.3: N (High Certainty, 95%)

Reason: The reported analyses align with those pre-specified in the methods.

Result of Domain 5: Low risk (High Certainty, 95%)

Reason: The reported results align with the pre-specified plans in the study registration.

Overall Bias Risk for the Article: Some concerns (Moderate Certainty, 80%)

Reason: While there are low risks of bias in the randomization process, missing data handling, and selection of reported results, there are some concerns regarding the potential influence of participants' knowledge of the intervention on the self-reported outcome measure.

Study 31-2

Based on the information provided in the article, here is my assessment of the risk of bias:

**Article ID**: Lenferink, 2019

**Outcome Name**: Health-related quality of life

**Registration number**: ACTRN12612000514808

Domain 1

1.1: Y (95% confidence)

Reason: The study used computerized minimisation for allocation, which is an adequate method of sequence generation.

1.2: Y (95% confidence)

Reason: An independent research assistant blinded to treatment assignment performed the allocation using a computerized program, indicating adequate concealment.

1.3: N (90% confidence)

Reason: Baseline characteristics were generally similar between groups, with the exception of 6-minute walk distance. This difference is likely due to chance rather than a problem with randomization.

Result of Domain 1: Low risk (90% confidence)

Reason: Adequate randomization and allocation concealment, with only minor baseline imbalances likely due to chance.

Domain 2

2.1: Y (95% confidence)

Reason: Due to the nature of the self-management intervention, blinding of participants was not possible.

2.2: Y (95% confidence)

Reason: The study states that blinding of personnel to treatment group was not possible.

2.3: PY (80% confidence)

Reason: Both groups received usual care, but the self-management group received additional education and support. No major non-protocol interventions are described.

2.4: N (85% confidence)

Reason: There is no indication of major implementation failures. Dropout rates were similar and relatively low in both groups.

2.5: PN (80% confidence)

Reason: Some non-adherence likely occurred but appears to be <10% based on high diary completion rates and similar dropout between groups.

2.6: N (90% confidence)

Reason: The study used intention-to-treat analysis, which is not an appropriate method to estimate the effect of adhering to the intervention.

Result of Domain 2: Some concerns (85% confidence)

Reason: Participants and personnel were aware of intervention status, but other bias risks appear low.

Domain 3

3.1: Y (95% confidence)

Reason: Over 80% of participants completed follow-up in both groups.

3.2: NA

Reason: Not applicable as missing data was minimal.

3.3: NA

Reason: Not applicable as missing data was minimal.

3.4: NA

Reason: Not applicable as missing data was minimal.

Result of Domain 3: Low risk (95% confidence)

Reason: High follow-up rates with minimal missing data.

Domain 4

4.1: N (90% confidence)

Reason: Validated questionnaires were used to measure health-related quality of life outcomes.

4.2: N (90% confidence)

Reason: The same measurement methods were used for both groups.

4.3: Y (95% confidence)

Reason: Health-related quality of life was measured by patient-reported questionnaires, and participants were aware of their group assignment.

4.4: Y (95% confidence)

Reason: Health-related quality of life is a subjective outcome that could be influenced by knowledge of the intervention received.

4.5: PN (80% confidence)

Reason: While patients' knowledge of intervention could influence responses, there is no clear evidence that it substantially impacted assessment.

Result of Domain 4: Some concerns (85% confidence)

Reason: While measurement methods were appropriate, the subjective nature of the outcome and lack of blinding creates some risk of bias.

Domain 5

5.1: Y (95% confidence)

Reason: The study was prospectively registered and outcomes appear to be analyzed as pre-specified.

5.2: N (90% confidence)

Reason: The reported outcomes match those pre-specified in the methods.

5.3: N (90% confidence)

Reason: The analyses appear to match those pre-specified in the methods.

Result of Domain 5: Low risk (90% confidence)

Reason: Pre-specified outcomes and analyses were followed with no evidence of selective reporting.

Overall Bias Risk for the Article: Some concerns (85% confidence)

Reason: While randomization, allocation concealment, and handling of missing data were strong, the inability to blind participants and subjective nature of the primary outcome create some risk of bias. However, these concerns are somewhat mitigated by the prospective registration and adherence to the pre-specified analysis plan.

Study 32-1

**Article ID**: Liang, 2019

**Outcome Name**: Health-related quality of life (SGRQ score)

**registration number**: ACTRN12614001155684

Domain 1

1.1: Y (95%)

Reason: The study used block randomization with an externally managed web-based randomization programme.

1.2: Y (95%)

Reason: An externally managed web-based randomization program was used, implying adequate allocation concealment.

1.3: N (80%)

Reason: Baseline characteristics were generally similar between groups, with only minor differences.

Result of the domain: Low risk (95%)

Reason: Adequate randomization process with no major concerns.

Domain 2

2.1: Y (95%)

Reason: Open-label study, participants were aware of their assigned intervention.

2.2: Y (95%)

Reason: Open-label study, care providers were aware of participants' assigned interventions.

2.3: NI (60%)

Reason: No clear information on non-protocol interventions provided.

2.4: Y (95%)

Reason: Low uptake of the intervention, with only 31% of participants completing the full intervention.

2.5: Y (95%)

Reason: Low uptake (31% full intervention completion) indicates substantial non-adherence.

2.6: N (95%)

Reason: The primary analysis was conducted using intention-to-treat analysis.

Result of the domain: High risk (95%)

Reason: Substantial deviations from intended interventions due to low uptake and adherence.

Domain 3

3.1: PN (80%)

Reason: At 6 months, 75% in intervention and 82% in usual care had SGRQ data, slightly below 95% threshold.

3.2: Y (95%)

Reason: Multiple imputation was used to handle missing data.

3.3: NI (60%)

Reason: No clear information on reasons for missing data in relation to the outcome.

3.4: NI (60%)

Reason: Insufficient information to determine if missingness depended on true value.

Result of the domain: Some concerns (80%)

Reason: Some missing data, but appropriate methods used to handle it. Reasons for missingness unclear.

Domain 4

4.1: N (95%)

Reason: SGRQ is a validated instrument for measuring health-related quality of life in COPD.

4.2: N (95%)

Reason: SGRQ would have been administered similarly in both groups.

4.3: N (95%)

Reason: Outcome assessments were performed by research assistants blinded to group allocation.

4.4: Y (95%)

Reason: SGRQ is a subjective patient-reported outcome that could potentially be influenced by knowledge of intervention.

4.5: N (80%)

Reason: No clear evidence that assessment was influenced by knowledge of intervention received.

Result of the domain: Low risk (90%)

Reason: Appropriate outcome measure and blinded outcome assessment.

Domain 5

5.1: Y (95%)

Reason: Study was prospectively registered and outcomes appear to be reported as pre-specified.

5.2: N (95%)

Reason: SGRQ results are reported comprehensively using the total score.

5.3: N (95%)

Reason: No evidence of selective reporting from multiple analyses.

Result of the domain: Low risk (95%)

Reason: Results reported as pre-specified without evidence of selection based on results.

Overall Bias Risk for the Article: High risk (90%)

Reason: High risk of bias due to substantial deviations from intended interventions, primarily due to low uptake and adherence to the intervention.

Study 32-2

**Article ID**: Liang, 2019

**Outcome Name**: Health-related quality of life (SGRQ score)

**registration number**: ACTRN12614001155684

Domain 1

1.1: Y (95%)

Reason: The study used block randomization with an externally managed web-based randomization programme.

1.2: Y (95%)

Reason: An externally managed web-based randomization program was used, implying adequate allocation concealment.

1.3: N (80%)

Reason: Baseline characteristics were generally similar between groups, with only minor differences.

Result of the domain: Low risk (95%)

Reason: Adequate randomization process with no major concerns.

Domain 2

2.1: Y (95%)

Reason: Open-label study, participants were aware of their assigned intervention.

2.2: Y (95%)

Reason: Open-label study, care providers were aware of participants' assigned interventions.

2.3: NI (60%)

Reason: No clear information on non-protocol interventions provided.

2.4: Y (95%)

Reason: Low uptake of the intervention, with only 31% of participants completing the full intervention.

2.5: Y (95%)

Reason: Low uptake (31% full intervention completion) indicates substantial non-adherence.

2.6: N (95%)

Reason: The primary analysis was conducted using intention-to-treat analysis.

Result of the domain: High risk (95%)

Reason: Substantial deviations from intended interventions due to low uptake and adherence.

Domain 3

3.1: PN (80%)

Reason: At 6 months, 75% in intervention and 82% in usual care had SGRQ data, slightly below 95% threshold.

3.2: Y (95%)

Reason: Multiple imputation was used to handle missing data.

3.3: NI (60%)

Reason: No clear information on reasons for missing data in relation to the outcome.

3.4: NI (60%)

Reason: Insufficient information to determine if missingness depended on true value.

Result of the domain: Some concerns (80%)

Reason: Some missing data, but appropriate methods used to handle it. Reasons for missingness unclear.

Domain 4

4.1: N (95%)

Reason: SGRQ is a validated instrument for measuring health-related quality of life in COPD.

4.2: N (95%)

Reason: SGRQ would have been administered similarly in both groups.

4.3: N (95%)

Reason: Outcome assessments were performed by research assistants blinded to group allocation.

4.4: Y (95%)

Reason: SGRQ is a subjective patient-reported outcome that could potentially be influenced by knowledge of intervention.

4.5: N (80%)

Reason: No clear evidence that assessment was influenced by knowledge of intervention received.

Result of the domain: Low risk (90%)

Reason: Appropriate outcome measure and blinded outcome assessment.

Domain 5

5.1: Y (95%)

Reason: Study was prospectively registered and outcomes appear to be reported as pre-specified.

5.2: N (95%)

Reason: SGRQ results are reported comprehensively using the total score.

5.3: N (95%)

Reason: No evidence of selective reporting from multiple analyses.

Result of the domain: Low risk (95%)

Reason: Results reported as pre-specified without evidence of selection based on results.

Overall Bias Risk for the Article: High risk (90%)

Reason: High risk of bias due to substantial deviations from intended interventions, primarily due to low uptake and adherence to the intervention.

Study 33-1

Based on the instructions provided and the information in the article, here is my assessment:

**Article ID**: Mitchell, 2014

**Outcome Name**: Health-related quality of life

**registration number**: ISRCTN35501175

Domain 1

1.1: Y (95%)

Reason: The article states "Participants were assigned to either usual care or SPACE FOR COPD via a web-based, concealed allocation programme, using simple randomisation codes prepared by the trial statistician"

1.2: Y (95%)

Reason: The article mentions "web-based, concealed allocation programme"

1.3: N (90%)

Reason: The article states "The only significant baseline difference between treatment groups was the CRQ-SR dyspnoea score; therefore, this was corrected for in subsequent analyses."

Result of the domain: Low risk (90%)

Reason: 1.2 is Y, 1.1 is Y, and 1.3 is N

Domain 2

2.1: Y (95%)

Reason: The study was not blinded - "single-blind randomised controlled trial"

2.2: Y (95%)

Reason: As a single-blind study, care providers were aware of intervention assignment

2.3: NI (80%)

Reason: No information provided about non-protocol interventions

2.4: N (90%)

Reason: No indication of substantial implementation failures

2.5: NI (90%)

Reason: The article does not report that more than 10% of participants had non-adherence to the assigned intervention

2.6: N (95%)

Reason: The article states "Analysis was carried out on an intention-to-treat basis"

Result of the domain: High risk (90%)

Reason: 2.1 and 2.2 are Y, 2.3 is NI, 2.4 and 2.5 are N, 2.6 is N

Domain 3

3.1: Y (95%)

Reason: The article states "There were no significant differences in demographics or baseline variables between those who completed and those who did not complete the study"

3.2: Y (90%)

Reason: The article states "Missing data were imputed in Stata (version 12; StataCorp, College Station, TX, USA) using multiple imputed chained equations"

3.3: N (90%)

Reason: No indication that missingness depended on true values

3.4: N (90%)

Reason: No indication that missingness likely depended on true values

Result of the domain: Low risk (90%)

Reason: 3.1 is Y

Domain 4

4.1: N (95%)

Reason: The CRQ-SR is an appropriate measure for health-related quality of life in COPD

4.2: N (95%)

Reason: No indication of differences in measurement between groups

4.3: Y (95%)

Reason: As a single-blind study, outcome assessors were likely aware of intervention assignment

4.4: Y (95%)

Reason: Health-related quality of life is a subjective measure that could be influenced by knowledge of intervention

4.5: N (90%)

Reason: No clear evidence that assessment was influenced by knowledge of intervention

Result of the domain: Some concerns (90%)

Reason: 4.1 and 4.2 are N, 4.3 and 4.4 are Y, 4.5 is N

Domain 5

5.1: Y (95%)

Reason: The trial was registered (ISRCTN35501175) and the article states "The primary pre-specified outcome was the change in the CRQ-SR dyspnoea domain from baseline to 6 months"

5.2: N (90%)

Reason: No indication of selective reporting from multiple outcome measurements

5.3: N (90%)

Reason: No indication of selective reporting from multiple analyses

Result of the domain: Low risk (90%)

Reason: 5.1 is Y, 5.2 and 5.3 are N

Overall Bias Risk for the Article: High risk (90%)

Reason: Domain 2 is high risk

Study 33-2

Based on the instructions provided and the information in the article, here is my assessment:

**Article ID**: Mitchell, 2014

**Outcome Name**: Health-related quality of life

**registration number**: ISRCTN35501175

Domain 1

1.1: Y (95%)

Reason: The article states "Participants were assigned to either usual care or SPACE FOR COPD via a web-based, concealed allocation programme, using simple randomisation codes prepared by the trial statistician"

1.2: Y (95%)

Reason: The article mentions "web-based, concealed allocation programme"

1.3: N (90%)

Reason: The article states "The only significant baseline difference between treatment groups was the CRQ-SR dyspnoea score; therefore, this was corrected for in subsequent analyses."

Result of the domain: Low risk (90%)

Reason: 1.2 is Y, 1.1 is Y, and 1.3 is N

Domain 2

2.1: Y (95%)

Reason: The study was not blinded - "single-blind randomised controlled trial"

2.2: Y (95%)

Reason: As a single-blind study, care providers were aware of intervention assignment

2.3: NI (80%)

Reason: No information provided about non-protocol interventions

2.4: N (90%)

Reason: No indication of substantial implementation failures

2.5: N (90%)

Reason: No indication of substantial non-adherence

2.6: N (95%)

Reason: The article states "Analysis was carried out on an intention-to-treat basis"

Result of the domain: High risk (90%)

Reason: 2.1 and 2.2 are Y, 2.3 is NI, 2.4 and 2.5 are N, 2.6 is N

Domain 3

3.1: Y (95%)

Reason: The article states "There were no significant differences in demographics or baseline variables between those who completed and those who did not complete the study"

3.2: Y (90%)

Reason: The article states "Missing data were imputed in Stata (version 12; StataCorp, College Station, TX, USA) using multiple imputed chained equations"

3.3: N (90%)

Reason: No indication that missingness depended on true values

3.4: N (90%)

Reason: No indication that missingness likely depended on true values

Result of the domain: Low risk (90%)

Reason: 3.1 is Y

Domain 4

4.1: N (95%)

Reason: The CRQ-SR is an appropriate measure for health-related quality of life in COPD

4.2: N (95%)

Reason: No indication of differences in measurement between groups

4.3: Y (95%)

Reason: As a single-blind study, outcome assessors were likely aware of intervention assignment

4.4: Y (95%)

Reason: Health-related quality of life is a subjective measure that could be influenced by knowledge of intervention

4.5: N (90%)

Reason: No clear evidence that assessment was influenced by knowledge of intervention

Result of the domain: Some concerns (90%)

Reason: 4.1 and 4.2 are N, 4.3 and 4.4 are Y, 4.5 is N

Domain 5

5.1: Y (95%)

Reason: The trial was registered (ISRCTN35501175) and the article states "The primary pre-specified outcome was the change in the CRQ-SR dyspnoea domain from baseline to 6 months"

5.2: N (90%)

Reason: No indication of selective reporting from multiple outcome measurements

5.3: N (90%)

Reason: No indication of selective reporting from multiple analyses

Result of the domain: Low risk (90%)

Reason: 5.1 is Y, 5.2 and 5.3 are N

Overall Bias Risk for the Article: High risk (90%)

Reason: Domain 2 is high risk

Study 34-1

Article ID: Oborne, 2009

Outcome Name: Treatment failure: need for systemic corticosteroids (of those starting inhaler)

registration number: ISRCTN 46018181

Domain 1

1.1: Y (95%)

Reason: The study used an allocation sequence of random permuted blocks generated using a random number table.

1.2: Y (95%)

Reason: Allocation concealment was achieved using identical active and placebo inhalers.

1.3: N (80%)

Reason: Baseline characteristics were well matched between groups, with only a slight difference in proportion of ever smokers.

Result of the domain: Low risk (95%)

Reason: Proper randomization and allocation concealment were used, with no major baseline imbalances.

Domain 2

2.1: N (95%)

Reason: The study was double-blind, so participants were unaware of their assigned intervention.

2.2: N (95%)

Reason: The study was double-blind, so people delivering interventions were unaware of participants' assigned intervention.

2.3: Y (90%)

Reason: No additional non-protocol interventions were mentioned or likely given the nature of the intervention.

2.4: N (90%)

Reason: No major implementation failures are reported among those who started the inhaler.

2.5: NI (80%)

Reason: No specific information reported regarding adherence in the article for those who started the inhaler.

2.6: N (95%)

Reason: The study used per-protocol analysis for this outcome.

Result of the domain: Some concerns (85%)

Reason: While the study was double-blind, there's no clear information on adherence for those who started the inhaler.

Domain 3

3.1: N (90%)

Reason: This outcome only includes participants who started the inhaler, which is not all randomized participants.

3.2: N (80%)

Reason: No evidence is provided that results were not biased by missing data.

3.3: Y (80%)

Reason: Missingness may depend on the true outcome, as only those who started the inhaler are included.

3.4: PY (75%)

Reason: It's plausible that those who didn't start the inhaler had better asthma control.

Result of the domain: High risk (80%)

Reason: This outcome only includes a subset of randomized participants, potentially introducing bias.

Domain 4

4.1: N (95%)

Reason: The method of measuring the outcome (need for systemic corticosteroids) is appropriate.

4.2: N (95%)

Reason: The outcome measurement method was consistent across intervention groups.

4.3: N (95%)

Reason: The study was double-blind, so outcome assessors were unaware of the intervention received.

4.4: N (90%)

Reason: The need for systemic corticosteroids is a relatively objective measure unlikely to be influenced by knowledge of the intervention.

4.5: N (90%)

Reason: No clear evidence that assessment of the outcome was influenced by knowledge of intervention received.

Result of the domain: Low risk (95%)

Reason: Outcome measurement was appropriate, consistent, and blinded, with low risk of bias.

Domain 5

5.1: Y (90%)

Reason: The study was registered (ISRCTN 46018181) and this outcome was pre-specified.

5.2: N (90%)

Reason: The reported outcome measurements align with those pre-specified in the methods.

5.3: N (90%)

Reason: The reported analysis is consistent with the pre-specified plan.

Result of the domain: Low risk (90%)

Reason: The study was pre-registered and reported results align with the pre-specified plan.

Overall Bias Risk for the Article: High risk (85%)

Reason: While most domains show low risk of bias, the high risk in Domain 3 due to the selective inclusion of participants who started the inhaler raises concerns about the overall validity of this specific outcome.

Study 34-2

Article ID: Oborne, 2009

Outcome Name: Treatment failure: need for systemic corticosteroids (of those starting inhaler)

registration number: ISRCTN 46018181

Domain 1

1.1 : Y (95% Confidence)

Reason: The study used "an allocation sequence of random permuted blocks of 10 generated using a random number table by an independent pharmacist".

1.2 : Y (95% Confidence)

Reason: The study mentions "allocation concealment" and used identical active and placebo inhalers.

1.3 : N (90% Confidence)

Reason: Baseline characteristics were well-matched between groups, with only a slight difference in smoking history.

Result of the domain: Low risk (95% Confidence)

Reason: Proper randomization and allocation concealment methods were used, with no concerning baseline differences between groups.

Domain 2

2.1 : N (95% Confidence)

Reason: The study was described as "double-blind".

2.2 : N (95% Confidence)

Reason: The study was "double-blind" and used identical active and placebo inhalers.

2.3 : NI (80% Confidence)

Reason: The article does not provide information on non-protocol interventions.

2.4 : N (90% Confidence)

Reason: No significant failures in implementing the intervention are reported affecting >10% of participants who started the inhaler.

2.5 : NI (90% Confidence)

Reason: No specific information is reported regarding adherence to the assigned intervention for those who started the inhaler.

2.6 : N (95% Confidence)

Reason: The study states they used per-protocol analysis for this outcome.

Result of the domain: Some concerns (85% Confidence)

Reason: While the study was double-blind, there's no information on adherence for those who started the inhaler, and a per-protocol analysis was used.

Domain 3

3.1 : N (95% Confidence)

Reason: This outcome is only for those who started the inhaler (94 out of 403 participants), so data is not available for all randomized participants.

3.2 : N (90% Confidence)

Reason: The study used per-protocol analysis for this outcome, which doesn't account for missing data from those who didn't start the inhaler.

3.3 : Y (85% Confidence)

Reason: Missingness likely depends on the true outcome, as those who didn't start the inhaler may have had different outcomes.

3.4 : Y (85% Confidence)

Reason: It's likely that missingness depended on the true outcome value, as those who didn't start the inhaler may have had different outcomes.

Result of the domain: High risk (90% Confidence)

Reason: This outcome only includes a subset of randomized participants, likely introducing bias.

Domain 4

4.1 : N (95% Confidence)

Reason: The outcome (need for systemic corticosteroids) is appropriate and clearly defined.

4.2 : N (95% Confidence)

Reason: The study was double-blind with identical inhalers, ensuring consistent outcome measurement across groups.

4.3 : N (95% Confidence)

Reason: The study was double-blind, so outcome assessors were unaware of intervention assignments.

4.4 : N (90% Confidence)

Reason: The outcome is relatively objective and unlikely to be influenced by knowledge of the intervention.

4.5 : N (90% Confidence)

Reason: There is no clear evidence that assessment of the outcome was influenced by knowledge of the intervention received.

Result of the domain: Low risk (95% Confidence)

Reason: The outcome was appropriate, objectively measured, and assessed in a blinded manner.

Domain 5

5.1 : Y (95% Confidence)

Reason: The study was registered (ISRCTN 46018181) and the analysis plan was pre-specified.

5.2 : N (90% Confidence)

Reason: The reported outcome measures align with those described in the methods, with no evidence of selection based on results.

5.3 : N (90% Confidence)

Reason: There is no evidence of multiple analyses of the data with selective reporting of results.

Result of the domain: Low risk (90% Confidence)

Reason: The study was pre-registered and there's no evidence of selective reporting.

Overall Bias Risk for the Article: High risk (90% Confidence)

Reason: While most domains show low risk of bias, the high risk in Domain 3 due to missing outcome data for a large portion of randomized participants raises significant concerns about the overall reliability of the results for this specific outcome.

Study 35-1

Based on the information provided in the article, here is my assessment of the risk of bias for the specified outcome:

**Article ID**: Rice, 2010

**Outcome Name**: Health-related quality of life

**registration number**: NCT00126776

Domain 1: Risk of bias arising from the randomization process

1.1: Y (High Certainty - 95%)

Reason: The article states "Patients with COPD were randomly assigned to disease management or usual care."

1.2: NI (High Certainty - 95%)

Reason: The article does not provide information about allocation concealment.

1.3: N (Moderate Certainty - 85%)

Reason: Table 1 shows baseline characteristics were generally well matched between groups, with only small differences in age and lung function noted.

Result of Domain 1: Some concerns (High Certainty - 95%)

Reason: While randomization was performed, allocation concealment was not described, leading to some concerns about the randomization process.

Domain 2: Risk of bias due to deviations from the intended interventions

2.1: Y (High Certainty - 95%)

Reason: Patients were aware of their intervention assignment as the disease management group received education and an action plan.

2.2: Y (High Certainty - 95%)

Reason: Care providers were aware of intervention assignments as they delivered the disease management program.

2.3: NI (Moderate Certainty - 80%)

Reason: The article does not provide information about non-protocol interventions.

2.4: N (Moderate Certainty - 80%)

Reason: The article does not report significant failures in implementing the intervention.

2.5: NI (Moderate Certainty - 80%)

Reason: The article does not report significant non-adherence to the assigned interventions.

2.6: N (High Certainty - 95%)

Reason: The analysis appears to be intention-to-treat based on the number of patients analyzed.

Result of Domain 2: Some concerns (High Certainty - 90%)

Reason: While participants and providers were aware of assignments, there is no clear evidence of deviation from intended interventions.

Domain 3: Missing outcome data

3.1: N (High Certainty - 95%)

Reason: Only 55% of usual care and 60% of disease management patients completed the quality of life questionnaire.

3.2: N (High Certainty - 95%)

Reason: No evidence is provided that results were not biased by missing data.

3.3: PY (Moderate Certainty - 80%)

Reason: Missingness could depend on the true value, as those with worse health may have been less likely to complete the questionnaire.

3.4: NI (Low Certainty - 60%)

Reason: Insufficient information to determine if missingness depended on its true value.

Result of Domain 3: High risk (High Certainty - 90%)

Reason: There was a high proportion of missing outcome data that could be related to the true outcome value.

Domain 4: Risk of bias in measurement of the outcome

4.1: N (High Certainty - 95%)

Reason: The St. George's Respiratory Questionnaire is an appropriate measure for health-related quality of life in COPD.

4.2: N (High Certainty - 95%)

Reason: The measurement method was the same for both groups.

4.3: Y (High Certainty - 95%)

Reason: The outcome was patient-reported and patients were aware of their intervention assignment.

4.4: Y (High Certainty - 95%)

Reason: Health-related quality of life is a subjective measure reported by patients who were aware of their intervention.

4.5: Y (Moderate Certainty - 80%)

Reason: Knowledge of the intervention received could influence patients' self-reported quality of life.

Result of Domain 4: High risk (High Certainty - 90%)

Reason: The outcome measure was subjective and patients were aware of their intervention assignment.

Domain 5: Risk of bias in selection of the reported result

5.1: Y (High Certainty - 95%)

Reason: The study was registered (NCT00126776) and the outcome was pre-specified.

5.2: N (High Certainty - 95%)

Reason: There is no evidence of selection from multiple outcome measurements.

5.3: N (High Certainty - 95%)

Reason: There is no evidence of selection from multiple analyses.

Result of Domain 5: Low risk (High Certainty - 95%)

Reason: The outcome was pre-specified and there is no evidence of selective reporting.

Overall Bias Risk for the Article: High risk (High Certainty - 90%)

The overall risk of bias is high primarily due to the high risk of bias from missing outcome data and measurement of the outcome. While the randomization process and intervention implementation appear to have been conducted appropriately, the subjective nature of the outcome measure combined with patients' awareness of their treatment assignment and the high proportion of missing data contribute to a high risk of bias for this outcome.

Study 35-2

Based on the provided information and instructions, here is my assessment of the risk of bias for the Rice et al. 2010 study:

**Article ID**: Rice, 2010

**Outcome Name**: Health-related quality of life

**registration number**: NCT00126776

Domain 1

1.1: Y (95% Confidence)

Reason: The study states "Patients were randomly assigned to disease management or usual care."

1.2: Y (95% Confidence)

Reason: The study mentions "central allocation" which indicates allocation was concealed.

1.3: N (90% Confidence)

Reason: Baseline characteristics were generally well-matched between groups, with only small differences noted in age and lung function.

Result of Domain 1: Low risk (95% Confidence)

Reason: Allocation sequence was adequately generated and concealed, and baseline differences do not suggest problems with randomization.

Domain 2

2.1: Y (95% Confidence)

Reason: Patients in the disease management group attended an education session and received an action plan, indicating they were aware of their assignment.

2.2: Y (95% Confidence)

Reason: The intervention involved case managers providing education and follow-up, indicating they were aware of assignments.

2.3: NI (90% Confidence)

Reason: The study does not provide clear information on non-protocol interventions across groups.

2.4: N (90% Confidence)

Reason: There is no indication of substantial failures in implementing the intervention.

2.5: NI (90% Confidence)

Reason: No information reported regarding adherence in the article.

2.6: N (95% Confidence)

Reason: The analysis appears to be intention-to-treat, which is not considered an appropriate analysis to estimate the effect of adhering to the intervention.

Result of Domain 2: Low risk (90% Confidence)

Reason: While participants and providers were aware of assignments, there is no evidence of substantial deviations from intended interventions.

Domain 3

3.1: PN (90% Confidence)

Reason: 55-60% of patients returned the quality of life questionnaire, indicating less than 95% but likely more than 90% completion.

3.2: N (90% Confidence)

Reason: The study does not report using methods to correct for potential bias from missing data.

3.3: PY (85% Confidence)

Reason: Given the nature of the outcome (quality of life), missingness may depend on its true value.

3.4: NI (80% Confidence)

Reason: There is insufficient information to determine if missingness in the outcome likely depended on its true value.

Result of Domain 3: Some concerns (85% Confidence)

Reason: There was substantial missing data for the quality of life outcome, and it's possible the missingness depended on true values.

Domain 4

4.1: N (95% Confidence)

Reason: The St. George's Respiratory Questionnaire is an appropriate and validated measure for respiratory health status.

4.2: N (95% Confidence)

Reason: The same questionnaire was used for both groups.

4.3: Y (95% Confidence)

Reason: Quality of life was self-reported by patients who were aware of their treatment assignment.

4.4: Y (95% Confidence)

Reason: Quality of life is a subjective measure reported by patients.

4.5: N (85% Confidence)

Reason: While patients were aware of their assignment, there is no clear evidence that this knowledge likely influenced their quality of life assessments.

Result of Domain 4: Some concerns (90% Confidence)

Reason: While the measurement method was appropriate, patients were aware of their assignment which could potentially influence their self-reported outcomes.

Domain 5

5.1: Y (95% Confidence)

Reason: The study was registered (NCT00126776) and the reported outcomes align with those pre-specified.

5.2: N (90% Confidence)

Reason: There's no evidence of selective reporting from multiple outcome measurements.

5.3: N (90% Confidence)

Reason: There's no evidence of selective reporting from multiple analyses.

Result of Domain 5: Low risk (90% Confidence)

Reason: The study appears to have followed a pre-specified analysis plan without selective reporting.

Overall Bias Risk for the Article: Some concerns (90% Confidence)

Reason: While randomization and intervention implementation appear sound, there are some concerns regarding missing outcome data and potential bias in the self-reported quality of life measure due to participants' awareness of their treatment assignment.

Study 36-1

Based on the information provided in the article, here is my assessment of the risk of bias:

**Article ID**: Rice-McDonald, 2005

**Outcome Name**: Treatment failure

**registration number**: Not reported

Domain 1: Risk of bias arising from the randomization process

1.1: Y (95% confidence)

Reason: The study is described as randomized with concealed allocation, implying an adequate randomization method was used.

1.2: Y (95% confidence)

Reason: The study mentions "concealed randomisation", indicating allocation was concealed.

1.3: N (90% confidence)

Reason: No significant baseline differences between groups are reported that would suggest a problem with randomization.

Result of Domain 1: Low risk (95% confidence)

Reason: Allocation sequence was adequately generated and concealed, and baseline differences do not suggest problems with randomization.

Domain 2: Risk of bias due to deviations from intended interventions

2.1: N (90% confidence)

Reason: The study is described as double-blind, indicating participants were unaware of their assigned intervention.

2.2: N (90% confidence)

Reason: The study is described as double-blind, indicating care providers were likely unaware of participants' assigned interventions.

2.3: NI (80% confidence)

Reason: The article does not provide information on non-protocol interventions.

2.4: N (80% confidence)

Reason: The article does not report failures in implementing the intervention affecting >10% of participants.

2.5: NI (80% confidence)

Reason: The article does not report non-adherence to assigned interventions affecting >10% of participants.

2.6: N (90% confidence)

Reason: The study states analysis was performed on an intention-to-treat basis.

Result of Domain 2: Low risk (85% confidence)

Reason: The study was double-blind and used intention-to-treat analysis, with no clear evidence of deviations from intended interventions.

Domain 3: Missing outcome data

3.1: Y (95% confidence)

Reason: Data appears to be available for nearly all randomized participants, with 18-19 out of 22 participants completing each arm.

3.2: N (90% confidence)

Reason: There is no evidence provided that results were not biased by missing data, and no appropriate methods to handle missing data are described.

3.3: NI (80% confidence)

Reason: The article does not provide information on reasons for missing data in relation to the outcome.

3.4: NI (80% confidence)

Reason: Insufficient information is provided to determine if missingness in the outcome depended on its true value.

Result of Domain 3: Low risk (85% confidence)

Reason: Data was available for nearly all randomized participants, and there is no clear evidence that missing data biased the results.

Domain 4: Risk of bias in measurement of the outcome

4.1: N (90% confidence)

Reason: The methods of measuring outcomes (treatment failure, PEF) appear appropriate.

4.2: N (90% confidence)

Reason: The study was double-blind, suggesting outcome measurement was likely consistent across intervention groups.

4.3: N (90% confidence)

Reason: The study was double-blind, indicating outcome assessors were likely unaware of the intervention received.

4.4: N (85% confidence)

Reason: The primary outcome of treatment failure was based on objective criteria, unlikely to be influenced by knowledge of the intervention.

4.5: N (85% confidence)

Reason: There is no clear evidence that assessment of the outcome was influenced by knowledge of the intervention received.

Result of Domain 4: Low risk (90% confidence)

Reason: The outcome measures were appropriate and assessed in a blinded manner, with low risk of bias in measurement.

Domain 5: Risk of bias in selection of the reported result

5.1: NI (80% confidence)

Reason: The article does not mention a pre-specified analysis plan or trial registration.

5.2: N (85% confidence)

Reason: The reported outcome measures align with those described in the methods, with no evidence of selection based on results.

5.3: N (85% confidence)

Reason: There is no evidence of multiple analyses of the data with selective reporting of results.

Result of Domain 5: Some concerns (80% confidence)

Reason: While there's no clear evidence of selective reporting, the lack of a pre-specified analysis plan raises some concerns.

Overall Bias Risk for the Article: Some concerns (85% confidence)

Reason: While most domains show low risk of bias, the lack of information on a pre-specified analysis plan in Domain 5 raises some concerns. However, the study appears to be well-designed overall with appropriate blinding and outcome measures.

Study 36-2

Based on the information provided in the article, here is my assessment of the risk of bias:

**Article ID**: Rice-McDonald, 2005

**Outcome Name**: Treatment failure

**registration number**: Not reported

Domain 1: Risk of bias arising from the randomization process

1.1: Y (High Certainty - 95%)

Reason: The study is described as randomized with concealed allocation.

1.2: Y (High Certainty - 95%)

Reason: The study used concealed randomization with treatment packs allocated in a concealed randomized order.

1.3: N (Moderate Certainty - 80%)

Reason: No significant baseline differences between groups are reported, suggesting randomization was successful.

Result of Domain 1: Low risk (High Certainty - 95%)

Reason: Proper randomization and allocation concealment were used, with no evidence of baseline imbalances.

Domain 2: Risk of bias due to deviations from the intended interventions

2.1: N (High Certainty - 95%)

Reason: The study is described as double-blind, indicating participants were unaware of their assigned intervention.

2.2: N (High Certainty - 95%)

Reason: The study is described as double-blind, indicating care providers were unaware of participants' assigned intervention.

2.3: NI (Low Certainty - 60%)

Reason: The article does not provide information on non-protocol interventions.

2.4: N (Moderate Certainty - 80%)

Reason: No major implementation failures are reported. Most participants completed their assigned treatment arms.

2.5: NI (Moderate Certainty - 80%)

Reason: No major non-adherence issues are reported.

2.6: N (High Certainty - 95%)

Reason: The study used intention-to-treat analysis, which is not considered an appropriate method to estimate the effect of adhering to the intervention.

Result of Domain 2: Low risk (High Certainty - 90%)

Reason: The study was double-blind with no major deviations from intended interventions reported.

Domain 3: Risk of bias due to missing outcome data

3.1: Y (High Certainty - 95%)

Reason: Outcome data appears to be available for nearly all randomized participants, with only a few dropouts reported.

3.2: N (Moderate Certainty - 80%)

Reason: No evidence is provided that results were not biased by missing data. No correction methods for potential bias are mentioned.

3.3: N (Moderate Certainty - 80%)

Reason: There is no indication that missingness in the outcome depended on its true value.

3.4: N (Moderate Certainty - 80%)

Reason: No evidence suggests that missingness in the outcome likely depended on its true value.

Result of Domain 3: Low risk (High Certainty - 90%)

Reason: Outcome data was nearly complete with no evidence of bias due to missing data.

Domain 4: Risk of bias in measurement of the outcome

4.1: N (High Certainty - 95%)

Reason: The methods of measuring outcomes (treatment failure rates, PEF) appear appropriate and reliable.

4.2: N (High Certainty - 95%)

Reason: The outcome measurement methods were consistent across intervention groups.

4.3: N (High Certainty - 95%)

Reason: The study was double-blind, indicating outcome assessors were unaware of the intervention received.

4.4: N (High Certainty - 90%)

Reason: The primary outcomes (treatment failure, PEF) are relatively objective measures unlikely to be influenced by knowledge of the intervention.

4.5: N (High Certainty - 90%)

Reason: Given the objective nature of the outcomes and blinding, it's unlikely that assessment was influenced by knowledge of the intervention.

Result of Domain 4: Low risk (High Certainty - 95%)

Reason: Outcome measurement was appropriate, consistent, and blinded, with low risk of bias.

Domain 5: Risk of bias in selection of the reported result

5.1: NI (Low Certainty - 50%)

Reason: The article does not mention a pre-specified analysis plan or registration.

5.2: N (Moderate Certainty - 80%)

Reason: There is no evidence of selective reporting of outcomes. All expected outcomes are reported.

5.3: N (Moderate Certainty - 80%)

Reason: There is no evidence of selective reporting from multiple analyses.

Result of Domain 5: Some concerns (Moderate Certainty - 75%)

Reason: While there's no clear evidence of selective reporting, the lack of a pre-specified analysis plan raises some concerns.

Overall Bias Risk for the Article: Some concerns (Moderate Certainty - 80%)

Reason: While most domains show low risk of bias, the lack of information about a pre-specified analysis plan in Domain 5 raises some concerns. However, the study appears to be generally well-conducted with appropriate randomization, blinding, and outcome measurement.

Study 37-1

Based on the provided article and instructions, here is my assessment of the risk of bias:

**Article ID**: Sánchez-Nieto, 2016

**Outcome Name**: Mortality

**Registration number**: Not reported

Domain 1: Risk of bias arising from the randomization process

1.1: NI (75% confidence)

Reason: The article does not describe the specific method of sequence generation.

1.2: NI (75% confidence)

Reason: The article does not mention allocation concealment methods.

1.3: N (90% confidence)

Reason: The article reports no significant differences in baseline characteristics between groups.

Result of Domain 1: Some concerns (80% confidence)

Reason: Allocation concealment is unclear, but baseline differences do not suggest problems with randomization.

Domain 2: Risk of bias due to deviations from intended interventions

2.1: Y (95% confidence)

Reason: Participants were aware of their assigned intervention as this was an open-label study.

2.2: Y (95% confidence)

Reason: Carers and people delivering interventions were aware of participants' assigned intervention.

2.3: NI (75% confidence)

Reason: The article does not provide information on non-protocol interventions.

2.4: N (90% confidence)

Reason: No evidence of substantial implementation failures reported.

2.5: N (90% confidence)

Reason: No evidence of substantial non-adherence reported.

2.6: N (90% confidence)

Reason: The analysis used intention-to-treat principle.

Result of Domain 2: Some concerns (80% confidence)

Reason: Participants and personnel were aware of assigned interventions, but impact on outcomes is unclear.

Domain 3: Missing outcome data

3.1: Y (95% confidence)

Reason: 88.5% of randomized participants completed the study, which is over 95% for the continuous outcome.

3.2: Y (90% confidence)

Reason: Reasons for missing data appear unrelated to outcome.

3.3: N (90% confidence)

Reason: Missingness unlikely to depend on true value based on reported reasons.

3.4: N (90% confidence)

Reason: No evidence that missingness depends on true outcome value.

Result of Domain 3: Low risk (90% confidence)

Reason: Low proportion of missing data and reasons unrelated to true outcome.

Domain 4: Risk of bias in measurement of the outcome

4.1: N (95% confidence)

Reason: Mortality is an objective outcome unlikely to be inappropriately measured.

4.2: N (95% confidence)

Reason: The measurement method was likely comparable between groups for mortality.

4.3: N (95% confidence)

Reason: Mortality is objective and unlikely to be influenced by assessor awareness.

4.4: N (95% confidence)

Reason: Mortality assessment is unlikely to be influenced by intervention knowledge.

4.5: N (95% confidence)

Reason: No evidence that assessment was influenced by intervention knowledge.

Result of Domain 4: Low risk (95% confidence)

Reason: Mortality is an objective outcome unlikely to be biased in measurement.

Domain 5: Risk of bias in selection of the reported result

5.1: NI (75% confidence)

Reason: No information on pre-specified analysis plan or trial registration provided.

5.2: N (90% confidence)

Reason: Unlikely multiple measurements for all-cause mortality.

5.3: N (90% confidence)

Reason: Unlikely multiple analyses for all-cause mortality.

Result of Domain 5: Some concerns (80% confidence)

Reason: Lack of information on pre-specified analysis plan, but low risk of selective reporting for mortality.

Overall Bias Risk for the Article: Some concerns (85% confidence)

Reason: Some concerns in randomization process and selection of reported results, but low risk for missing data and outcome measurement. Mortality as an objective outcome reduces overall risk.

Study 37-2

**Article ID**: Sánchez-Nieto, 2016

**Outcome Name**: Mortality

**Registration number**: Not reported

Domain 1

1.1: NI (90% confidence)

Reason: The article does not report specific methods for generating the random sequence.

1.2: NI (90% confidence)

Reason: No information is provided about allocation concealment methods.

1.3: N (95% confidence)

Reason: Table 1 shows baseline characteristics were similar between groups with no significant differences.

Result of Domain 1: Some concerns (90% confidence)

Reason: Randomization process details are unclear, but baseline characteristics are balanced.

Domain 2

2.1: Y (95% confidence)

Reason: This was an open-label study, so participants were aware of their assigned intervention.

2.2: Y (95% confidence)

Reason: As an open-label study, providers were also aware of participants' assignments.

2.3: NI (90% confidence)

Reason: The article does not provide information on non-protocol interventions.

2.4: Y (90% confidence)

Reason: 11 out of 96 randomized patients (11.5%) did not complete the study, which is more than 10%.

2.5: NI (90% confidence)

Reason: The article does not provide information on adherence to the assigned interventions.

2.6: N (95% confidence)

Reason: The analysis used intention-to-treat, which is not appropriate for estimating per-protocol effects.

Result of Domain 2: High risk (95% confidence)

Reason: Participants and providers were aware of assignments, high dropout rate, and inappropriate analysis methods.

Domain 3

3.1: N (95% confidence)

Reason: 85 out of 96 randomized patients (88.5%) completed the study, which is less than 95%.

3.2: N (90% confidence)

Reason: No methods to correct for potential bias from missing data are reported.

3.3: Y (90% confidence)

Reason: Mortality data could be missing due to loss to follow-up, which may be related to the participants' health status.

3.4: N (85% confidence)

Reason: There's no clear evidence that missingness in mortality outcome depended on its true value.

Result of Domain 3: High risk (90% confidence)

Reason: Incomplete outcome data for mortality, with no methods reported to address potential bias.

Domain 4

4.1: N (95% confidence)

Reason: All-cause mortality is an appropriate and objective measure.

4.2: N (95% confidence)

Reason: Mortality would be measured the same way for both groups.

4.3: N (95% confidence)

Reason: Knowledge of intervention is unlikely to influence assessment of mortality.

4.4: N (95% confidence)

Reason: All-cause mortality is an objective measure.

4.5: N (95% confidence)

Reason: No clear evidence that assessment of mortality was influenced by knowledge of intervention.

Result of Domain 4: Low risk (95% confidence)

Reason: Mortality is an objective outcome not subject to bias in measurement.

Domain 5

5.1: NI (90% confidence)

Reason: No information is provided about pre-specification of the analysis plan.

5.2: N (95% confidence)

Reason: Only one measure of mortality is reported, as expected.

5.3: N (95% confidence)

Reason: There is no indication of multiple analyses of the mortality data.

Result of Domain 5: Some concerns (90% confidence)

Reason: Lack of information on pre-specification of the analysis plan.

Overall Bias Risk for the Article: High risk (95% confidence)

Reason: High risk of bias due to lack of blinding, high dropout rate, and inappropriate analysis methods in Domain 2, as well as high risk due to missing outcome data in Domain 3.

Study 38-1

Based on the information provided in the article, here is my assessment of the risk of bias:

**Article ID**: Titova, 2015

**Outcome Name**: Health-related quality of life

**Registration number**: Not reported

Domain 1: Risk of bias arising from the randomization process

1.1: NI (50% confidence)

Reason: The article does not describe the specific method used for sequence generation.

1.2: NI (50% confidence)

Reason: The article does not mention if or how allocation was concealed.

1.3: N (80% confidence)

Reason: The baseline characteristics between groups were similar, suggesting no major problems with randomization.

Result of Domain 1: Some concerns (75% confidence)

Reason: Allocation concealment was not described, raising some concerns about the randomization process.

Domain 2: Risk of bias due to deviations from intended interventions

2.1: Y (90% confidence)

Reason: This was an open study, so participants were aware of their assigned intervention.

2.2: Y (90% confidence)

Reason: As an open study, care providers were also aware of participants' assigned interventions.

2.3: NI (50% confidence)

Reason: The article does not provide information on non-protocol interventions.

2.4: N (80% confidence)

Reason: There is no indication of substantial implementation failures.

2.5: N (80% confidence)

Reason: The article does not report significant non-adherence issues.

2.6: N (90% confidence)

Reason: The analysis appears to be intention-to-treat, which is not an appropriate method to estimate the effect of adhering to intervention.

Result of Domain 2: High risk (80% confidence)

Reason: Participants and providers were aware of assigned interventions, and an inappropriate analysis method was used.

Domain 3: Risk of bias due to missing outcome data

3.1: N (90% confidence)

Reason: Only 58% of included patients were available for evaluation after 2 years.

3.2: N (80% confidence)

Reason: No evidence is provided that results were not biased by missing data.

3.3: Y (80% confidence)

Reason: Missingness likely depends on true values, as those who died during follow-up had more severe baseline characteristics.

3.4: Y (80% confidence)

Reason: Missingness is likely related to true values, given the differences in baseline characteristics of those who died.

Result of Domain 3: High risk (85% confidence)

Reason: Substantial missing data that likely depends on true values.

Domain 4: Risk of bias in measurement of the outcome

4.1: NI (50% confidence)

Reason: The article does not provide details on how health-related quality of life was measured.

4.2: NI (50% confidence)

Reason: Without information on the measurement method, it's unclear if it differed between groups.

4.3: Y (80% confidence)

Reason: For health-related quality of life, assessors (likely the patients themselves) were aware of the intervention received.

4.4: Y (90% confidence)

Reason: Health-related quality of life is a subjective outcome that could be influenced by knowledge of the intervention.

4.5: Y (80% confidence)

Reason: As a subjective outcome, assessment of health-related quality of life is likely to be influenced by knowledge of the intervention.

Result of Domain 4: High risk (80% confidence)

Reason: The outcome is subjective and likely influenced by knowledge of the intervention.

Domain 5: Risk of bias in selection of the reported result

5.1: NI (50% confidence)

Reason: The article does not mention a pre-specified analysis plan or trial registration.

5.2: NI (50% confidence)

Reason: Without a pre-specified plan, it's unclear if there was selection from multiple outcome measurements.

5.3: NI (50% confidence)

Reason: Without a pre-specified plan, it's unclear if there was selection from multiple analyses.

Result of Domain 5: Some concerns (70% confidence)

Reason: Lack of information on a pre-specified analysis plan raises some concerns.

Overall Bias Risk for the Article: High risk (85% confidence)

Reason: High risk of bias in Domains 2, 3, and 4, with some concerns in Domains 1 and 5.

Study 38-2

Based on the information provided in the article, here is my assessment of the risk of bias:

**Article ID**: Titova, 2015

**Outcome Name**: Health-related quality of life

**registration number**: Not reported

Domain 1: Risk of bias arising from the randomization process

1.1: NI (80% confidence)

Reason: The article does not describe the specific method used to generate the allocation sequence.

1.2: N (90% confidence)

Reason: The article states that allocation was based on the patients' address of permanent residence, which does not conceal the allocation.

1.3: N (80% confidence)

Reason: There were no significant differences in baseline characteristics between the intervention and control groups.

Result of Domain 1: High risk (90% confidence)

Reason: The allocation sequence was not concealed (1.2 is N).

Domain 2: Risk of bias due to deviations from intended interventions

2.1: Y (90% confidence)

Reason: Participants were aware of their assigned intervention as this was an open study.

2.2: Y (90% confidence)

Reason: Care providers were aware of participants' assigned intervention as this was an open study.

2.3: NI (80% confidence)

Reason: The article does not provide information on non-protocol interventions.

2.4: N (80% confidence)

Reason: There is no indication of substantial implementation failures.

2.5: NI (80% confidence)

Reason: The article does not report non-adherence rates exceeding 10%.

2.6: NI (80% confidence)

Reason: The article does not provide information on the analysis method for HRQoL.

Result of Domain 2: Some concerns (80% confidence)

Reason: Participants and providers were aware of assignment, and there's insufficient information on the analysis method.

Domain 3: Risk of bias due to missing outcome data

3.1: NI (80% confidence)

Reason: The article does not provide specific information on HRQoL data completeness.

3.2: N (80% confidence)

Reason: No evidence that results for HRQoL were unbiased by missing data.

3.3: PY (70% confidence)

Reason: Missingness might be related to health status and thus to HRQoL.

3.4: NI (70% confidence)

Reason: Insufficient information to determine if missingness in HRQoL depended on its true value.

Result of Domain 3: Some concerns (75% confidence)

Reason: Insufficient information on missing HRQoL data and potential reasons for missingness.

Domain 4: Risk of bias in measurement of the outcome

4.1: NI (70% confidence)

Reason: The article does not specify the HRQoL measurement tool used.

4.2: NI (70% confidence)

Reason: Insufficient information on how HRQoL was measured in each group.

4.3: Y (80% confidence)

Reason: HRQoL is typically a patient-reported outcome, and patients were aware of their intervention.

4.4: Y (90% confidence)

Reason: HRQoL is a subjective measure likely to be influenced by knowledge of the intervention received.

4.5: PY (80% confidence)

Reason: As a subjective measure, HRQoL assessment was likely influenced by knowledge of the intervention.

Result of Domain 4: High risk (85% confidence)

Reason: HRQoL is a subjective measure likely influenced by knowledge of the intervention.

Domain 5: Risk of bias in selection of the reported result

5.1: NI (80% confidence)

Reason: No information on pre-specified analysis plan for HRQoL.

5.2: NI (80% confidence)

Reason: Insufficient information on HRQoL measurement to determine if multiple eligible measurements were taken.

5.3: NI (80% confidence)

Reason: Insufficient information on HRQoL analysis to determine if multiple eligible analyses were conducted.

Result of Domain 5: Some concerns (80% confidence)

Reason: Lack of information on pre-specified analysis plan and potential multiple measurements or analyses.

Overall Bias Risk for the Article: High risk (85% confidence)

Reason: High risk of bias in Domains 1 and 4, with some concerns in Domains 2, 3, and 5.

Study 39-1

I apologize for the misunderstanding. Let me reassess the risk of bias for the correct outcome:

**Article ID**: Walters, 2013

**Outcome Name**: Respiratory-related hospital admissions

**Registration number**: ACTR12608000112369

Domain 1: Risk of bias arising from the randomization process

1.1: Y (95% confidence)

Reason: The study states "Randomisation: By random numbers code, block stratified on location"

1.2: Y (95% confidence)

Reason: The study states "allocation by sequentially numbered, opaque and sealed envelopes"

1.3: N (90% confidence)

Reason: Table 1 shows baseline characteristics were generally similar between groups, with only smoking history significantly different

Result of Domain 1: Low risk (95% confidence)

Reason: Allocation sequence was adequately generated and concealed, and baseline differences do not suggest a problem with randomization

Domain 2: Risk of bias due to deviations from intended interventions

2.1: Y (90% confidence)

Reason: Participants were likely aware of their assigned intervention as blinding was not mentioned

2.2: Y (90% confidence)

Reason: Care providers (nurses) were aware of participants' assigned intervention

2.3: NI (80% confidence)

Reason: No information provided on non-protocol interventions

2.4: N (80% confidence)

Reason: No information suggesting substantial implementation failures

2.5: N (80% confidence)

Reason: No specific information is reported regarding adherence

2.6: N (90% confidence)

Reason: Analysis was intention-to-treat

Result of Domain 2: Some concerns (85% confidence)

Reason: Participants and providers were aware of assigned interventions, but no evidence of substantial deviations from intended interventions

Domain 3: Missing outcome data

3.1: Y (95% confidence)

Reason: Data on hospital admissions were likely available for all or nearly all participants as they were retrieved from electronic records

3.2: NA

Reason: Not applicable as there is likely no missing outcome data

3.3: NA

Reason: Not applicable as there is likely no missing outcome data

3.4: NA

Reason: Not applicable as there is likely no missing outcome data

Result of Domain 3: Low risk (95% confidence)

Reason: Hospital admission data likely complete from electronic records

Domain 4: Measurement of the outcome

4.1: N (95% confidence)

Reason: Hospital admissions are an objective measure retrieved from electronic records

4.2: N (95% confidence)

Reason: Measurement method (electronic records) would not differ between groups

4.3: N (95% confidence)

Reason: Outcome assessors (those retrieving data from electronic records) were likely unaware of intervention assignment

4.4: N (95% confidence)

Reason: As an objective measure from records, assessment is unlikely to be influenced by knowledge of intervention

4.5: N (95% confidence)

Reason: No evidence that assessment was influenced by knowledge of intervention

Result of Domain 4: Low risk (95% confidence)

Reason: Objective outcome from electronic records, unlikely to be biased by knowledge of intervention

Domain 5: Selection of the reported result

5.1: Y (90% confidence)

Reason: Study was registered and outcomes align with protocol

5.2: N (90% confidence)

Reason: Reported results correspond to intended measurements

5.3: N (90% confidence)

Reason: No indication of selection of results based on multiple analyses

Result of Domain 5: Low risk (90% confidence)

Reason: Analysis appears to follow pre-specified plan without selection of results

Overall Bias Risk for the Article: Some concerns (85% confidence)

Reason: Some concerns due to lack of blinding of participants and providers, but low risk for other domains, particularly for this objective outcome measure

Study 39-2

I apologize for the misunderstanding. I'll reassess the study based on the correct outcome of respiratory-related hospital admissions. Thank you for the clarification.

**Article ID**: Walters, 2013

**Outcome Name**: Respiratory-related hospital admissions

**Registration number**: ACTR12608000112369

Domain 1

1.1: Y (100% confidence)

Reason: The study used "random numbers code" for randomization.

1.2: Y (100% confidence)

Reason: Allocation was concealed using "sequentially numbered, opaque and sealed envelopes".

1.3: N (100% confidence)

Reason: Baseline characteristics were generally similar between groups, with only smoking history differing significantly.

Result of the domain: Low risk (100% confidence)

Reason: Adequate randomization and allocation concealment, with no concerning baseline imbalances.

Domain 2

2.1: Y (100% confidence)

Reason: Participants were aware of their assigned intervention.

2.2: Y (100% confidence)

Reason: Health mentors delivering the intervention were aware of participants' assignments.

2.3: NI (100% confidence)

Reason: No information provided on non-protocol interventions.

2.4: Y (100% confidence)

Reason: More than 10% of the participants (12%) did not receive the allocated intervention.

2.5: NI (100% confidence)

Reason: No information about non-adherence.

2.6: N (100% confidence)

Reason: Analysis appears to be intention-to-treat.

Result of the domain: High risk (100% confidence)

Reason: Participants and personnel were aware of assignments, and there were substantial deviations from intended interventions for more than 10% of participants.

Domain 3

3.1: Y (100% confidence)

Reason: Data on hospital admissions were retrieved from electronic records, likely available for all or nearly all participants.

3.2: Y (100% confidence)

Reason: Use of electronic records suggests complete data, minimizing risk of bias from missing data.

3.3: N (100% confidence)

Reason: Missingness unlikely as data were retrieved from electronic records.

3.4: N (100% confidence)

Reason: Not applicable as data were likely complete.

Result of the domain: Low risk (100% confidence)

Reason: Complete outcome data likely available from electronic records.

Domain 4

4.1: N (100% confidence)

Reason: Hospital admissions data from electronic records is an appropriate measure.

4.2: N (100% confidence)

Reason: The outcome was likely measured consistently across groups using electronic records.

4.3: N (100% confidence)

Reason: Outcome assessors (electronic system) were unaware of intervention assignment.

4.4: N (100% confidence)

Reason: Assessment of hospital admissions from records is objective and unlikely to be influenced by knowledge of intervention.

4.5: N (100% confidence)

Reason: No clear evidence that assessment was influenced by intervention knowledge.

Result of the domain: Low risk (100% confidence)

Reason: Objective outcome measure from electronic records, unlikely to be influenced by knowledge of intervention.

Domain 5

5.1: Y (100% confidence)

Reason: The study was registered and outcomes appear to be reported as pre-specified.

5.2: N (100% confidence)

Reason: Reported results correspond to intended measurements.

5.3: N (100% confidence)

Reason: No evidence of multiple analyses being conducted.

Result of the domain: Low risk (100% confidence)

Reason: Outcome reporting appears complete and in line with pre-specified plans.

Overall Bias Risk for the Article: High risk (100% confidence)

Study 40-1

Here is my assessment of the risk of bias for the article based on the instructions provided:

**Article ID**: Wang, 2020

**Outcome Name**: Health-related quality of life

**registration number**: Not reported

Domain 1

1.1: NI (75% confidence)

Reason: The article does not describe any specific methods of sequence generation.

1.2: Y (95% confidence)

Reason: The article states "The allocation sequence was generated and released to the interventionist on a case-by-case basis by an independent department that specialized in supplying randomly generated sequences for research."

1.3: N (90% confidence)

Reason: The article reports no significant differences in baseline characteristics between the two groups.

Result of Domain 1: Low risk (80% confidence)

Reason: Allocation was adequately concealed (1.2 is Y) and there were no baseline differences suggesting problems with randomization (1.3 is N).

Domain 2

2.1: Y (95% confidence)

Reason: The article states this was a single-blind study, implying participants were aware of their assigned intervention.

2.2: Y (95% confidence)

Reason: As a single-blind study, the nurses delivering the intervention were aware of participants' assignments.

2.3: NI (80% confidence)

Reason: The article does not provide information on non-protocol interventions.

2.4: N (90% confidence)

Reason: The article reports an attrition rate of 7.14%, which is less than 10%.

2.5: NI (90% confidence)

Reason: The article does not report non-adherence rates exceeding 10%.

2.6: N (95% confidence)

Reason: The article states "An intention-to-treat principle was applied to all analyses."

Result of Domain 2: Some concerns (80% confidence)

Reason: Participants and personnel were aware of assigned interventions, but there is no evidence of substantial deviations from intended interventions.

Domain 3

3.1: Y (95% confidence)

Reason: The article reports 143 out of 154 participants (92.9%) completed the 12-month study.

3.2: N (90% confidence)

Reason: No evidence of methods to correct for bias due to missing data.

3.3: N (85% confidence)

Reason: There is no indication that missingness depended on the true value of the outcome.

3.4: N (85% confidence)

Reason: There is no evidence suggesting missingness in the outcome depended on its true value.

Result of Domain 3: Low risk (90% confidence)

Reason: Data was available for nearly all randomized participants and an appropriate analysis was used to handle missing data.

Domain 4

4.1: N (95% confidence)

Reason: The St George Respiratory Questionnaire (SGRQ) is described as a valid, reliable instrument for assessing health-related quality of life in COPD patients.

4.2: N (90% confidence)

Reason: The article does not indicate any differences in measurement methods between groups.

4.3: Y (95% confidence)

Reason: As a single-blind study, outcome assessors were likely aware of intervention assignments.

4.4: Y (95% confidence)

Reason: The SGRQ is a subjective measure reported by patients who were aware of their group assignment.

4.5: N (80% confidence)

Reason: While the outcome is subjective, there is no clear evidence that assessment was influenced by knowledge of the intervention received.

Result of Domain 4: Some concerns (85% confidence)

Reason: While the measurement method was appropriate, outcome assessors were aware of intervention assignments for this subjective outcome.

Domain 5

5.1: NI (90% confidence)

Reason: The article does not mention pre-registration or a pre-specified analysis plan.

5.2: N (85% confidence)

Reason: The reported results for the SGRQ appear to correspond to all intended outcome measurements described in the methods.

5.3: N (85% confidence)

Reason: There is no evidence of multiple eligible analyses of the data.

Result of Domain 5: Some concerns (85% confidence)

Reason: While there is no evidence of selective reporting, the lack of a pre-specified analysis plan raises some concerns.

Overall Bias Risk for the Article: Some concerns (85% confidence)

Reason: While some domains were assessed as low risk, there were some concerns in multiple domains, particularly regarding blinding and the lack of a pre-specified analysis plan.

Study 40-2

Based on the information provided in the article, here is my assessment of the risk of bias for the specified outcome:

**Article ID**: Wang, 2020

**Outcome Name**: Health-related quality of life

**Registration number**: Not reported

Domain 1: Risk of bias arising from the randomization process

1.1: NI (75% certainty)

Reason: The article does not provide specific details about the randomization sequence generation method.

1.2: Y (95% certainty)

Reason: The article states "The allocation sequence was generated and released to the interventionist on a case-by-case basis by an independent department that specialized in supplying randomly generated sequences for research."

1.3: N (90% certainty)

Reason: The article reports "There were no significant differences in demographic and clinical data between the two groups at baseline (Table 1)."

Result of Domain 1: Low risk (80% certainty)

Reason: While the randomization method is unclear, allocation concealment was adequate and baseline differences do not suggest problems with randomization.

Domain 2: Risk of bias due to deviations from intended interventions

2.1: Y (95% certainty)

Reason: The intervention involved face-to-face education sessions, so participants were aware of their assigned intervention.

2.2: Y (95% certainty)

Reason: The nurses delivering the intervention were aware of participants' assignments.

2.3: NI (70% certainty)

Reason: The article does not provide information about non-protocol interventions.

2.4: N (80% certainty)

Reason: The article reports an attrition rate of 7.14%, which is less than 10%.

2.5: NI (80% certainty)

Reason: The article does not report non-adherence issues.

2.6: N (90% certainty)

Reason: The article states "An intention-to-treat principle was applied to all analyses."

Result of Domain 2: Some concerns (75% certainty)

Reason: While participants and personnel were aware of assignments, there's no evidence of substantial deviations from intended interventions.

Domain 3: Missing outcome data

3.1: Y (90% certainty)

Reason: The study reports a low attrition rate of 7.14%, indicating data was available for nearly all randomized participants.

3.2: N (85% certainty)

Reason: No evidence of methods to correct for bias due to missing data.

3.3: N (80% certainty)

Reason: With low attrition and use of intention-to-treat analysis, it's unlikely that missingness depends on the true outcome value.

3.4: N (80% certainty)

Reason: Given the low attrition rate and analysis method, it's unlikely that missingness depends on the true outcome value.

Result of Domain 3: Low risk (85% certainty)

Reason: There was low attrition and appropriate analysis methods were used to handle missing data.

Domain 4: Risk of bias in measurement of the outcome

4.1: N (90% certainty)

Reason: The study used the St George Respiratory Questionnaire (SGRQ), which is described as a valid, sensitive and reliable instrument for measuring HRQoL in COPD patients.

4.2: N (85% certainty)

Reason: The article does not indicate any differences in outcome measurement between groups.

4.3: Y (95% certainty)

Reason: The SGRQ is a patient-reported outcome, and participants were aware of their intervention assignment.

4.4: Y (90% certainty)

Reason: As a subjective patient-reported measure, assessment could be influenced by knowledge of the intervention received.

4.5: N (75% certainty)

Reason: While there's potential for bias, there's no clear evidence that assessment was actually influenced by knowledge of the intervention.

Result of Domain 4: Some concerns (80% certainty)

Reason: While the measurement tool is appropriate, the subjective nature of the outcome and participants' awareness of intervention could introduce some bias.

Domain 5: Risk of bias in selection of the reported result

5.1: NI (90% certainty)

Reason: The article does not mention pre-registration or a pre-specified analysis plan.

5.2: N (85% certainty)

Reason: The reported results for SGRQ appear to include all subscales and the total score, suggesting no selective reporting of favorable results.

5.3: N (85% certainty)

Reason: The analysis methods appear consistent between the methods and results sections, with no evidence of selective reporting based on results.

Result of Domain 5: Some concerns (85% certainty)

Reason: While there's no clear evidence of selective reporting, the lack of a pre-specified analysis plan raises some concerns.

Overall Bias Risk for the Article: Some concerns (80% certainty)

Reason: While randomization and handling of missing data appear adequate, there are some concerns regarding blinding and the potential for bias in the subjective outcome measure. The lack of a pre-registered analysis plan also raises some concerns about selective reporting.

Appendix 3. Supplementary table

Table S1 Included Cochrane Reviews list

| Title | Number of included RCTs |
| --- | --- |
| (Ultra‐)long‐acting insulin analogues for people with type 1 diabetes mellitus | 26 |
| Addition of long‐acting beta2 agonists or long‐acting muscarinic antagonists versus doubling the dose of inhaled corticosteroids (ICS) in adolescents and adults with uncontrolled asthma with medium dose ICS: a systematic review and network meta‐analysis | 35 |
| Altering the availability or proximity of food, alcohol, and tobacco products to change their selection and consumption | 23 |
| Antibiotics for the treatment of COVID‐19 | 10 |
| Antibiotics versus placebo for acute bacterial conjunctivitis | 21 |
| Anticholinergic deprescribing interventions for reducing risk of cognitive decline or dementia in older adults with and without prior cognitive impairment | 3 |
| Antioxidants to prevent respiratory decline in people with Duchenne muscular dystrophy and progressive respiratory decline | 2 |
| Antiplatelet agents for the treatment of adults with COVID‐19 | 6 |
| Antipsychotic dose reduction compared to dose continuation for people with schizophrenia | 22 |
| Antipsychotic polypharmacy reduction versus polypharmacy continuation for people with schizophrenia | 5 |
| Cannabis and cannabinoids for symptomatic treatment for people with multiple sclerosis | 25 |
| Cleavage‐stage versus blastocyst‐stage embryo transfer in assisted reproductive technology | 32 |
| Colchicine for the treatment of COVID‐19 | 4 |
| Corticosteroid implants for chronic non‐infectious uveitis | 4 |
| Different types of percutaneous endovascular interventions for acute ischemic stroke | 2 |
| Digital technology for monitoring adherence to inhaled therapies in people with cystic fibrosis | 2 |
| Educational, supportive and behavioural interventions to improve usage of continuous positive airway pressure machines in adults with obstructive sleep apnoea | 41 |
| Effectiveness and tolerability of dual and triple combination inhaler therapies compared with each other and varying doses of inhaled corticosteroids in adolescents and adults with asthma: a systematic review and network meta‐analysis | 17 |
| Effects of a gluten‐reduced or gluten‐free diet for the primary prevention of cardiovascular disease | 1 |
| Efficacy and safety of COVID‐19 vaccines | 41 |
| Electromagnetic‐guided versus endoscopic‐guided postpyloric placement of nasoenteral feeding tubes | 4 |
| Exercise for acutely hospitalised older medical patients | 24 |
| Exercise‐based cardiac rehabilitation for adults after heart valve surgery | 6 |
| Fecal microbiota transplantation for the treatment of recurrent Clostridioides difficile (Clostridium difficile) | 6 |
| First‐line therapy for adults with advanced renal cell carcinoma: a systematic review and network meta‐analysis | 55 |
| Fluvoxamine for the treatment of COVID‐19 | 2 |
| High flow nasal cannula for respiratory support in term infants | 6 |
| Higher versus lower fractions of inspired oxygen or targets of arterial oxygenation for adults admitted to the intensive care unit | 7 |
| House modifications for preventing malaria | 7 |
| Hyperimmune immunoglobulin for people with COVID‐19 | 5 |
| Hypothermia for neuroprotection in adults after cardiac arrest | 12 |
| Implanted spinal neuromodulation interventions for chronic pain in adults | 15 |
| Inhaled corticosteroids for the treatment of COVID‐19 | 3 |
| Inspiratory muscle training, with or without concomitant pulmonary rehabilitation, for chronic obstructive pulmonary disease (COPD) | 25 |
| Interleukin‐6 blocking agents for treating COVID‐19: a living systematic review | 49 |
| Interventions for myopia control in children: a living systematic review and network meta‐analysis | 17 |
| Interventions from pregnancy to two years after birth for parents experiencing complex post‐traumatic stress disorder and/or with childhood experience of maltreatment | 67 |
| Interventions in outside‐school hours childcare settings for promoting physical activity amongst schoolchildren aged 4 to 12 years | 9 |
| Ivermectin for preventing and treating COVID‐19 | 11 |
| Janus kinase inhibitors for the treatment of COVID‐19 | 6 |
| Low‐carbohydrate versus balanced‐carbohydrate diets for reducing weight and cardiovascular risk | 61 |
| Mammography in combination with breast ultrasonography versus mammography for breast cancer screening in women at average risk | 10 |
| Mental Health First Aid as a tool for improving mental health and well‐being | 3 |
| Mindfulness‐based psychological interventions for improving mental well‐being in medical students and junior doctors | 10 |
| Nirmatrelvir combined with ritonavir for preventing and treating COVID‐19 | 2 |
| Non‐biologic, steroid‐sparing therapies for non‐infectious intermediate, posterior, and panuveitis in adults | 11 |
| Nutritional interventions for preventing and treating pressure ulcers | 33 |
| Pain management for medical abortion before 14 weeks’ gestation | 4 |
| Palivizumab for preventing severe respiratory syncytial virus (RSV) infection in children | 5 |
| Pars plana vitrectomy with internal limiting membrane flap versus pars plana vitrectomy with conventional internal limiting membrane peeling for large macular hole | 4 |
| PEG‐asparaginase treatment regimens for acute lymphoblastic leukaemia in children: a network meta‐analysis | 7 |
| Perioperative enhanced recovery programmes for women with gynaecological cancers | 7 |
| Peripheral nerve blocks for hip fractures in adults | 43 |
| Pharmacological interventions for self‐harm in adults | 7 |
| Primary‐level and community worker interventions for the prevention of mental disorders and the promotion of well‐being in low‐ and middle‐income countries | 67 |
| Progestogens for prevention of luteinising hormone (LH) surge in women undergoing controlled ovarian hyperstimulation as part of an assisted reproductive technology (ART) cycle | 17 |
| Physical activity interventions for people with congenital heart disease | 14 |
| Physical exercise for people with Parkinson’s disease: a systematic review and network meta‐analysis | 15 |
| Psychological interventions for depression in adolescent and adult congenital heart disease | 3 |
| Psychosocial interventions for self‐harm in adults | 76 |
| Re‐feeding versus discarding gastric residuals to improve growth in preterm infants | 0 |
| Remdesivir for the treatment of COVID‐19 | 55 |
| Rho kinase inhibitor for primary open‐angle glaucoma and ocular hypertension | 15 |
| Routine monitoring of gastric residual for prevention of necrotising enterocolitis in preterm infants | 5 |
| SARS‐CoV‐2‐neutralising monoclonal antibodies for treatment of COVID‐19 | 6 |
| SARS‐CoV‐2‐neutralising monoclonal antibodies to prevent COVID‐19 | 4 |
| Serenoa repens for the treatment of lower urinary tract symptoms due to benign prostatic enlargement | 22 |
| Skin care interventions in infants for preventing eczema and food allergy | 33 |
| Social network interventions to support cardiac rehabilitation and secondary prevention in the management of people with heart disease | 54 |
| Systemic corticosteroids for the treatment of COVID‐19: Equity‐related analyses and update on evidence | 16 |
| Systemic interventions for treatment of Stevens‐Johnson syndrome (SJS), toxic epidermal necrolysis (TEN), and SJS/TEN overlap syndrome | 1 |
| Telerehabilitation for people with low vision | 1 |
| The combination of transcatheter arterial chemoembolisation (TACE) and thermal ablation versus TACE alone for hepatocellular carcinoma | 0 |
| The effect of time spent in rehabilitation on activity limitation and impairment after stroke | 23 |
| Therapeutics for treating mpox in humans | 0 |
| Topical corticosteroids for dry eye | 22 |
| Topical ophthalmic anesthetics for corneal abrasions | 9 |
| Tumor necrosis factor (TNF) inhibitors for juvenile idiopathic arthritis‐associated uveitis | 3 |
| Treatments for Morton's neuroma | 6 |
| Ultrafiltration for acute heart failure | 14 |
| Vitamin D supplementation for the treatment of COVID‐19: a living systematic review | 3 |
| Vocabulary interventions for second language (L2) learners up to six years of age | 11 |
| Self‐management interventions for people with chronic obstructive pulmonary disease | 26 |
| Increased versus stable doses of inhaled corticosteroids for exacerbations of chronic asthma in adults and children | 6 |
| Mental Health First Aid as a tool for improving mental health and well‐being | 3 |
| Digital technology for delivering and monitoring exercise programs for people with cystic fibrosis | 4 |
| Title | Number of included RCTs |

Table S2. Characteristics of included RCTs

| Name, year | Popation | Intervation | Comparison |
| --- | --- | --- | --- |
| Pressler, 2016 | Patients after transcatheter aortic valve implantation | Exercise | Usual care |
| Ireland, 2017 | Intern medical practitioners | Mindfulness | Active control |
| Beigel 2020 | Covid-19 | Remdesivir | Placebo |
| Hu 2020 | Hospitalized older patients | Simplified reablement program | Usual care |
| Novotna, 2011 | Multiple sclerosis spasticity | Nabiximols | Placebo |
| Bart, 2005 | Hospitalized Patients With Heart Failure | Ultrafiltration | Usual care |
| Pinto‐Fraga, 2016 | Dry eye disease | Fluorometholone | Polyvinyl alcohol |
| Dissanayake 2019 | Atopic Dermatitis or Food Allergy | Combination of synbiotics and skincare; Synbiotics; Skincare | No intervention |
| Frattarelli, 2003 | Patients who initiated IVF | Day 5 Embryo Transfer | Day 3 Embryo Transfer |
| Griffiths 2019 | Adolescents | Mentalization-based group therapy | Usual care |
| Horby 2021 | Covid-19 | Dexamethasone | Placebo |
| Beautrais, 2010 | Patients presented to the psychiatric emergency service following self-harm or attempted suicide | Postcard intervention | Usual care |
| Huhn 2020 | Chronic schizophrenia or schizoafective disorder | Individualized antipsychotic dose reduction | Continuous maintenance treatment |
| Ooi, 2021 | Overweigh tor Obesity | Branched-chain amino acid supplementation | Placebo |
| Kawanishi, 2014 | Mental health problems who had attempted suicide | Assertive case management | Usual care |
| Liljeroos, 2012 | Heart Failure | Integrated Educational and psychosocial Intervention in Patient-Partner | Usual care |
| Tauber, 2011 | Bacterial Conjunctivitis | Moxifloxacin ophthalmic solution | Vehicle |
| Carretero, 2008 | Normally nourished or mildly undernourished geriatric Patients | Oral Nutritional Supplements | No intervention |
| Van Anholt, 2010 | Non-malnourished patients | Specific nutritional support | Placebo |
| Gonzalez‐Ortega, 2021 | First-episode psychosis | Cognitive Behavioural Therapy | Usual care |
| Bischoff, 2012 | Chronic obstructive pulmonary disease | Self management; Routine monitoring | Usual care |
| Coultas, 2005 | Chronic obstructive pulmonary disease | Nurse-assisted medical management; Nurse-assisted collaborative management | Usual care |
| Curran, 2022 | Cystic Fibrosis | Wearable technology, goal setting and text message feedback | Wearable technology only |
| Fan, 2012 | Chronic obstructive pulmonary disease | Comprehensive care management program | Usual care |
| FitzGerald, 2004 | Asthma exacerbations | Doubling the dose of budesonide | Continued maintenance dose of budesonide |
| Harrison, 2004 | Asthma exacerbations | Active inhaler corticosteroid | Placebo inhaler corticosteroid |
| Hernández, 2015 | Chronic obstructive pulmonary disease | Community-based integrated care | Usual care |
| Johnson‐Warrington, 2016 | Chronic obstructive pulmonary disease | Supported self-management program | Usual care |
| Jorm, 2010 | General community | an e-learning CD, read a Mental Health First Aid manual | Waiting list |
| Kessler, 2018 | Chronic obstructive pulmonary disease | multicomponent home-based disease-management programme | Usual care |
| Lenferink, 2019 | Chronic obstructive pulmonary disease | Patient-tailored self-management | Usual care |
| Liang, 2019 | Chronic obstructive pulmonary disease | The RADICALS model of care | Usual care |
| Mitchell, 2014 | Chronic obstructive pulmonary disease | SPACE FOR COPD | Usual care |
| Oborne, 2009 | Asthma | Active corticosteroid inhaler | Placebo corticosteroid inhaler |
| Rice, 2010 | Chronic obstructive pulmonary disease | Disease management | Usual care |
| Rice‐McDonald, 2005 | Asthma | Active corticosteroid inhaler | Placebo corticosteroid inhaler |
| Sanchez‐Nieto, 2016 | Chronic obstructive pulmonary disease | Self-management plan | Usual care |
| Titova, 2015 | Chronic obstructive pulmonary disease | Integrated care | Usual care |
| Walters, 2013 | Chronic obstructive pulmonary disease | Health mentoring | Usual care |
| Wang, 2019 | Chronic obstructive pulmonary disease | Nurse-led self-management program | Usual care |
