# Supplementary figures and images for: Large Language Model–Assisted Risk-of-Bias Assessment in Randomized Controlled Trials Using the Revised Risk-of-Bias Tool: Evaluation Study
Source: J Med Internet Res. 2025 Jun 24;27:e70450. doi: 10.2196/70450 (PMC12238788; doi:10.2196/70450)

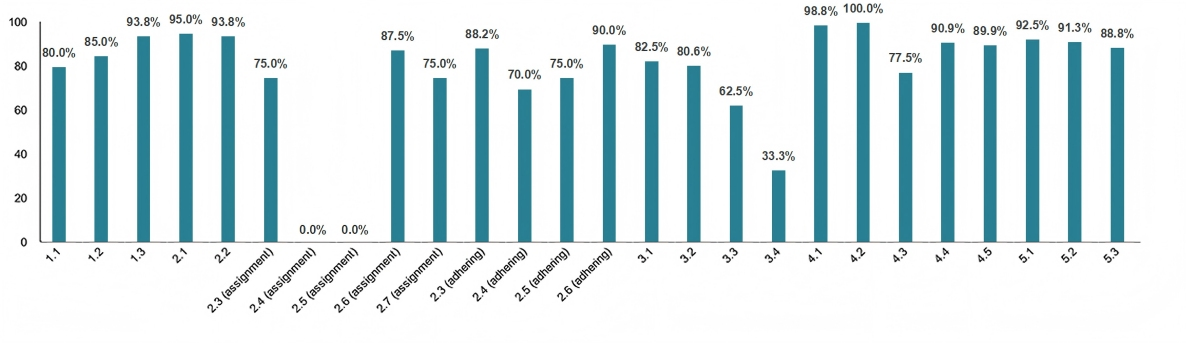

Supplement: Multimedia Appendix 2 [file jmir_v27i1e70450_app2.png]
